# Charge–Transfer Complexes and Fluorescence Modulation in Amide- and Carboxy-Substituted 2‑Phenyl-1,3,2-benzodiazaboroles

**DOI:** 10.1021/acsomega.5c07928

**Published:** 2026-01-19

**Authors:** Julius Green, Bradley Blake, Alexander Rash

**Affiliations:** Chemistry Department, State University of New York at Cortland, P.O. Box 2000, Cortland, New York 13045, United States

## Abstract

A library of amide- and carboxy- functionalized 2-phenyl-1,3,2-benzodiazaborole derivatives was synthesized via microwave-assisted cyclic condensation to explore the effects of pseudoaromaticity on charge-transfer complex (CTC) formation and photophysical behavior. All compounds were characterized by NMR, IR, UV–vis, and fluorescence spectroscopy. While absorbance profiles remained consistent (λ_max_ = 298 – 324 nm), several derivatives exhibited strong bathochromic emission (λ_em_ = 363 – 555 nm) and exceptionally large Stokes shifts (Δ*v* > 150 nm), particularly those bearing –OCH_3_ groups. Red-shifted fluorescence and DFT calculations suggest antiparallel dimeric CTCs stabilized by B–N delocalization. These results highlight amide- and carboxy- 1,3,2-benzodiazaborole frameworks as tunable pseudoaromatic systems with potential applications in optical sensing and functional material design.

## Graphical abstract

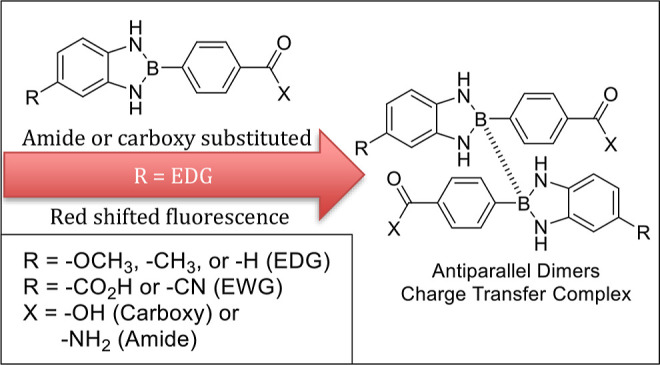

## 1 Introduction

### 1.1 Pseudoaromaticity

Pseudoaromaticity is a crucial concept in modern chemistry that enables the rational design and understanding of molecules with partial π-delocalization, offering insights into atypical bonding, reactivity, and electronic properties beyond classical aromatic systems.^1^ The concept of pseudoaromaticity emerged in the late 1950s through the pioneering work of David P. Craig, who proposed that certain compounds, despite sharing some features with aromatic systems, lack fully symmetrical valence bond descriptions in their ground states.^1,2^ This distinction was formally recognized at the 1971 Jerusalem Symposium on Aromaticity, Pseudoaromaticity, and Antiaromaticity, establishing pseudoaromaticity as an intermediate category between classical aromaticity and nonaromatic character.^1^

Unlike classical aromatic systems governed by Hückel’s 4n + 2 rule, which exhibit extensive π-delocalization and uniform bond lengths, pseudoaromatic compounds often show bond-length alternation, partial π-delocalization, and lower resonance stabilization energies (Table 1).^3^ These systems often exhibit magnetic ring currents and planar structures but deviate from traditional criteria, allowing tunable electronic behaviors that are responsive to structural and environmental changes. The formalization of the definition of pseudoaromaticity has led to reclassification of some aromatic compounds; for example, work by Bertelli and Andrews, and later Gleiter and Haberhauer, led to reclassification of tropones and tropolones as pseudoaromatic due to their behavior resembling polyenone structures and limited π-electron delocalization (Figure 1).^4,5^ Within this domain, the 2-phenyl-1,3,2-benzodiazaborole (00) scaffold has emerged as a versatile pseudoaromatic platform (Figure 1).

**Table 1:**
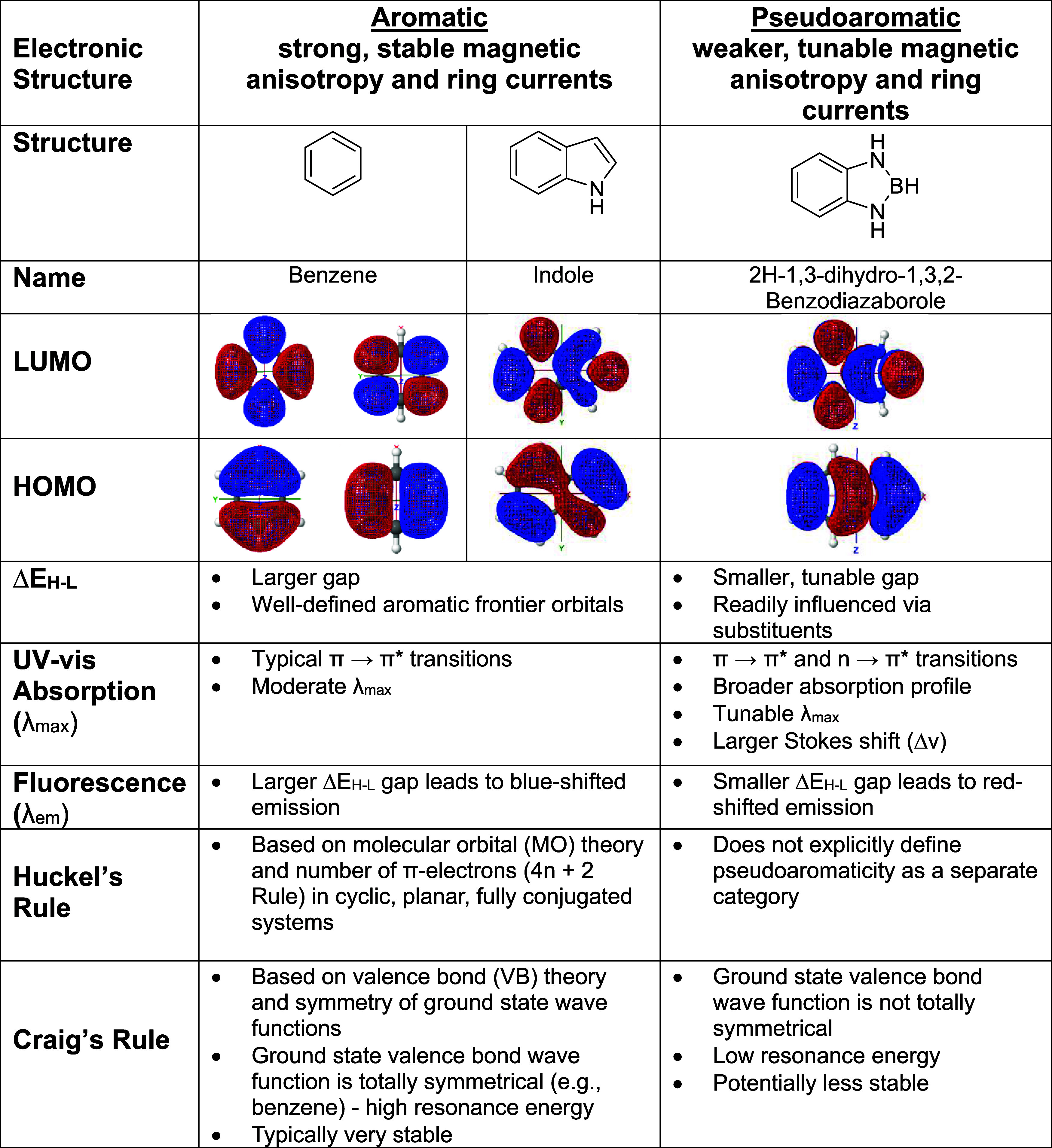
Comparative Electronic and Optical Properties of Benzene, Indole, and 2H-1,3,2-Benzodiazaborole^a^

a This table contrasts key structural, electronic, and photophysical features of benzene (aromatic), indole (heteroaromatic), and 2H-1,3,2-benzodiazaborole (pseudoaromatic). Included are frontier molecular orbital surfaces, UV–vis absorption behavior (λ_max_), fluorescence emission behavior (λ_em_), and theoretical classifications of aromaticity based on Hückel’s and Craig’s rules. As a pseudoaromatic indole isostere, 2H-1,3,2-benzodiazaborole exhibits reduced and tunable HOMO–LUMO gaps (Δ*E*_H–L_), broader absorption profiles, red-shifted emission, and greater environmental sensitivity.

**Figure 1:**
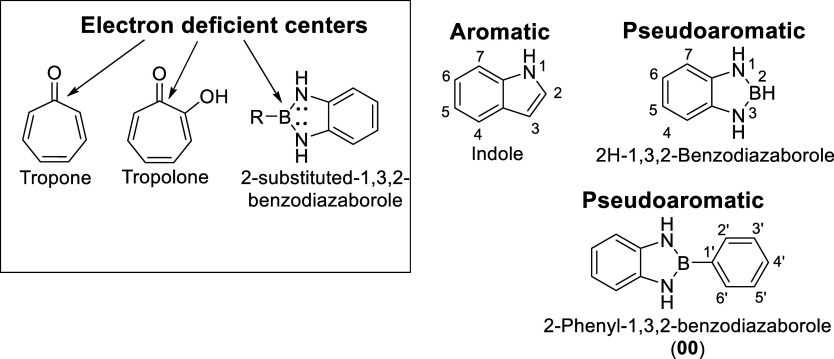
Representative structures of pseudoaromatic systems. In the upper left inset highlights tropone, tropolone, and 2-substituted-1,3,2-benzodiazaborole as pseudoaromatic according to the definition proposed by David P. Craig, which describes systems with partial delocalization and nonsymmetric ground state wave functions. In these compounds, an electron-deficient center (carbonyl carbon or boron) possesses a p_*z*_ orbital that participates in conjugation without contributing signifcant electron density, thereby reducing resonance energy and deviating from classical aromatic behavior. Indole is shown as a benchmark classical aromatic heterocycle, with 10 π-electrons delocalized over a fused bicyclic system. Although 2H-1,3,2-benzodiazaborole is an isostere of indole due to the same number of valence electrons, it is pseudoaromatic: partial back-donation from nitrogen lone pairs into the vacant boron p_*z*_ orbital reduces delocalization, resulting in lower resonance energy and attenuated magnetic anisotropy. 2-Phenyl-1,3,2-benzodiazaborole (**00**) is an air-stable analogue of 2H-1,3,2-benzodiazaborole, in which a phenyl substituent on boron enhances stability while preserving the pseudoaromatic N–B–N framework.

### 1.2 Benzodiazaboroles

Since their discovery, also in the late 1950s, 2-phenyl-1,3,2-benzodiazaboroles have attracted sustained interest as boron-containing heterocycles with unusual electronic properties. Seminal work by Dewar et al.^6^ and Letsinger and Hamilton^7,8^ independently reported the synthesis of 2-phenyl-1,3,2-benzodiazaboroles derivatives using arylboronic reagents and *o*-phenylenediamines. A foundational study by Nyilas and Soloway^9^ established the condensation of aromatic boronic acids with *o*-phenylenediamine as the principal synthetic route (eq 1; Scheme 1). Their 1959 spectroscopic analysis first hinted at pseudoaromatic character, revealing diagnostic IR bands and substituent-sensitive UV absorbance consistent with π-delocalization across the N–B–N triad.

**Scheme 1:**
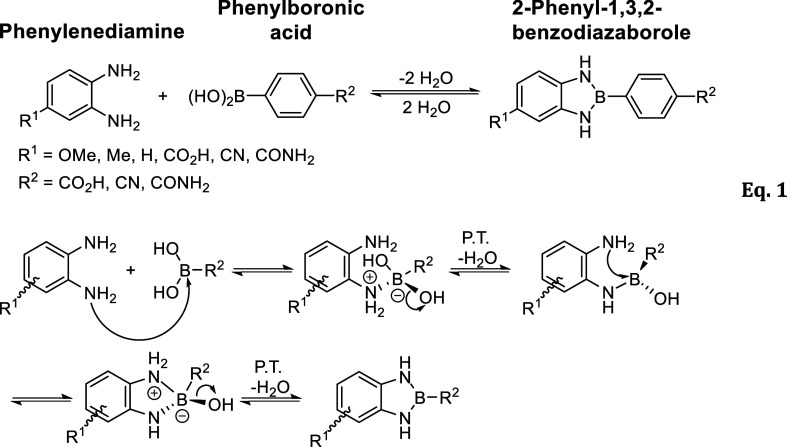
Proposed Mechanism for the Microwave-Assisted Cyclic Condensation of *o*-Phenylenediamines with Substituted Phenylboronic Acids to Yield 2-Phenyl-1,3,2-benzodiazaborole Derivatives

a Initial coordination of one amine to the boronic acid forms a B–N Lewis adduct, followed by dehydration to generate a neutral B–N bond. Subsequent intramolecular attack by the second amine and a second dehydration step results in cyclization to the pseudoaromatic 1,3,2-benzodiazaborole ring.

Throughout the 20th century, advances in X-ray crystallography and NMR supported the existence of partial ring current effects, while the 21st century brought a paradigm shift. Weber’s group demonstrated that 2-phenyl-1,3,2-benzodiazaboroles systems function as π-electron donors—not acceptors—overturning assumptions about tricoordinate boron and revealing intramolecular charge-transfer (ICT) characteristics.^10–12^ This was reinforced by quantitative NICS analyses from the Molander group, confirming pseudoaromaticity as distinct from classical aromaticity.^13^ More recently, studies on electronic behavior have revealed that nitrogen substituents can reversibly tune 2-phenyl-1,3,2-benzodiazaboroles units from donor to acceptor motifs, underscoring their versatility in molecular design.^14^

The N–B–N bonding motif found in 1,3,2-benzodiazaboroles represents a particularly intriguing class of pseudoaromatic systems, where the central boron atom participates in π-conjugation with adjacent nitrogen atoms to form 10 π-electron heterocyclic frameworks.^10^ Unlike typical B–N bonds, the diazaborolyl moiety of these systems exhibit unique electronic properties, functioning as π-electron donors and contributing significantly to the Highest Occupied Molecular Orbital (HOMO) of conjugated molecules, contrary to the more common π-acceptor role associated with B–N bonds.^10,15^

The 2-phenyl-1,3,2-benzodiazaborole scaffold offers exceptional versatility for exploring pseudoaromaticity through systematic substitution, as the planar N–B–N core maintains aromatic character while allowing fine-tuning of electronic properties through peripheral functionalization.^16^ The 10 π-electron aromatic core can be chemically modulated into π-acceptors through strategic substitution of electron-withdrawing groups (EWGs), enabling highly emissive push–pull architectures with tunable ICT character.^15^ Recent investigations of boranol-containing heterocycles suggest that the enhanced π-delocalization capabilities of nitrogen over oxygen make N–B–N motifs more effective for stabilizing pseudoaromatic character, particularly when nitrogen lone pairs are available for conjugation (Figure 2).^17^ N–B–N versus O–B–O systems differ in both bond strength and thermodynamics; N–B and O–B bonds measure ∼1.44 Å and ∼1.39 Å, respectively.^18^ The N–B–N motif supports pseudoaromaticity via strong π-donation from nitrogen lone pairs, enabling extended delocalization and tunable electronic behavior.^19^ Additionally, B–N bonds are weaker and more polarizable (bond order ∼0.95) than B–O (∼1.24), facilitating dynamic charge-transfer (CT) modulation.^18,19^

**Figure 2:**
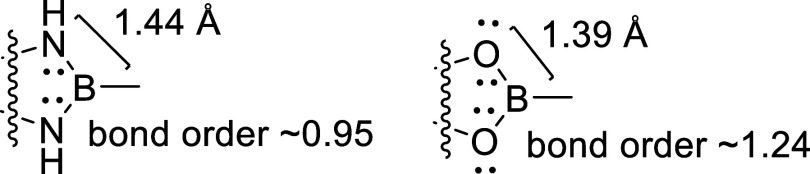
Comparative structure and thermodynamic properties of N–B–N and O–B–O bonding motifs. N–B and O–B bonds measure ∼1.44 Å and ∼1.39 Å, respectively. While boronate ester (O–B–O) formation is more thermodynamically favorable (Δ*H* = 6.1–6.9 kcal/mol) than diazaborole (N–B–N) formation (Δ*H* = 10.0–10.3 kcal/mol), the latter offers unique electronic advantages. The N–B–N motif supports pseudoaromaticity via strong π-donation from nitrogen lone pairs, enabling extended delocalization and tunable electronic behavior. B–N bonds are weaker and more polarizable (bond order ∼0.95) than B–O (∼1.24), facilitating dynamic charge-transfer modulation.

### 1.3 Charge Transfer Complexes

Charge transfer complexes (CTCs) represent a class of supramolecular assemblies formed through electronic CT between electron donor and acceptor entities, where a fraction of electronic charge is transferred between molecular components, creating electrostatic attraction that provides a stabilizing force for the molecular complex (Figure 3).^20^ These donor–acceptor architectures have emerged as versatile platforms for developing novel optical and electronic materials, particularly when ICT occurs upon photoexcitation, creating push–pull systems where donor groups function as “push” units and acceptor moieties serve as “pull” units.^21^ Within this framework, N–B–N containing systems, like 2-phenyl-1,3,2-benzodiazaborole derivatives, have garnered significant attention due to their unique electronic properties where the diazaborolyl moiety predominantly contributes to the HOMO while functioning as an effective π-donor substituent rather than a traditional π-acceptor (Figure 3).^22^

**Figure 3:**
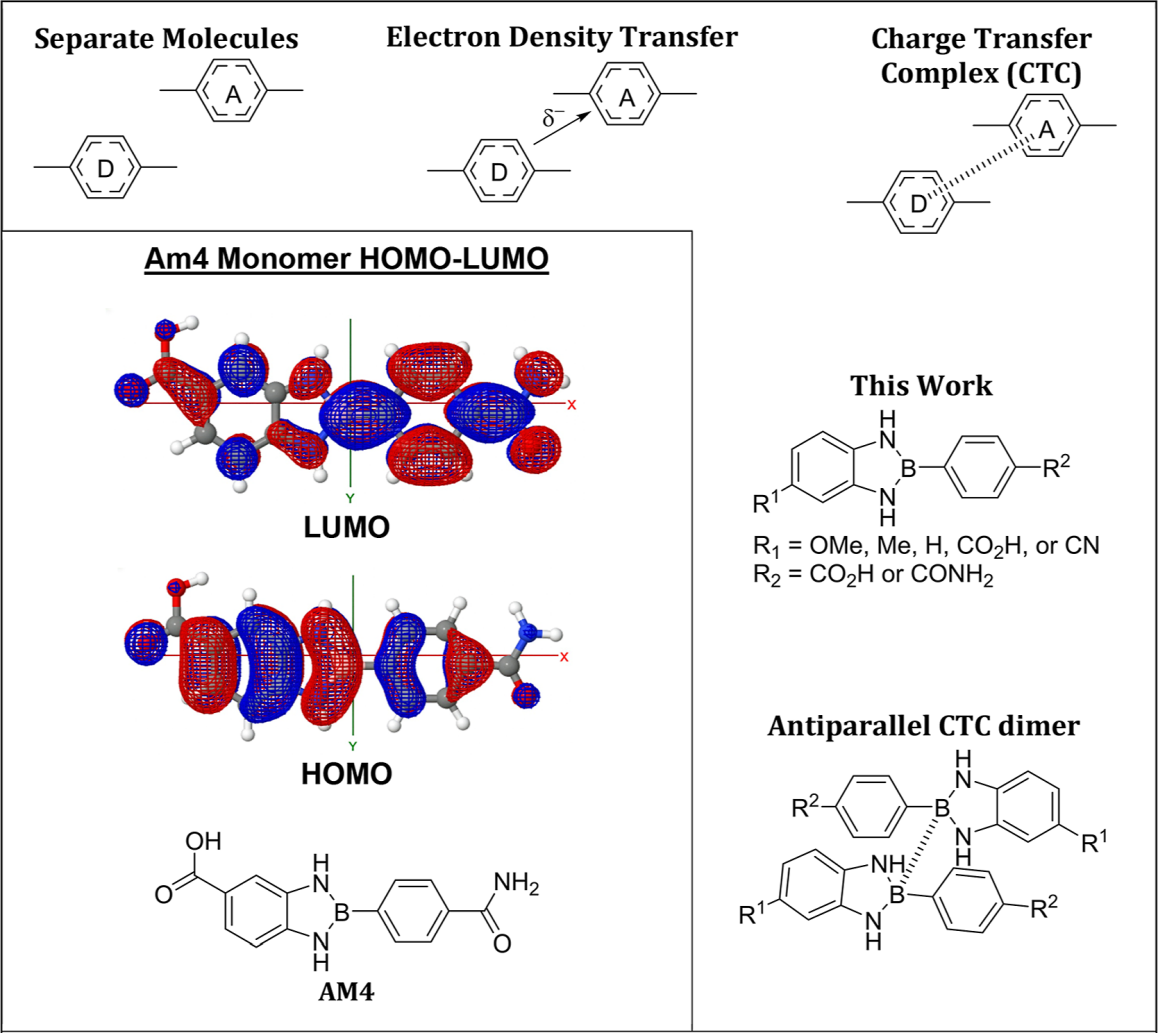
Proposed general charge-transfer complex (CTC) formation in amide- and carboxy-substituted 2-phenyl-1,3,2-benzodiazaborole derivatives. Generic CTCs form through partial electron donation from a donor (D) to an acceptor (A), often via π–π stacking or coordination. Our calculations predict an antiparallel dimeric CTC with overlap of the boron atoms p_*z*_ orbitals, forming pseudoaromatic dimers with red-shifted fluorescence and large stokes shifts (Δν). HOMO–LUMO diagrams show energy level shifts from monomer to dimer.

The 10 π-electron benzodiazaborole core can be strategically transformed into π-acceptor units through incorporation of strong EWGs, enabling the construction of highly luminescent push–pull molecules with tunable CT characteristics.^22^ Remarkably, recent advances have demonstrated that benzodiazaborole-based donor–acceptor conjugated polymers exhibit distinct electrochromic behavior with continuous spectral changes attributed to charge carrier generation, highlighting their potential in optoelectronic applications.^23^ The intersection of CT phenomena with pseudoaromaticity has opened new avenues through the development of partially aromatized ICT systems, where ICT occurs within pre-excited donor–acceptor structures containing pseudoaromatic or unstable aromatic rings as acceptor moieties, leading to common partially aromatized CT states that exhibit unique photophysical properties distinct from conventional aromatic systems.^24^

To date, there are little reports of carboxy-substituted 2-phenyl-1,3,2-benzodiazaborole and no reports of amide-substituted derivatives. The potential of these functional groups to form and tune a CTC and the role of the N–B–N motif therefore remains unexplored. In these cases, pseudoaromaticity can facilitate partial charge delocalization leading to new optical transitions, large Stokes shifts, and red-shifted emissions, features of growing interest in molecular sensing, photophysics, and optoelectronic materials.

The development of amide- and carboxy-substituted 2-phenyl-1,3,2-benzodiazaborole derivatives introduces a new frontier in designing pseudoaromatic frameworks that promote ICT and allow for highly tunable excited-state behavior. In these 10 π-electron systems, the benzodiazaborole core plays a crucial role in the HOMO distribution, while EWGs such as amide or carboxy substituents localize the Lowest Unoccupied Molecular Orbital (LUMO) on the 2-phenyl ring, forming donor–π–acceptor architectures.^11^ The inclusion of polar functional groups facilitates photoinduced charge separation, creating emissive excited states that significantly differ from π → π* transitions in symmetrical boron heterocycles. Prior studies have largely focused on metal coordination or ICT in luminescent systems rather than intermolecular CTC formation.^25^ Studies of functionalized benzodiazaboroles like C-diazaborolyl-carboranes by Weber and co-workers have shown that these systems are predicted to yield exceptionally large Stokes shifts, dual emission, and remote CT, indicative of strong ICT behavior.^10,14,26,27^ In this context, amide- and carboxy-substituted 2-phenyl-1,3,2-benzodiazaboroles represent a novel class of asymmetrical donor systems with enhanced charge-separation capabilities, offering unique platforms for constructing organic CTCs with potential applications in optoelectronics.

### 1.4 This Work

In this work, we report the synthesis of novel amide- and carboxy-functionalized 2-phenyl-1,3,2-benzodiazaborole derivatives via microwave-irradiation (MWI) assisted cyclic condensation (Scheme 1). We use a combination of spectroscopic techniques and Density Functional Theory (DFT) calculations to evaluate structure and behavior. These compounds were designed to investigate how substitution patterns affect synthesis and photophysical behavior and to probe the relationship between pseudoaromaticity and CT. By tuning the substituents and analyzing emission behavior across solvent systems, we aim to uncover how structural features drive fluorescence and dimerization, positioning 2-phenyl-1,3,2-benzodiazaboroles frameworks as promising scaffolds for functional material development.

## 2 Results and Discussion

### 2.1 Synthetic Optimization

A series of 2-(4′-carbamoylphenyl)- and 2-(4′-carboxyphenyl)-1,3,2-benzodiazaboroles was synthesized using MWI-assisted cyclic condensation of substituted *o*-phenylenediamines with *p*-amide- or *p-*carboxy-substituted phenylboronic acids (Scheme 1). Initial attempts to synthesize carboxy- and amide-substituted derivatives using toluene (PhMe) or a 1:1 mixture of PhMe and ethyl acetate (EtOAc) resulted in minimal conversion. The addition of NEt_3_ (10–20 mol %) modestly improved yields, but significant enhancement was observed upon incorporating 2–4% DMSO (v/v) into diglyme or a 1:1 solution of PhMe/EtOAc, likely due to increased solubility of polar starting materials. Post reaction purification by stirring the crude in hot EtOAc or EtOAc: acetone (1:1) for 30 min followed by hot vacuum filtration afforded pure products in moderate yields (25–83%). Melting points ranged from 271 to over 300 °C, substantially higher than the parent unsubstituted 2-phenyl-1,3,2-benzodiazaborole compound, 00, (212–214 °C). Many products displayed opalescent or colored appearances, suggesting extended conjugation or aggregation (Table 2).

**Table 2:**
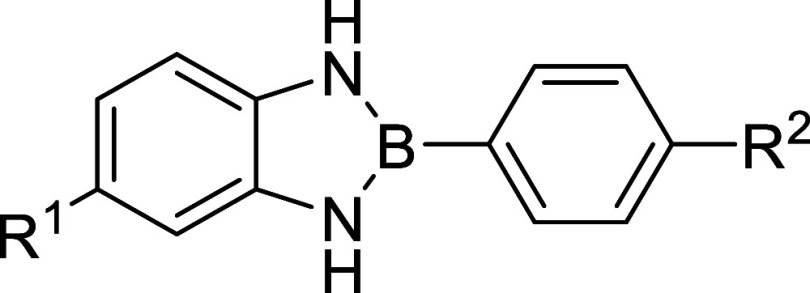
Summary of synthesized amide- and carboxy-substituted 2-phenyl-1,3,2-benzodiazaborole derivatives^a^

compound (*R*_1_ – *R*_2_)	% yield	melting Point (°C)	description	^1^H NMR HN–B–NH (δ = ppm)	Calc. Δ*E*_H_–_L_ (eV	extinction coefficient in acetonitrile (ε = M^–1^cm^–1^)
00 (H–H)	33	212 – 214	sandy white powder	9.12	3.72	20,310
**CA1 (OCH**_**3**_–CO_2_H)	62	>300	opalescent, metallic copper powder	9.21	2.88	-----
				9.09		
**Am1 (OCH**_**3**_–CONH_2_)	30	271 – 275	brown powder	9.16	3.02	3,325
				9.03		
**CA2 (CH**_**3**_–CO_2_H)	67	295 – 296	white powder	9.16	3.07	-----
**Am2 (CH**_**3**_–CONH_2_)	60	301 – 303	opalescent peach powder	9.10	3.21	21,643
CA3 (H – CO_2_H)	83	291 – 293	opalescent white powder	9.28	3.14	-----
Am3 (H–CONH_2_)	64	290 – 291	opalescent white powder	9.24	3.26	17,188
CA4 (CO_2_H – CO_2_H)	79	>300	white powder	9.71	3.04	1,006
				9.54		
CN4 (CO_2_H–CN)	2	>300	white powder	9.78	3.28	19,260
				9.61		
Am4 (CO_2_H–CONH_2_)	25	296 – 298	white, opalescent powder	9.65	3.41	-----
				9.48		
CA5 (CN–CO_2_H)	32	>300	white powder	9.78	3.29	-----
				9.61		
Am5 (CN–CONH_2_)	50	287 – 289	white powder	9.86	3.45	27,622
				9.63		

a Includes yield (%); melting point (°C); physical description; ^1^H NMR chemical shifts of boronic amines (δ = ppm); DFT-calculated HOMO–LUMO energy gaps, Calc. Δ*E*_H_–_L_, (eV); and extinction coefficients, ε, in acetonitrile (M^–1^cm^–1^).

### 2.2 NMR Characterization (^1^H, ^13^C, ^11^B, HSQC/HMBC)

^1^H NMR spectra were acquired using a 300 MHz Bruker Avance II spectrometer and confirmed the boronic –NH signals between δ = 9.03–9.86 ppm (Supporting Information). Strong electron-donating groups (EDGs), (i.e., –OCH_3_) and strong EWGs (i.e., –CO_2_H and –CN) caused electronic nonequivalence, generating two distinct singlets for the boronic amines, while –CH_3_ and –H substituents yielded merged signals. All amide compounds had two distinct amide NH_2_ signals around δ = 7.4–7.7 ppm with one signal overlapped with the phenyl ring AB pair (Figure 4). D_2_O experiments confirmed the presence of the ionizable amide protons as seen in Figure 4. Strangely, the boronic amines do not decrease in intensity although they do decrease in the unsubstituted parent **00** (data not shown). This suggests a strong boronic N–H bond, inaccessibility due to intermolecular interactions, or a change in the electronic structure due to the ionization of the amide protons. In bis-substituted compounds, the three hydrogen atoms on the benzodiazaborole ring appear as the most upfield signals in the aromatic region (δ = 7.58–6.43 ppm), while the four protons on the *para*-substituted phenyl ring display an AB splitting pattern more downfield (8.10–7.84 ppm), consistent with their symmetric substitution pattern (Supporting Information).

**Figure 4:**
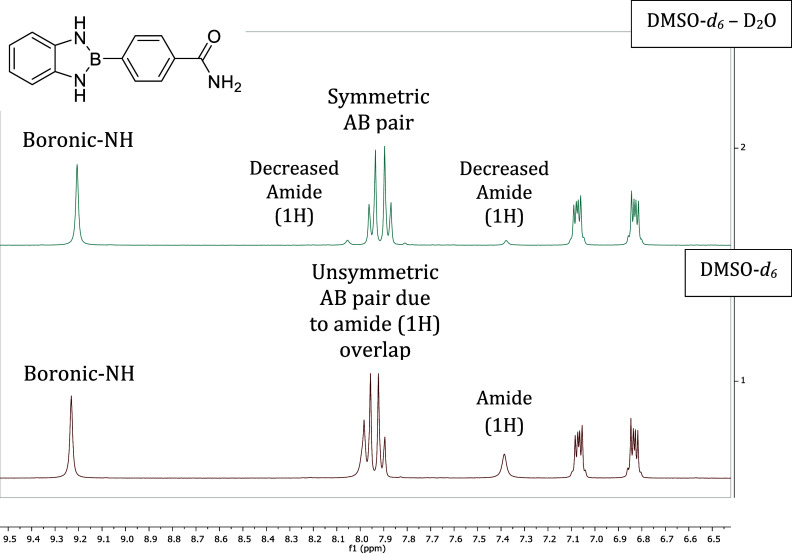
^1^H NMR spectra of the Am3 derivative before and after D_2_O exchange, illustrating the disappearance of labile protons. Prior to exchange, the amide protons appear as two signals: one at 7.39 ppm and a second overlapping with the benzodiazaborole AB pair, resulting in an asymmetric pattern. After D_2_O addition, the AB pattern becomes more symmetric and the 7.39 ppm signal diminishes, confirming exchange of the amide N–H proton. Notably, the boronic N–H signal remains unchanged, suggesting either reduced accessibility due to intermolecular interactions or a stronger N–H bond resistant to exchange.

^13^C NMR spectra consistently showed one fewer carbon than expected due to quadrupolar broadening and signal suppression from the ^11^B nucleus on the *ipso* carbon (Supporting Information). The quadrupolar relaxation of boron causes severe line broadening of the adjacent carbon’s signal, often to the point where it becomes undetectable above the baseline noise.^19,28–31^ HSQC and HMBC experiments were conducted to confirm the ^1^H and ^13^C NMR assignments (Supporting Information). The HSQC spectrum of compounds **CA1** showed a direct one-bond correlation between aromatic protons and their corresponding carbons, including strong cross-peak between δ H = 6.45 ppm and δ_C = 105.63 ppm; δ H = 6.68 ppm, δ_C = 97.98 ppm; and δ H = 6.94 ppm and δ_C = 111.20 ppm, consistent with three –CH signals on the benzodiazaborole ring. As expected, the boronic –NH proton displayed no corresponding carbon signal in HSQC. In the HMBC spectrum, long-range ^1^H – ^13^C correlations further validated the structure. For example, in **Am3**, the boronic –NH protons at δ H = 9.30 ppm showed a three-bond correlation to carbons at δ_C = 111.40 and 137.55 ppm, confirming their assignment as benzodiazaborole carbons. Additionally, the two amide –NH_2_ signals at δ_H = 7.44 ppm and buried under the AB pair 7.97 ppm exhibited two- and three-bond correlations to aromatic carbons at δ_C = 135.34 and 168.33 respectively supporting assignment of δ_C = 135.34 ppm as the *ipso* C and 168.33 ppm as the carbonyl.

^11^B NMR spectra were acquired using a 500 MHz Bruker Avance III HD spectrometer. ^11^B spectra showed the boron atom’s electronic structure is affected by the benzo substituent with the EDG **Am2** more upfield (δ = 29.0 ppm) than the EWG **Am5** (δ = 29.99 ppm, Supporting Information). ^11^B NMR together with HSQC and HMBC spectra provided complementary information that confirmed the full structural assignment, including the location of the boronic –NH, amide, and carboxyl substituents and confirming the integrity of the pseudoaromatic scaffold.

### 2.3 Hydrolytic Stability and Degradation Trends

1,3,2-Benzodiazaboroles have had conflicting reports of air-induced hydrolysis, reverting to the parent *o*-phenylenediamine and boronic acid (eq 1).^9,17,29,32–35^ Most of our derivatives appear to be air stable; however, we have noticed potential hydrolytic degradation in EDG-substituted amide derivatives (**Am1** and **Am2**), as evidenced by the emergence of new signals in the ^1^H NMR spectra over time (Supporting Information Figure 1). In contrast, derivatives bearing EWGs (**Am4** and **Am5**) appear to be more inert under ambient conditions. Efforts to synthesize amide salt analogues to improve hydrolytic stability—either by starting from the salt or by postsynthetic modification—were unsuccessful, although investigations are ongoing. Anecdotally, we have heated bis-carboxy (CA4) in water at 70 °C for several hours without signs of product hydrolysis as measured by ^1^H NMR (data not shown). These results suggest that substitution patterns significantly influence hydrolytic stability and may guide future development of benzodiazaborole-based systems for aqueous or functionalized applications.

### 2.4 IR Spectroscopy and N–B–N Motif Assignment

Infrared spectroscopy (IR) spectra were obtained using a Thermo Scientific Nicolet iS50-FT-IR spectrometer. The IR spectra of the amide- and carboxy-substituted 2-phenyl-1,3,2-benzodiazaborole derivatives provide key vibrational signatures that support successful formation of the desired heterocycles and highlight their structural features (Supporting Information). Characteristic broad bands in the 3327–3442 cm^–1^ region are attributed to N—H stretching from the 2-phenyl-1,3,2-benzodiazaborole core, while additional signals between 3158–3170 cm^–1^ confirm the presence of terminal amide –NH_2_ groups. Amide- and carboxy-carbonyl C=O stretching bands appear within 1611–1673 cm^–1^. Peculiarly, we noticed several derivatives (**Am1**, **Am2**, and **Am5**) show reduced intensities of the carbonyl stretching bands. The presence of weakened and partially resolved symmetric carbonyl modes suggests that the carbonyl groups experience more than one local environment, which may be consistent with dimeric association. Diagnostic B–N stretching absorptions between 1317–1407 cm^–1^ are also observed, supporting formation of the pseudoaromatic N–B–N motif. B—N frequencies for **CA1** and **CA3** display similar symmetric features, indicating analogous electronic or structural environments. Bands in the 1154–1287 cm^–1^ range are assigned to B—C_aryl_ stretching modes, further corroborating incorporation of the boron center into the conjugated system.

### 2.5 UV–Vis Absorption

UV–vis spectra were recorded on a Thermo Fisher Scientific Evolution 201 UV–visible Spectrophotometer using quartz cuvettes. UV–vis absorption maxima (λ_max_ = 298 – 319 nm in ethanol, EtOH; acetonitrile, MeCN; or DMSO) showed minor variation (Figure 5). In contrast, the corresponding molar extinction coefficients vary substantially, ε = 1006 – 27622 M^–1^ cm^–1^ indicating pronounced differences in transition intensity (Table 2). Multiple absorbance peaks suggest π → π* and possible *n* → π* transitions or CTCs; however, the intensity and breadth of these bands indicate that allowed π → π* transitions dominate the absorption spectra, while any *n* → π* contributions from nitrogen lone pairs are weak.

**Figure 5:**
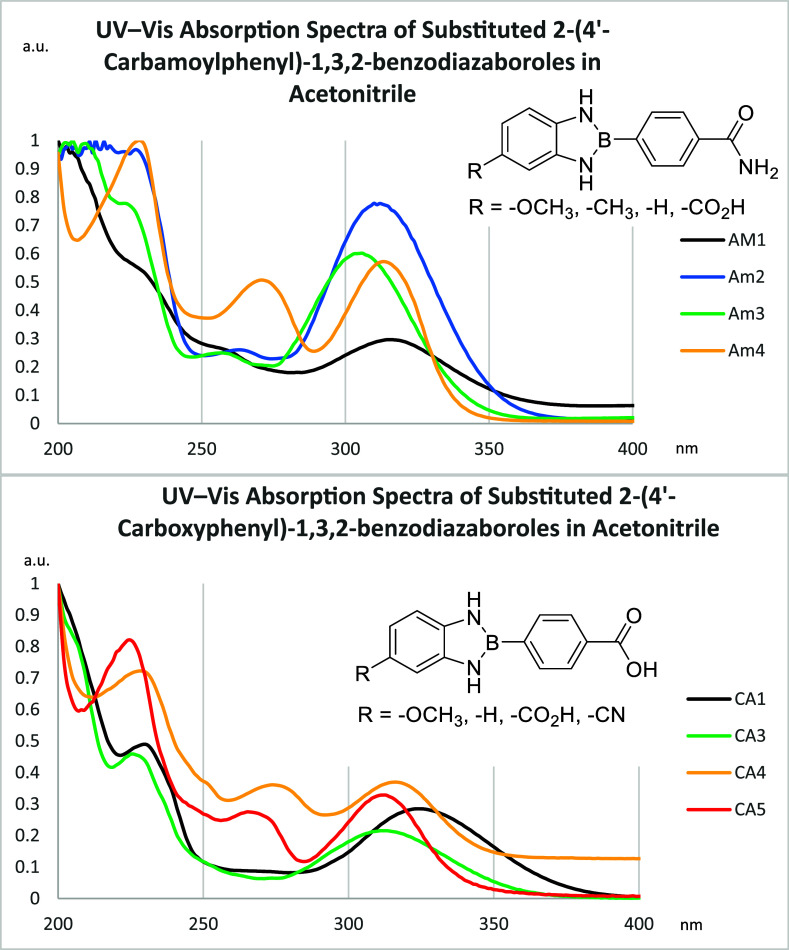
Overlayed UV–vis absorption spectra of amide- (**Am1**–**Am4**) and carboxy-substituted (**CA1**, **CA3**–**CA5**) 2-phenyl-1,3,2-benzodiazaboroles in MeCN at 1.0 × 10^–4^ M concentration and room temperature. All spectra are normalized to highlight relative spectral features. Absorption maxima (λ_max_) are clustered within a narrow range of 305–325 nm, consistent with π → π* transitions influenced by the electronic nature of the substituents.

### 2.6 Fluorescence Emission and Excitation

Fluorescence spectra were recorded on an Agilent Technologies Cary Eclipse Fluorescence Spectrophotometer using quartz cuvettes. Fluorescence emission ranged from λ_em_ = 363 – 555 nm in EtOH, MeCN, or DMSO, using λ_ex_ = 300 nm for amide derivatives and 315 nm for carboxy derivatives (Supporting Information). Under identical solvent conditions, the parent compound **00** exhibits significantly blue-shifted emission (λ_em_ = 366 – 383 nm). Comparatively, many substituted analogues display pronounced substituent- and solvent-dependent bathochromic shifts with EDGs producing the most red-shifted fluorescence (Figure 6). Notably, **Am3** (λ_em_ = 555 nm, DMSO) and **CA1** (λ_em_ = 525 nm, MeCN; Figure 6) exhibit the largest red shifts. Corresponding Stokes shifts (Δν) ranged from 65–243 nm with **Am4**, **Am2**, and **CA1** exhibiting the largest values (Δν = 243 nm, DMSO; 205 nm, EtOH; and 201 nm, MeCN respectively; Supporting Information Table 1). Collectively, these results suggest ICT pathways in which EDGs and polar solvents stabilize the emissive excited state.

**Figure 6:**
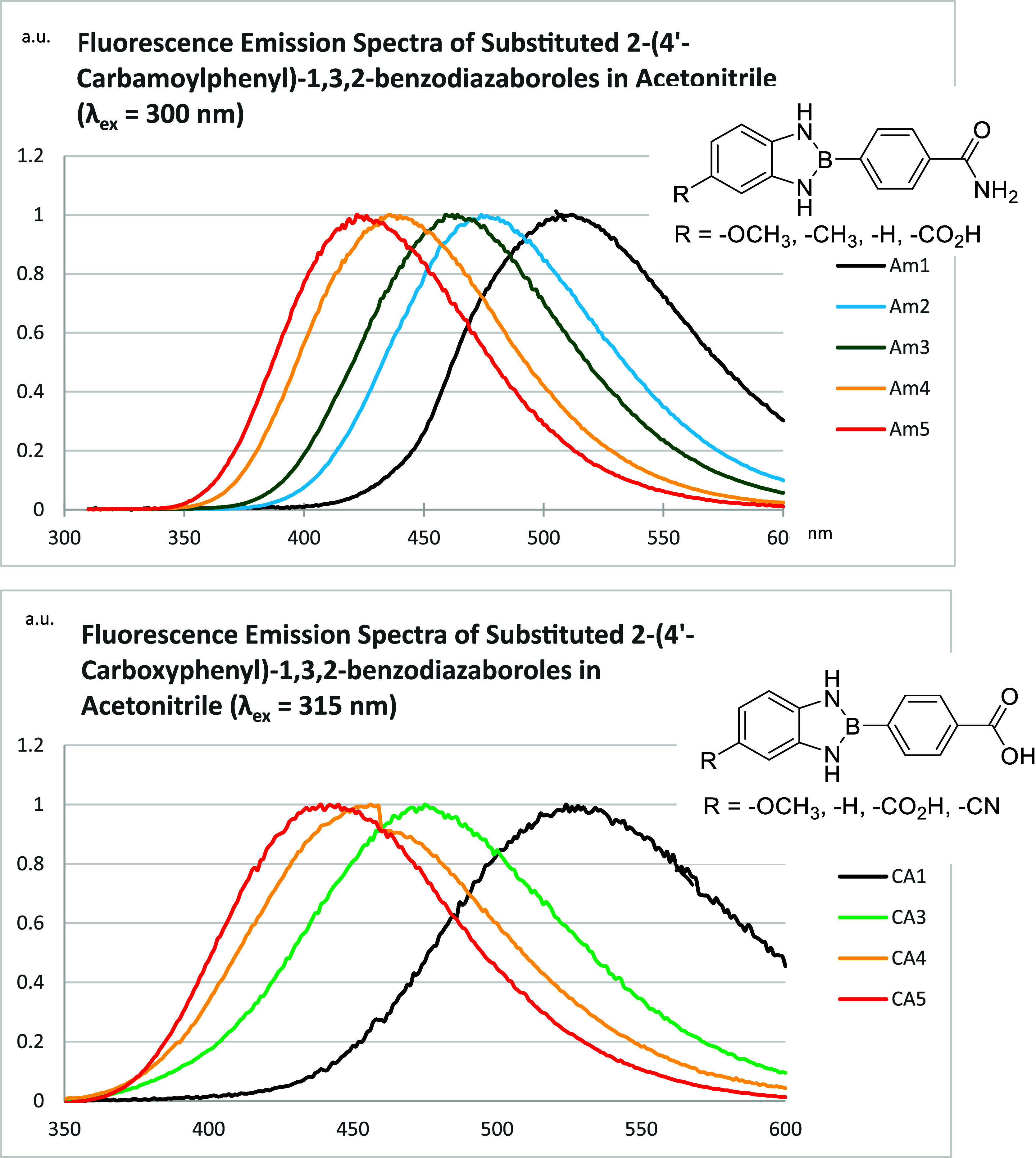
Normalized fluorescence emission spectra of amide- (**Am1**–**Am5**) and carboxy-substituted (**CA1**, **CA3**–**CA5**) 2-phenyl-1,3,2-benzodiazaboroles in MeCN (1.0 × 10^–4^ M) at 25 °C. EDGs (e.g., –OCH_3_, –CH_3_) induce red-shifted (bathochromic) emission, while electron-withdrawing substituents (e.g., –CO_2_H, –CN) lead to broadened, lower-energy emission profiles. Both trends are consistent with enhanced charge-transfer character.

Fluorescence excitation spectra were compared to corresponding UV–vis absorption profiles, generally coinciding with or slightly red-shifted from the absorbance maxima (λ_max_), indicating that the emissive states are accessed through efficient *S*_0_ → *S*_1_ singlet–singlet electronic transitions, where *S*_0_ and *S*_1_ denote the ground and first excited singlet states, respectively (Supporting Information). Across both amide- and carboxy-substituted series, the excitation spectra preferentially track the lower-energy absorption features rather than higher-energy bands, consistent with selective population of relaxed π → π* excited states. The alignment between spectral bathochromic shifts and orbital energies suggests that pseudoaromatic delocalization and N → B donor–acceptor interactions facilitate in stabilizing low-energy transitions. Stabilization of low-energy transitions reflects a reduction in the HOMO–LUMO gap arising from pseudoaromatic delocalization and donor–acceptor interactions. The absence of distinct excitation features uniquely attributable to *n* → π* transitions further support a photophysical model dominated by π-delocalized and CT like excitations. Overall, the combined UV–vis, excitation, and emission behavior supports a model in which electronic transitions are finely modulated by substituent-controlled conjugation, polarizability, and intermolecular interactions, consistent with tunable ICT character rather than isolated local excitations.

### 2.7 DFT Modeling: HOMO–LUMO, Dimer Formation, and Charge–Transfer Complex Evidence

Density Functional Theory (DFT) calculations were performed using the B3LYP/6-31G functional and 6-31G via the GAMESS platform to estimate frontier orbital energies. Geometry optimizations were carried out at the Restricted Hartree–Fock method level, and the HOMO–LUMO energy gaps (Δ*E*_H–L_) were extracted from the optimized structures (Figure 7; Supporting Information). The computed Δ*E*_H–L_ values for the compound library ranged from 2.88 to 3.45 eV, demonstrating significant electronic tunability (Table 2). The unsubstituted parent compound, **00**, exhibited a HOMO–LUMO gap of 3.72 eV, while derivatives bearing strong EDGs displayed smaller gaps, consistent with enhanced conjugation and charge delocalization across the B–N scaffold. Dimeric models of selected compounds were also evaluated to probe potential CT interactions. Notably, the antiparallel dimer of **Am4** showed a reduced gap of 3.10 eV relative to the monomeric form (3.41 eV), supporting intermolecular electronic communication via π-stacking or donor–acceptor alignment (Figure 7).

**Figure 7:**
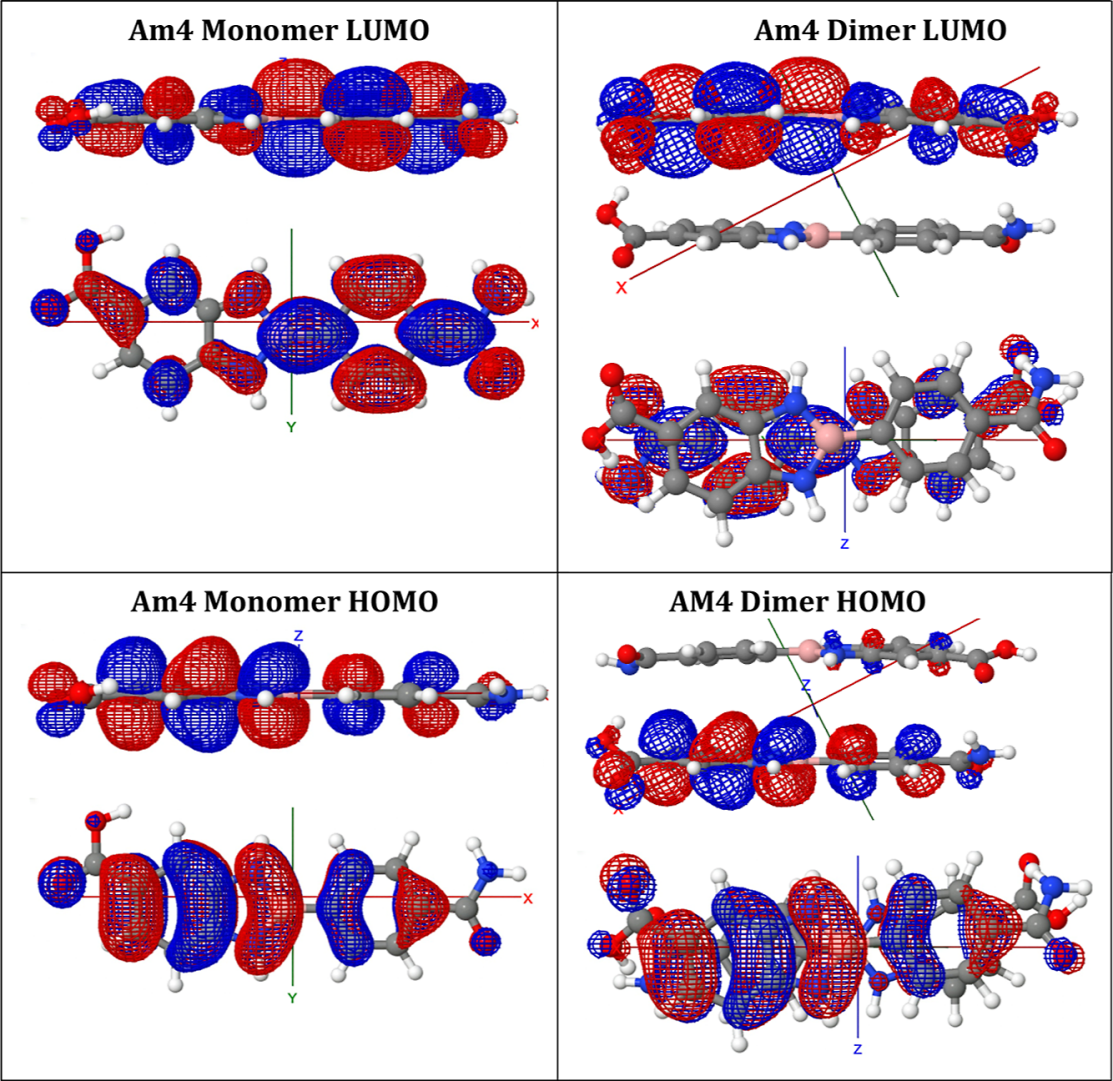
DFT-calculated frontier molecular orbitals (HOMO and LUMO) for **Am4** in monomeric and antiparallel dimeric configurations. Visualized orbital distributions and energy gaps illustrate substituent effects, intermolecular interactions, and potential charge-transfer character in antiparallel dimers. Among the dimer geometries examined, the antiparallel configuration was calculated to be the lowest in energy; higher-energy arrangements (e.g., parallel) are not shown.

The observed variation in Δ*E*_H–L_ values across the compound series reflects the impact of substituent electronics on the pseudoaromatic benzodiazaborole core. Lower gaps in –OCH_3_ and –CH_3_ derivatives support stronger donor character and delocalization, aligning with their red-shifted fluorescence and large Stokes shifts. The good agreement between theoretical HOMO–LUMO gaps and experimental photophysical trends (i.e., λ_max_, λ_em_, λ_ex_, Δν) underscores the validity of using computational tools to predict structure–property relationships in pseudoaromatic heterocycles. Furthermore, the dimeric CTC models exhibit frontier orbital localization across monomer units, supporting the proposed mechanism of through-space or through-bond CT observed in spectroscopy. These models support evidence that amide- and carboxy-substituted 2-phenyl-1,3,2-benzodiazaborole with strong EDGs act as intramolecular donor–acceptor units that can form dimers. These findings highlight the importance of both molecular substitution and intermolecular orientation in controlling the electronic structure and photophysical properties of 2-phenyl-1,3,2-benzodiazaborole derivatives.

Collectively, these results suggest that amide- and carboxy- substitution enhances pseudoaromatic delocalization, promoting CT interactions and red-shifted photophysical behavior. Spectroscopic trends, DFT data, and structural analysis support the formation of intermolecular dimers stabilized via B–N coordination and π-stacking, confirming the unique electronic environments induced by substitution, and underscoring the potential of these materials in fluorescence-based sensing and optoelectronics.

### 2.8 Methods

All experiments except ^11^B NMR and high-resolution mass spectroscopy (HRMS) were conducted at State University of New York (SUNY) Cortland; ^11^B NMR and HRMS were acquired at Cornell University. Starting materials and reagents were obtained from Sigma-Aldrich, Combi-Blocks, TCI, or AA Blocks and used without further purification. MWI reactions were carried out in a CEM Discover 2.0 microwave reactor using 10 mL Pyrex pressure vessels fitted with TFM septa vial caps. Melting point ranges were measured using a Stuart SMP 10 melting point apparatus and are reported uncorrected. UV–vis spectra were recorded on a Thermo Fisher Scientific Evolution 201 UV–visible Spectrophotometer using quartz cuvettes. Fluorescence spectra were recorded on an Agilent Technologies Cary Eclipse fluorescence spectrophotometer using quartz cuvettes. IR spectra were obtained using a Thermo Scientific Nicolet iS50-FT-IR spectrometer. ^1^H and ^13^C NMR spectra were recorded on a 300 MHz Varian Avance II spectrometer; ^11^B spectra were recorded on a 500 mHz Varian Avance III HD spectrometer. Samples were dissolved in DMSO-*d*_6_, and chemical shifts are reported in ppm relative to tetramethylsilane (TMS) or DMSO-*d*_6_ as an internal standard; ^11^B NMR chemical shifts are reported without a standard. HRMS spectra were acquired on a DART-SVP (Direct Analysis in Real Time) ion source (IonSense, Saugus, MA) coupled to an Exactive Orbitrap mass spectrometer (Thermo Scientific, Bremen, Germany).

Computations were performed using the GAMESS software suite via the Chem Compute platform.^36,37^ Initial wave function estimations were obtained using the Restricted Hartree–Fock (RHF) method. Geometry optimizations were conducted at the density functional theory (DFT) level, employing the B3LYP functional with the 6-31G basis set. Graphics were rendered in JSmol.^38^

The parent 2-phenyl-1,3,2-benzodiazaborole (**00)** was synthesized according to literature methods.^29^

#### 2.8.1 Synthesis of Amide- or Carboxy-Substituted 2-Phenyl-1,3,2-benzodiazaboroles

Amide derivatives were synthesized by combining 3,4-diaminobenzamide or *p*-aminophenylboronic acid (1.0 eq ) with the corresponding substituted phenylboronic acid or phenylenediamine (1.2–1.4 eq ) and heating at 115 °C for 10 min under microwave irradiation. Reactions were carried out under one of two solvent conditions:

Condition A: DMSO in diglyme 2–4% v/v (3–4 mL) and triethylamine (20 mol %) were used for EWG-substituted phenylenediamine with all *p*-amido- or *p*-carboxyphenylboronic acids.

Condition B: PhMe/EtOAc (1:1) with triethylamine (20 mol %) was used for EDG-substituted phenylenediamine reactions.

The resulting crude solids were stirred in hot ethyl acetate (70 °C) for 30–60 min, vacuum filtered while hot, and dried for several hours in a vacuum oven at 75 °C.
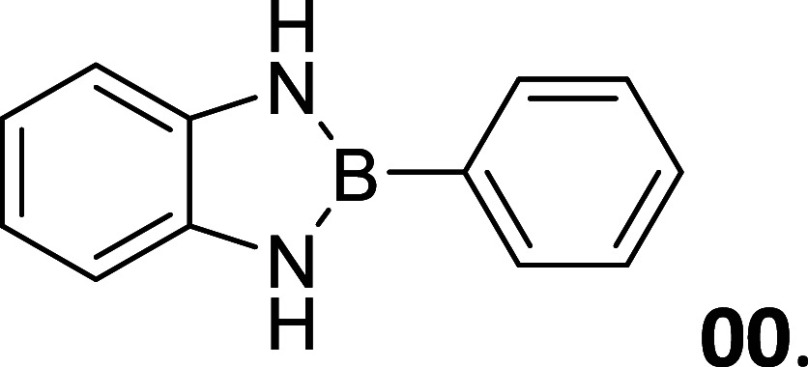


#### 2.8.2 2-Phenylbenzo­[*d*]­1,3,2-diazaborole

33% Yield, mp = 212–214 °C, sandy white powder; IR spectrum (KBr), ν, cm^–1^: 3441, 3418, 1359, 1420, 1173, 1269. ^1^H 301 MHz, DMSO-*d*_6_): δ 12.73 (s, 1H), 9.21 (s, 1H), 9.09 (s, 1H), 7.97 (s, 4H), 6.94 (d, *J* = 8.4 Hz, 1H), 6.68 (d, *J* = 2.4 Hz, 1H), 6.45 (dd, *J* = 8.4, 2.5 Hz, 1H), 3.71 (s, 3H). ^13^C NMR (76 MHz, DMSO-*d*_6_): δ 167.53, 153.31, 137.84, 133.30, 131.30, 128.64, 113.73, 110.74, 104.67, 97.50, 55.42. λ_MAX_ (EtOH) = 294 nm, λ_MAX_ (MeCN) = 296 nm, λ_MAX_ (DMSO) = 300 nm; λ_Em_ (EtOH) = 366 nm, λ_Em_ (MeCN) = 380 nm, λ_Em_ (DMSO) = 383 nm.
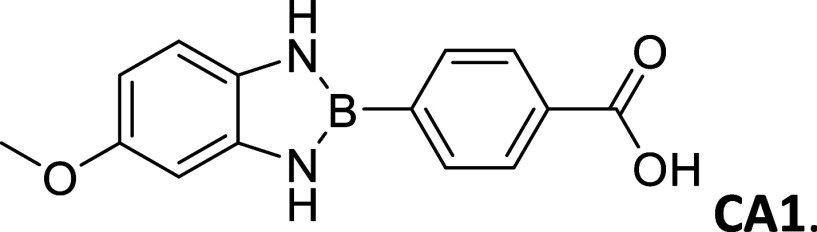


#### 2.8.3 2-(4′-Carboxyphenyl)-5-Methoxybenzo­[*d*]­1,3,2-diazaborole

Procedure B: 62% Yield, mp = >300 °C, Opalescent metallic copper powder; IR spectrum (KBr), ν, cm^–1^: 3447, 3424, 1370, 1398, 1418, 1153, 1290. ^1^H 301 MHz, DMSO-*d*_6_): δ 12.73 (s, 1H), 9.21 (s, 1H), 9.09 (s, 1H), 7.97 (s, 4H), 6.94 (d, *J* = 8.4 Hz, 1H), 6.68 (d, *J* = 2.4 Hz, 1H), 6.45 (dd, *J* = 8.4, 2.5 Hz, 1H), 3.71 (s, 3H). ^13^C NMR (76 MHz, DMSO-*d*_6_): δ 167.53, 153.31, 137.84, 133.30, 131.30, 128.64, 113.73, 110.74, 104.67, 97.50, 55.42. λ_MAX_ (MeCN) = 324 nm; λ_Em_ (MeCN) = 525 nm. HRMS (DART/Orbitrap) *m*/*z*: [M + H]^+^ calcd for C_14_H_13_BN_2_O_3,_ 268.1019; found, 269.10920.
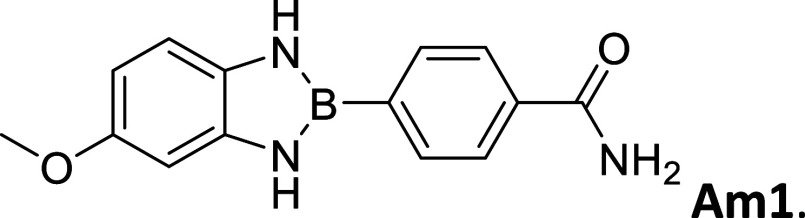


#### 2.8.4 2-(4′-Aminocarbonylphenyl)-5-methoxybenzo­[*d*]­1,3,2-diazaborole

Procedure B: 30% yield. mp = 271–275 °C. Brown powder, IR spectrum (KBr), ν, cm^–1^: 3442, 3383, 1355, 1407, 1154, 1258. ^1^H NMR (301 MHz, DMSO-*d*_6_): δ 9.16 (s, 1H), 9.03 (s, 1H), 8.06–7.85 (m, 5H), 7.39 (s, 1H), 6.93 (d, *J* = 8.4 Hz, 1H), 6.67 (d, *J* = 2.5 Hz, 1H), 6.44 (dd, *J* = 8.5, 2.5 Hz, 1H), 3.71 (s, 3H). ^13^C NMR (76 MHz, DMSO-*d*_6_): δ 167.96, 153.24, 137.86, 134.64, 133.05, 131.32, 126.87, 110.61, 104.51, 97.46, 55.41. λ_MAX_ (EtOH) = 300 nm, λ_MAX_ (MeCN) = 314 nm; λ_Em_ (EtOH) = 505 nm, λ_Em_ (MeCN) = 508 nm. HRMS (DART/Orbitrap) *m*/*z*: [M + H]^+^ calcd for C_14_H_14_BN_3_O_2,_ 267.1179; found, 268.12518.
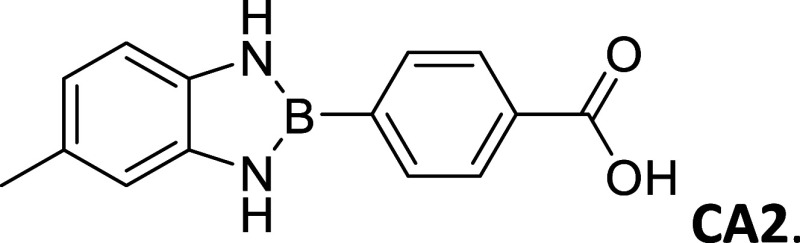


#### 2.8.5 2-(4′-Carboxyphenyl)-5-methylbenzo­[*d*]­1,3,2-diazaborole

Procedure B: 67% Yield, mp = 295–296 °C, Chalky white powder; IR spectrum (KBr), ν, cm^–1^: 3447, 3418, 1398, 1182, 1288. ^1^H NMR (301 MHz, DMSO-*d*_6_): δ 12.90 (s, 1H), 9.16 (d, *J* = 3.4 Hz, 2H), 8.08–7.88 (m, 4H), 6.94 (d, *J* = 7.8 Hz, 1H), 6.88 (s, 1H), 6.72–6.56 (m, 1H), 2.30 (s, 3H). ^13^C NMR (76 MHz, DMSO-*d*_6_): δ 167.50, 137.25, 134.87, 133.38, 131.12, 128.63, 127.16, 119.29, 111.64, 110.62, 21.17. λ_MAX_ (EtOH) = 300 nm; λ_Em_ (EtOH) = 370 nm. HRMS (DART/Orbitrap) *m*/*z*: [M + H]^+^ calcd for C_14_H_13_BN_2_O_2,_ 252.1070; found, 253.11428.
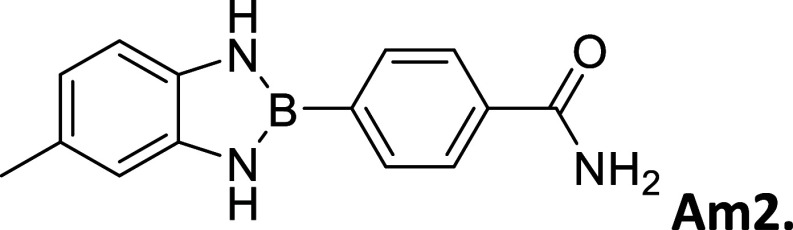


#### 2.8.6 2-(4′-Aminocarbonylphenyl)-5-methylbenzo­[*d*]­1,3,2-diazaborole

Procedure B: 60% yield. mp = 301–303 °C. Opalescent peach powder, IR spectrum (KBr), ν, cm^–1^: 3439, 3415, 1405, 1114, 1261. ^1^H NMR (301 MHz, DMSO-*d*_6_): δ 9.10 (d, *J* = 3.9 Hz, 2H), 7.92 (q, *J* = 8.0 Hz, 5H), 7.38 (s, 1H), 6.93 (d, *J* = 7.8 Hz, 1H), 6.87 (s, 1H), 6.64 (dd, *J* = 8.0, 1.5 Hz, 1H), 2.30 (s, 3H). ^13^C NMR (76 MHz, DMSO-*d*_6_): δ 168.42, 137.51, 135.22, 133.63, 127.33, 118.93, 111.41. λ_MAX_ (EtOH) = 312, λ_MAX_ (MeCN): 311 nm; λ_Em_ EtOH = 401 nm, λ_Em_ MeCN = 473 nm. HRMS (DART/Orbitrap) *m*/*z*: [M + H]^+^ calcd for C_14_H_14_BN_3_O, 251.1230; found, 252.13027.
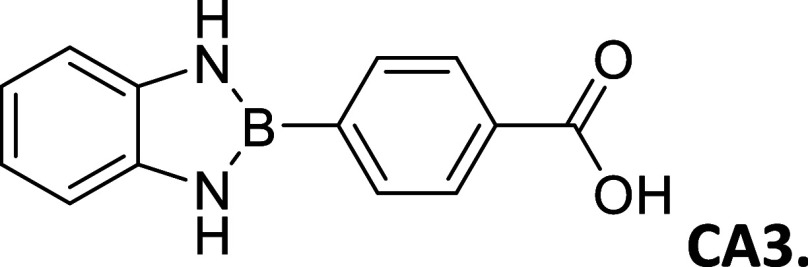


#### 2.8.7 2-(4′-Carboxyphenyl)­benzo­[*d*]­1,3,2-diazaborole

Procedure A: 83% Yield, mp = 291–293 °C, Opalescent white powder. ^1^H NMR (301 MHz, DMSO-*d*_6_): δ 16.04 (s, 1H), 12.84 (s, 2H), 11.84 (s, 1H), 11.44 (s, 3H), 10.62 (dt, *J* = 7.5, 3.8 Hz, 2H), 10.39 (dd, *J* = 5.7, 3.2 Hz, 2H). ^13^C (76 MHz, DMSO-*d*_6_): δ 167.51, 137.03, 134.12, 133.43, 128.65, 128.12, 118.57, 111.06. λ_MAX_ (MeCN) = 310 nm, λ_MAX_ (DMSO) = 307 nm; λ_Em_ (MeCN) = 475 nm.
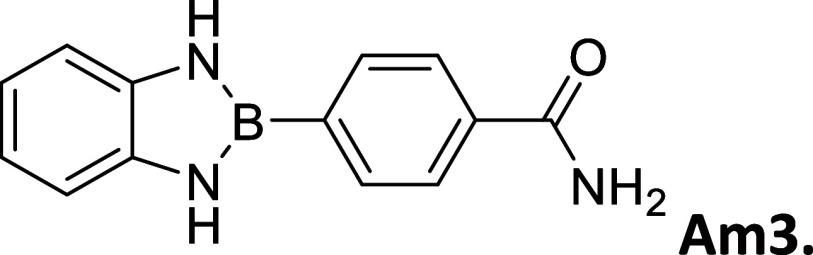


#### 2.8.8 2-(4′-Aminocarbonylphenyl)­Benzo­[*d*]­1,3,2-diazaborole

Procedure A: 64% yield. mp = 290–291 °C. Opalescent white powder. ^1^H NMR (301 MHz, DMSO-*d*_6_): δ 9.24 (s, 2H), 8.06–7.84 (m, 5H), 7.39 (s, 1H), 7.07 (dd, *J* = 5.7, 3.2 Hz, 2H), 6.83 (dd, *J* = 5.7, 3.2 Hz, 2H). ^13^C NMR (76 MHz, DMSO-*d*_6_): δ 167.96, 137.05, 134.75, 133.16, 126.87, 118.46, 110.94. λ_MAX_ (EtOH) = 298 nm, λ_MAX_ (MeCN) = 305 nm; λ_Em_ (EtOH) = 363 nm, λ_Em_ (MeCN) = 465 nm. HRMS (DART/Orbitrap) *m*/*z*: [M + H]^+^ calcd for C_13_H_12_BN_3_O, 237.1073; found, 238.11462.
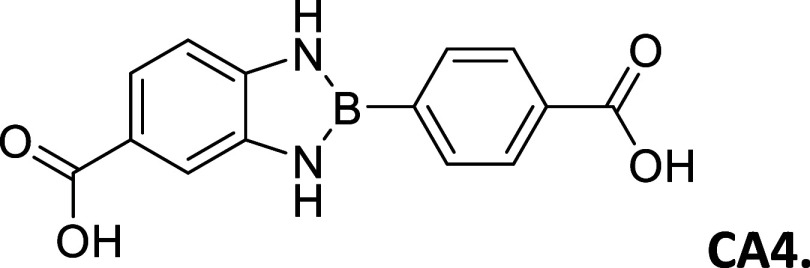


#### 2.8.9 2-(4′-Carboxyphenyl)-5-carboxybenzo­[*d*]­1,3,2-diazaborole

Procedure A: 79% Yield, mp = >300 °C, White powder; IR spectrum (KBr), ν, cm^–1^: 3459,3449, 1412, 1172, 1249. ^1^H NMR (301 MHz, DMSO-*d*_6_): δ 12.54 (s, 2H), 9.71 (s, 1H), 9.54 (s, 1H), 8.01 (d, *J* = 1.1 Hz, 4H), 7.68 (s, 1H), 7.63–7.51 (m, 1H), 7.13 (d, *J* = 8.2 Hz, 1H). ^13^C NMR (76 MHz, DMSO-*d*_6_): δ 168.24, 167.42, 141.25, 136.77, 133.58, 131.59, 128.74, 121.37, 120.97, 112.16, 110.48. λ_MAX_ (EtOH) = 319 nm, λ_MAX_ (MeCN) = 319 nm; λ_Em_ (EtOH) = 366 nm, λ_Em_ (MeCN) = 380 nm. HRMS (DART/Orbitrap) *m*/*z*: [M + H]^+^ calcd for C_14_H_11_BN_2_O_4,_ 282.0812; found, 283.08823.
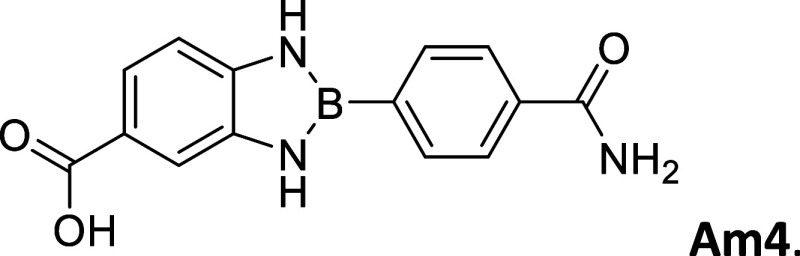


#### 2.8.10 2-(4′Aminocarbonylphenyl)-5-carboxybenzo­[*d*]­1,3,2-diazaborole

Procedure A: 25% yield. mp = 296–298 °C. Opalescent white powder, IR spectrum (KBr), ν, cm^–1^: 3430, 1335, 1373, 1400, 1431, 1111, 1174, 1217, 1240, 1267. ^1^H NMR (301 MHz, DMSO-*d*_6_): δ 9.24 (s, 2H), 8.02–7.87 (m, 5H), 7.39 (s, 1H), 7.07 (dd, *J* = 5.7, 3.2 Hz, 2H), 6.84 (dd, *J* = 5.7, 3.2 Hz, 2H). ^13^C NMR (76 MHz, DMSO-*d*_6_): δ 168.42, 137.51, 135.22, 133.63, 127.33, 118.93, 111.41. λ_MAX_ (EtOH) = 314 nm, λ_MAX_ (MeCN) = 313 nm, λ_MAX_ (DMSO) = 312 nm; λ_Em_ (EtOH) = 445 nm, λ_Em_ (DMSO) = 430 nm. HRMS (DART/Orbitrap) *m*/*z*: [M + H]^+^ calcd for C_14_H_12_BN_3_O_3,_ 281.0972; found, 282.10445.
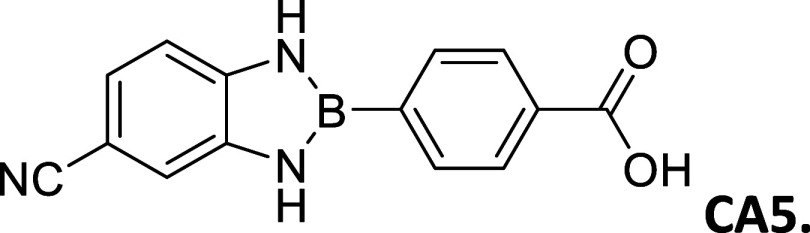


#### 2.8.11 2-(4′-Carboxyphenyl)-5-cyanobenzo­[*d*]­1,3,2-diazaborole

Procedure A: 32% Yield, mp = >300 °C, White powder; IR spectrum (KBr), ν, cm^–1^: 3455, 3398, 1420, 1294. ^1^H NMR (301 MHz, DMSO-*d*_6_): δ 12.14 (s, 1H), 9.78 (s, 1H), 9.61 (s, 1H), 8.08 (d, *J* = 7.8 Hz, 2H), 7.92 (d, *J* = 7.7 Hz, 2H), 7.70 (s, 1H), 7.58 (dd, *J* = 8.2, 1.6 Hz, 1H), 7.16 (d, *J* = 8.2 Hz, 1H). ^13^C NMR (76 MHz, DMSO-*d*_6_): δ 168.13, 141.05, 136.61, 134.01, 131.61, 121.44, 121.17, 118.99, 112.27, 111.98, 110.61. λ_MAX_ (MeCN) = 312 nm; λ_Em_ (MeCN) = 438 nm. HRMS (DART/Orbitrap) *m*/*z*: [M + H]^+^ calcd for C_14_H_10_BN_3_O_2,_ 263.0866; found, 264.09388.
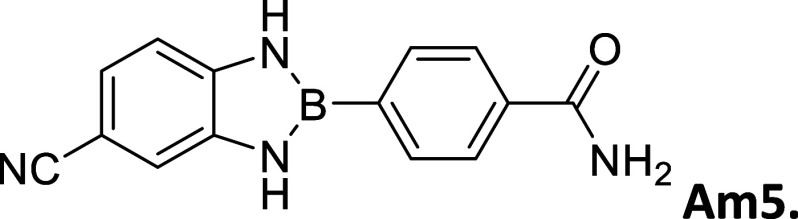


#### 2.8.12 2-(4′-Aminocarbonylphenyl)-5-cyanobenzo­[*d*]­1,3,2-diazaborole

Procedure A: 50% yield. mp = 287–289 °C White Powder. IR spectrum (KBr), ν, cm^–1^: 3327, 1317, 1405, 1116, 1189, 1287. ^1^H NMR (301 MHz, DMSO-*d*_6_): δ 9.86 (s, 1H), 9.69 (s, 1H), 8.06–7.90 (m, 5H), 7.42 (d, *J* = 1.6 Hz, 2H), 7.29 (d, *J* = 1.6 Hz, 1H), 7.23 (s, 1H). ^13^C NMR (76 MHz, DMSO-*d*_6_): δ 167.90, 141.32, 137.21, 135.40, 133.46, 127.05, 123.77, 120.85, 113.89, 111.71, 99.99. λ_MAX_ (EtOH) = 312 nm, λ_MAX_ (MeCN) = 308 nm; λ_Em_ (EtOH) = 374 nm, λ_Em_ (MeCN) = 422 nm, λ_Em_ (DMSO) = 428 nm. HRMS (DART/Orbitrap) *m*/*z*: [M + H]^+^ calcd for C_14_H_11_BN_4_O, 262.1026; found, 263.10987.
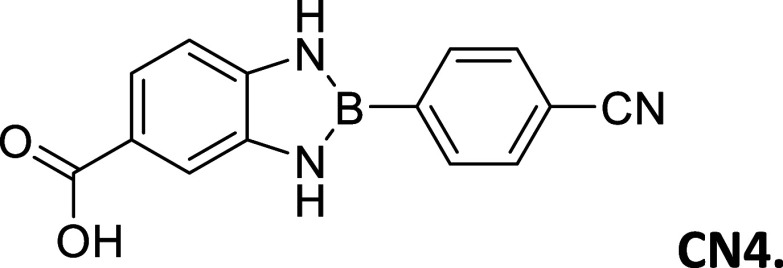


#### 2.8.13 2-(4′-Cyanophenyl)-5-carboxybenzo­[*d*]­1,3,2-diazaborole

Procedure A: 2% Yield, mp = >300 °C, White powder. ^1^H NMR (301 MHz, DMSO-*d*_6_): δ 12.14 (s, 1H), 9.78 (s, 1H), 9.61 (s, 1H), 8.08 (d, *J* = 7.8 Hz, 2H), 7.92 (d, *J* = 7.7 Hz, 2H), 7.70 (s, 1H), 7.58 (dd, *J* = 8.2, 1.6 Hz, 1H), 7.16 (d, *J* = 8.2 Hz, 1H). ^13^C NMR (76 MHz, DMSO-*d*_6_): δ 168.13, 141.05, 136.61, 134.01, 131.61, 121.44, 121.17, 118.99, 112.26, 111.98, 110.61. λ_MAX_ (EtOH) = 312 nm λ_MAX_ (MeCN) = 308 nm; λ_Em_ EtOH = 374 nm, λ_Em_ MeCN = 422 nm, λ_Em_ DMSO = 428 nm. HRMS (DART/Orbitrap) *m*/*z*: [M + H]^+^ calcd for C_14_H_10_BN_3_O_2,_ 263.0866; found, 263.09752.

## 3 Safety Information

N/A.

## 4 Conclusions

This study demonstrates that amide- and carboxy-substituted 2-phenyl-1,3,2-benzodiazaborole derivatives serve as structurally tunable pseudoaromatic scaffolds capable of forming stable CTCs. These materials exhibit large Stokes shifts and red-shifted fluorescence, particularly in the presence of strong EDGs, attributable to extended B–N delocalization. Spectroscopic anomalies, including NMR shielding and emission trends, support the formation of dimeric structures stabilized by π-stacking and donor–acceptor interactions. These insights reveal that pseudoaromatic amide- and carboxy-substituted 2-phenyl-1,3,2-benzodiazaborole derivatives possess significant promise for the development of functional materials in fluorescence-based sensing and optoelectronics.

## Supplementary Material



## Notes

Experimental procedures; spectroscopic data (^1^H, ^13^C, ^11^B, HSQC, and HMBC NMR spectra; infrared, UV–vis absorption, fluorescence emission, and excitation spectra); high resolution mass spectrometry (HRMS) data; computational details including frontier molecular orbital plots and optimized Cartesian coordinates; and additional figures and tables referenced in the main text (PDF)
