# Supplementary material for: Charge–Transfer Complexes and Fluorescence Modulation in Amide- and Carboxy-Substituted 2‑Phenyl-1,3,2-benzodiazaboroles
Source: ACS Omega. 2026 Jan 19;11(4):5248–60. doi: 10.1021/acsomega.5c07928 (PMC12878505; doi:10.1021/acsomega.5c07928)
Supplement: Supplementary file 1 [file ao5c07928_si_001.pdf]

Charge-Transfer Complexes and Fluorescence Modulation in Amide- and Carboxy-substituted 2-Phenyl-1,3,2-benzodiazaboroles

Supplementary Information

Table of Contents

Synthesis 4
Computational Methods 5
SI Figure 1: Am2 Hydrolysis 6
SI Table 1 7
00 8
^{1}H NMR 8
^{13}C NMR 9
Extended Scan ^{13}C NMR Showing Boronic *ipso* C 10
IR 11
Computational Data 12
CA1 13
^{13}C NMR 14
HSQC 15
HMBC 16
IR 17
UV-vis 18
Fluorescence Emission 18
HRMS 19
Computational Data 20
Am1 21
^{1}H NMR 21
^{13}C NMR 22
^{11}B NMR 23
IR 24
UV-vis 25
Fluorescence Emission 26
Fluorescence Excitation 27
HRMS 28
Computational Data 29
CA2 30
^{1}H NMR 30
^{13}C NMR 31
IR 32
HRMS 33
Computational Data 34
Am2 35
^{1}H NMR 35
^{11}B NMR 36
IR 37
UV-vis 38
Fluorescence Emission 39

Charge-Transfer Complexes and Fluorescence Modulation in Amide- and Carboxy-substituted 2-Phenyl-1,3,2-benzodiazaboroles

Supplementary Information

Fluorescence Excitation 40
HRMS 41
Computational Data 42
CA3 43
^{1}H NMR 43
^{13}C NMR 44
HSQC 45
HMBC 46
IR 47
Fluorescence Emission 50
Computational Data 51
Am3 52
^{1}H NMR 52
^{13}C NMR 53
HSQC 54
HMBC 55
IR 56
UV-vis 57
Fluorescence Emission 58
Fluorescence Excitation 59
HRMS 60
Computational Data 61
CA4 62
^{1}H NMR 62
^{13}C NMR 63
IR 64
UV-vis 65
Fluorescence Emission 66
Fluorescence Excitation 67
HRMS 68
Computational Data 69
CN4 70
^{1}H NMR 70
^{13}C NMR 71
IR 72
UV-vis 73
Fluorescence Emission 74
Fluorescence Excitation 75
HRMS 76
Computational Data 77
Am4 78
^{1}H NMR 78

Charge-Transfer Complexes and Fluorescence Modulation in Amide- and Carboxy-substituted 2-Phenyl-1,3,2-benzodiazaboroles

Supplementary Information

IR 79
UV-vis 80
Fluorescence Emission 81
Fluorescence Excitation 82
HRMS 84
Computational Data 85
CA5 86
^{1}H NMR 86
^{13}C NMR 87
IR 88
UV-vis 89
Fluorescence Emission 89
HRMS 90
Computational Data 91
Am5 92
^{1}H NMR 92
^{13}C NMR 93
^{11}B NMR 94
IR 95
UV-vis 96
Fluorescence Emission 97
Fluorescence Excitation 98
HRMS 99
Computational Data 100
Optimized Cartesian Coordinates 101
00 101
Am1 102
CA1 103
Am2 104
CA2 105
Am3 106
CA3 107
Am4 108
CA4 109
CN4 110
Am5 111
CA5 112
Am4 Dimer 113

Synthesis

All experiments except ^{11}B NMR and high resolution mass spectroscopy (HRMS) were conducted at SUNY Cortland; ^{11}B NMR and HRMS were acquired at Cornell University. Starting materials and reagents were obtained from Sigma Aldrich, Combi-Blocks, TCI, or AA Blocks and used without further purification. MWI reactions were carried out in a CEM Discover 2.0 Microwave Reactor using 10 mL Pyrex pressure vessels fitted with TFM septa vial caps. Melting point ranges were measured using a Stuart SMP 10 melting point apparatus and are reported uncorrected. UV-vis spectra were recorded on a Thermo Fisher Scientific Evolution 201 UV-Visible Spectrophotometer using quartz cuvettes. Fluorescence spectra were recorded on an Agilent Technologies Cary Eclipse Fluorescence Spectrophotometer using quartz cuvettes. IR spectra were obtained using a Thermo Scientific Nicolet iS50-FT-IR spectrometer. ^{1}H and ^{13}C NMR spectra were recorded on a 300 MHz Varian Avance II spectrometer; ^{11}B spectra were recorded on a 500 mHz Varian Avance III HD spectrometer. Samples were dissolved in DMSO-d_6, and chemical shifts are reported in ppm relative to tetramethylsilane (TMS) or DMSO-d_6 as an internal standard; ^{11}B NMR chemical shifts are reported without a standard. High Resolution Mass Spectrometry (HRMS) spectra were acquired on a DART-SVP (Direct Analysis in Real Time) ion source (IonSense, Saugus, MA) coupled to an Exactive Orbitrap mass spectrometer (Thermo Scientific, Bremen, Germany).

Computations were performed using the GAMESS software suite via the Chem Compute platform[32, 33]. Initial wavefunction estimations were obtained using the Restricted Hartree-Fock (RHF) method. Geometry optimizations were conducted at the density functional theory (DFT) level, employing the B3LYP functional with the 6-31G basis set. Graphics were rendered in JSmol[34].

The parent 2-phenyl-1,3,2-benzodiazaborole was synthesized according to literature methods[29].

Synthesis of Amide- or Carboxy-Substituted 2-Phenyl-1,3,2-Benzodiazaboroles. Amide derivatives were synthesized by combining 3,4-diaminobenzamide or *p*-amidophenylboronic acid (1.0 Eq.) with the corresponding substituted phenylboronic acid or phenylenediamine (1.2 – 1.4 Eq.) and heating at 115 °C for 10 min under microwave irradiation. Reactions were carried out under one of two solvent conditions:

<u>Condition A</u>: DMSO in diglyme 2 – 4 % v/v (3 – 4 mL) and triethylamine (20 mol%) were used for EWG-substituted phenylenediamine with all *p*-amido- or *p*-carboxyphenylboronic acids.

<u>Condition B</u>: PhMe / EtOAc (1 : 1) with triethylamine (20 mol%) was used for EDG-substituted phenylenediamine reactions.

Computational Methods

All quantum chemical calculations were performed using the GAMESS program package accessed via the ChemCompute web-based platform. Initial electronic structure calculations employed Restricted Hartree–Fock (RHF) wavefunctions for closed-shell systems. Geometry optimizations were subsequently carried out using Density Functional Theory (DFT) with the B3LYP hybrid functional in conjunction with the 6-31G(d) basis set unless otherwise noted.

Geometry optimizations were performed without symmetry constraints, and all optimized structures correspond to local minima on the potential energy surface, as confirmed by the absence of imaginary vibrational frequencies where frequency calculations were conducted. Solvent effects were not explicitly included unless stated; thus, all reported geometries and orbital energies correspond to gas-phase optimized structures.

Frontier molecular orbital energies (HOMO and LUMO) were extracted from the optimized wavefunctions, and HOMO–LUMO gaps ($\Delta E_{H\text{–}L}$) were calculated directly from the corresponding orbital eigenvalues. Visualization of molecular orbitals and electron density distributions was performed using JSmol.

Dimeric models were constructed from optimized monomer geometries and re-optimized at the same level of theory to probe intermolecular electronic interactions and potential charge-transfer complex (CTC) formation. Interaction energies were estimated by comparing total electronic energies of dimeric and monomeric species.

All quantum chemical calculations were performed using the GAMESS program package accessed via the ChemCompute web-based platform. Initial electronic structure calculations employed Restricted Hartree–Fock (RHF) wavefunctions for closed-shell systems. Geometry optimizations were subsequently carried out using Density Functional Theory (DFT) with the B3LYP hybrid functional in conjunction with the 6-31G(d) basis set unless otherwise noted. Geometry optimizations were performed without symmetry constraints, and all optimized structures correspond to local minima on the potential energy surface, as confirmed by the absence of imaginary vibrational frequencies where frequency calculations were conducted. Solvent effects were not explicitly included unless stated; thus, all reported geometries and orbital energies correspond to gas-phase optimized structures.

Frontier molecular orbital energies (HOMO and LUMO) were extracted from the optimized wavefunctions, and HOMO–LUMO gaps (ΔE_H–L) were calculated directly from the corresponding orbital eigenvalues. Visualization of molecular orbitals and electron density distributions was performed using JSmol.

Dimeric models were constructed from optimized monomer geometries and re-optimized at the same level of theory to probe intermolecular electronic interactions and potential charge-transfer complex (CTC) formation. Interaction energies were estimated by comparing total electronic energies of dimeric and monomeric species.

SI Figure 1: Am2 Hydrolysis

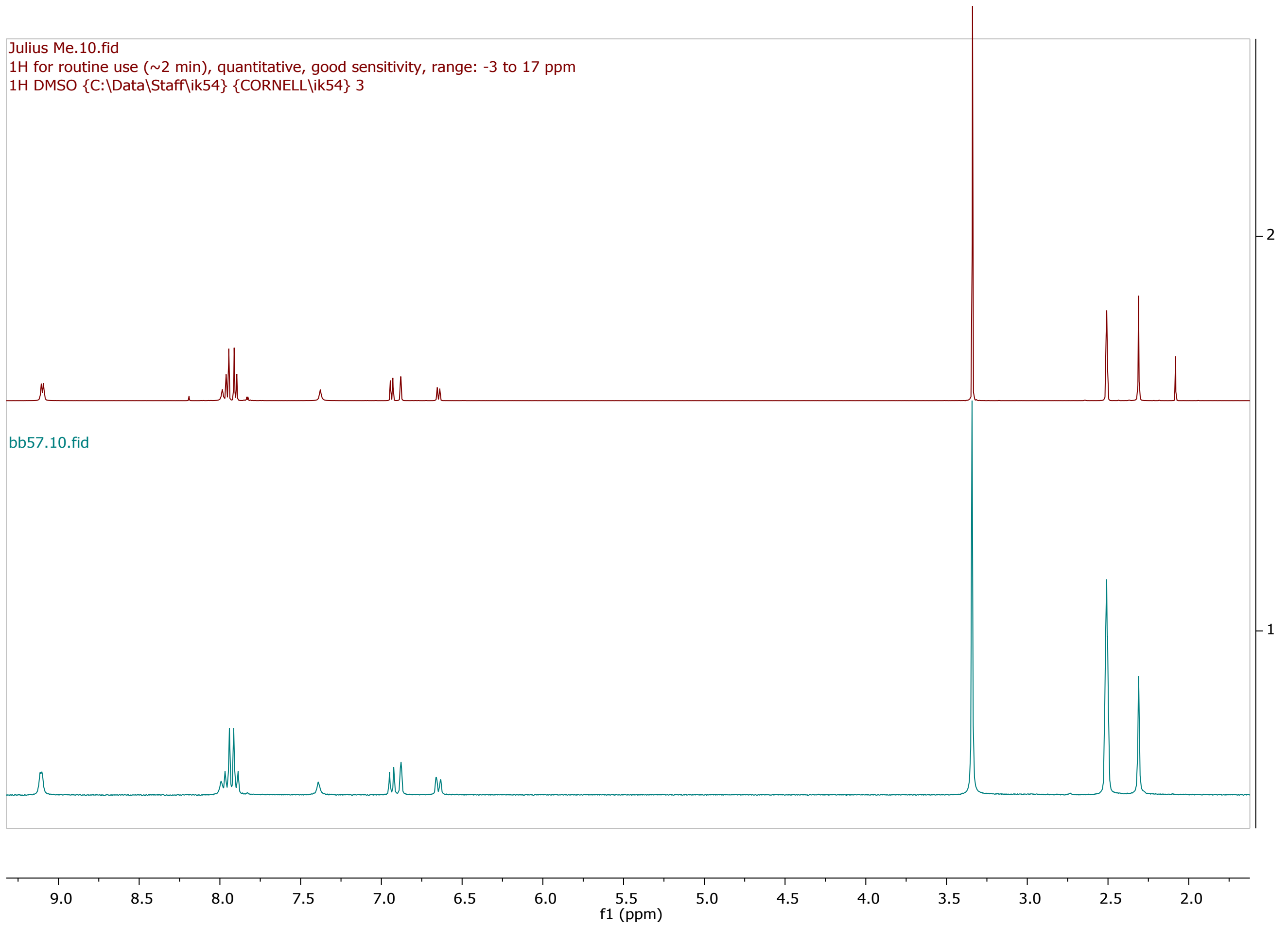

SI Figure 1. Stacked ^{1}H NMR spectrum of Am2 derivative illustrating potential air-induced hydrolysis. The bottom spectrum was recorded immediately after purification; the top spectrum was recorded 3 weeks post-synthesis. A new singlet (δ = 2.08 ppm), attributed to liberated methyl species, appears in the top spectrum, suggesting hydrolytic degradation. Similar behavior was observed I the **Am1** derivative. In contrast, derivatives bearing stronger EWGs exhibit greater resistance to hydrolysis under ambient conditions.

SI Table 1

SI Table 1. Stokes shifts (Δv, nm) for amide- and carboxy-substituted 2-phenyl-1,3,2-benzodiazaboroles measured in ethanol (EtOH), acetonitrile (MeCN), and dimethyl sulfoxide (DMSO). Values were calculated from the difference between absorption and emission maxima ($\Delta\nu = \lambda_{em} - \lambda_{abs}$). Not all compounds were evaluated in DMSO.

Compound	Solvent		
	EtOH (nm)	MeCN (nm)	DMSO (nm)
**00**	72	84	83
**CA1**	-----	201	-----
**Am1**	205	193	-----
**CA2**	70	-----	-----
**Am2**	88	163	-----
**CA3**		165	-----
**Am3**	65	159	-----
**CA4**	139	141	-----
**CN4**	124	125	-----
**Am4**	131	123	243
**CA5**	-----	126	-----
**Am5**	350	62	115

00

00

^{1}H NMR

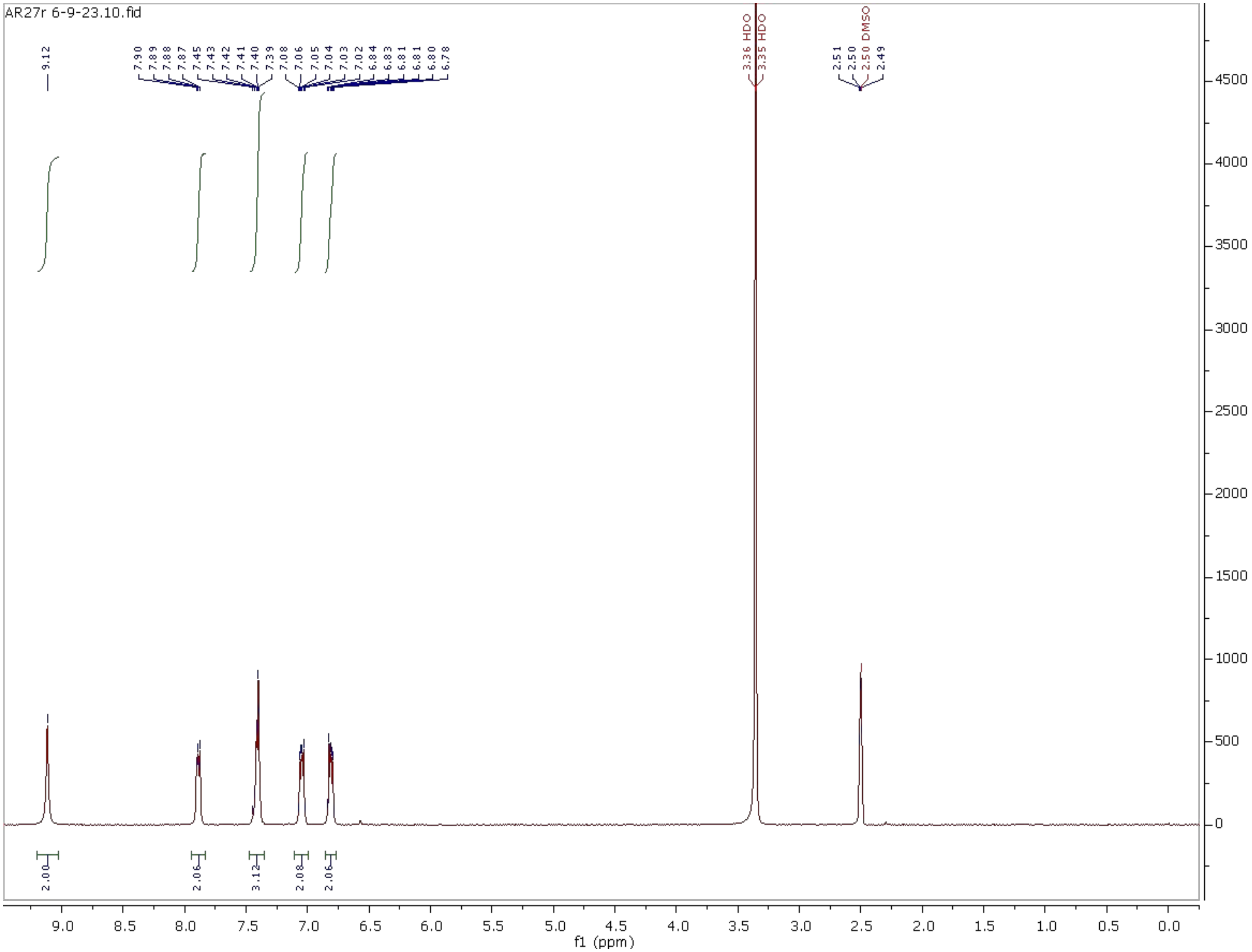
AR27r 6-9-23.10.fid
9.12
7.90
7.89
7.88
7.87
7.45
7.43
7.42
7.41
7.40
7.39
7.08
7.06
7.05
7.04
7.03
7.02
6.84
6.83
6.81
6.80
6.78
3.36 HDO
3.35 HDO
2.51
2.50
2.50 DMSO
2.49
2.00
2.06
3.12
2.08
2.06
f1 (ppm)

00

^{13}C NMR

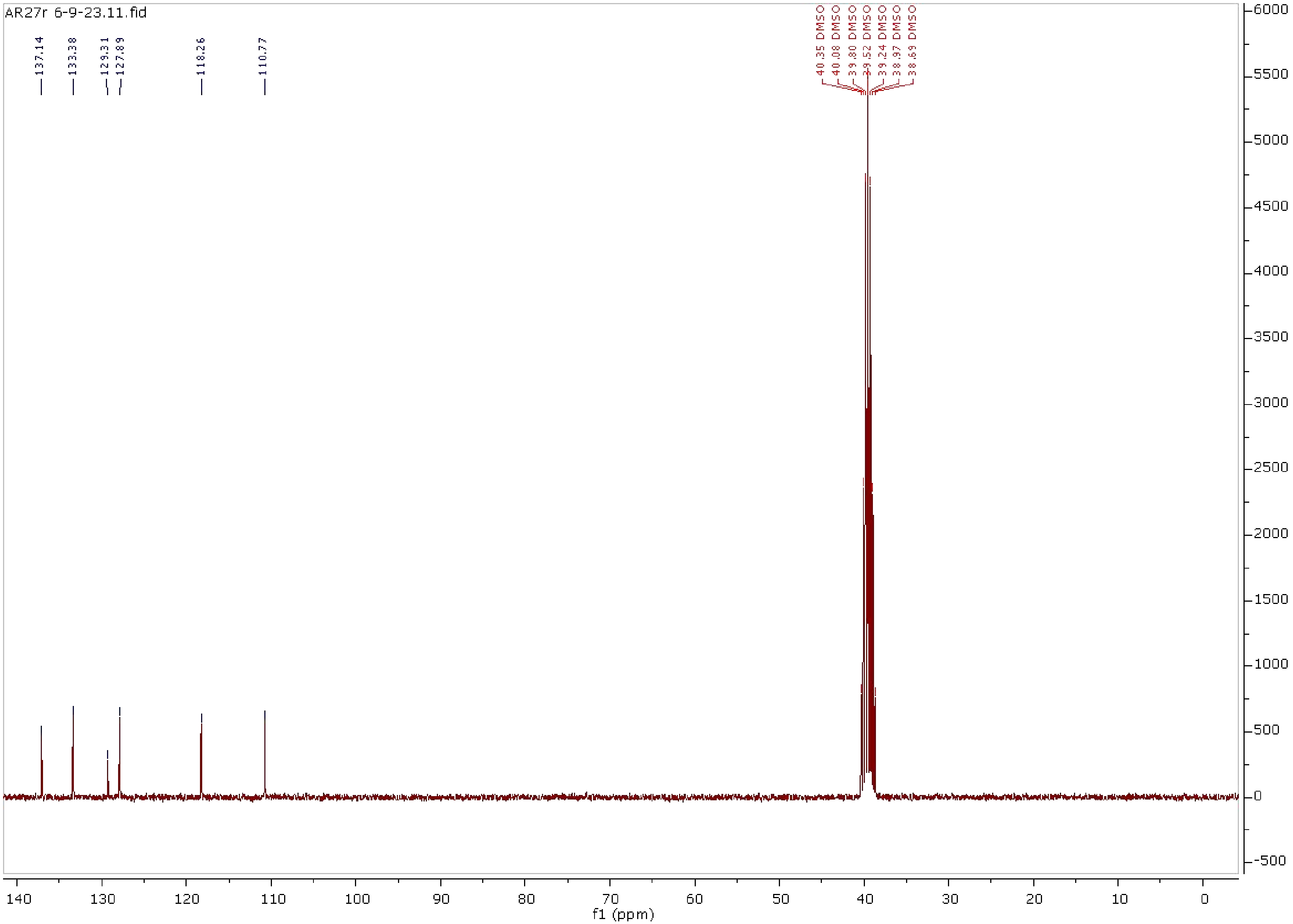
AR27r 6-9-23.11.fid
137.14
133.38
129.31
127.89
118.26
110.77
40.35 DMSO
40.08 DMSO
39.80 DMSO
39.52 DMSO
39.24 DMSO
38.97 DMSO
38.69 DMSO
f1 (ppm)

Extended Scan ^{13}C NMR Showing Boronic *ipso* C

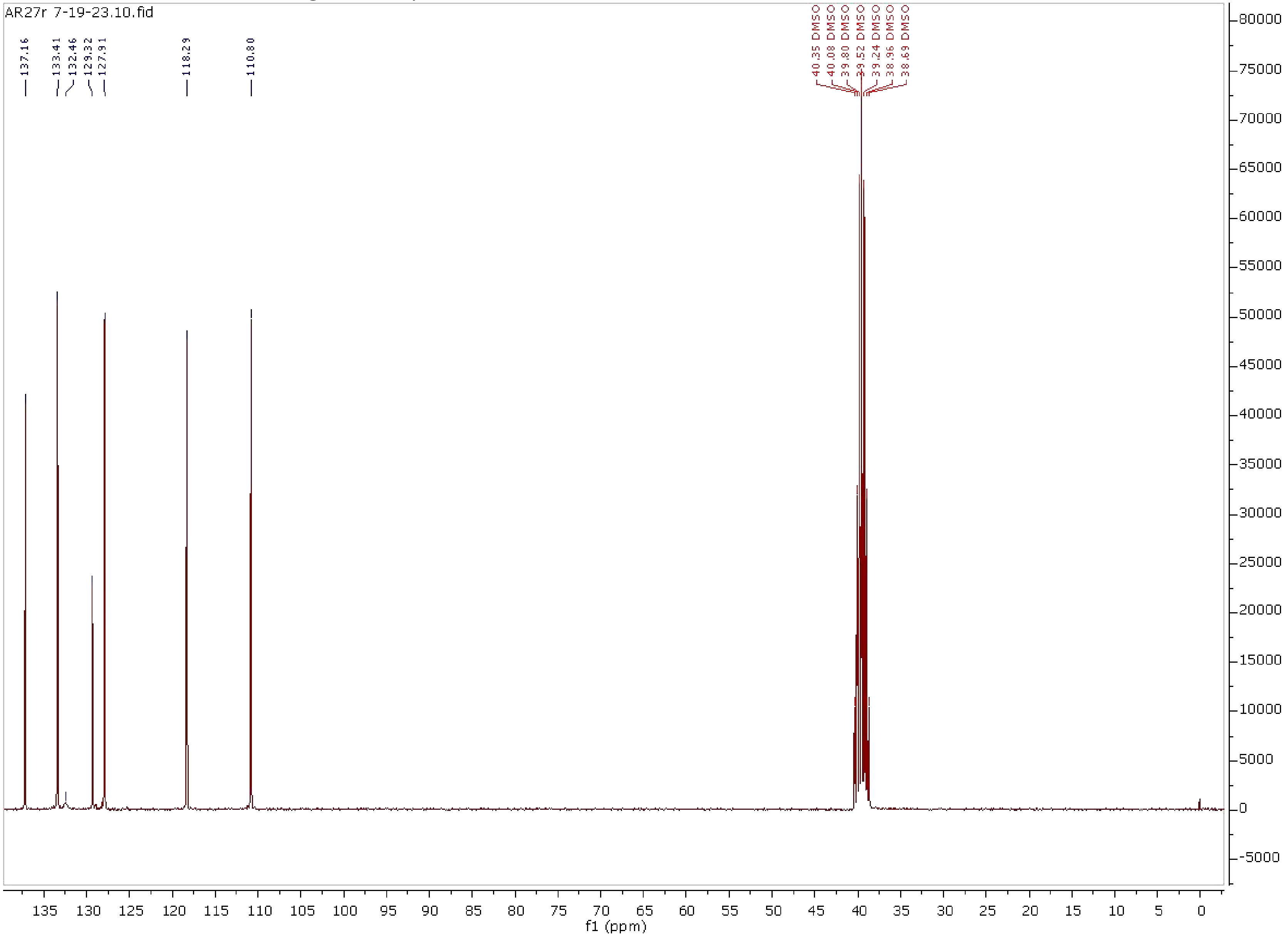

IR

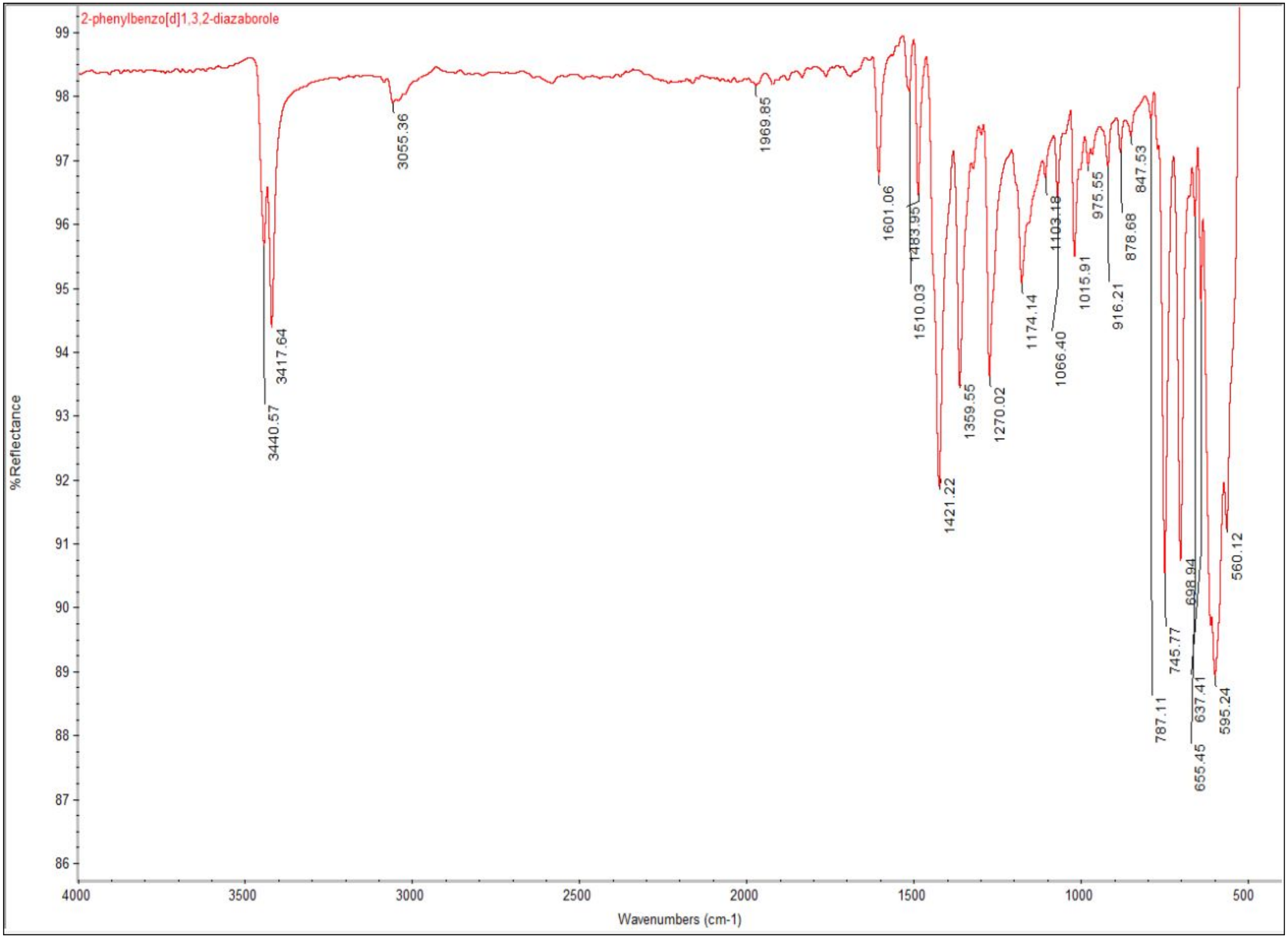
2-phenylbenzo[d]1,3,2-diazaborole
%Reflectance
Wavenumbers (cm-1)
3440.57
3417.64
3055.36
1969.85
1601.06
1483.95
1510.03
1421.22
1359.55
1270.02
1174.14
1103.18
1066.40
1015.91
975.55
916.21
878.68
847.53
787.11
745.77
698.94
655.45
637.41
595.24
560.12

Computational Data

MO 52/61
Energy = -0.0203 a.u.
Symmetry = A
Use the slider to adjust cutoff (start with 0.05)

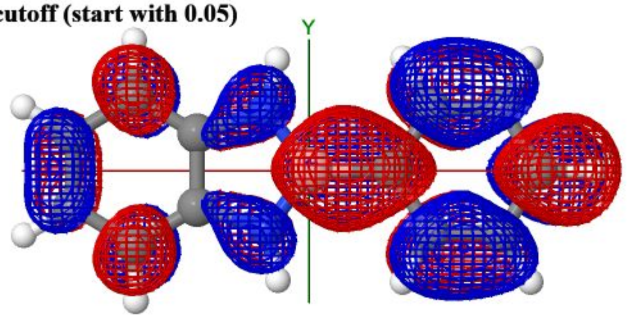

MO 51/61
Energy = -0.1912 a.u.
Symmetry = A
Use the slider to adjust cutoff (start with 0.05)

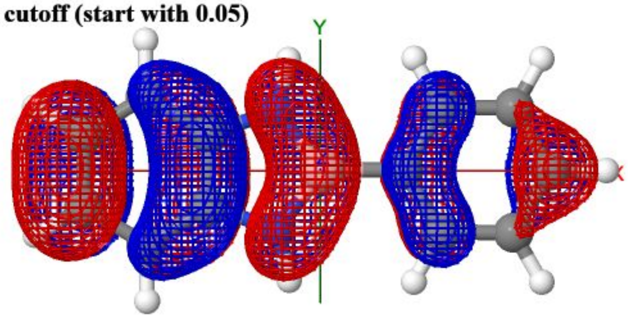

CA1

CA1

^{1}H NMR

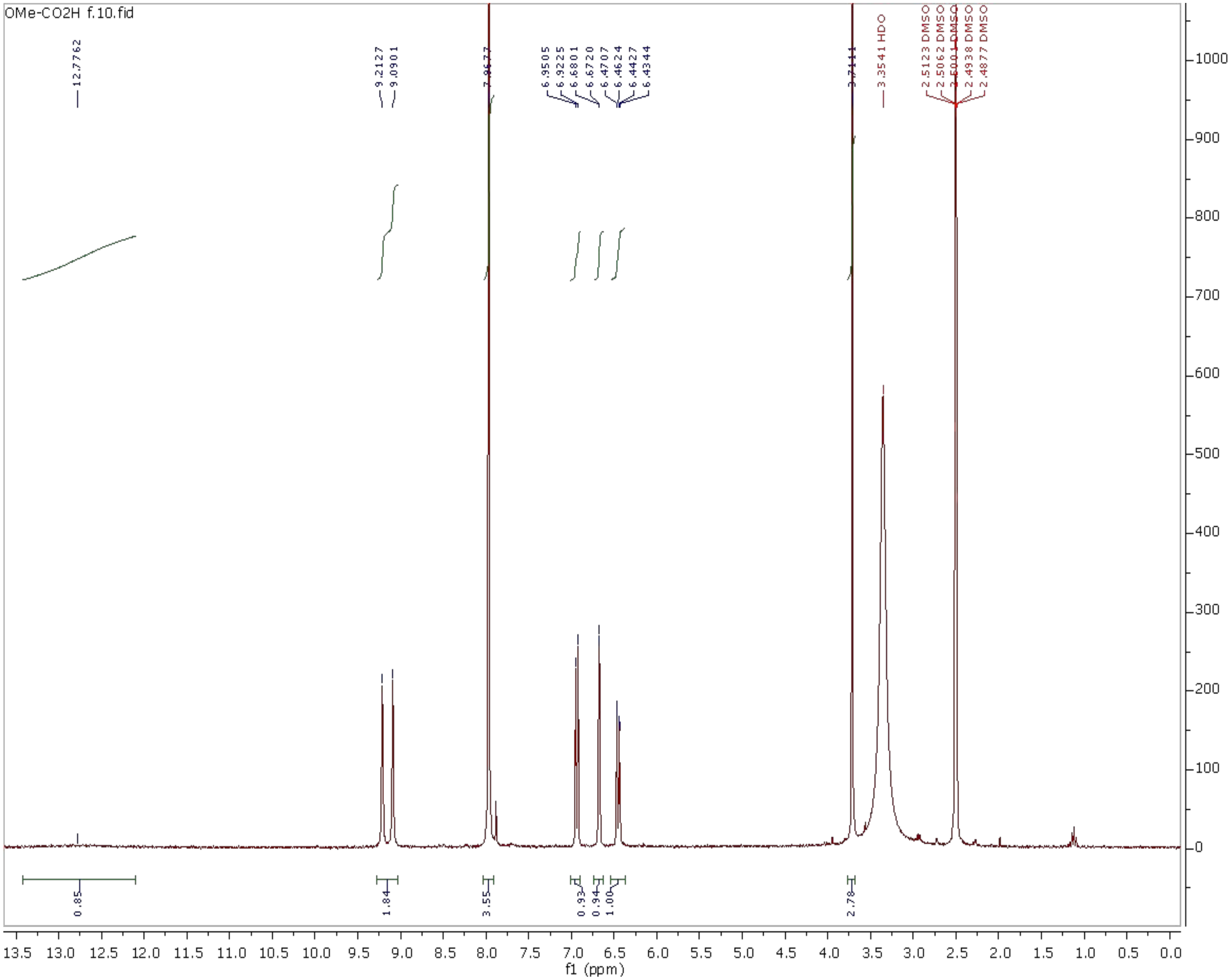

CA1

^{13}C NMR

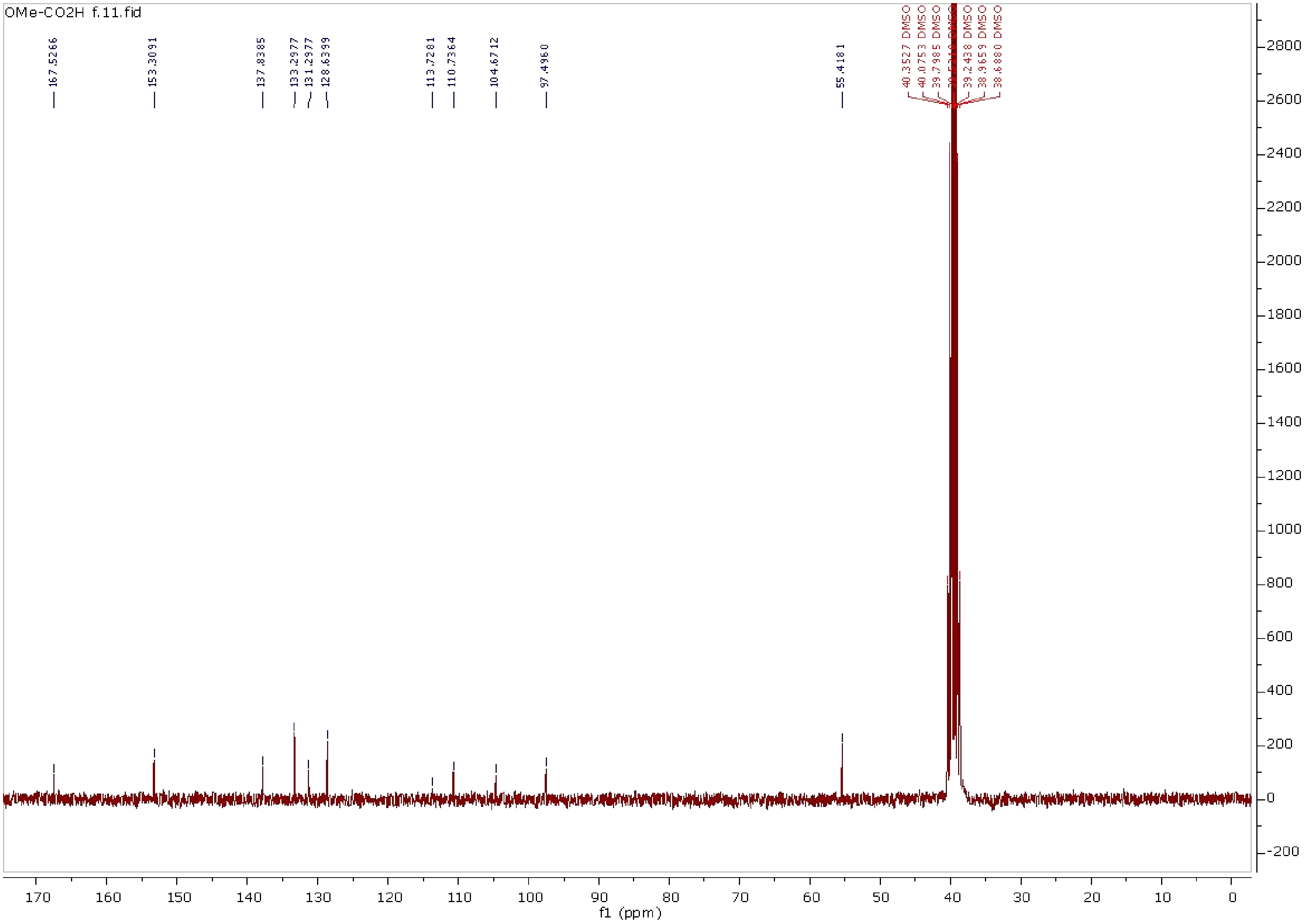

OMe-CO2H f.11.fid
167.5266
153.3091
137.8385
133.2977
131.2977
128.6399
113.7281
110.7364
104.6712
97.4960
55.4181
40.3527 DMSO
40.0753 DMSO
39.7985 DMSO
39.2438 DMSO
38.9659 DMSO
38.6880 DMSO
f1 (ppm)

CA1

HSQC

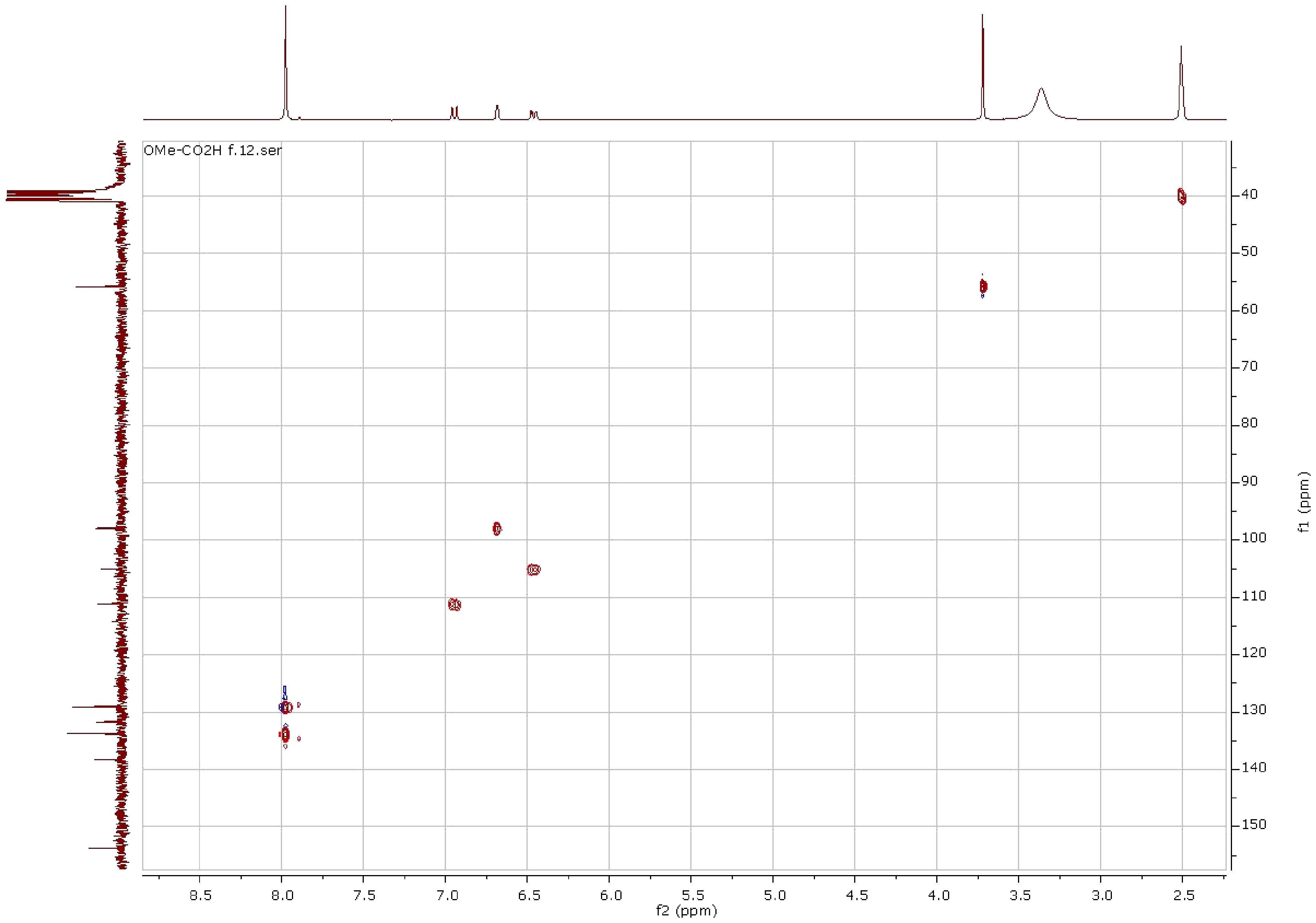

OMe-CO2H f.12.ser
f2 (ppm)
f1 (ppm)
8.5
8.0
7.5
7.0
6.5
6.0
5.5
5.0
4.5
4.0
3.5
3.0
2.5
40
50
60
70
80
90
100
110
120
130
140
150

CA1

HMBC

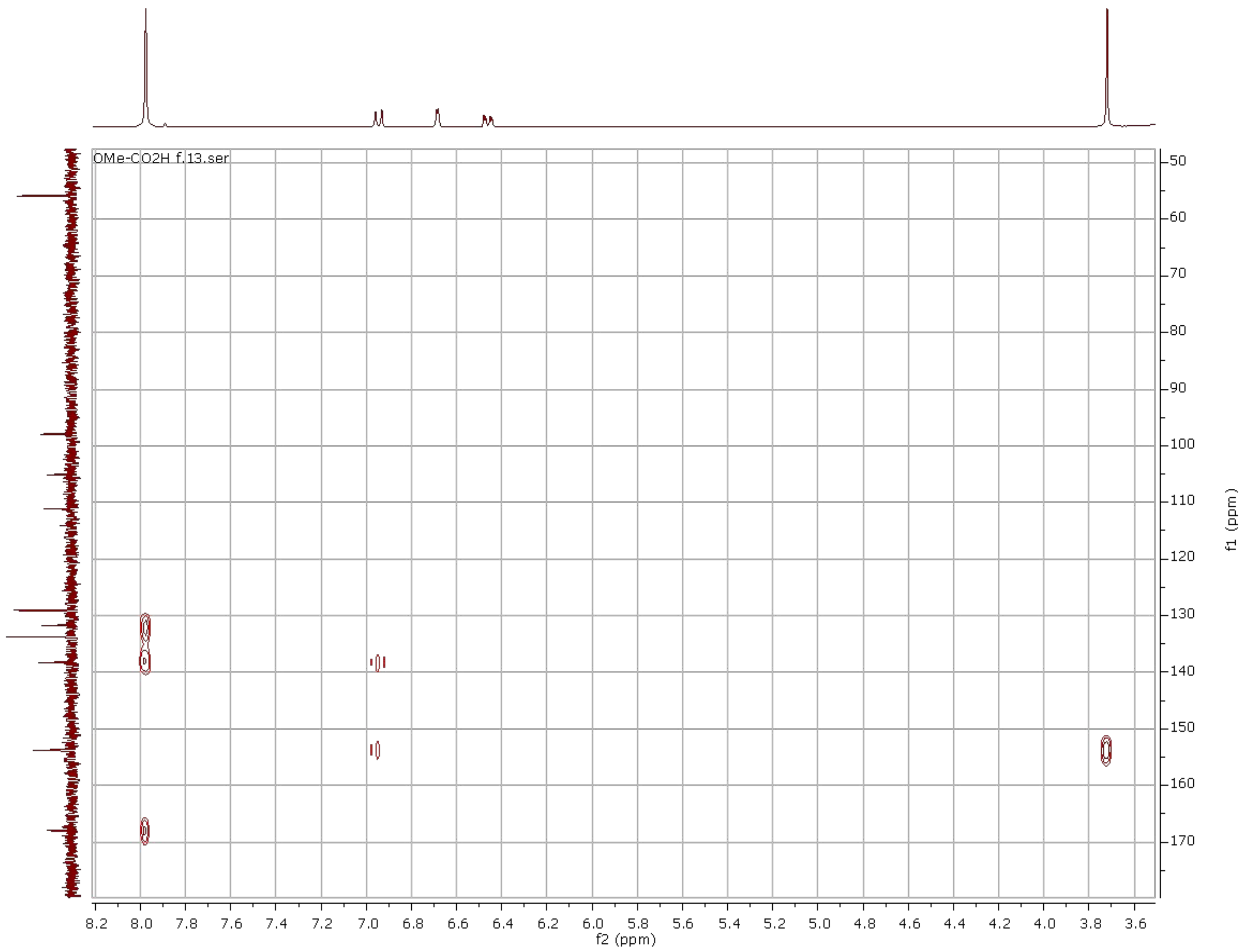

OMe-CO2H f.13.ser
8.2
8.0
7.8
7.6
7.4
7.2
7.0
6.8
6.6
6.4
6.2
6.0
5.8
5.6
5.4
5.2
5.0
4.8
4.6
4.4
4.2
4.0
3.8
3.6
f2 (ppm)
50
60
70
80
90
100
110
120
130
140
150
160
170
f1 (ppm)

CA1

IR

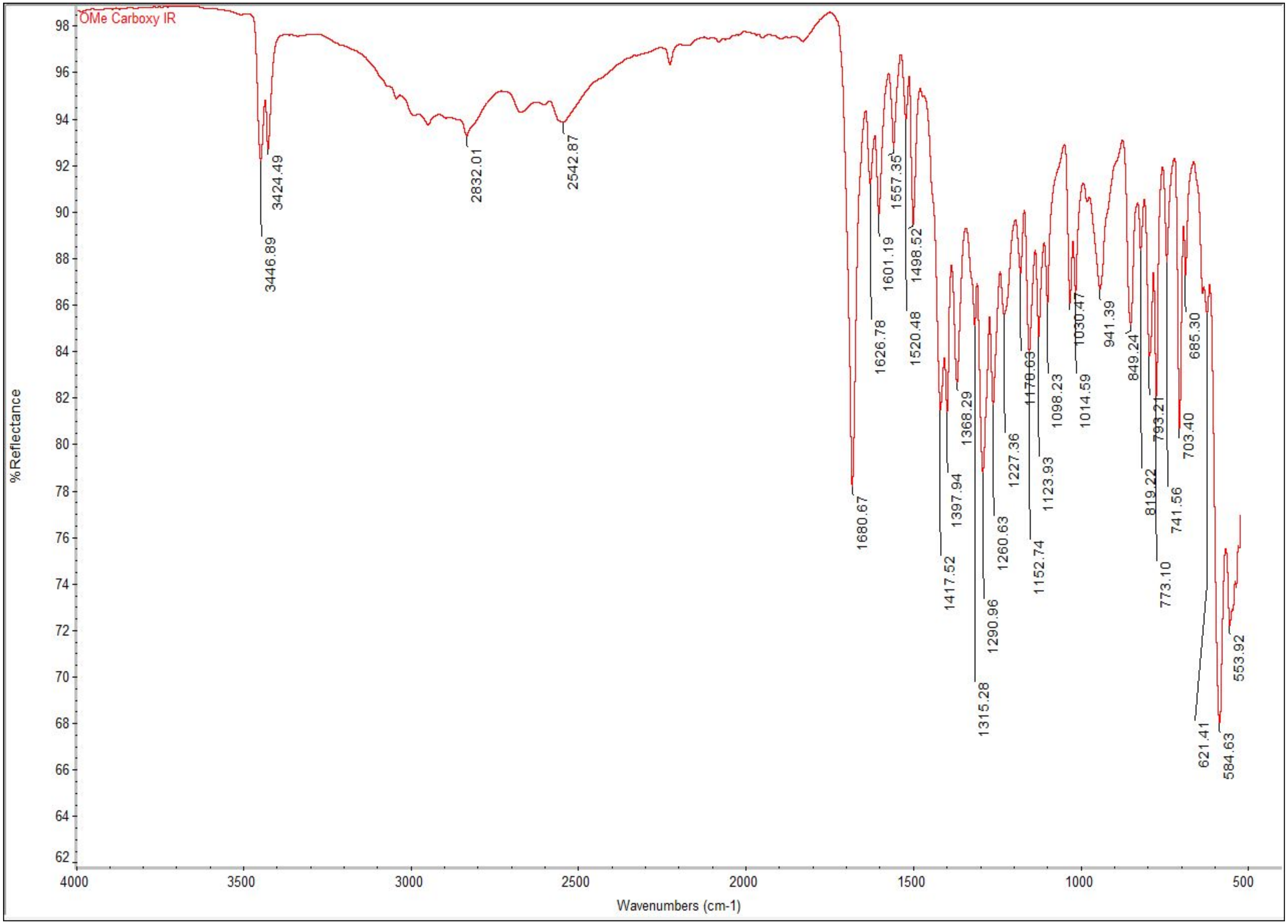

OMe Carboxy IR
%Reflectance
Wavenumbers (cm-1)
98
96
94
92
90
88
86
84
82
80
78
76
74
72
70
68
66
64
62
4000
3500
3000
2500
2000
1500
1000
500
3446.89
3424.49
2832.01
2542.87
1680.67
1626.78
1601.19
1557.35
1520.48
1498.52
1417.52
1397.94
1368.29
1315.28
1290.96
1260.63
1227.36
1178.63
1152.74
1123.93
1098.23
1030.47
1014.59
941.39
849.24
819.22
793.21
773.10
741.56
703.40
685.30
621.41
584.63
553.92

CA1

UV-vis

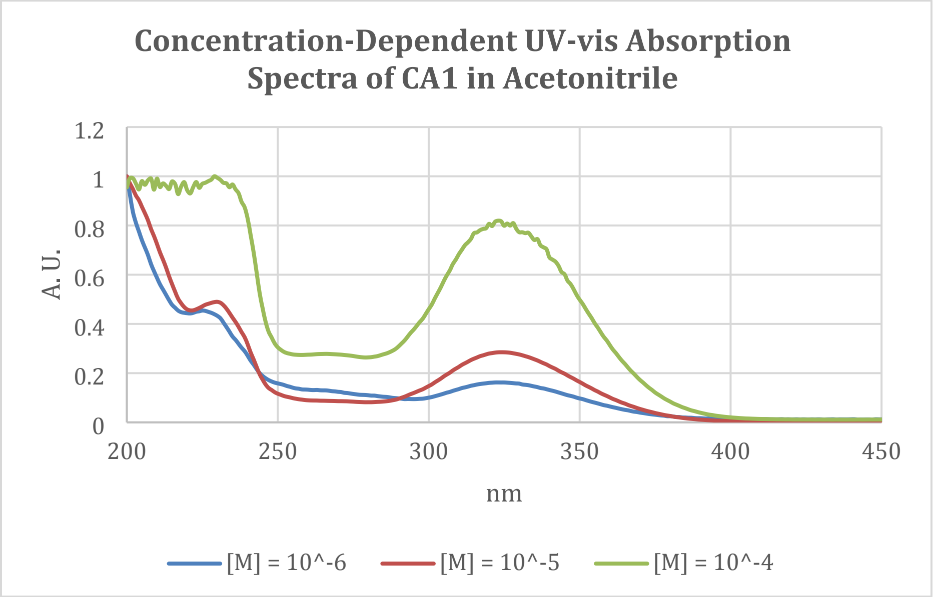

Fluorescence Emission

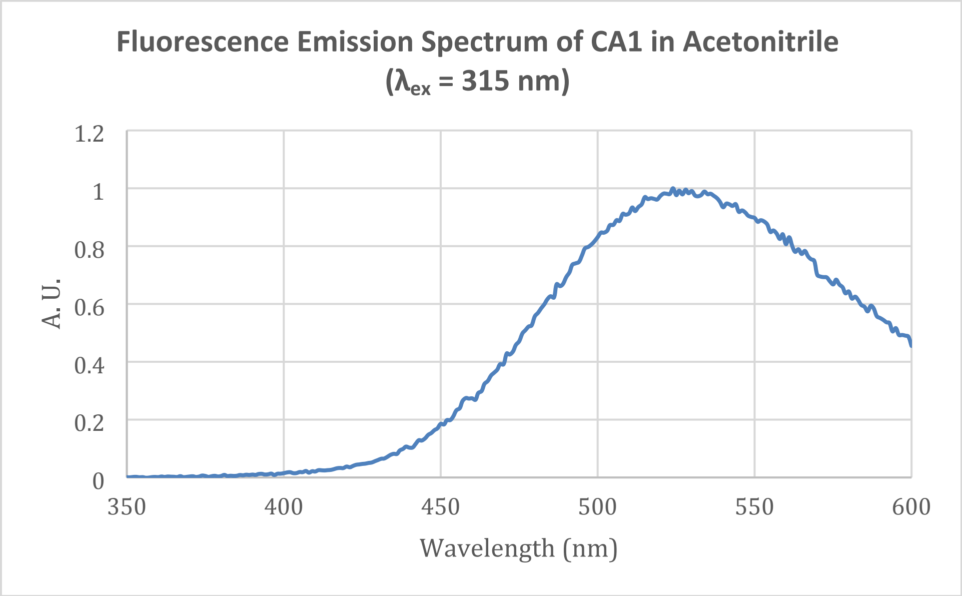

CA1

HRMS

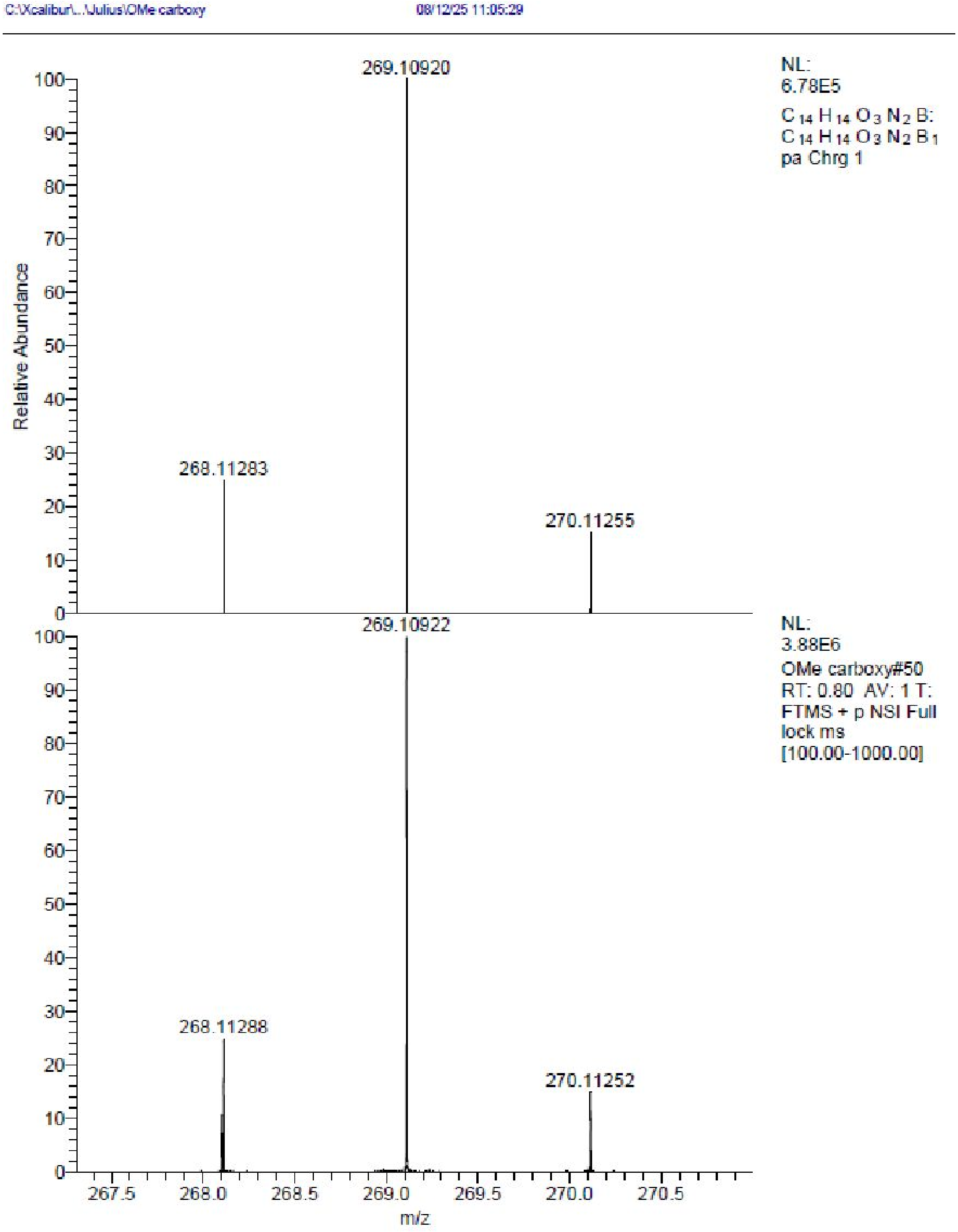

C:\Xcalibur\...\Julius\OMe carboxy
08/12/25 11:05:29
NL:
6.78E5
C 14 H 14 O 3 N 2 B:
C 14 H 14 O 3 N 2 B 1
pa Chrg 1
269.10920
268.11283
270.11255
NL:
3.88E6
OMe carboxy#50
RT: 0.80 AV: 1 T:
FTMS + p NSI Full
lock ms
[100.00-1000.00]
269.10922
268.11288
270.11252
Relative Abundance
m/z

CA1

Computational Data

MO 71/80
Energy = -0.0575 a.u.
Symmetry = A
Use the slider to adjust cutoff (start with 0.05)

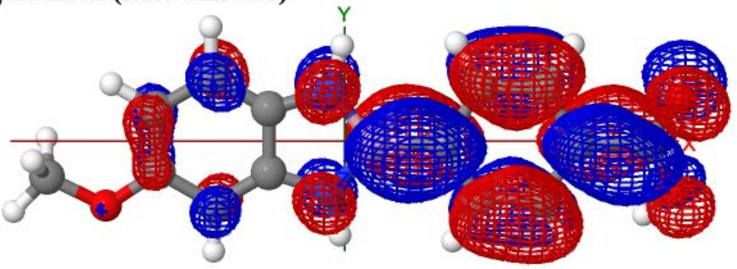

MO 70/80
Energy = -0.1897 a.u.
Symmetry = A
Use the slider to adjust cutoff (start with 0.05)

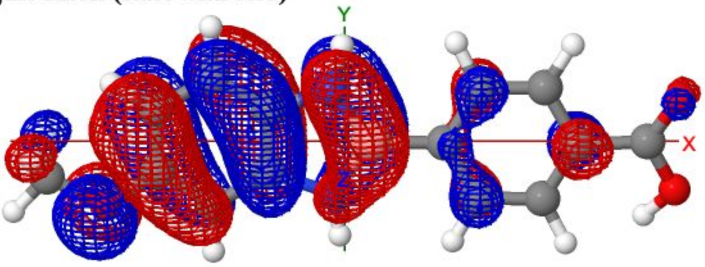

Am1

Am1

^1H NMR

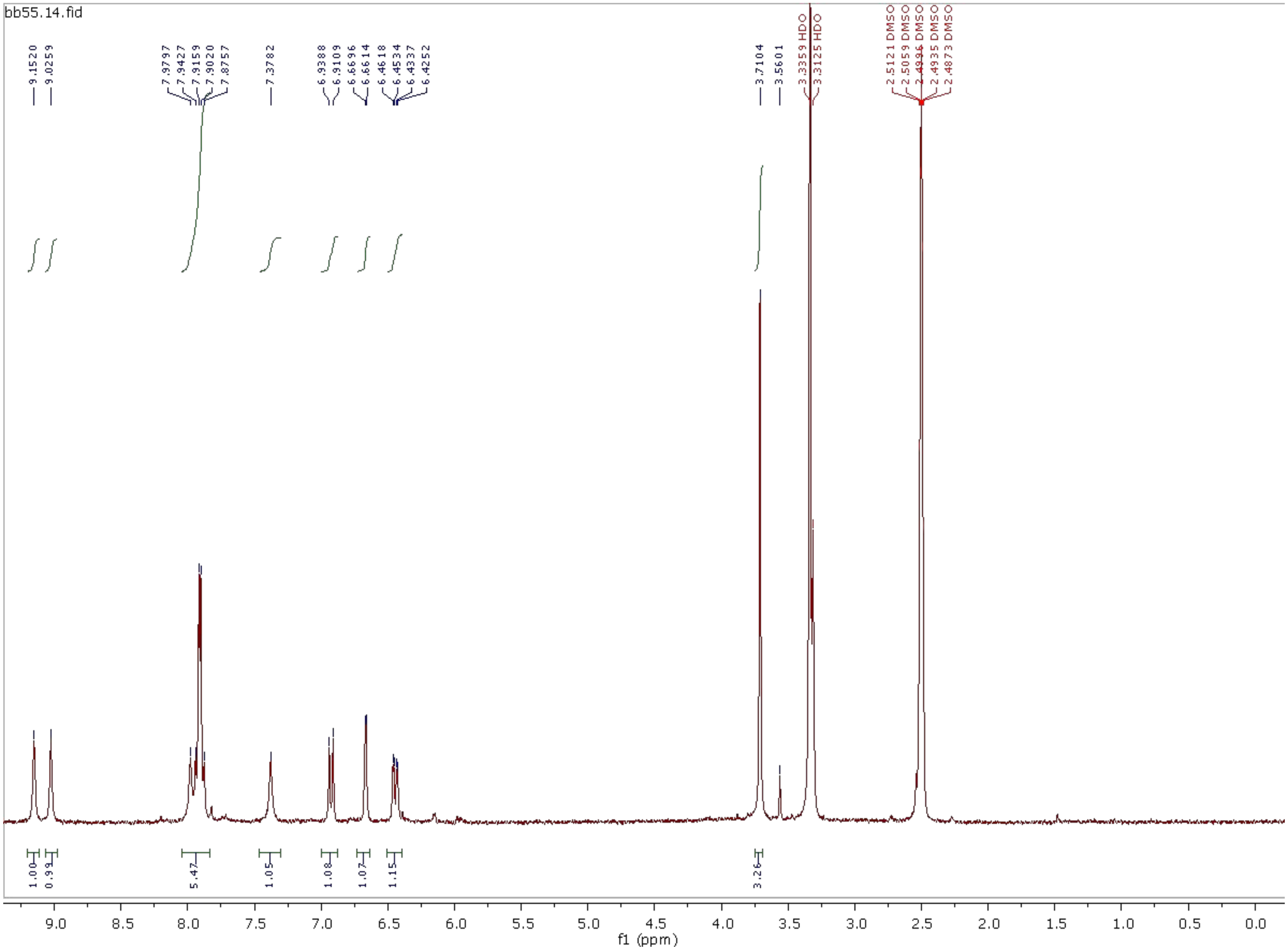

Am1

^{13}C NMR

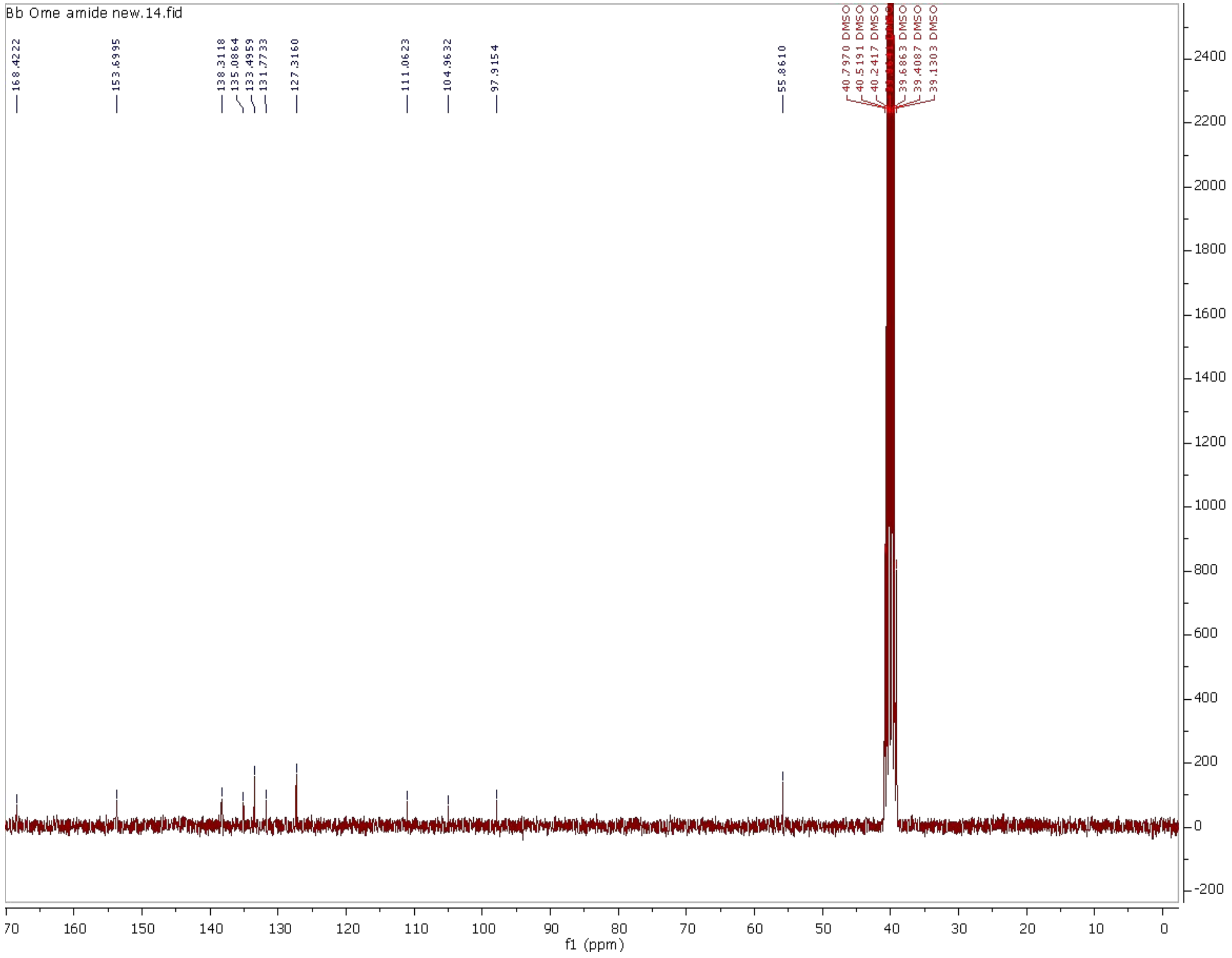
Bb Ome amide new.14.fid
168.4222
153.6995
138.3118
135.0864
133.4959
131.7733
127.3160
111.0623
104.9632
97.9154
55.8610
40.7970 DMSO
40.5191 DMSO
40.2417 DMSO
39.6863 DMSO
39.4087 DMSO
39.1303 DMSO
f1 (ppm)

Am1

^{11}B NMR

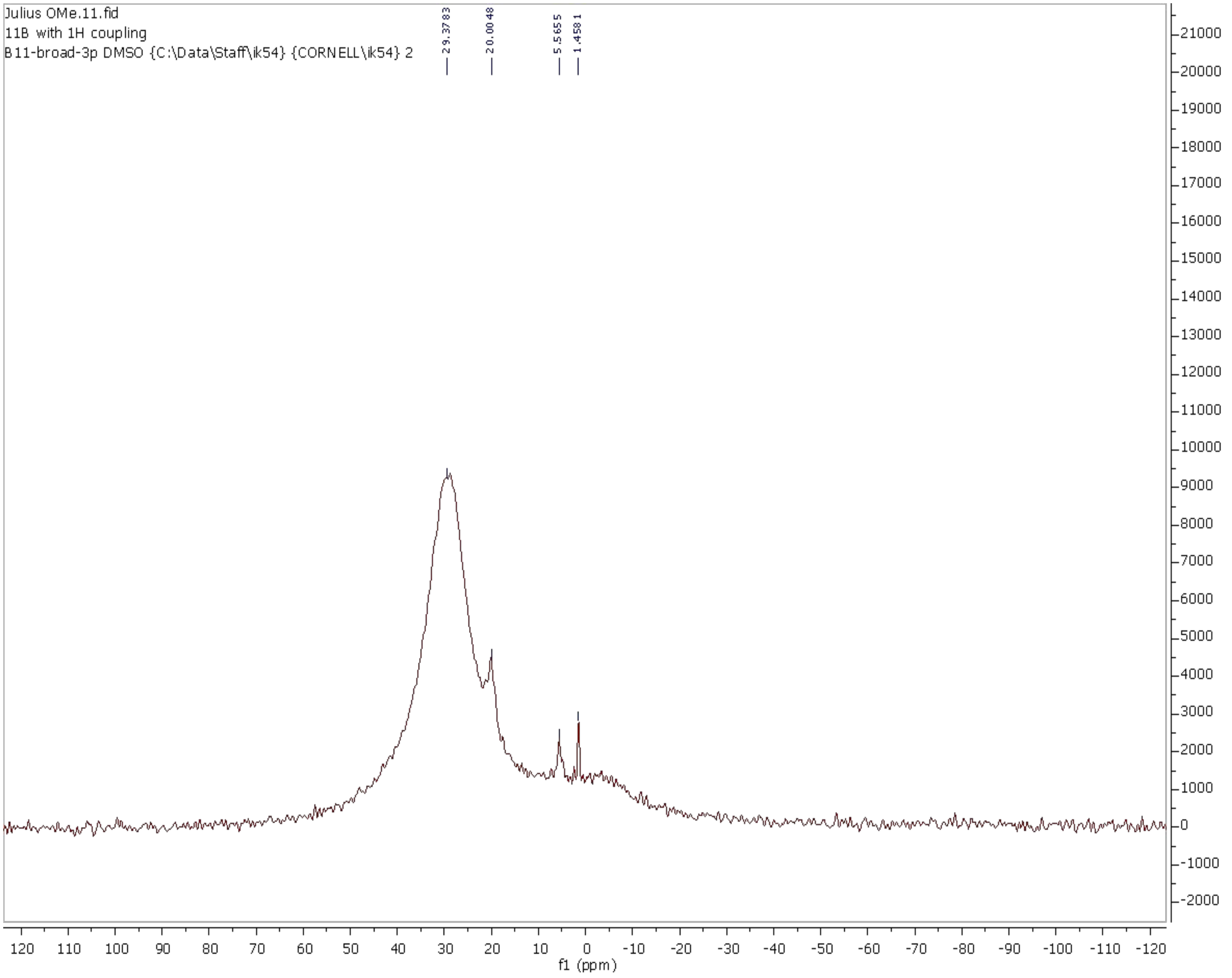

Julius OMe.11.fid
11B with 1H coupling
B11-broad-3p DMSO {C:\Data\Staff\ik54} {CORNELL\ik54} 2
29.3783
20.0048
5.5655
1.4581
f1 (ppm)

Am1

IR

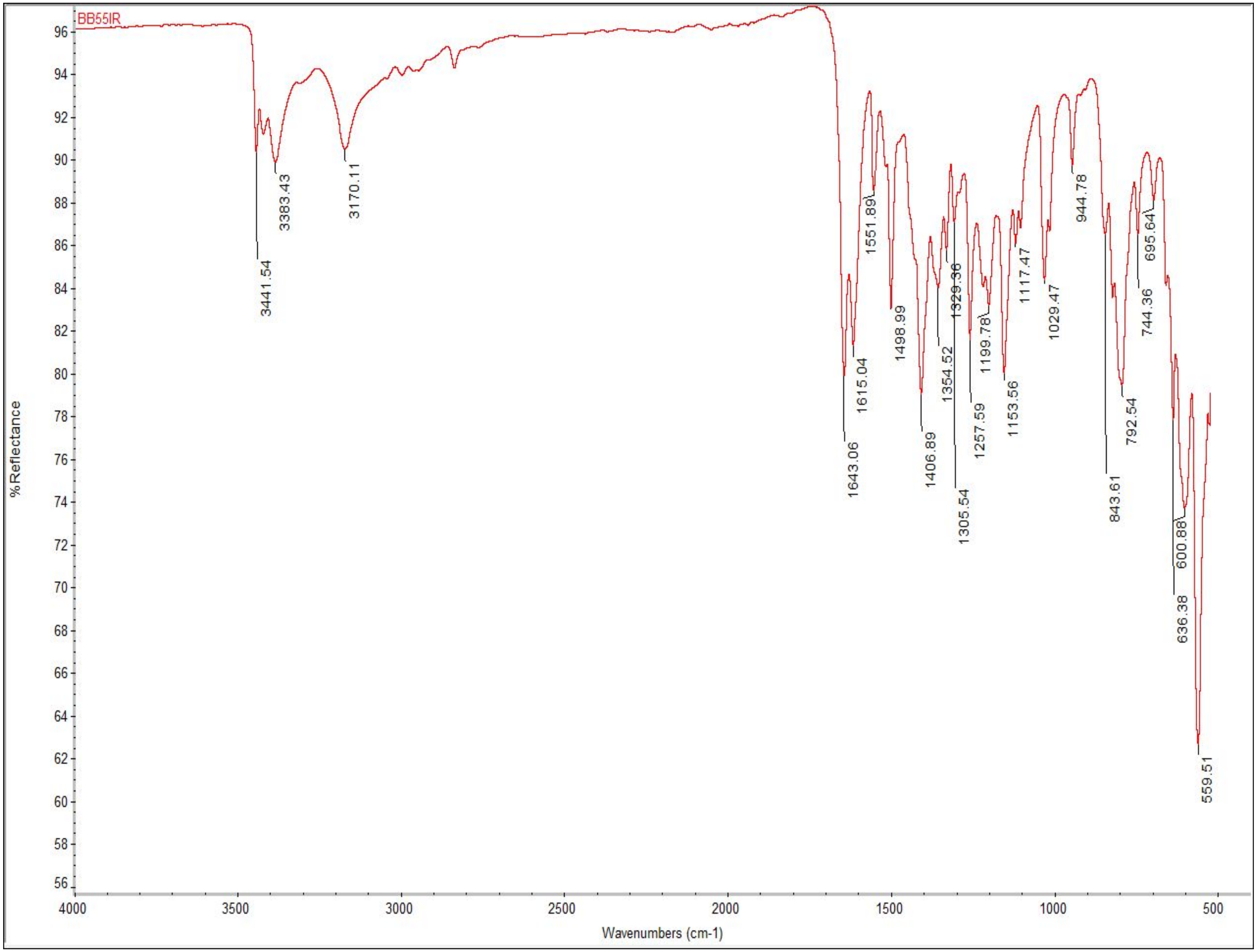
BB55IR
%Reflectance
Wavenumbers (cm-1)
4000
3500
3000
2500
2000
1500
1000
500
96
94
92
90
88
86
84
82
80
78
76
74
72
70
68
66
64
62
60
58
56
3441.54
3383.43
3170.11
1643.06
1615.04
1551.89
1498.99
1406.89
1354.52
1329.36
1305.54
1257.59
1199.78
1153.56
1117.47
1029.47
944.78
843.61
792.54
744.36
695.64
636.38
600.88
559.51

Am1

UV-vis

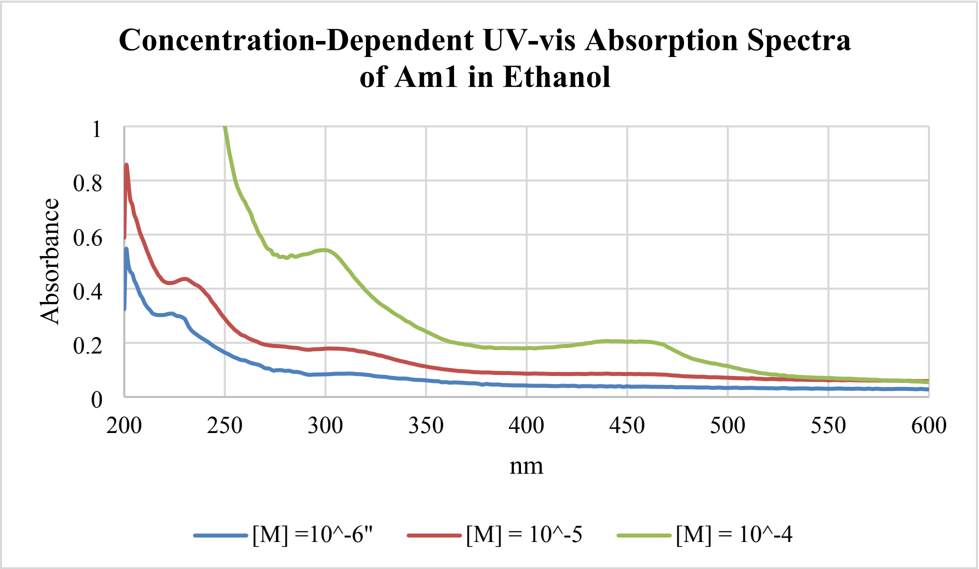

Concentration-Dependent UV-vis Absorption Spectra of Am1 in Ethanol
Absorbance
1
0.8
0.6
0.4
0.2
0
200
250
300
350
400
450
500
550
600
nm
[M] =10^-6"
[M] = 10^-5
[M] = 10^-4

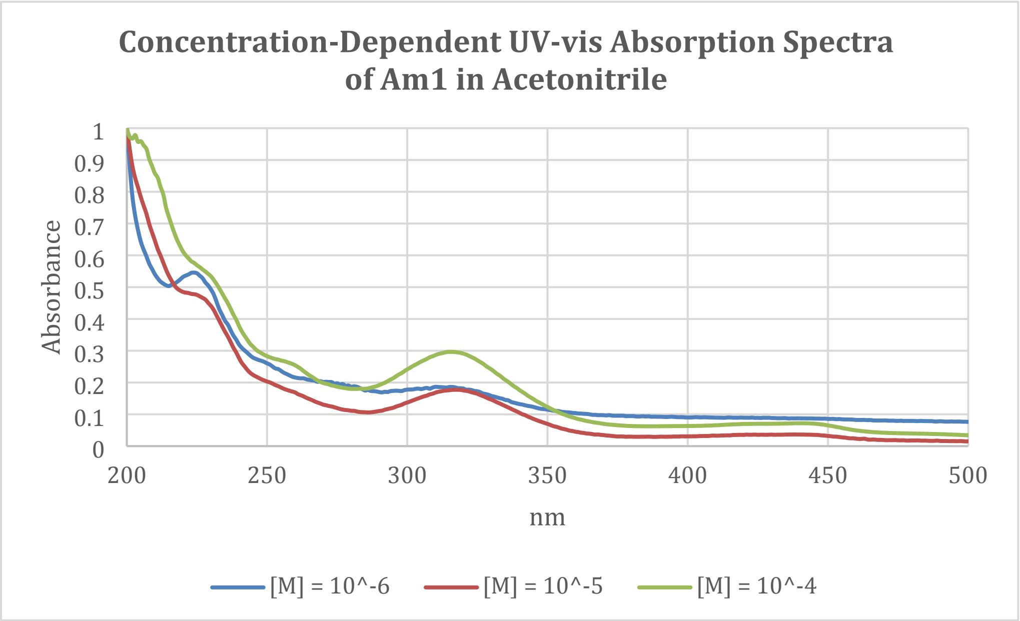

Concentration-Dependent UV-vis Absorption Spectra of Am1 in Acetonitrile
Absorbance
1
0.9
0.8
0.7
0.6
0.5
0.4
0.3
0.2
0.1
0
200
250
300
350
400
450
500
nm
[M] = 10^-6
[M] = 10^-5
[M] = 10^-4

Am1

Fluorescence Emission

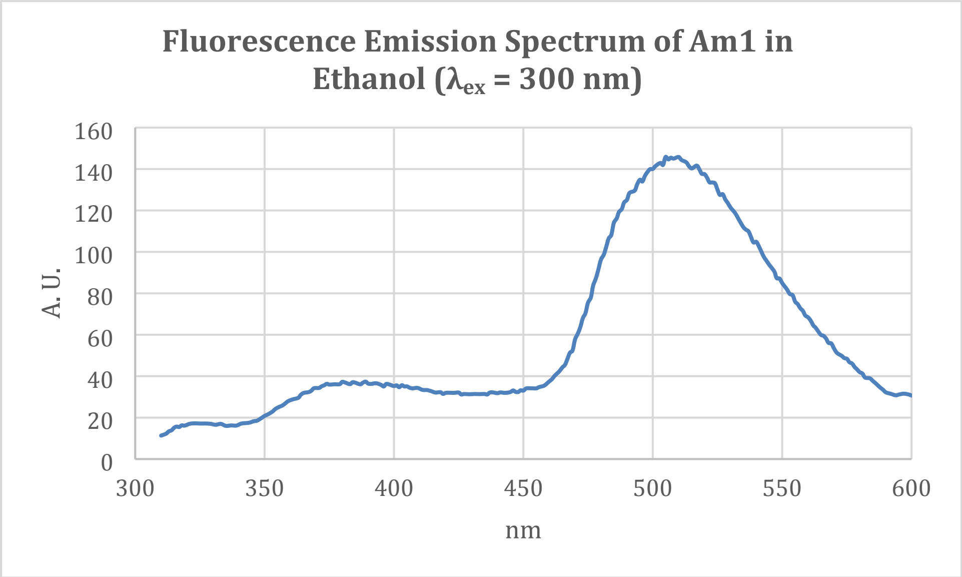

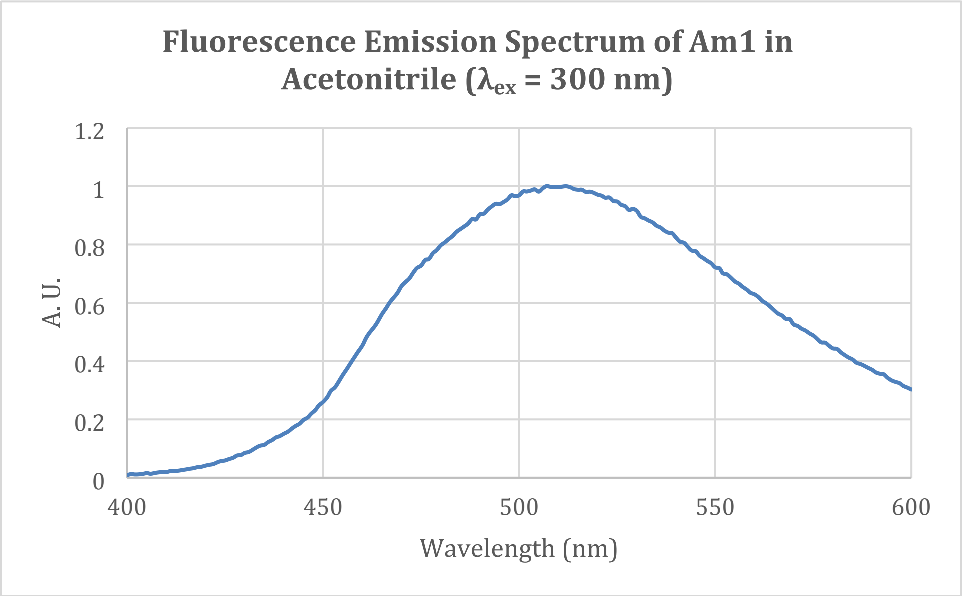

Am1

Fluorescence Excitation

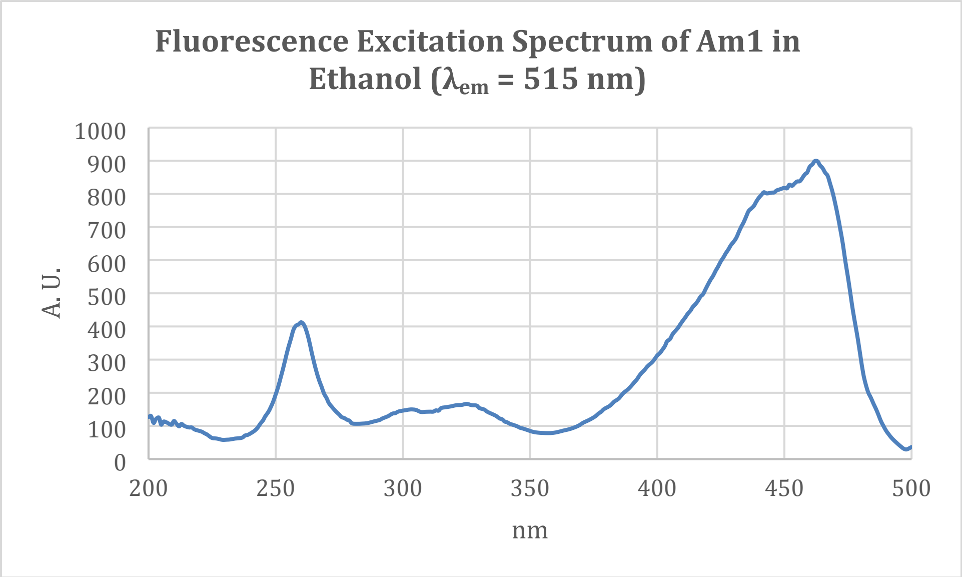

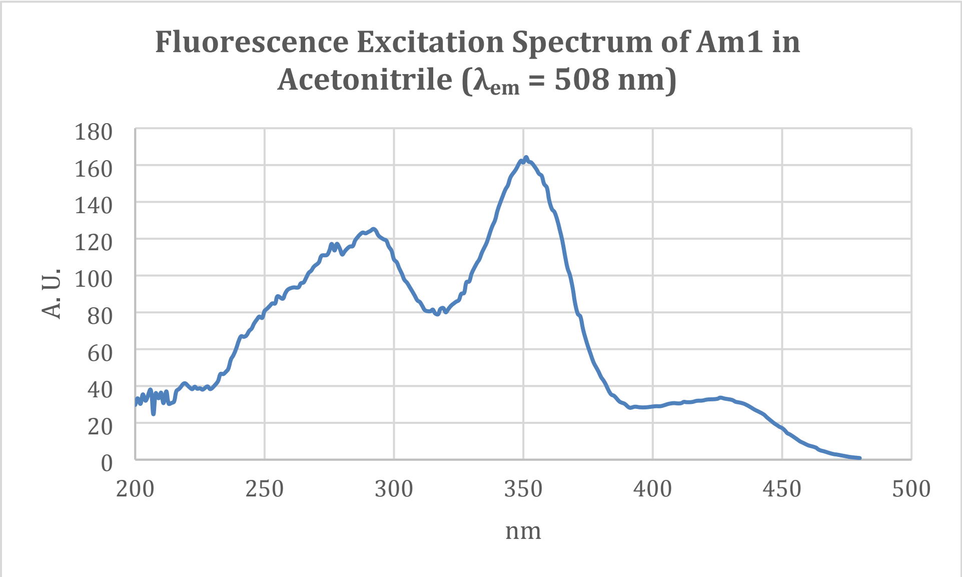

Am1

HRMS

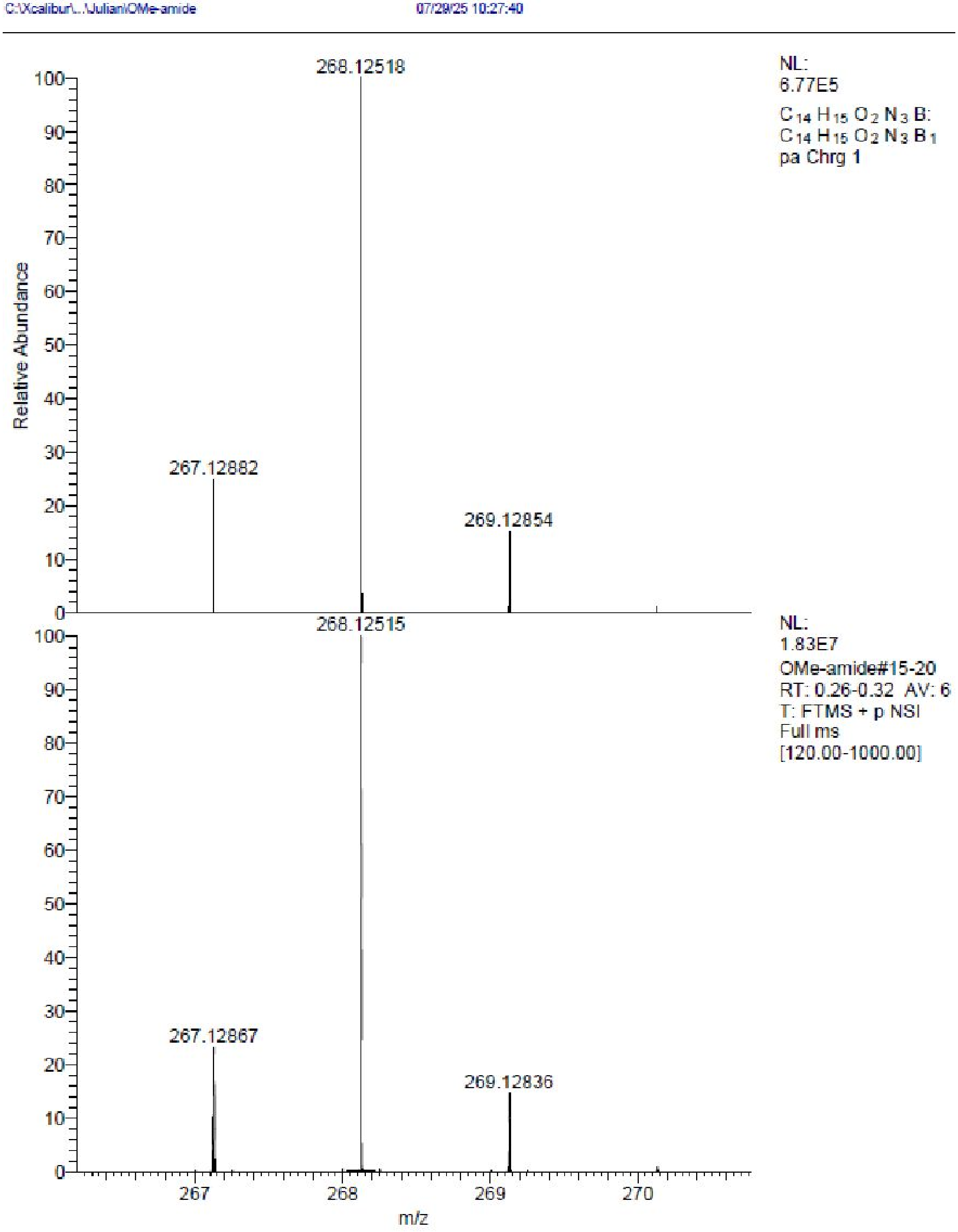
C:\Xcalibur\...\Julian\OMe-amide
07/29/25 10:27:40
268.12518
267.12882
269.12854
NL:
6.77E5
C 14 H 15 O 2 N 3 B:
C 14 H 15 O 2 N 3 B 1
pa Chrg 1
268.12515
267.12867
269.12836
NL:
1.83E7
OMe-amide#15-20
RT: 0.26-0.32 AV: 6
T: FTMS + p NSI
Full ms
[120.00-1000.00]
Relative Abundance
m/z

Am1

Computational Data

MO 71/80
Energy = -0.046 a.u.
Symmetry = A
Use the slider to adjust cutoff (start with 0.05)

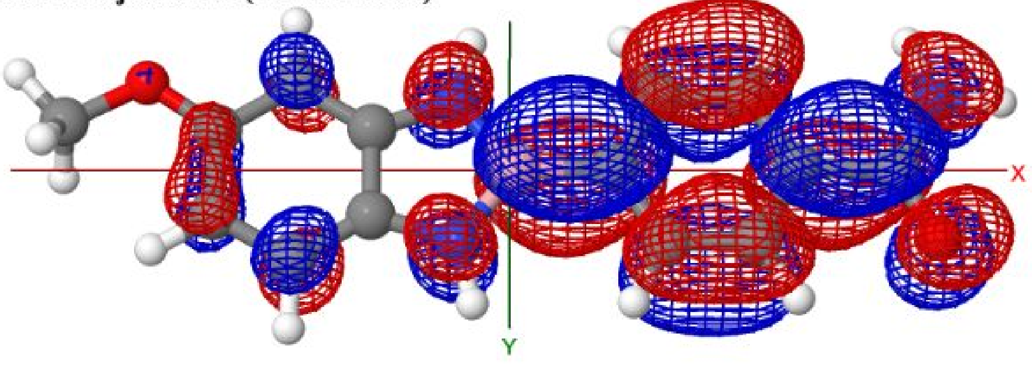

MO 70/80
Energy = -0.1844 a.u.
Symmetry = A
Use the slider to adjust cutoff (start with 0.05)

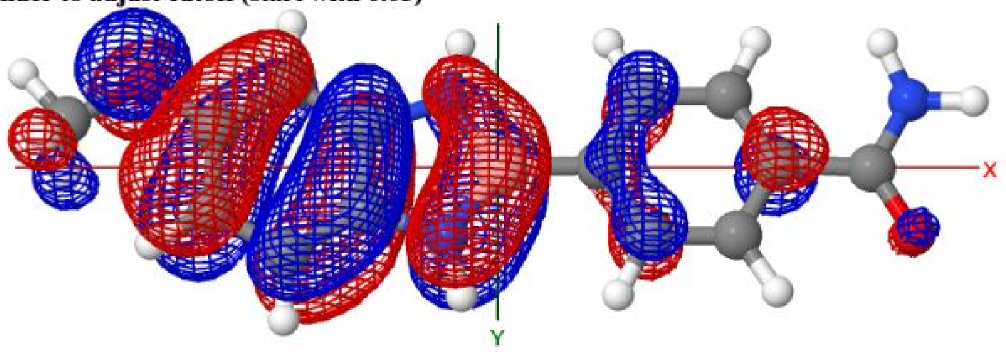

CA2

CA2

^{1}H NMR

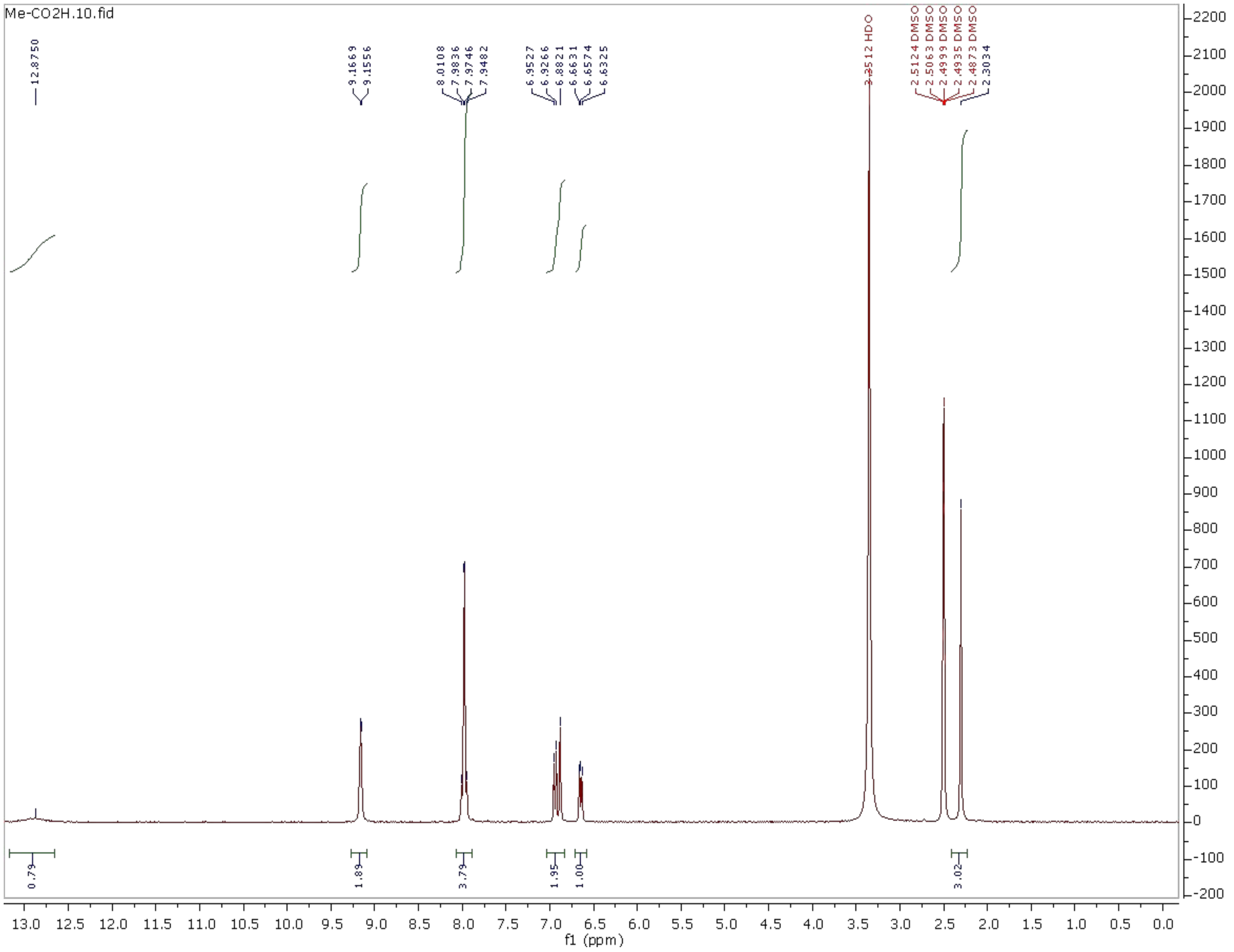

CA2

^{13}C NMR

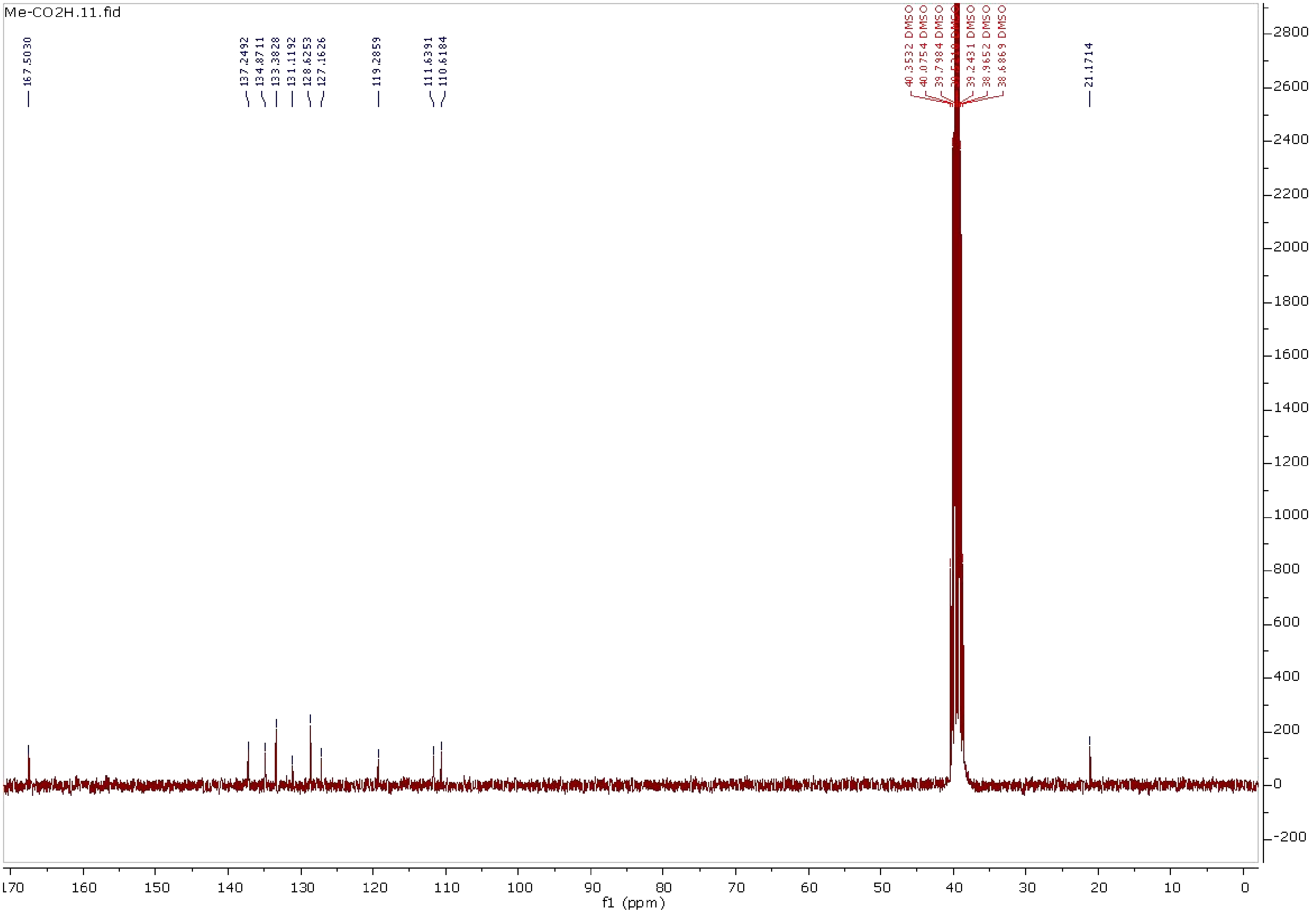
Me-CO2H.11.fid
167.5030
137.2492
134.8711
133.3828
131.1192
128.6253
127.1626
119.2859
111.6391
110.6184
40.3532 DMSO
40.0754 DMSO
39.7984 DMSO
39.2431 DMSO
38.9652 DMSO
38.6869 DMSO
21.1714
f1 (ppm)

CA2

IR

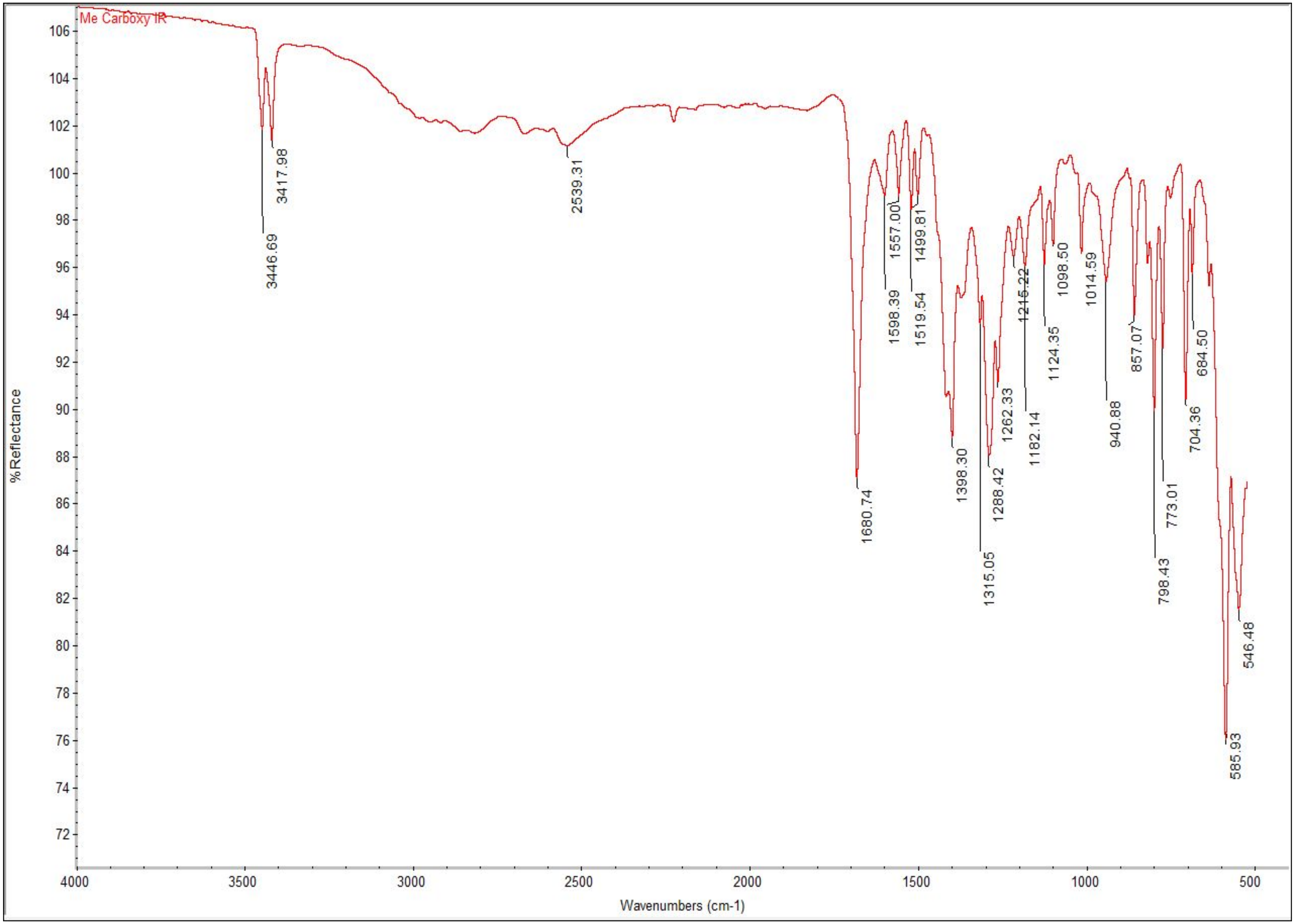

Me Carboxy IR
%Reflectance
Wavenumbers (cm-1)
3446.69
3417.98
2539.31
1680.74
1598.39
1557.00
1519.54
1499.81
1398.30
1315.05
1288.42
1262.33
1215.22
1182.14
1124.35
1098.50
1014.59
940.88
857.07
798.43
773.01
704.36
684.50
585.93
546.48

CA2

HRMS

C:\Xcalibur\...\Julius\Me carboxy 08/12/25 11:08:42

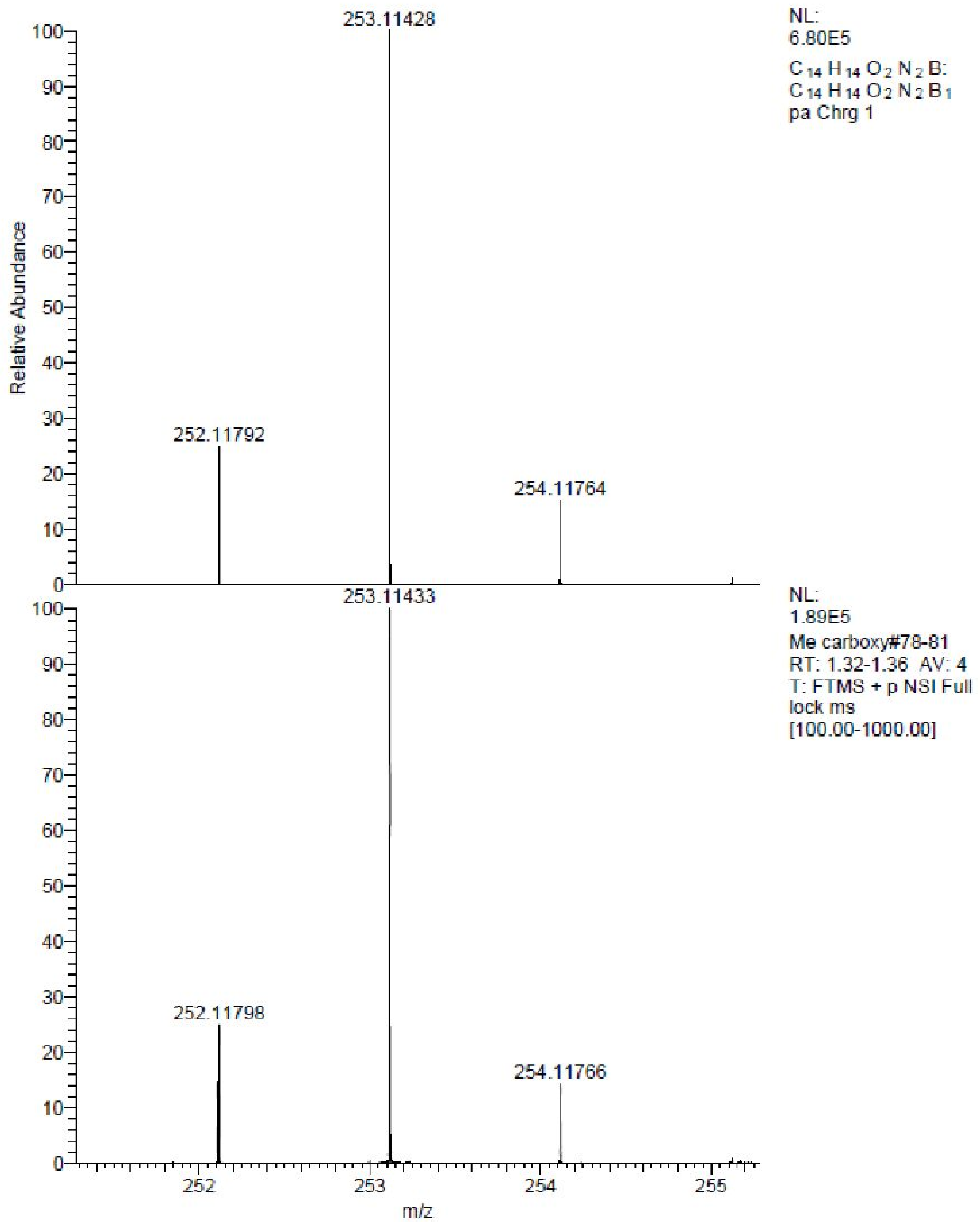

CA2

Computational Data

MO 67/76
Energy = -0.0577 a.u.
Symmetry = A
Use the slider to adjust cutoff (start with 0.05)

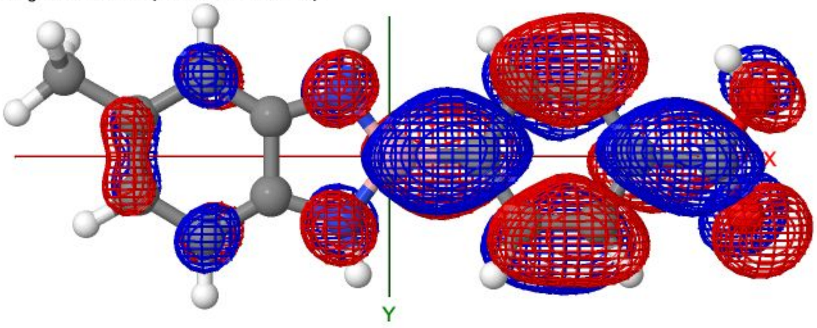

MO 66/76
Energy = -0.1988 a.u.
Symmetry = A
Use the slider to adjust cutoff (start with 0.05)

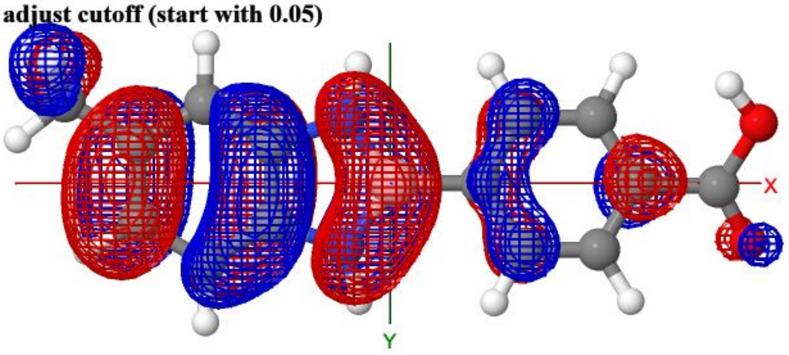

Am2

Am2

^{1}H NMR

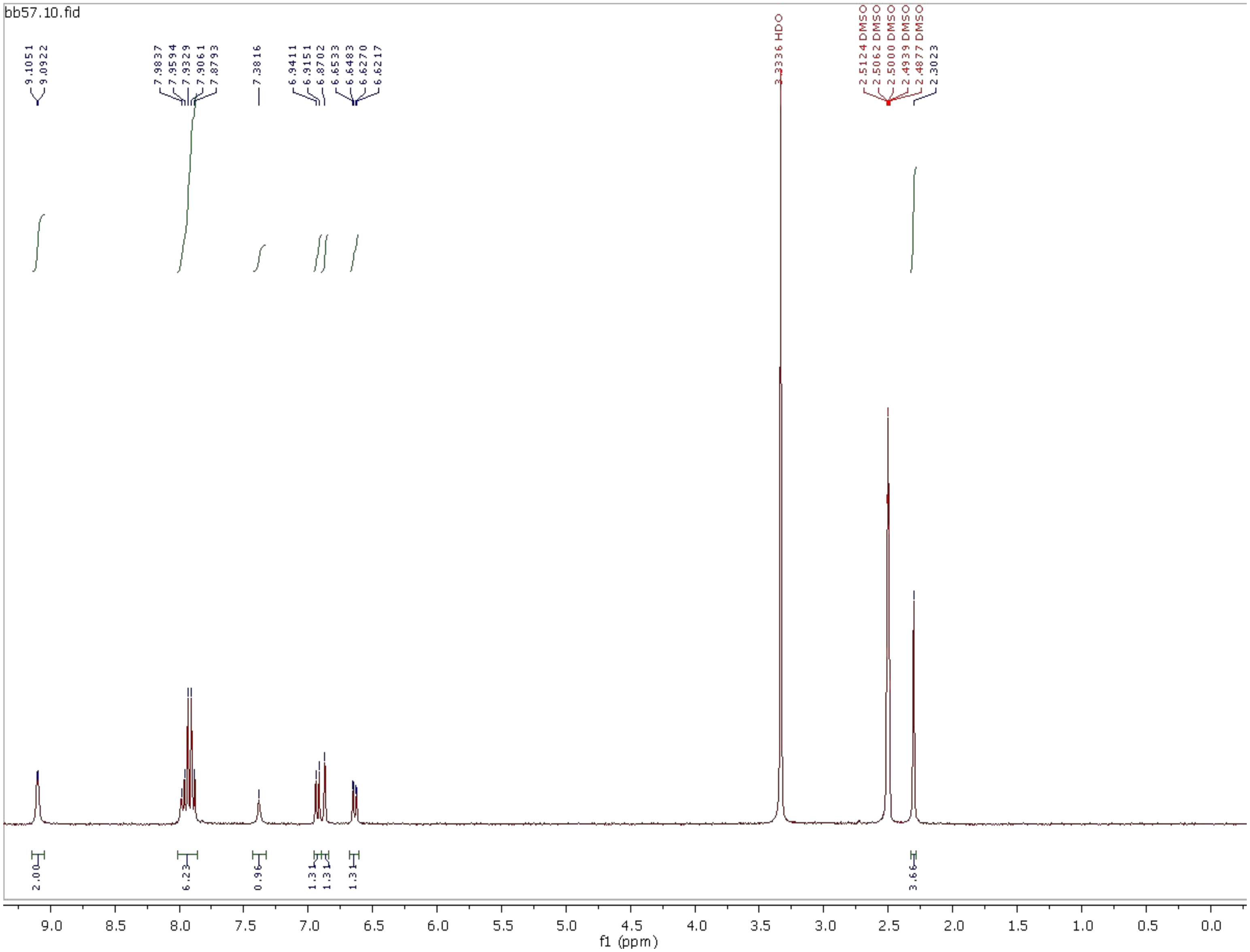

Am2

^{11}B NMR

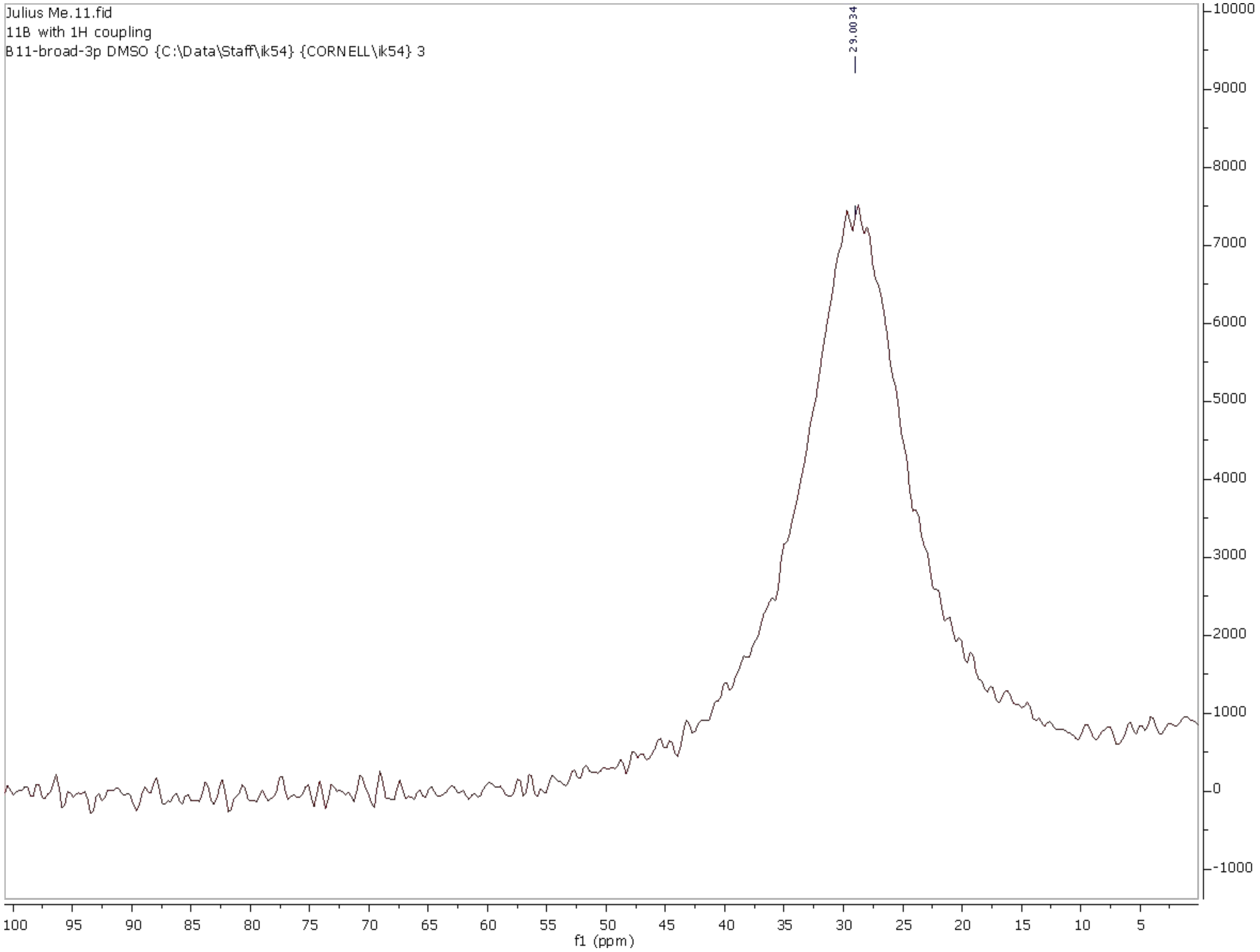
Julius Me.11.fid
11B with 1H coupling
B11-broad-3p DMSO {C:\Data\Staff\ik54} {CORNELL\ik54} 3
29.0034
10000
9000
8000
7000
6000
5000
4000
3000
2000
1000
0
-1000
100
95
90
85
80
75
70
65
60
55
50
45
40
35
30
25
20
15
10
5
f1 (ppm)

Am2

IR

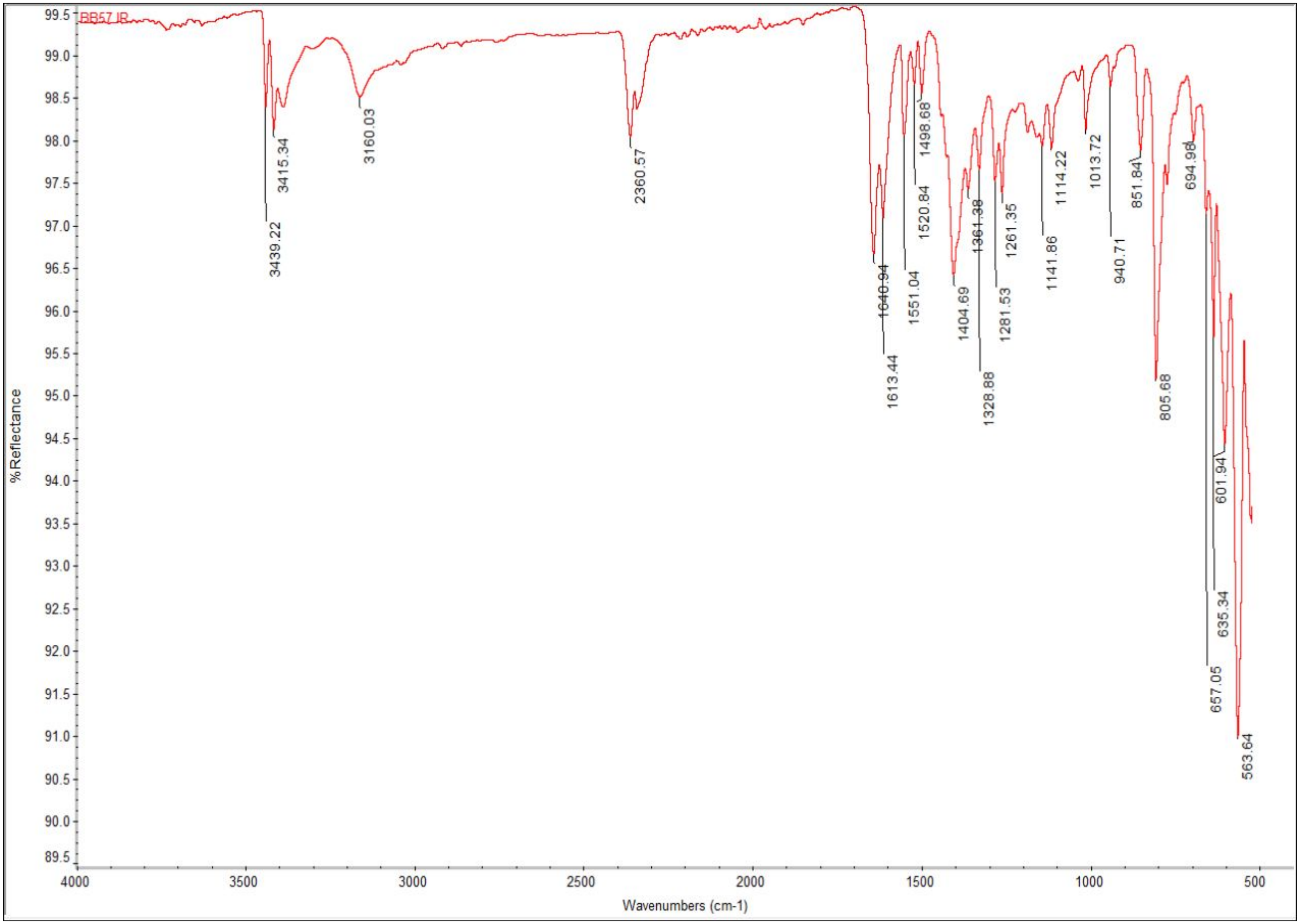

BB57 IR
% Reflectance
Wavenumbers (cm-1)
3439.22
3415.34
3160.03
2360.57
1640.94
1613.44
1551.04
1520.84
1498.68
1404.69
1361.38
1328.88
1281.53
1261.35
1141.86
1114.22
1013.72
940.71
851.84
805.68
694.98
657.05
635.34
601.94
563.64

Am2

UV-vis

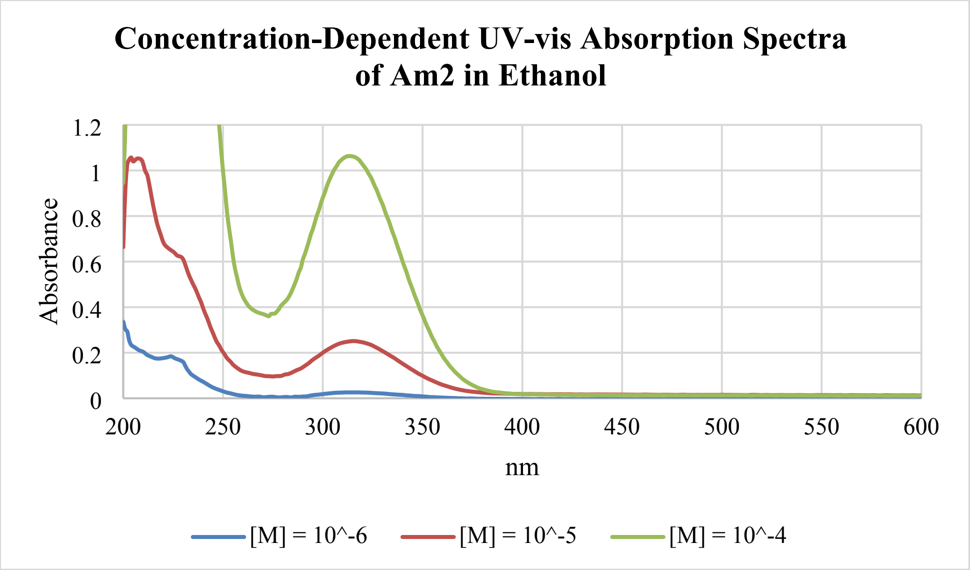

Concentration-Dependent UV-vis Absorption Spectra of Am2 in Ethanol
Absorbance
nm
[M] = 10^-6
[M] = 10^-5
[M] = 10^-4

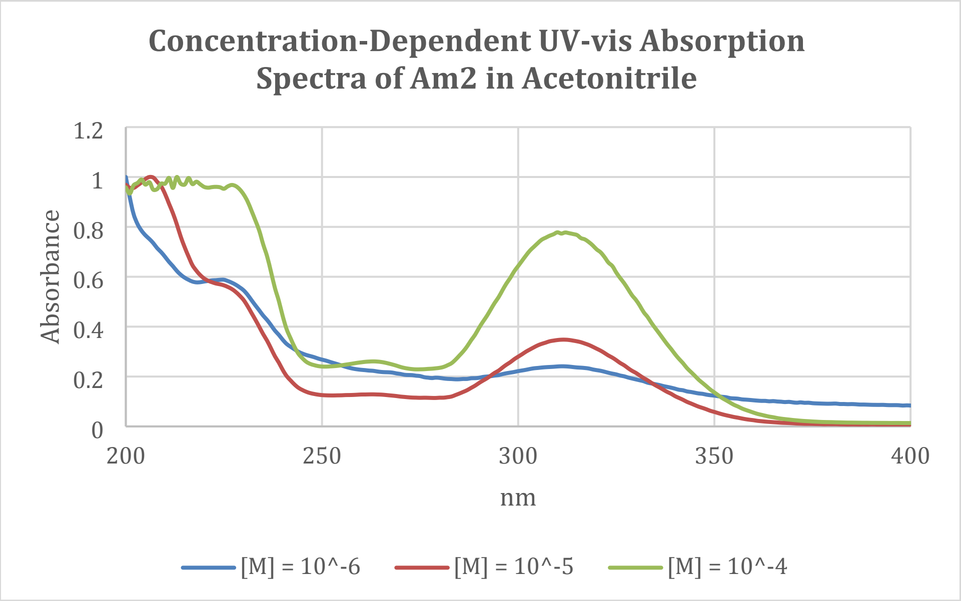

Concentration-Dependent UV-vis Absorption Spectra of Am2 in Acetonitrile
Absorbance
nm
[M] = 10^-6
[M] = 10^-5
[M] = 10^-4

Am2

Fluorescence Emission

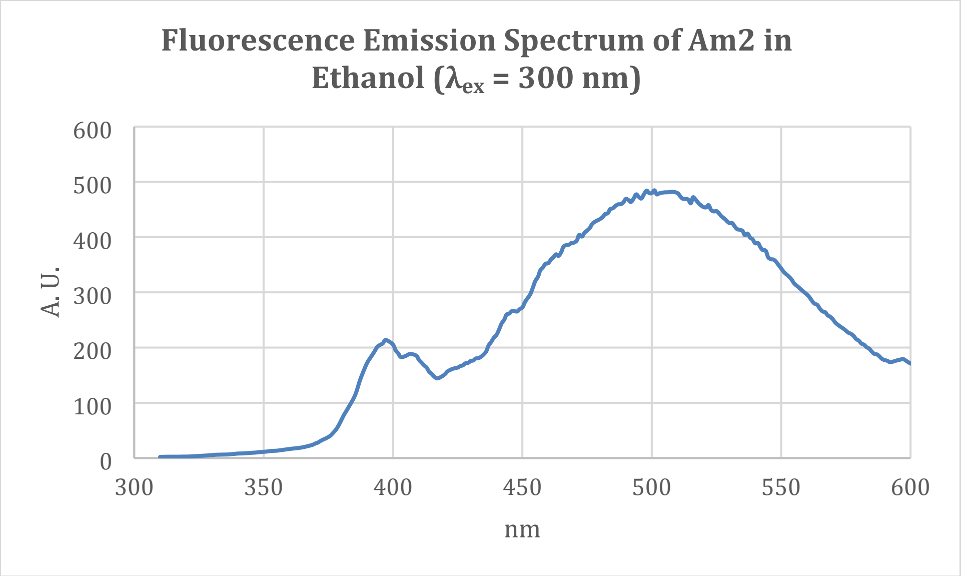

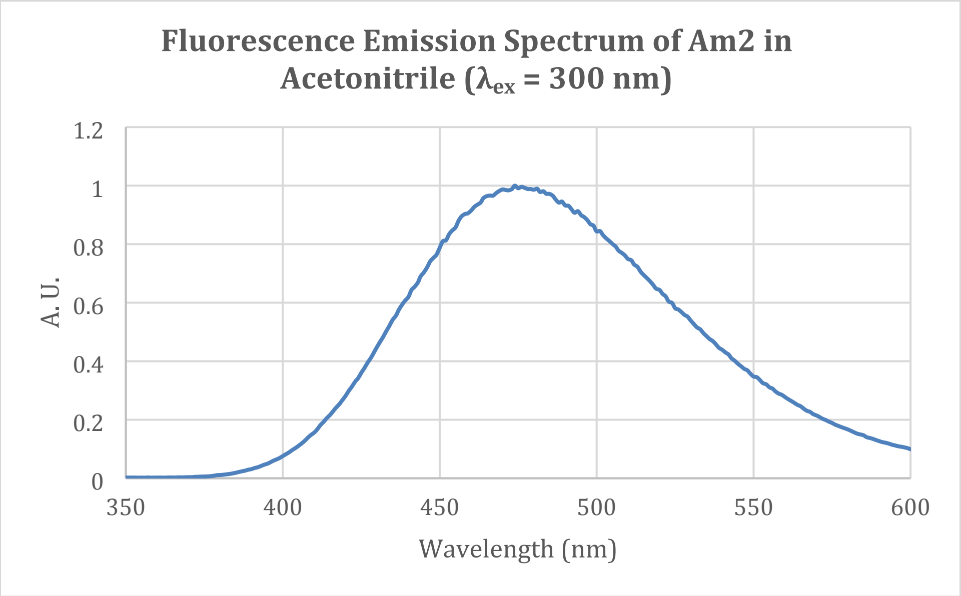

Am2

Fluorescence Excitation

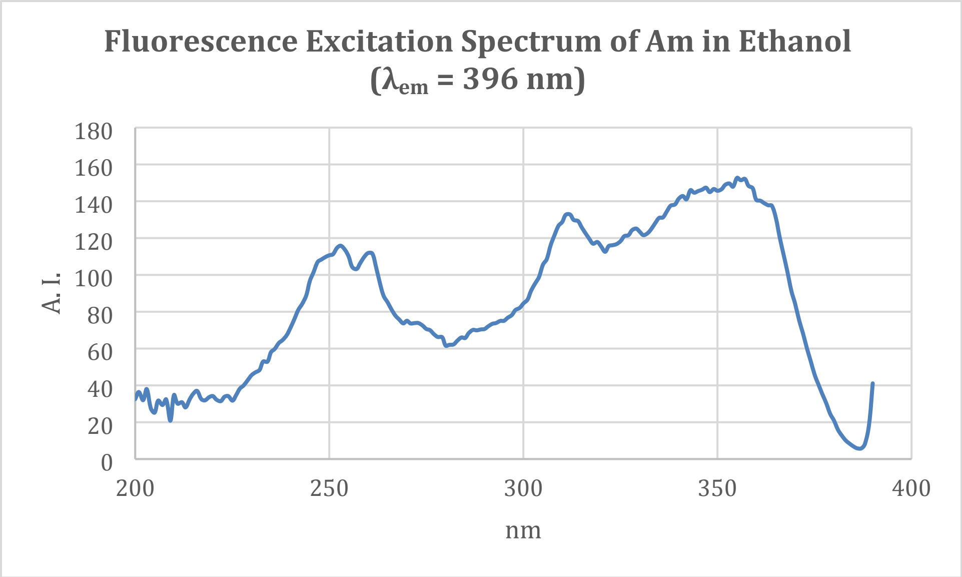

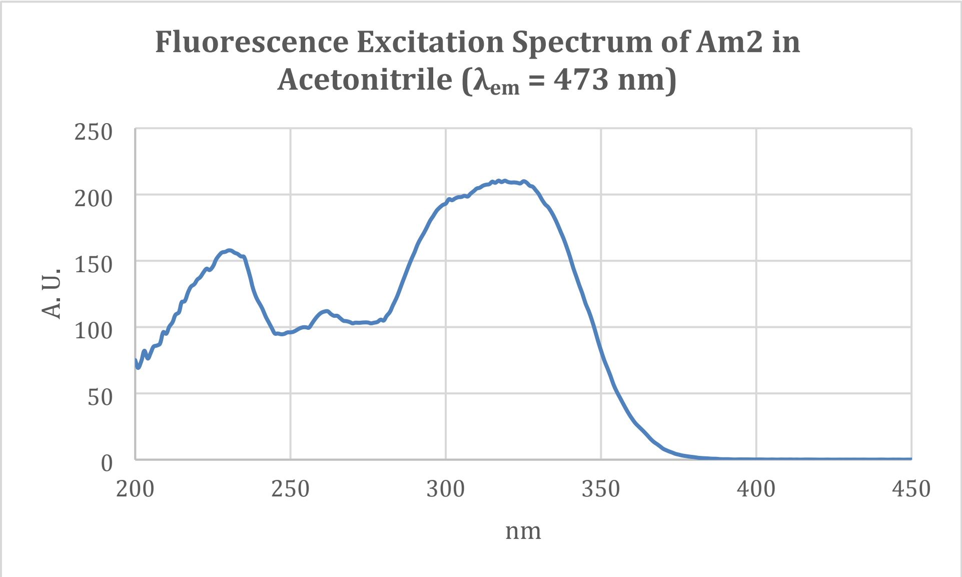

Am2

HRMS

C:\Xcalibur\...\Julian\me-amide 07/29/25 10:24:11

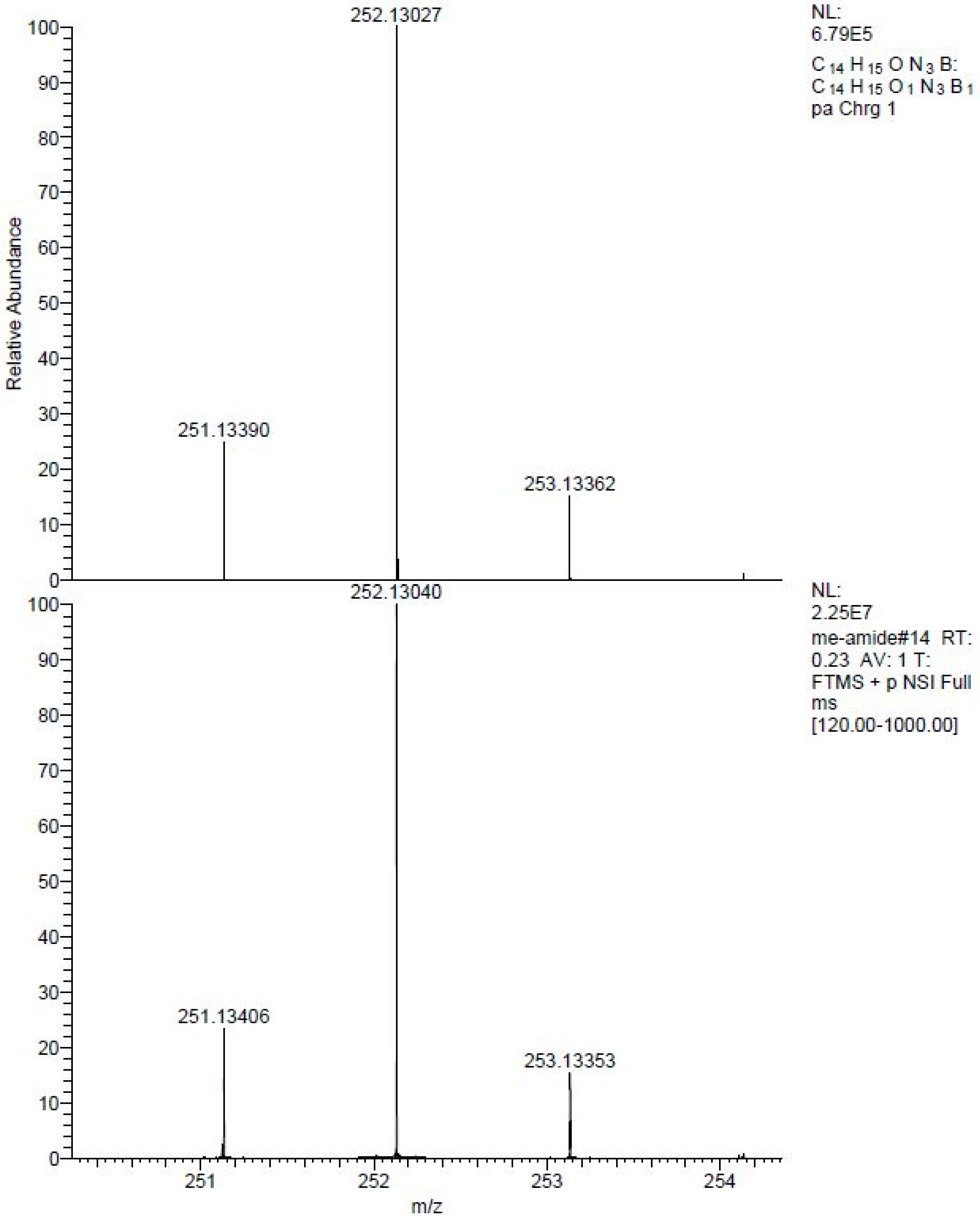

Am2

Computational Data

MO 67/76
Energy = -0.0454 a.u.
Symmetry = A''
Use the slider to adjust cutoff (start with 0.05)

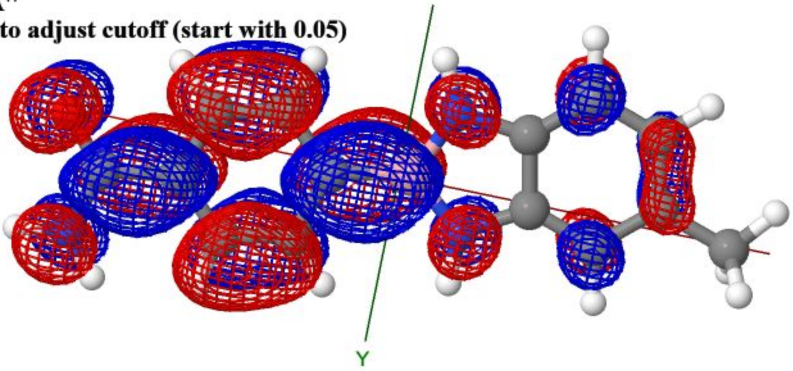

MO 66/76
Energy = -0.1927 a.u.
Symmetry = A''
Use the slider to adjust cutoff (start with 0.05)

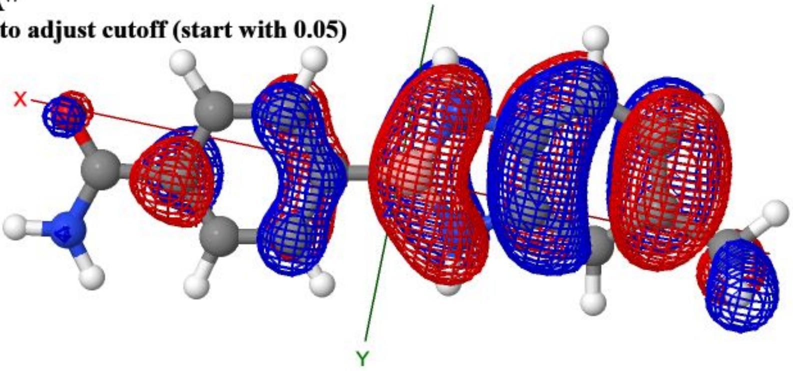

CA3

CA3

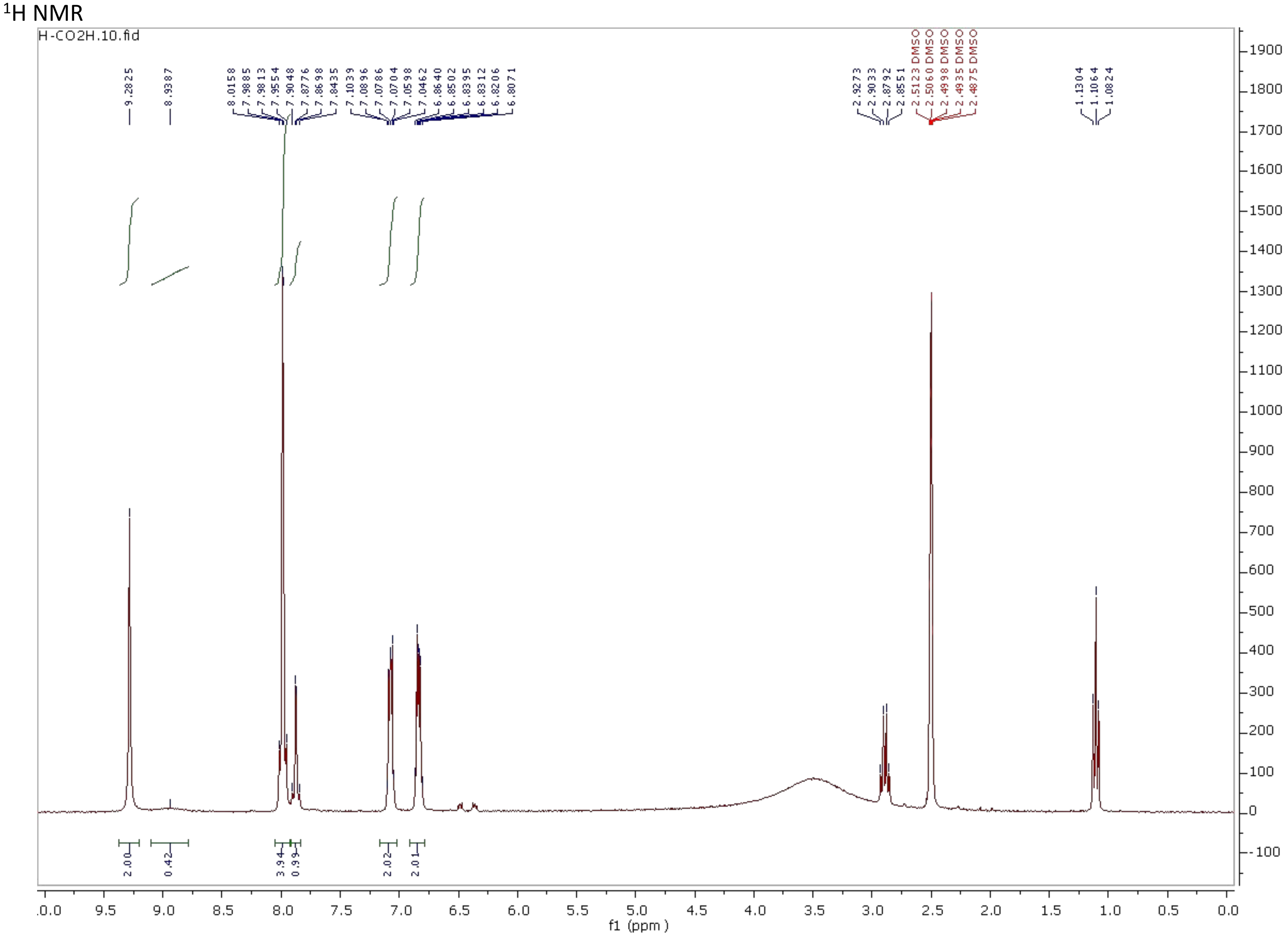

1H NMR
H-CO2H.10.fid
9.2825
8.9387
8.0158
7.9885
7.9813
7.9554
7.9048
7.8776
7.8698
7.8435
7.1039
7.0896
7.0786
7.0704
7.0598
7.0462
6.8640
6.8502
6.8395
6.8312
6.8206
6.8071
2.9273
2.9033
2.8792
2.8551
2.5123 DMSO
2.5060 DMSO
2.4998 DMSO
2.4935 DMSO
2.4875 DMSO
1.1304
1.1064
1.0824
2.00
0.42
3.94
0.99
2.02
2.01
f1 (ppm)

CA3

^{13}C NMR

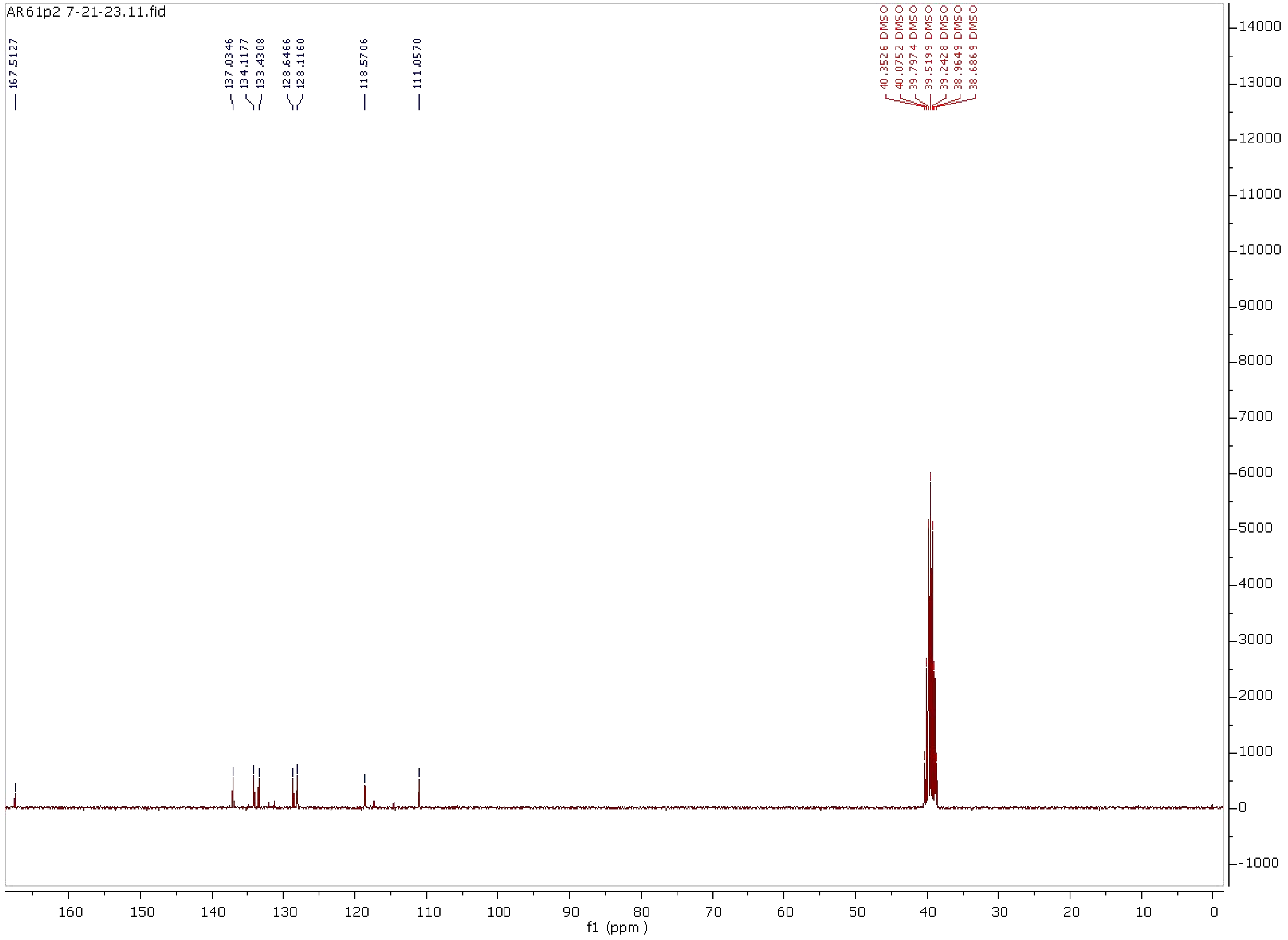
AR61p2 7-21-23.11.fid
167.5127
137.0346
134.1177
133.4308
128.6466
128.1160
118.5706
111.0570
40.3526 DMSO
40.0752 DMSO
39.7974 DMSO
39.5199 DMSO
39.2428 DMSO
38.9649 DMSO
38.6869 DMSO
f1 (ppm)

CA3

HSQC

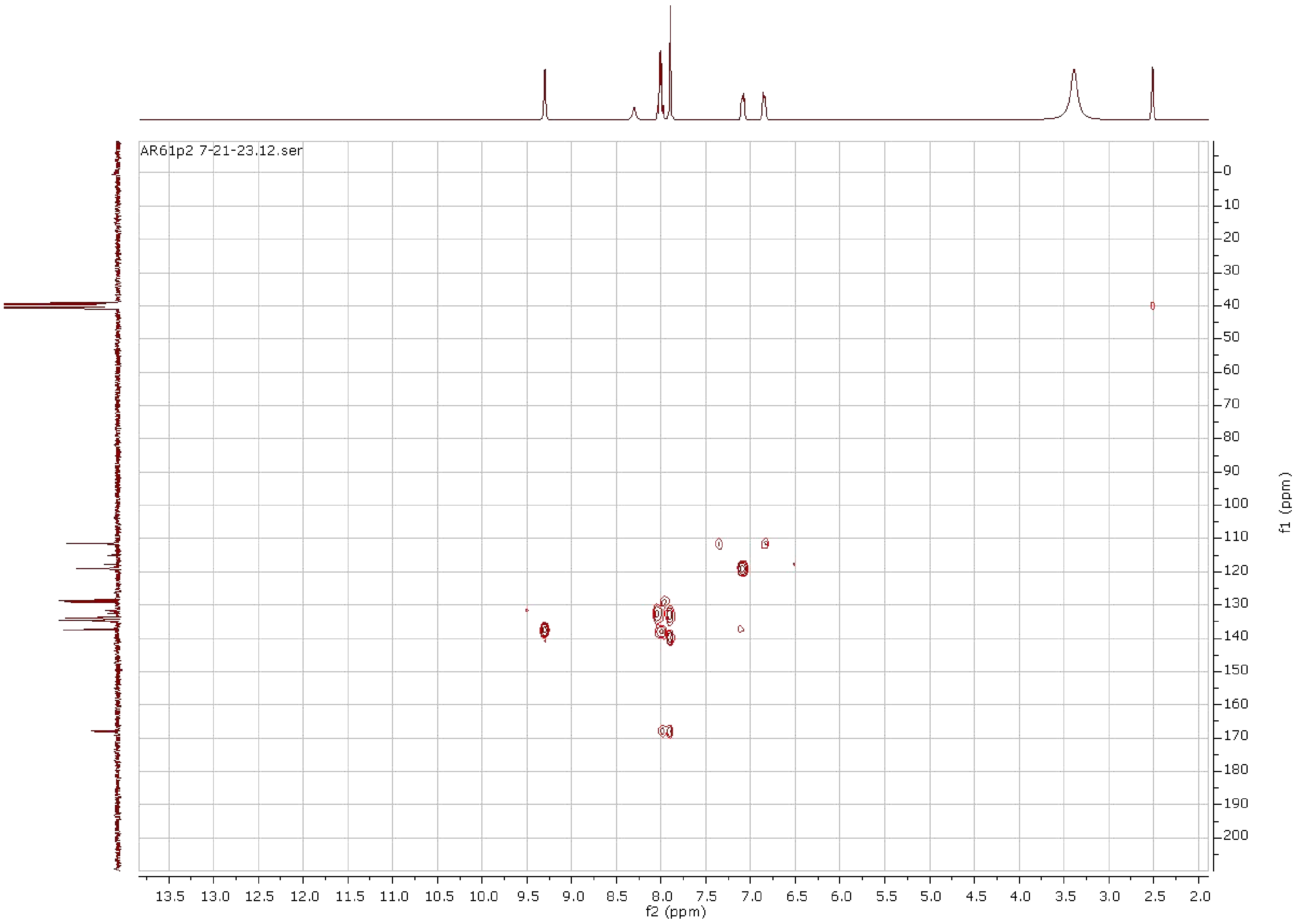
AR61p2 7-21-23.12.ser
13.5 13.0 12.5 12.0 11.5 11.0 10.5 10.0 9.5 9.0 8.5 8.0 7.5 7.0 6.5 6.0 5.5 5.0 4.5 4.0 3.5 3.0 2.5 2.0
f2 (ppm)
0 10 20 30 40 50 60 70 80 90 100 110 120 130 140 150 160 170 180 190 200
f1 (ppm)

CA3

HMBC

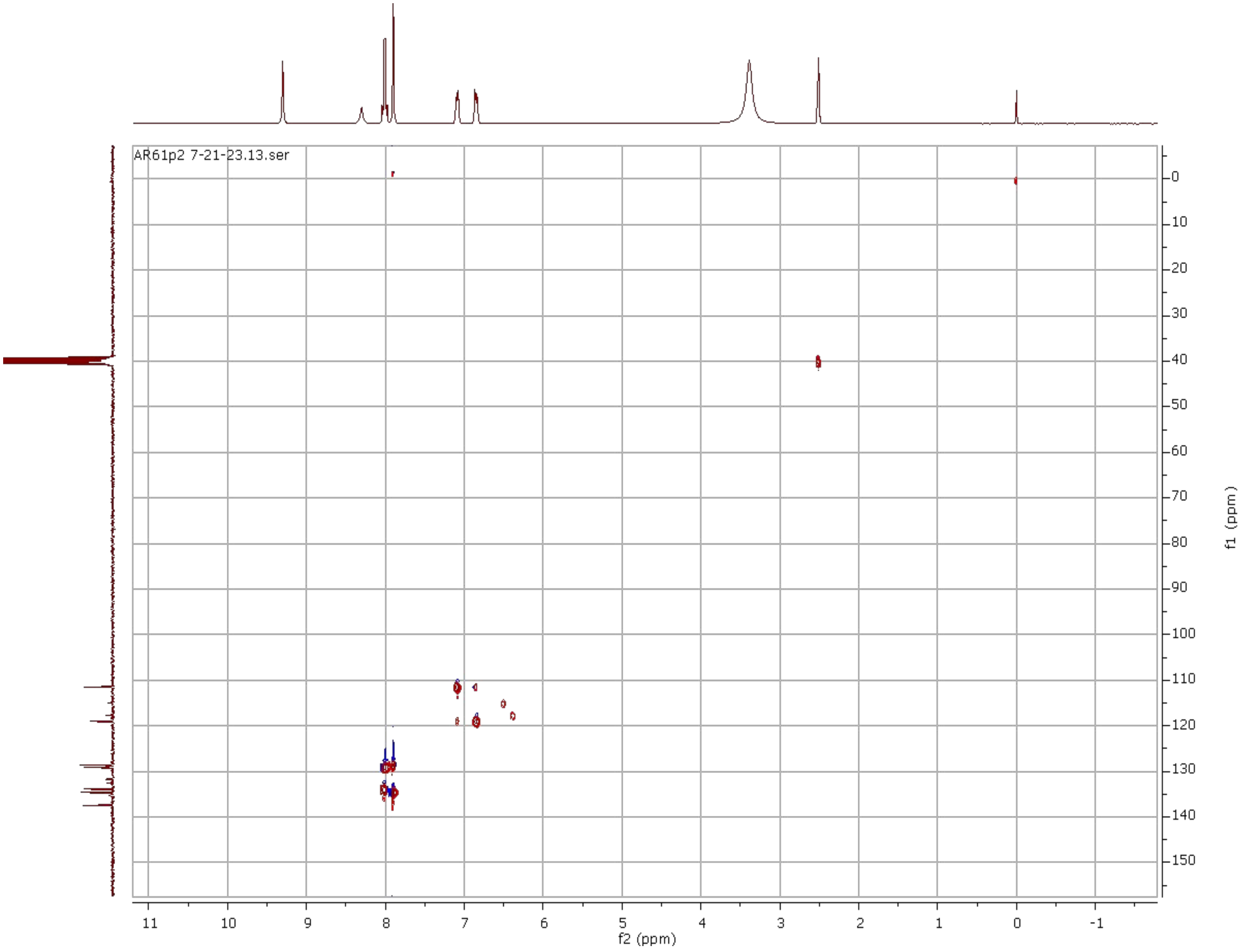
AR61p2 7-21-23.13.ser
f2 (ppm)
f1 (ppm)
11
10
9
8
7
6
5
4
3
2
1
0
-1
0
10
20
30
40
50
60
70
80
90
100
110
120
130
140
150

CA3

IR

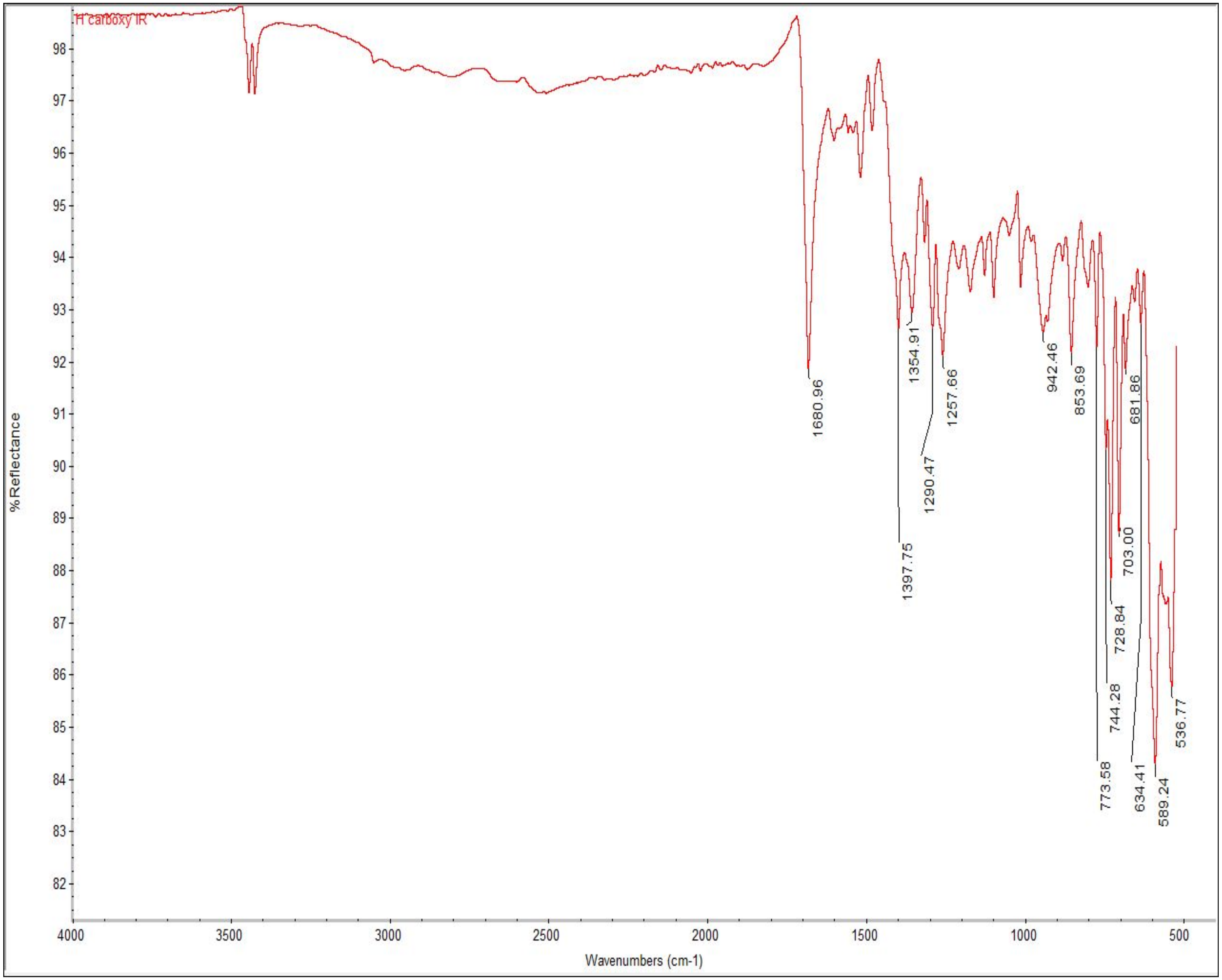
H carboxy IR
%Reflectance
98
97
96
95
94
93
92
91
90
89
88
87
86
85
84
83
82
1680.96
1354.91
1397.75
1290.47
1257.66
942.46
853.69
681.86
703.00
728.84
744.28
773.58
634.41
589.24
536.77
4000
3500
3000
2500
2000
1500
1000
500
Wavenumbers (cm-1)

CA3

UV-vis

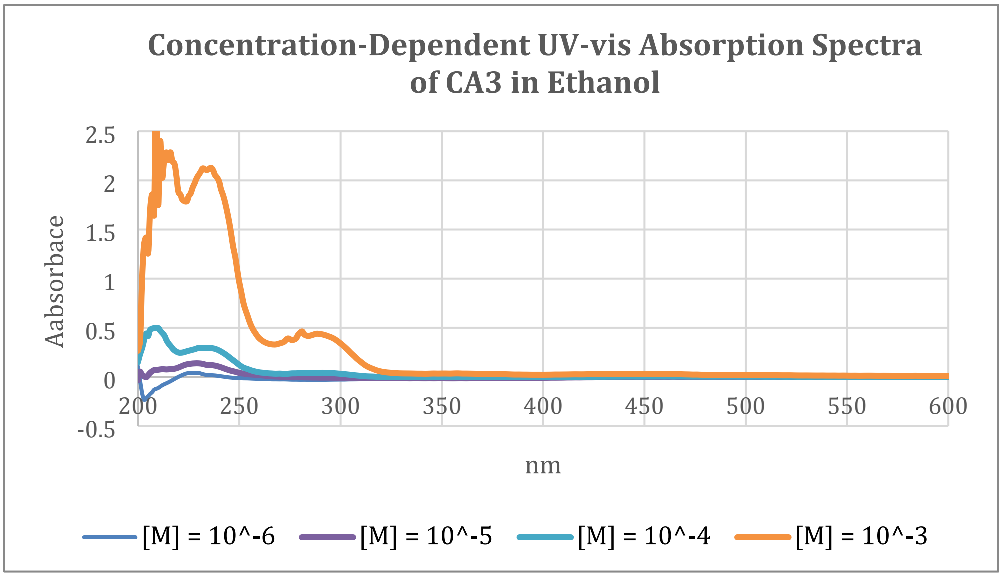

Concentration-Dependent UV-vis Absorption Spectra of CA3 in Ethanol
Aabsorbace
nm
2.5
2
1.5
1
0.5
0
-0.5
200
250
300
350
400
450
500
550
600
[M] = 10^-6
[M] = 10^-5
[M] = 10^-4
[M] = 10^-3

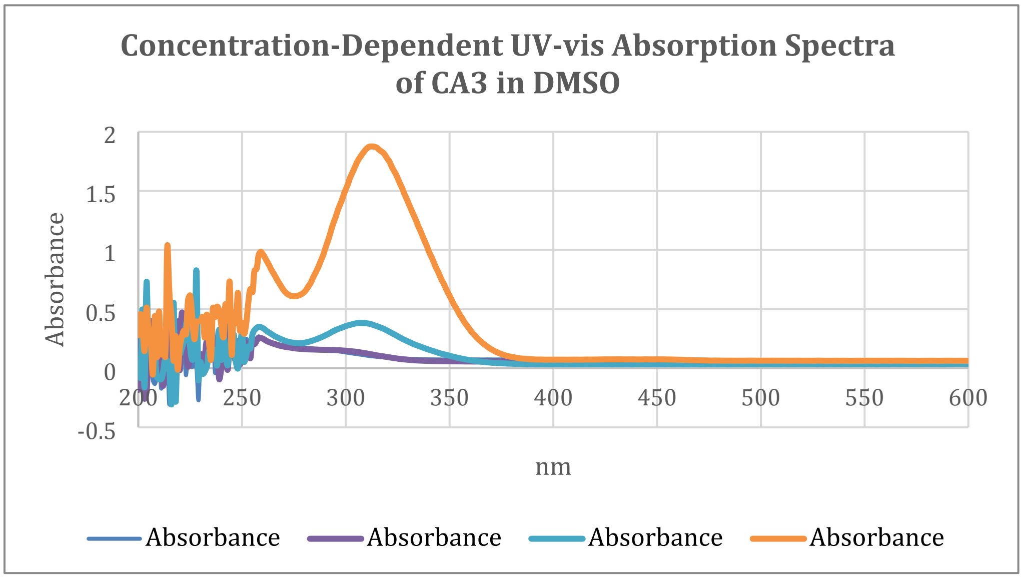

Concentration-Dependent UV-vis Absorption Spectra of CA3 in DMSO
Absorbance
nm
2
1.5
1
0.5
0
-0.5
200
250
300
350
400
450
500
550
600
Absorbance
Absorbance
Absorbance
Absorbance

CA3

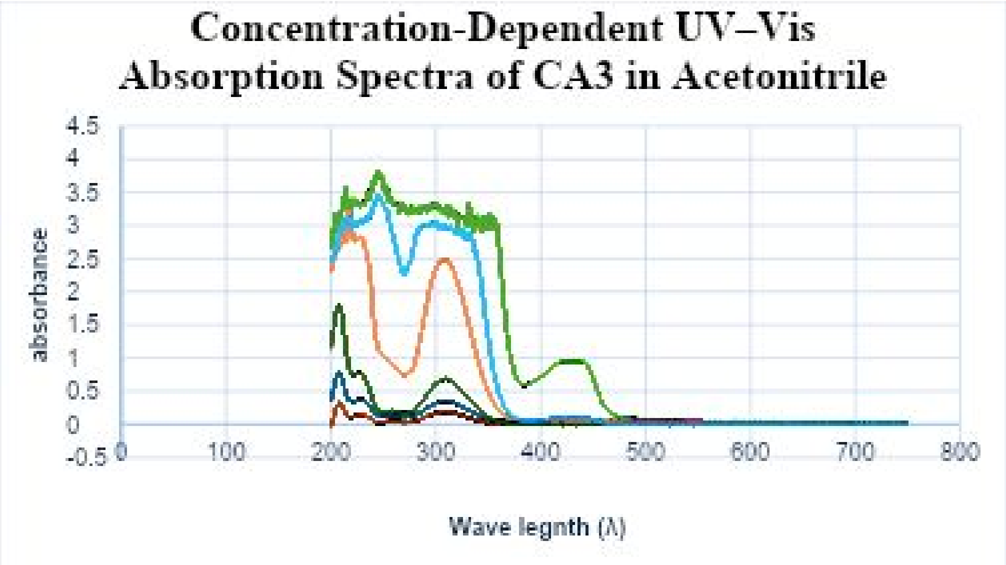
Concentration-Dependent UV–Vis Absorption Spectra of CA3 in Acetonitrile
absorbance
4.5
4
3.5
3
2.5
2
1.5
1
0.5
0
-0.5
0
100
200
300
400
500
600
700
800
Wave legnth (λ)

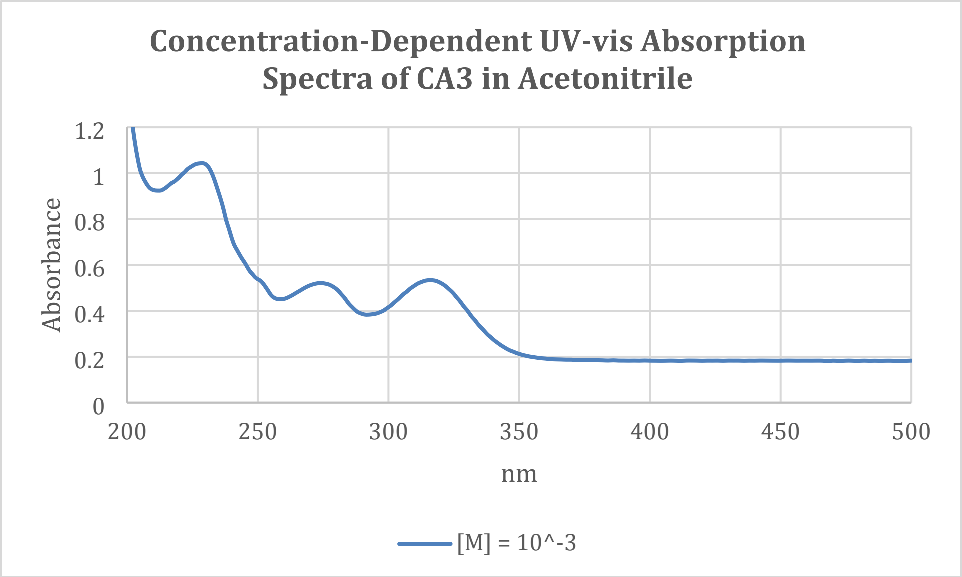
Concentration-Dependent UV-vis Absorption Spectra of CA3 in Acetonitrile
Absorbance
1.2
1
0.8
0.6
0.4
0.2
0
200
250
300
350
400
450
500
nm
[M] = 10^-3

CA3

Fluorescence Emission

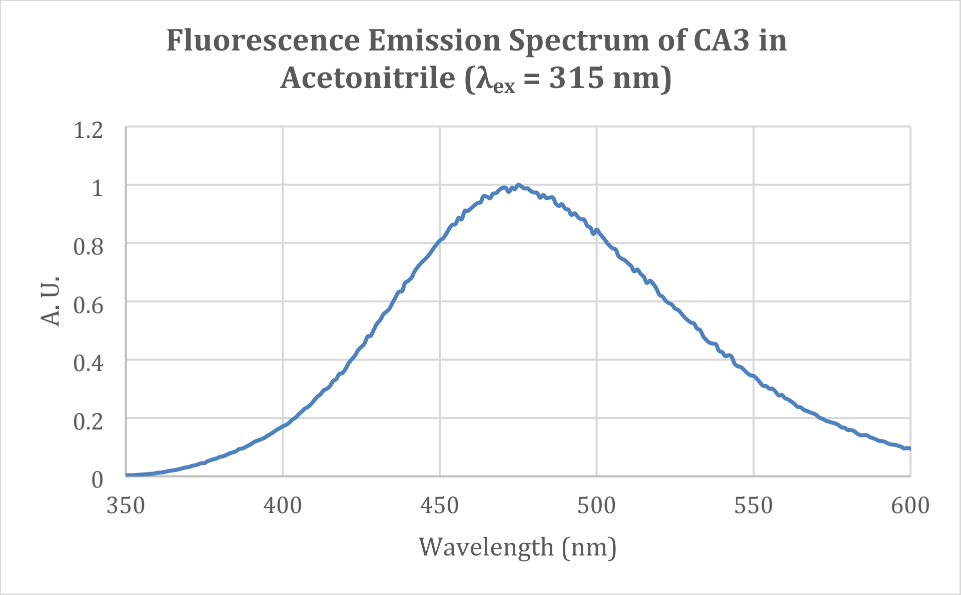

CA3

Computational Data

MO 63/72
Energy = -0.0585 a.u.
Symmetry = A
Use the slider to adjust cutoff (start with 0.05)

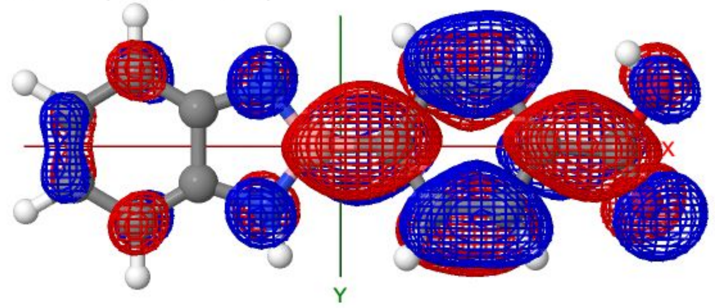

MO 62/72
Energy = -0.203 a.u.
Symmetry = A
Use the slider to adjust cutoff (start with 0.05)

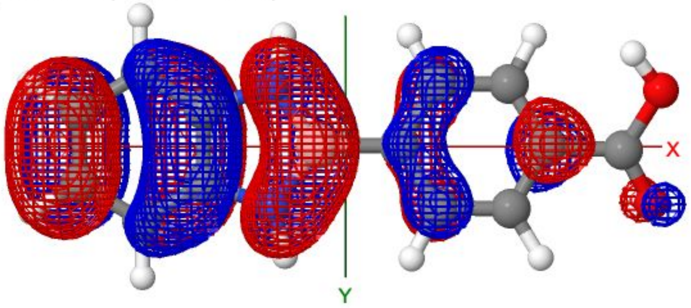

Am3

Am3

^{1}H NMR

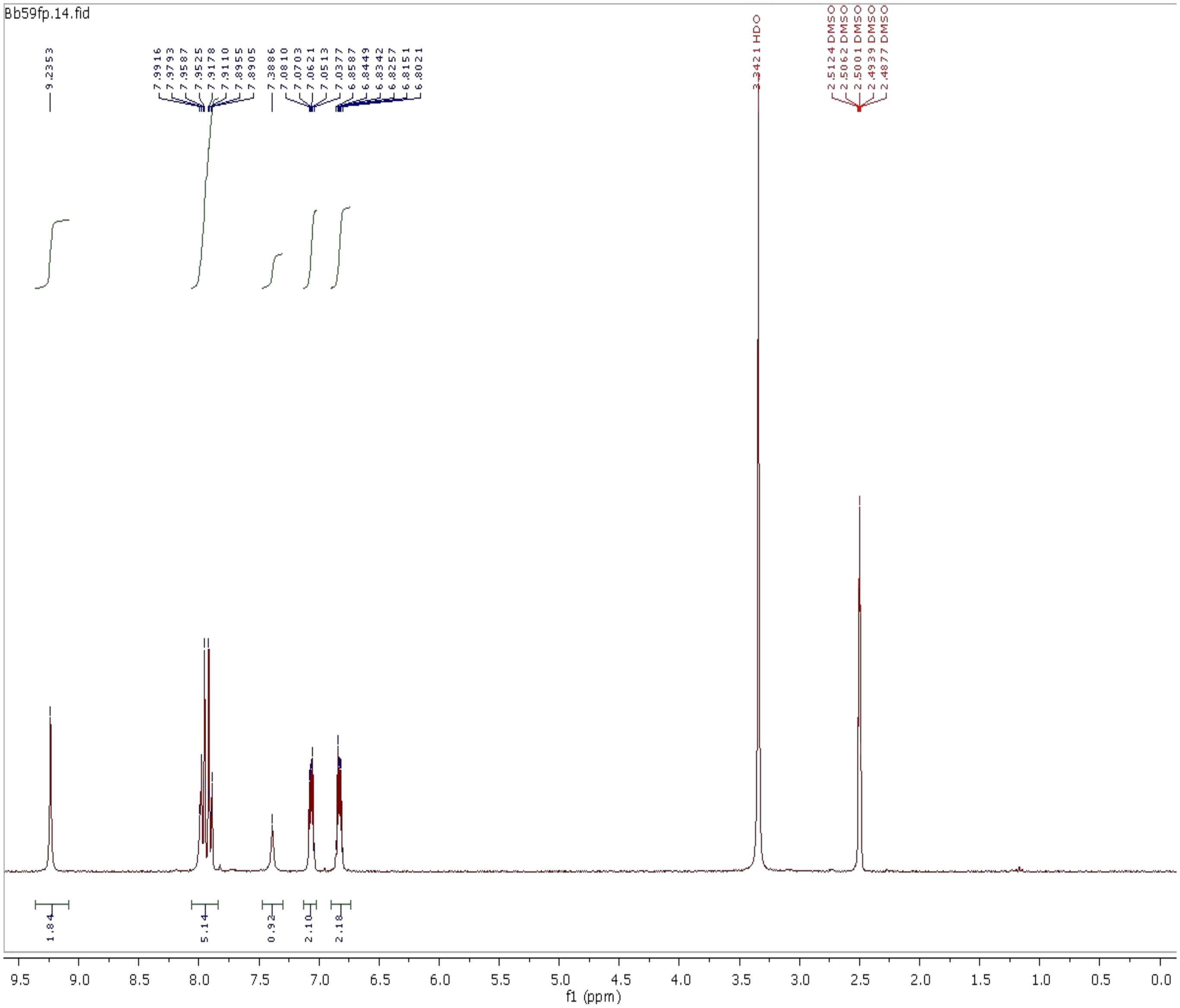

Am3

^{13}C NMR

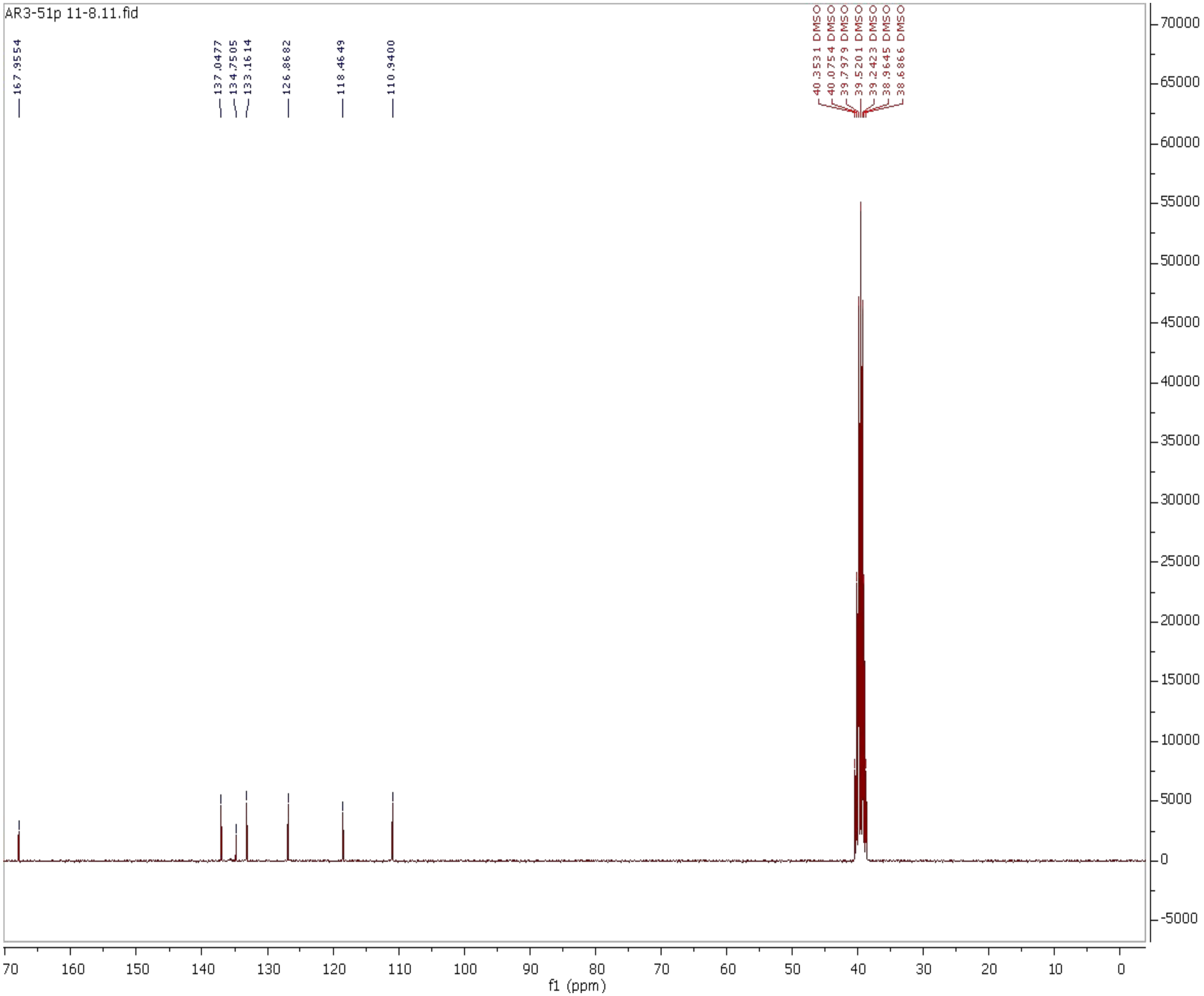
AR3-51p 11-8.11.fid
167.9554
137.0477
134.7505
133.1614
126.8682
118.4649
110.9400
40.3531 DMSO
40.0754 DMSO
39.7979 DMSO
39.5201 DMSO
39.2423 DMSO
38.9645 DMSO
38.6866 DMSO
f1 (ppm)

Am3

HSQC

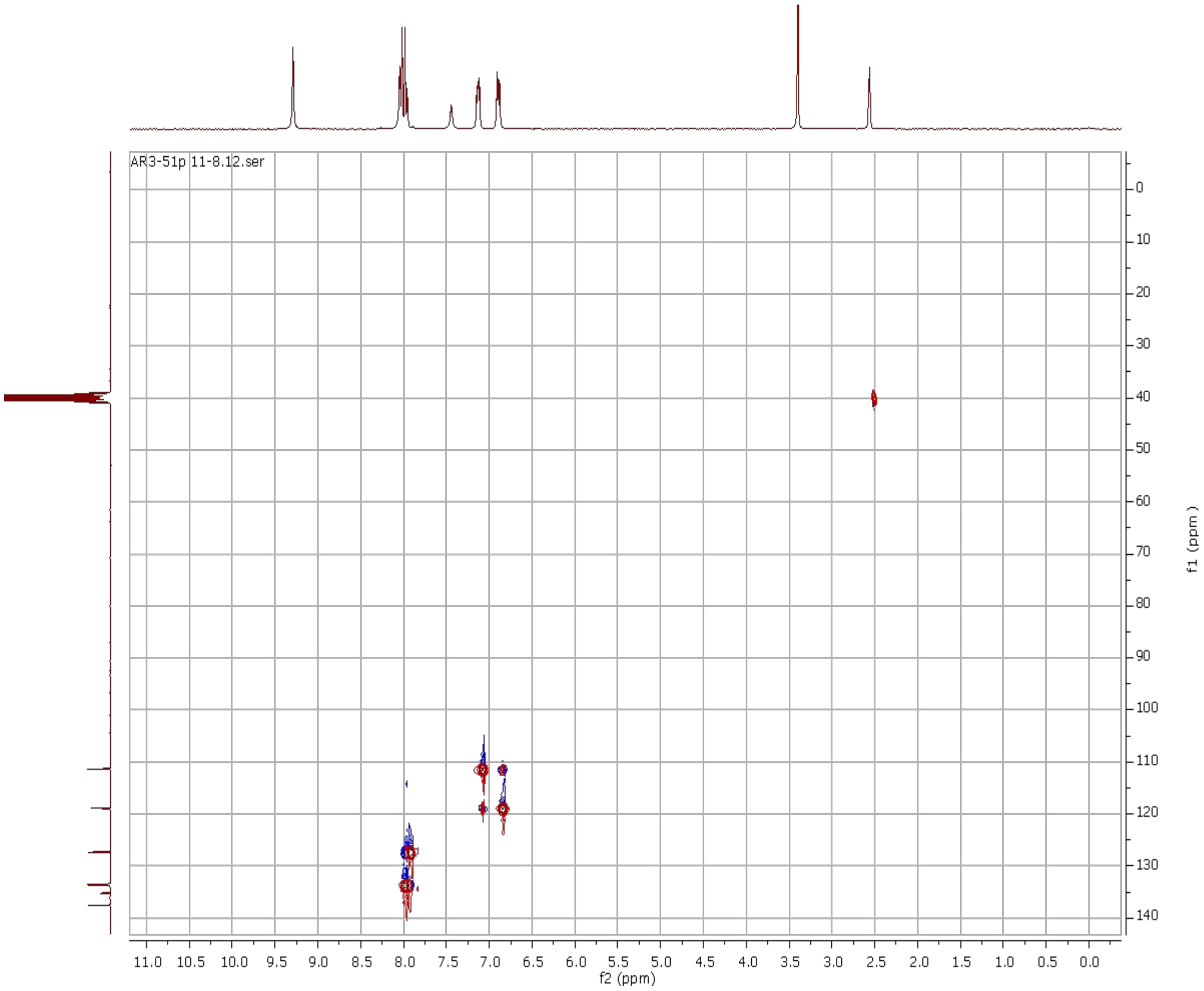

AR3-51p 11-8.12.ser
0
10
20
30
40
50
60
70
80
90
100
110
120
130
140
f1 (ppm)
11.0 10.5 10.0 9.5 9.0 8.5 8.0 7.5 7.0 6.5 6.0 5.5 5.0 4.5 4.0 3.5 3.0 2.5 2.0 1.5 1.0 0.5 0.0
f2 (ppm)

Am3

HMBC

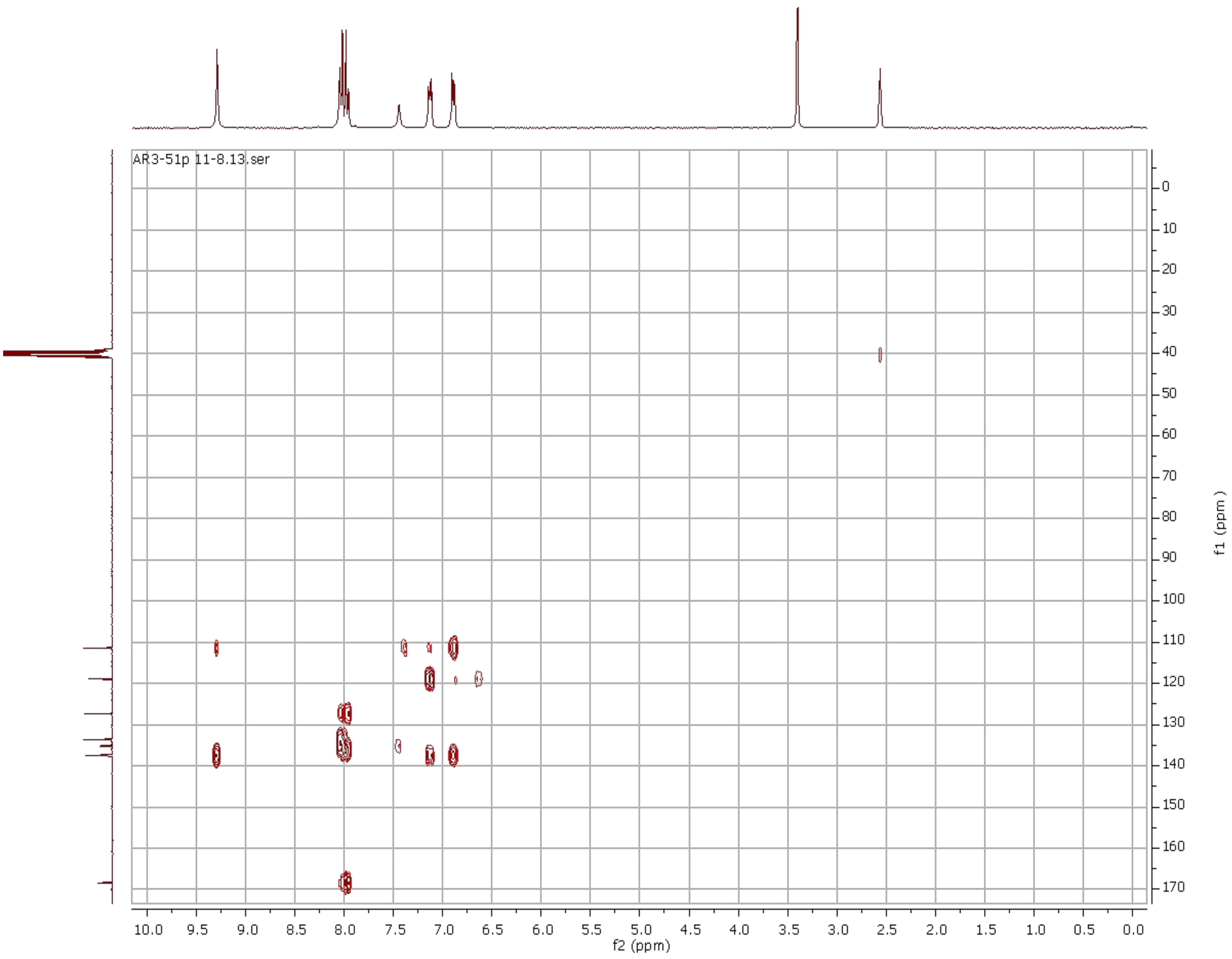

AR3-51p 11-8.13.ser
f1 (ppm)
0
10
20
30
40
50
60
70
80
90
100
110
120
130
140
150
160
170
f2 (ppm)
10.0
9.5
9.0
8.5
8.0
7.5
7.0
6.5
6.0
5.5
5.0
4.5
4.0
3.5
3.0
2.5
2.0
1.5
1.0
0.5
0.0

Am3

IR

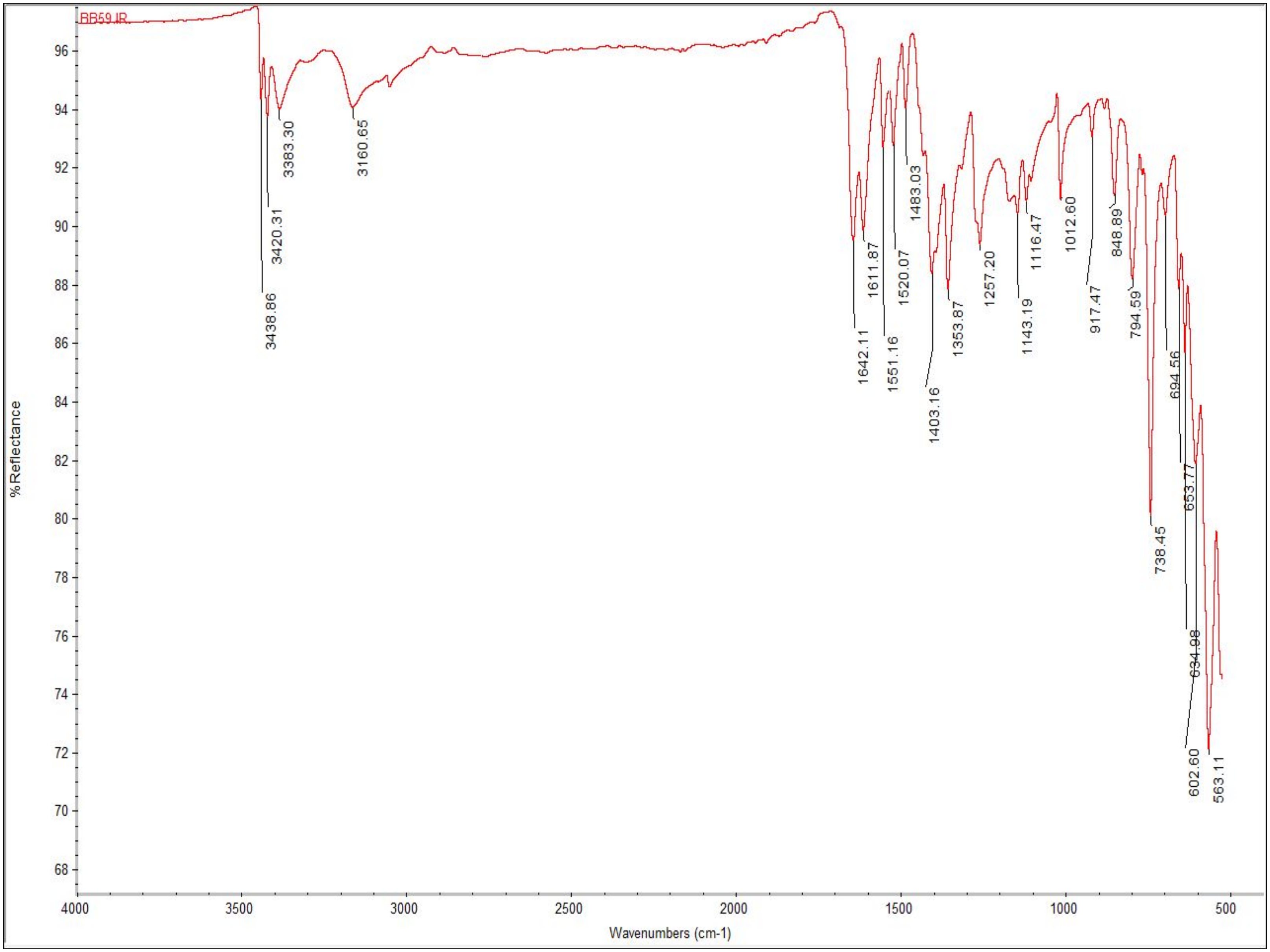

BB59.IR
%Reflectance
96
94
92
90
88
86
84
82
80
78
76
74
72
70
68
3438.86
3420.31
3383.30
3160.65
1642.11
1611.87
1551.16
1520.07
1483.03
1403.16
1353.87
1257.20
1143.19
1116.47
1012.60
917.47
848.89
794.59
738.45
694.56
653.77
634.98
602.60
563.11
4000
3500
3000
2500
2000
1500
1000
500
Wavenumbers (cm-1)

Am3

UV-vis

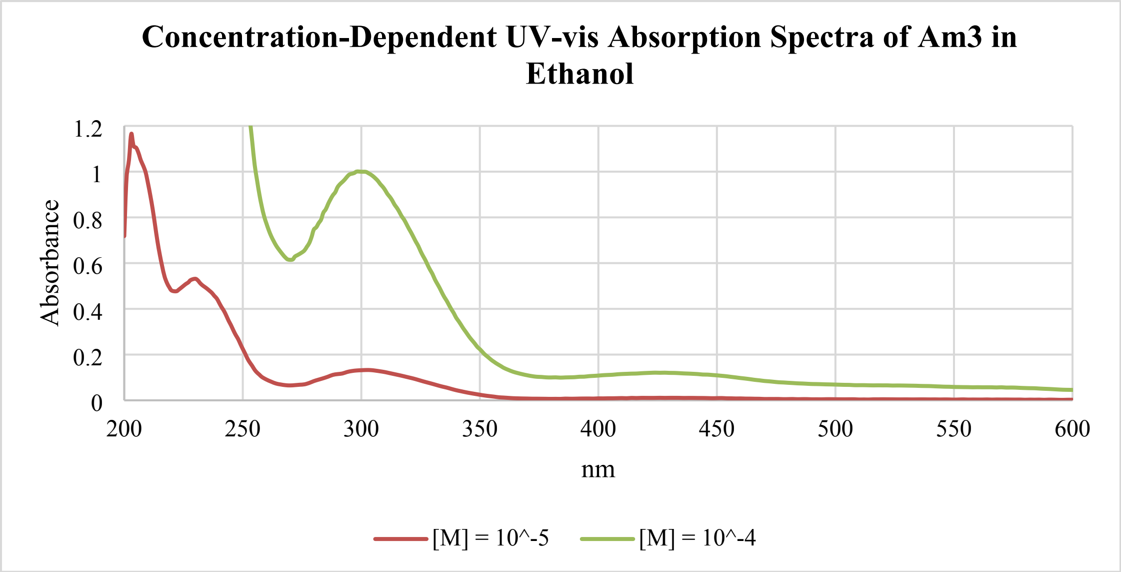
Concentration-Dependent UV-vis Absorption Spectra of Am3 in Ethanol
Absorbance
1.2
1
0.8
0.6
0.4
0.2
0
200
250
300
350
400
450
500
550
600
nm
[M] = 10^-5
[M] = 10^-4

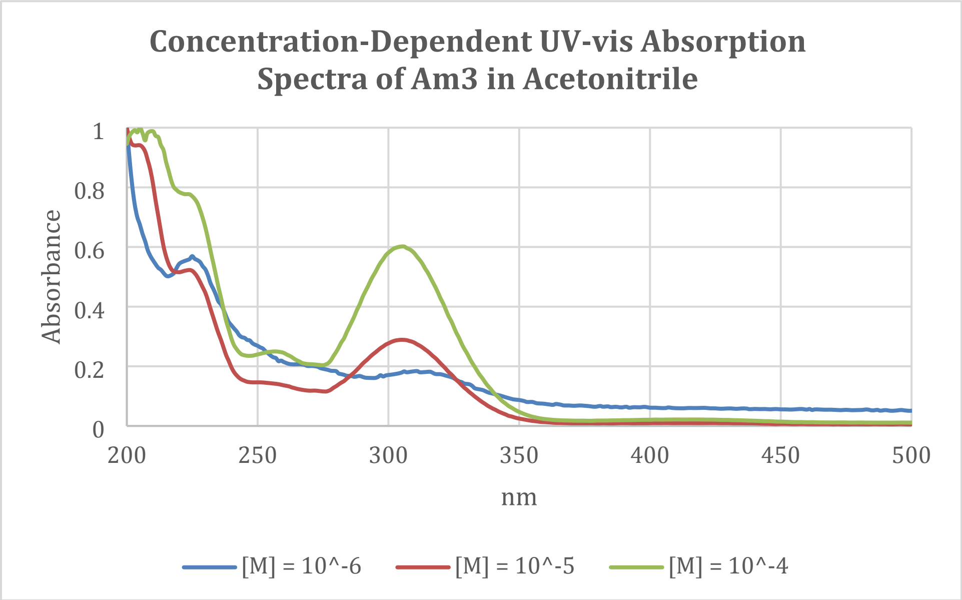
Concentration-Dependent UV-vis Absorption Spectra of Am3 in Acetonitrile
Absorbance
1
0.8
0.6
0.4
0.2
0
200
250
300
350
400
450
500
nm
[M] = 10^-6
[M] = 10^-5
[M] = 10^-4

Am3

Fluorescence Emission

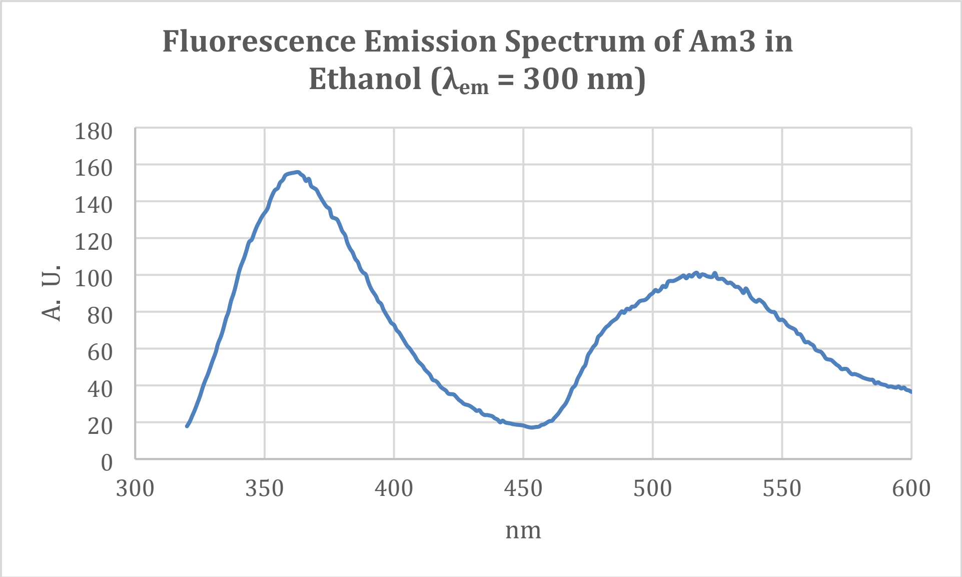

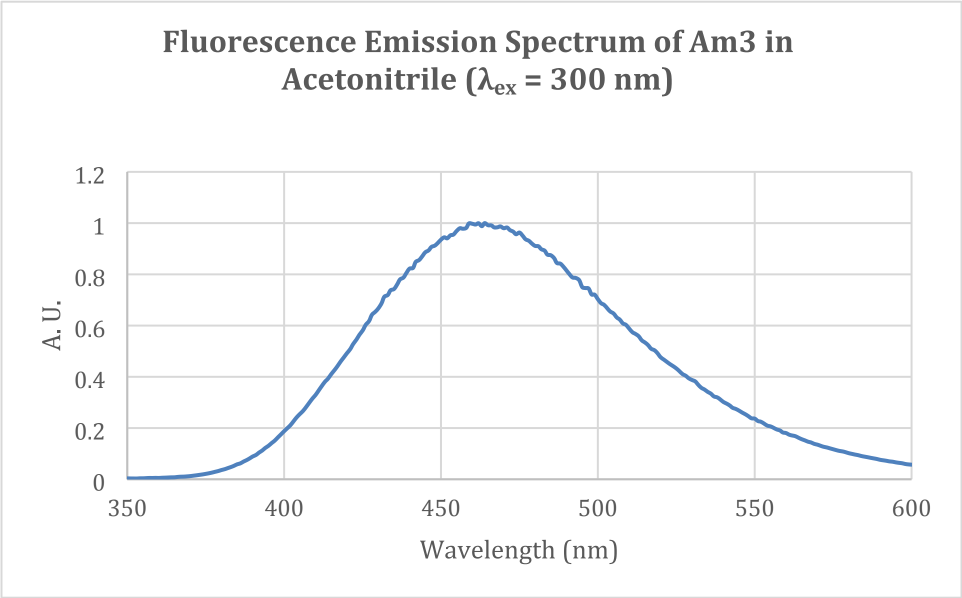

Am3

Fluorescence Excitation

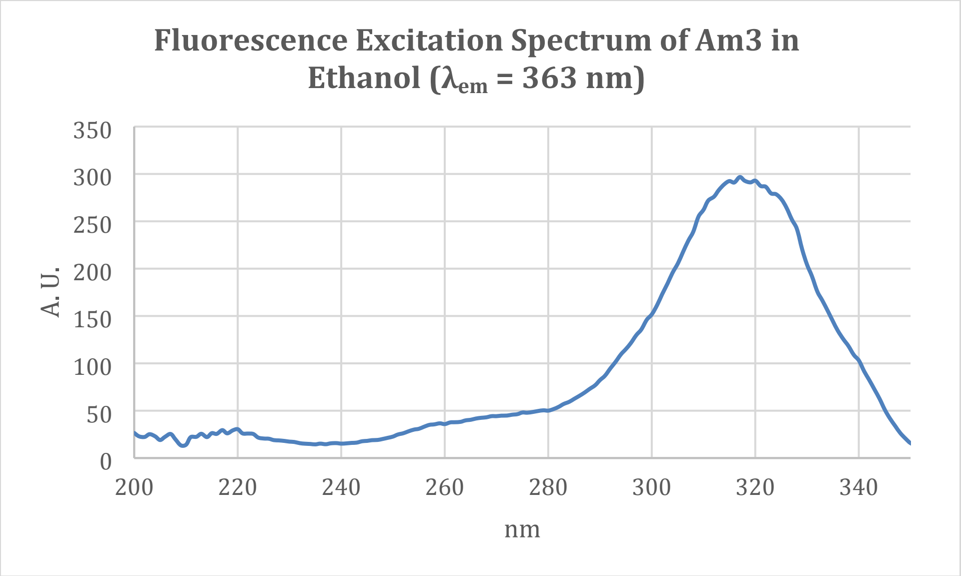

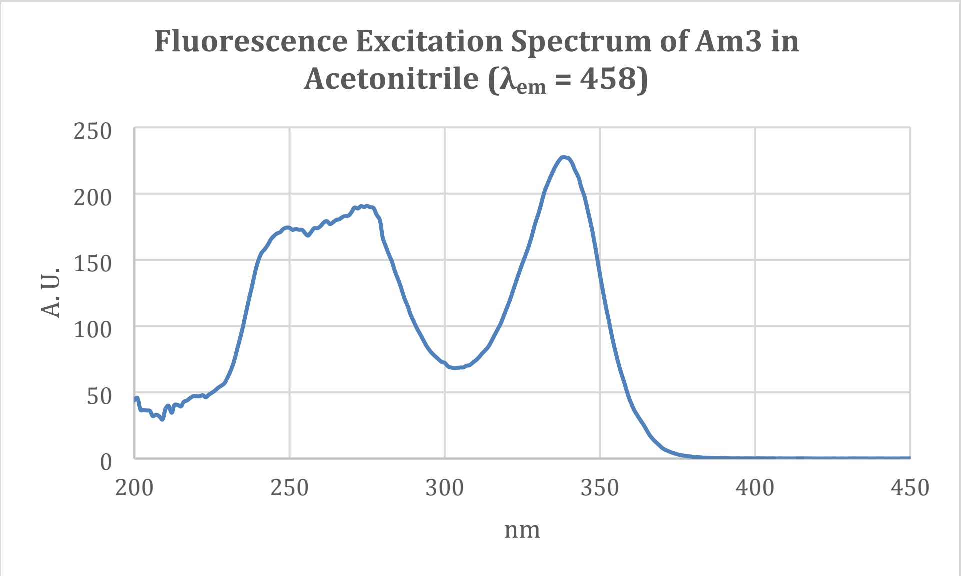

Am3

HRMS

C:\Xcalibur\...\Julian\AR3-51k 07/29/25 10:22:12

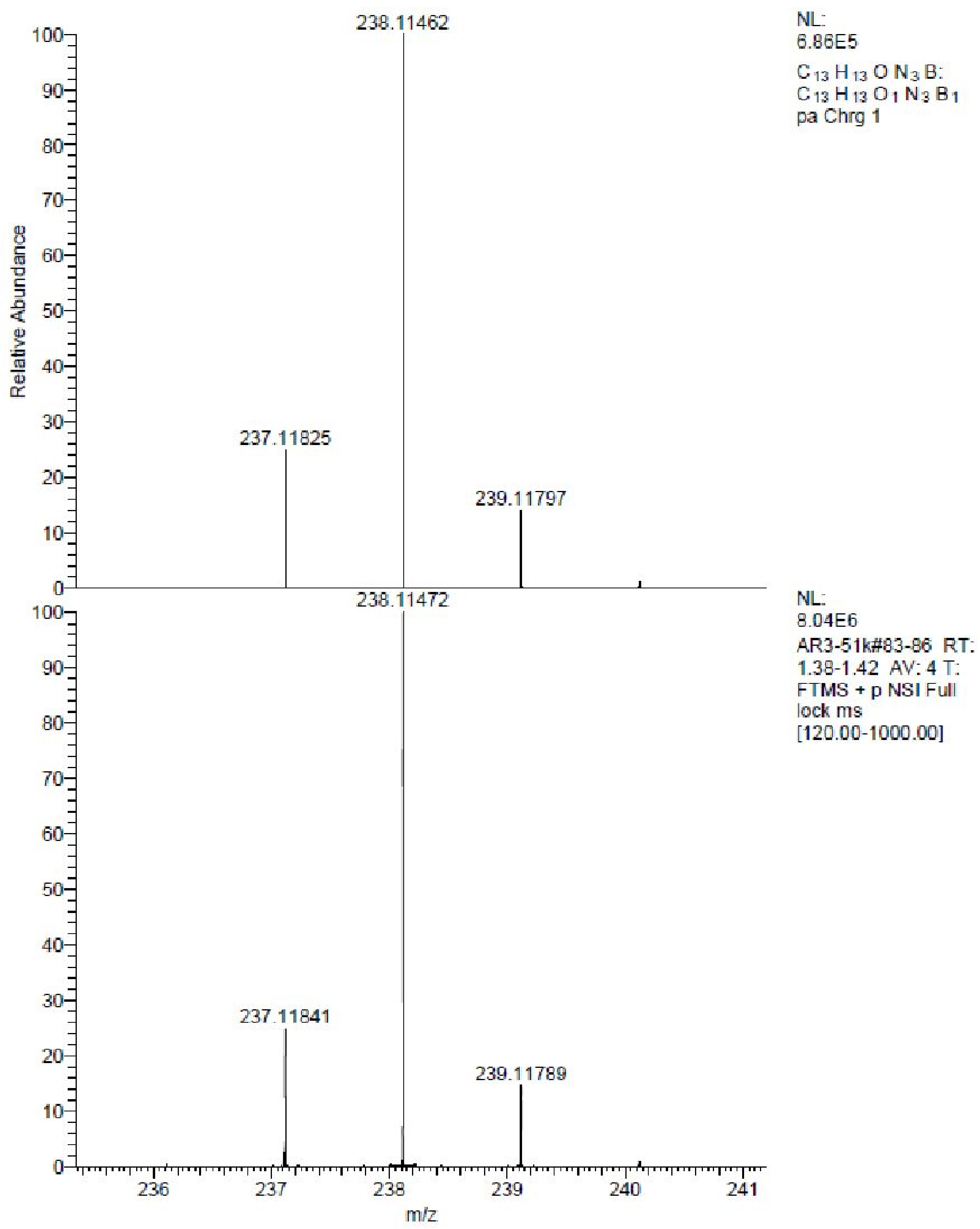

Am3

Computational Data

MO 63/72
Energy = -0.0473 a.u.
Symmetry = A
Use the slider to adjust cutoff (start with 0.05)

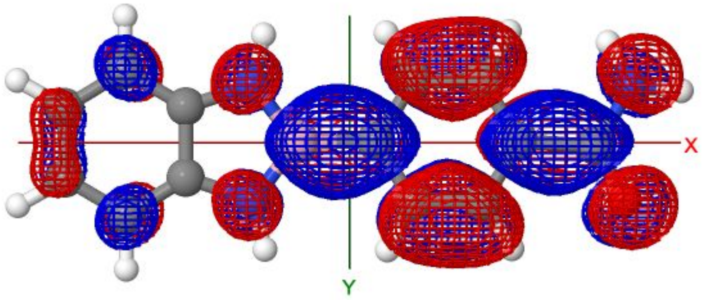

MO 62/72
Energy = -0.1969 a.u.
Symmetry = A
Use the slider to adjust cutoff (start with 0.05)

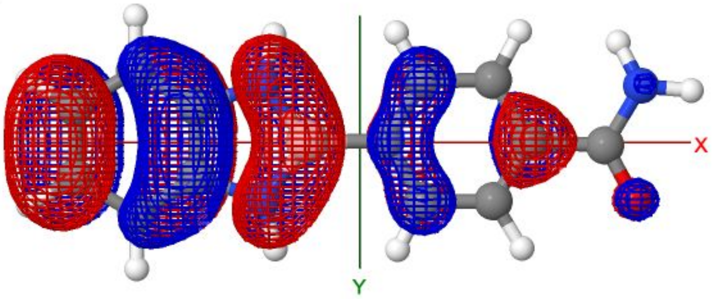

CA4

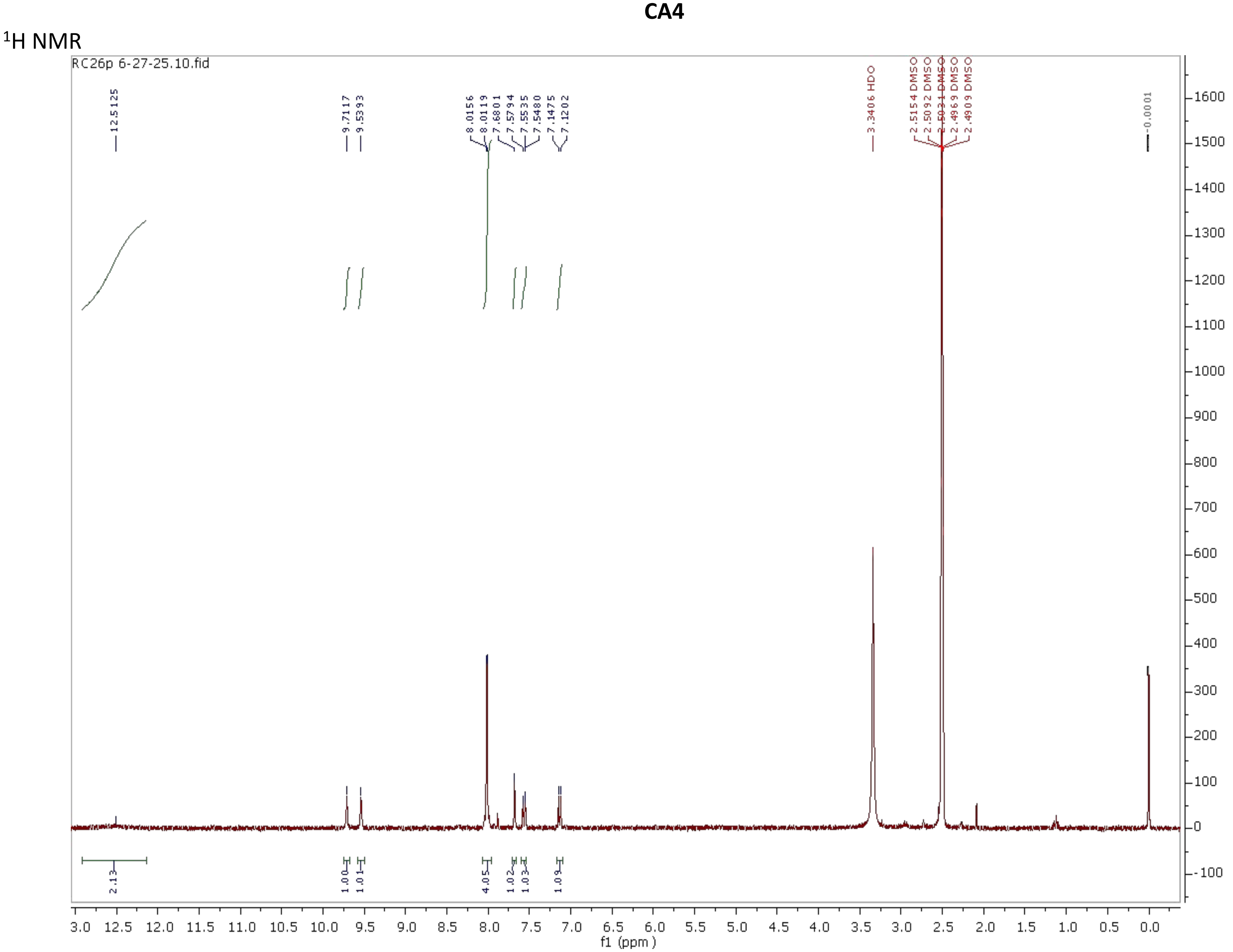
CA4
1H NMR
RC26p 6-27-25.10.fid
12.5125
9.7117
9.5393
8.0156
8.0119
7.6801
7.5794
7.5535
7.5480
7.1475
7.1202
3.3406 HDO
2.5154 DMSO
2.5092 DMSO
2.5031 DMSO
2.4969 DMSO
2.4909 DMSO
-0.0001
2.13
1.00
1.01
4.05
1.02
1.03
1.09
f1 (ppm)

CA4

^{13}C NMR

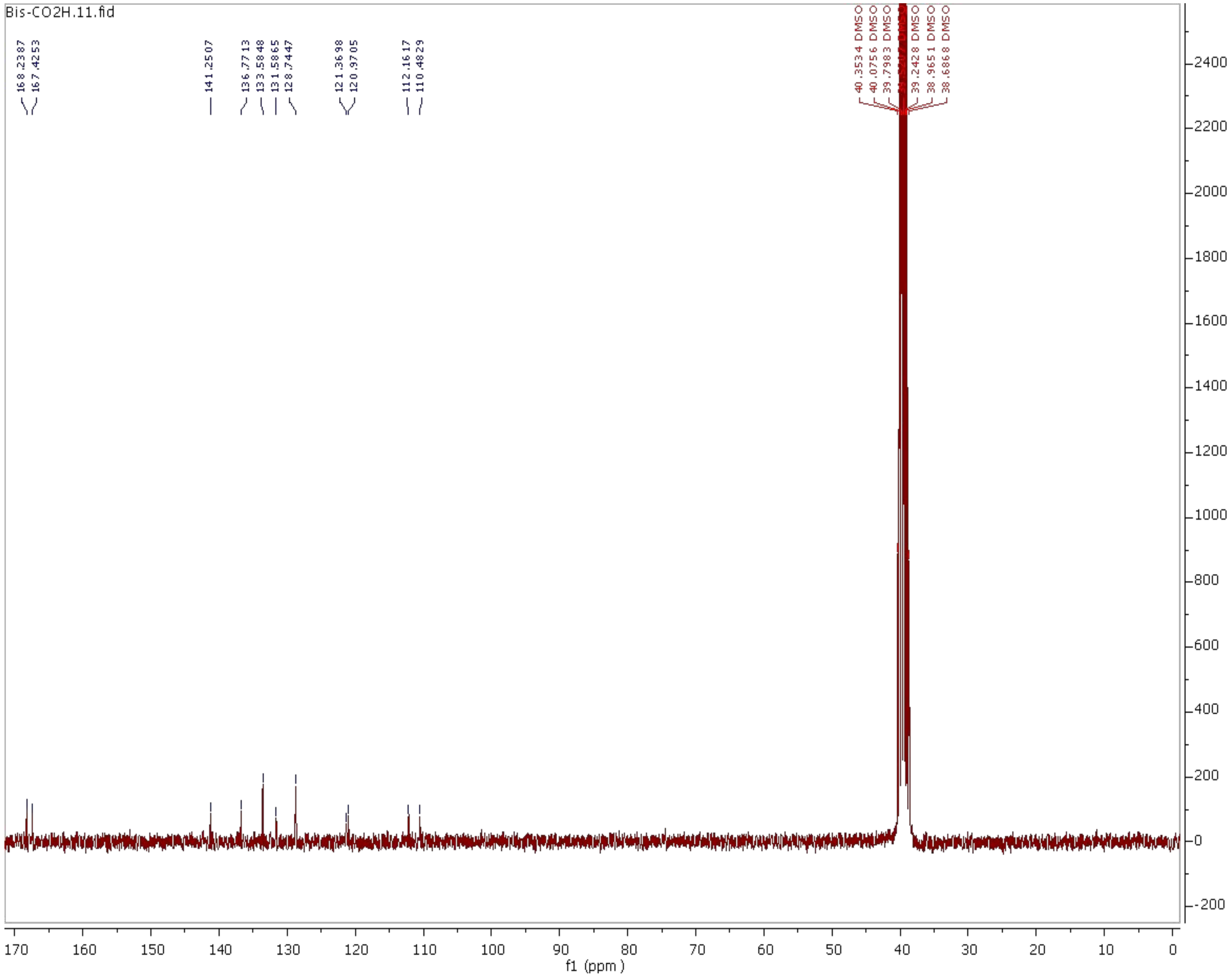

Bis-CO2H.11.fid
168.2387
167.4253
141.2507
136.7713
133.5848
131.5865
128.7447
121.3698
120.9705
112.1617
110.4829
40.3534 DMSO
40.0756 DMSO
39.7983 DMSO
39.2428 DMSO
38.9651 DMSO
38.6868 DMSO
f1 (ppm)

CA4

IR

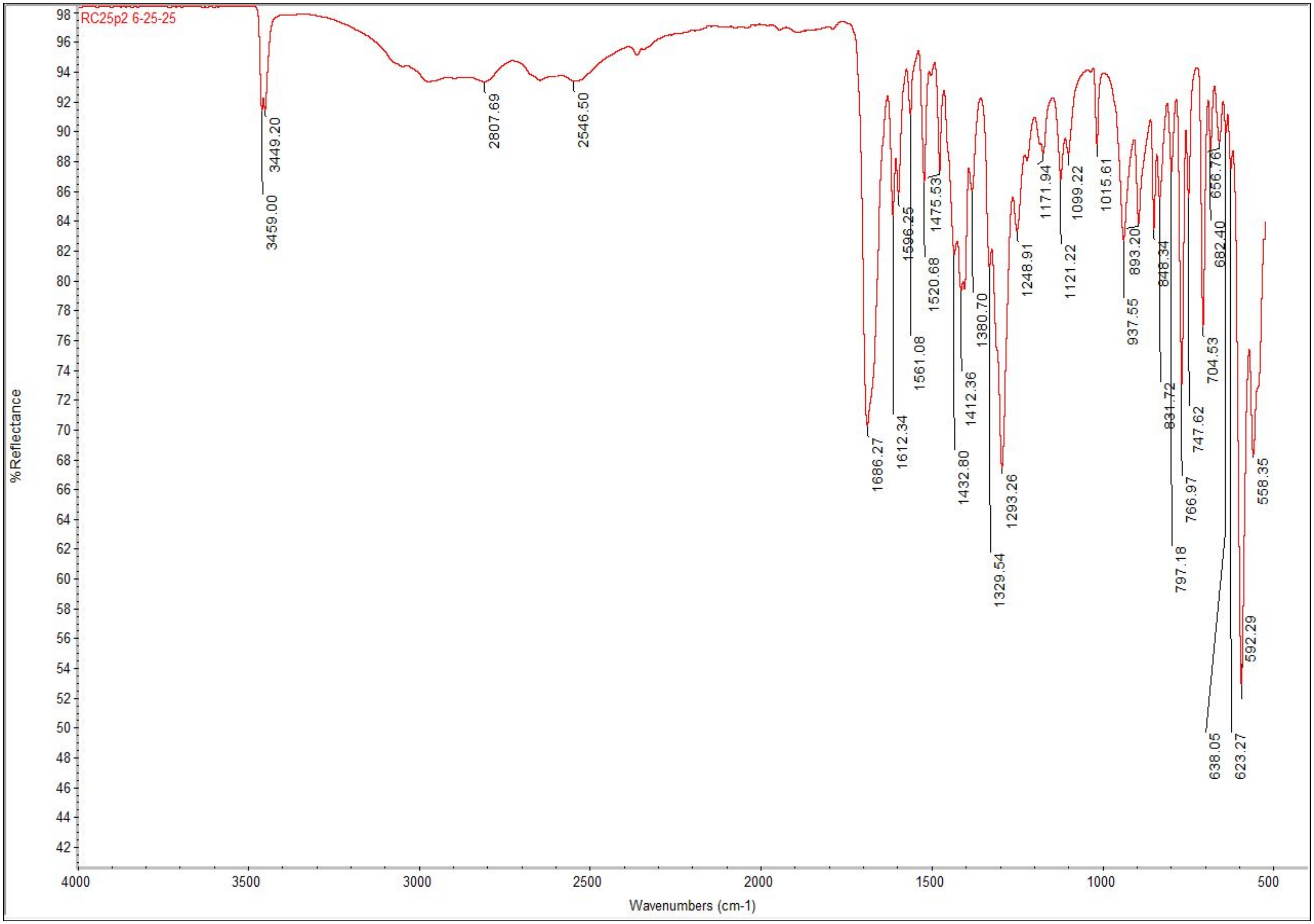
RC25p2 6-25-25
%Reflectance
Wavenumbers (cm-1)
3459.00
3449.20
2807.69
2546.50
1686.27
1612.34
1596.25
1561.08
1520.68
1475.53
1432.80
1412.36
1380.70
1329.54
1293.26
1248.91
1171.94
1121.22
1099.22
1015.61
937.55
893.20
848.34
831.72
797.18
766.97
747.62
704.53
682.40
656.76
638.05
623.27
592.29
558.35

CA4

UV-vis

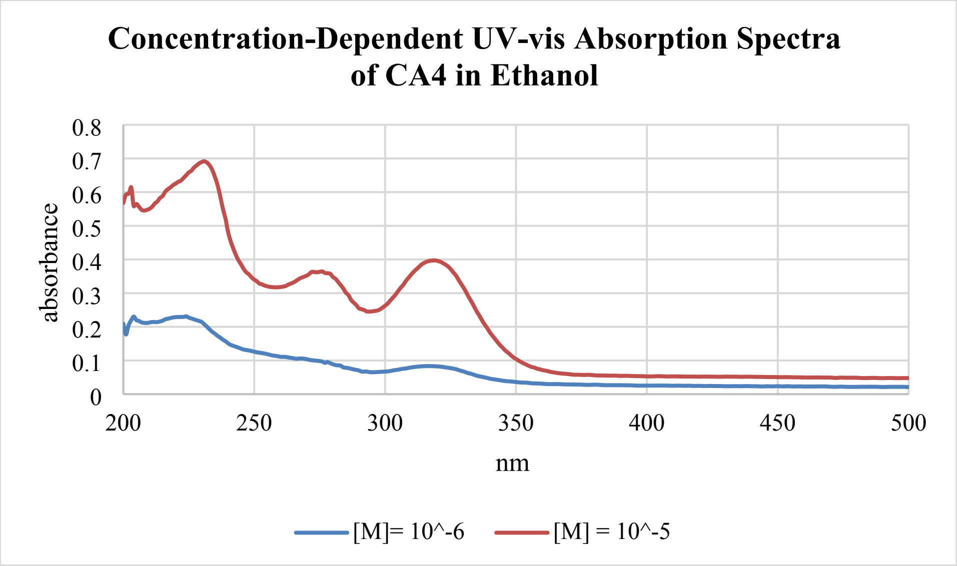

Concentration-Dependent UV-vis Absorption Spectra of CA4 in Ethanol
absorbance
0.8
0.7
0.6
0.5
0.4
0.3
0.2
0.1
0
200
250
300
350
400
450
500
nm
[M]= 10^-6
[M] = 10^-5

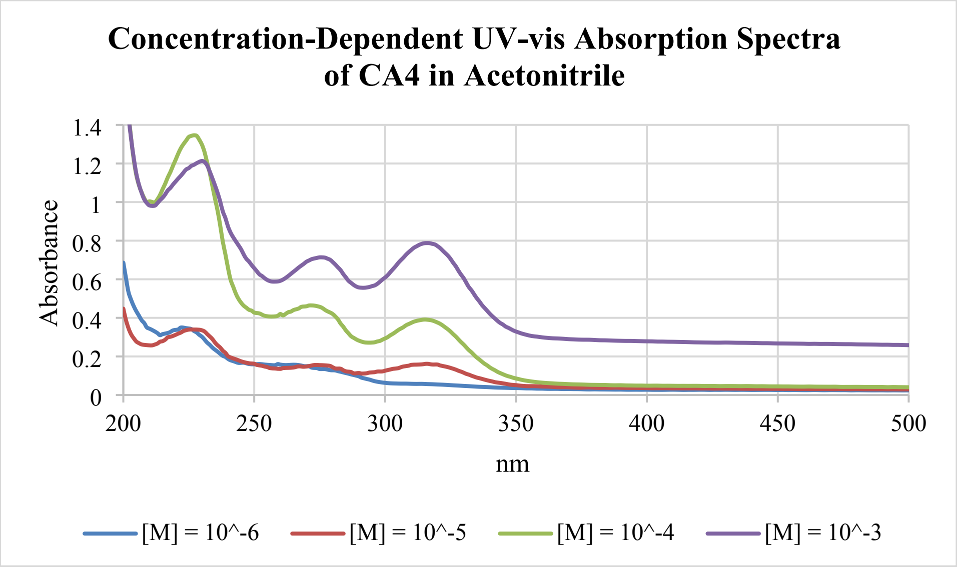

Concentration-Dependent UV-vis Absorption Spectra of CA4 in Acetonitrile
Absorbance
1.4
1.2
1
0.8
0.6
0.4
0.2
0
200
250
300
350
400
450
500
nm
[M] = 10^-6
[M] = 10^-5
[M] = 10^-4
[M] = 10^-3

CA4

Fluorescence Emission

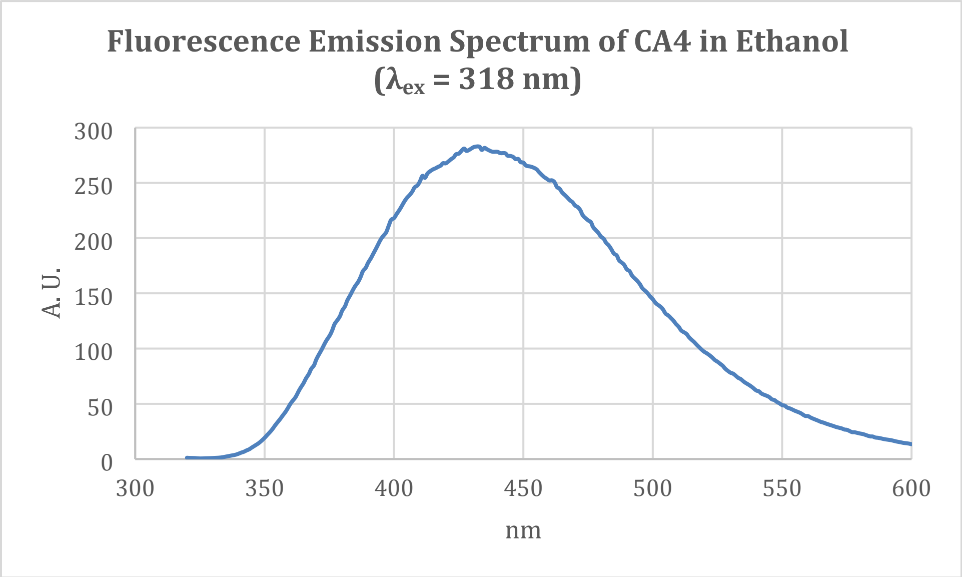

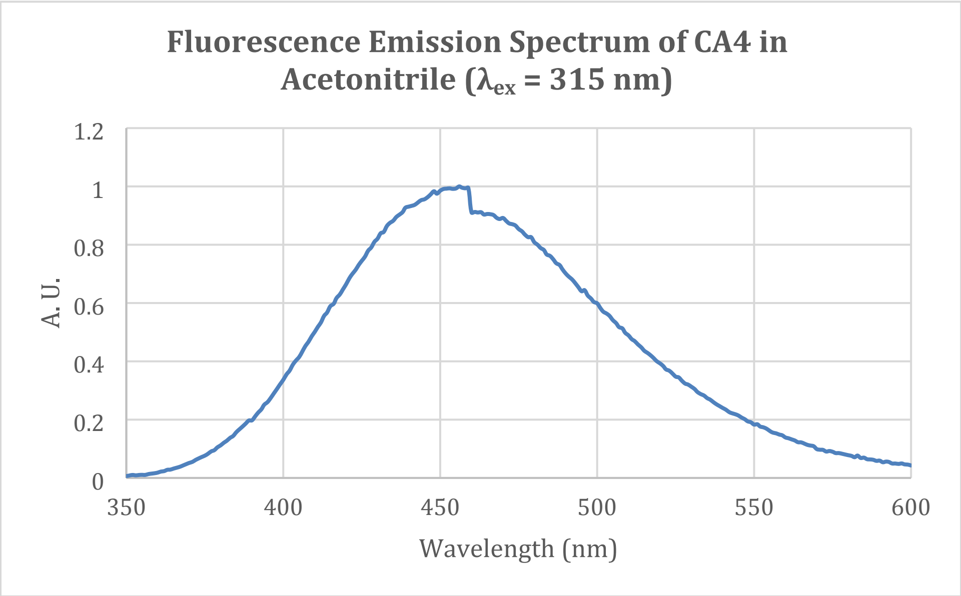

CA4

Fluorescence Excitation

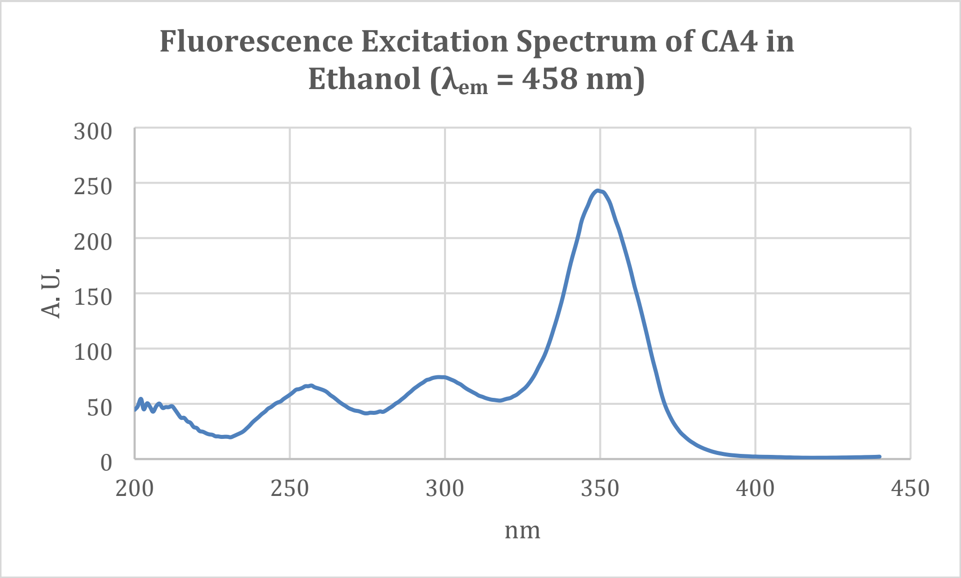

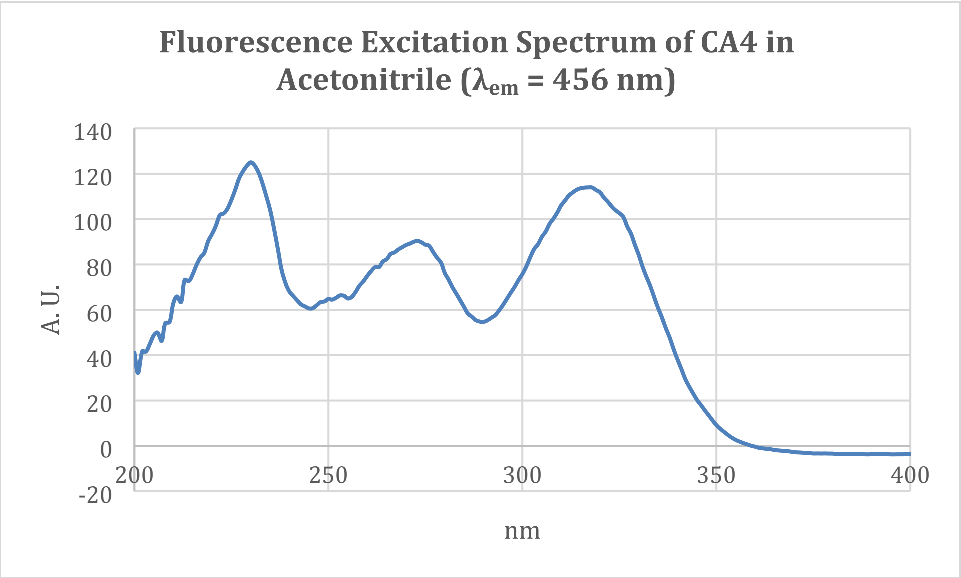

CA4

HRMS

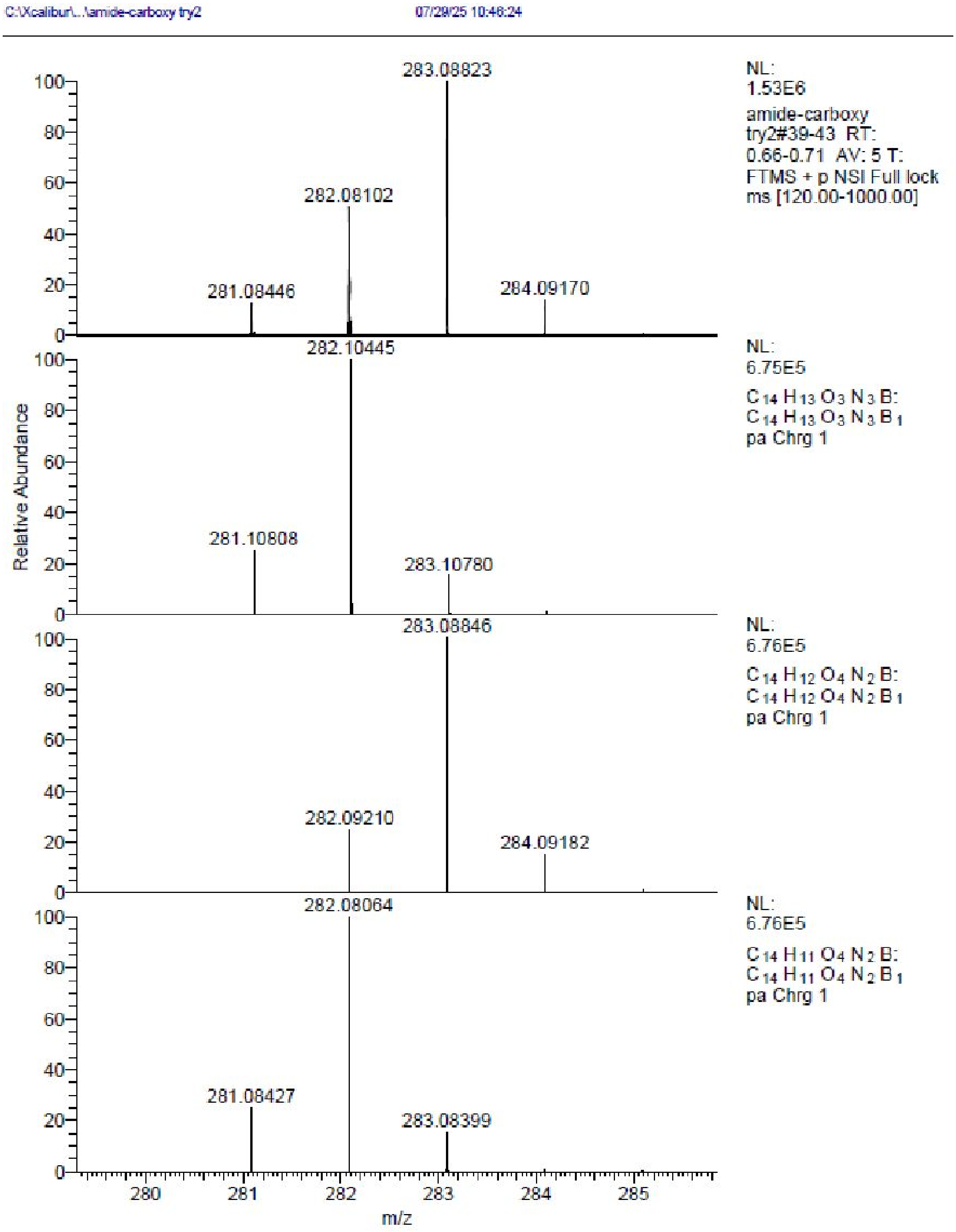
C:\Xcalibur\...\amide-carboxy try2
07/29/25 10:46:24
NL:
1.53E6
amide-carboxy try2#39-43 RT: 0.66-0.71 AV: 5 T: FTMS + p NSI Full lock ms [120.00-1000.00]
283.08823
282.08102
281.08446
284.09170
NL:
6.75E5
C 14 H 13 O 3 N 3 B:
C 14 H 13 O 3 N 3 B 1
pa Chrg 1
282.10445
281.10808
283.10780
NL:
6.76E5
C 14 H 12 O 4 N 2 B:
C 14 H 12 O 4 N 2 B 1
pa Chrg 1
283.08846
282.09210
284.09182
NL:
6.76E5
C 14 H 11 O 4 N 2 B:
C 14 H 11 O 4 N 2 B 1
pa Chrg 1
282.08064
281.08427
283.08399
Relative Abundance
m/z

CA4

Computational Data

MO 74/83
Energy = -0.0625 a.u.
Symmetry = A
Use the slider to adjust cutoff (start with 0.05)

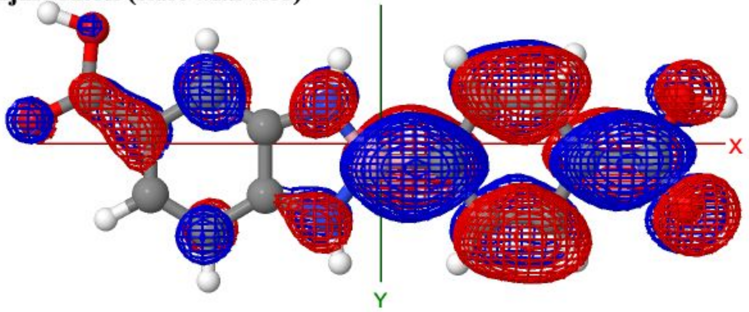

MO 73/83
Energy = -0.2115 a.u.
Symmetry = A
Use the slider to adjust cutoff (start with 0.05)

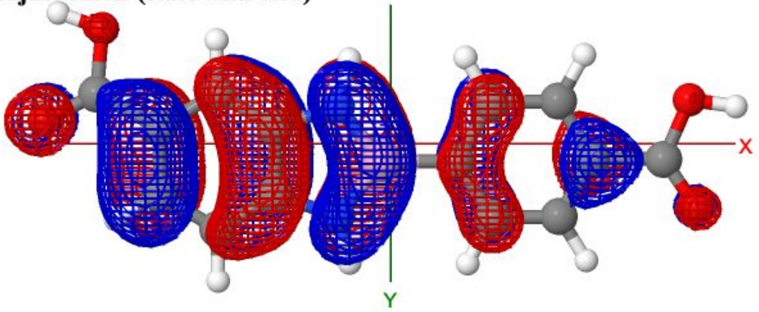

CN4

CN4

^{1}H NMR

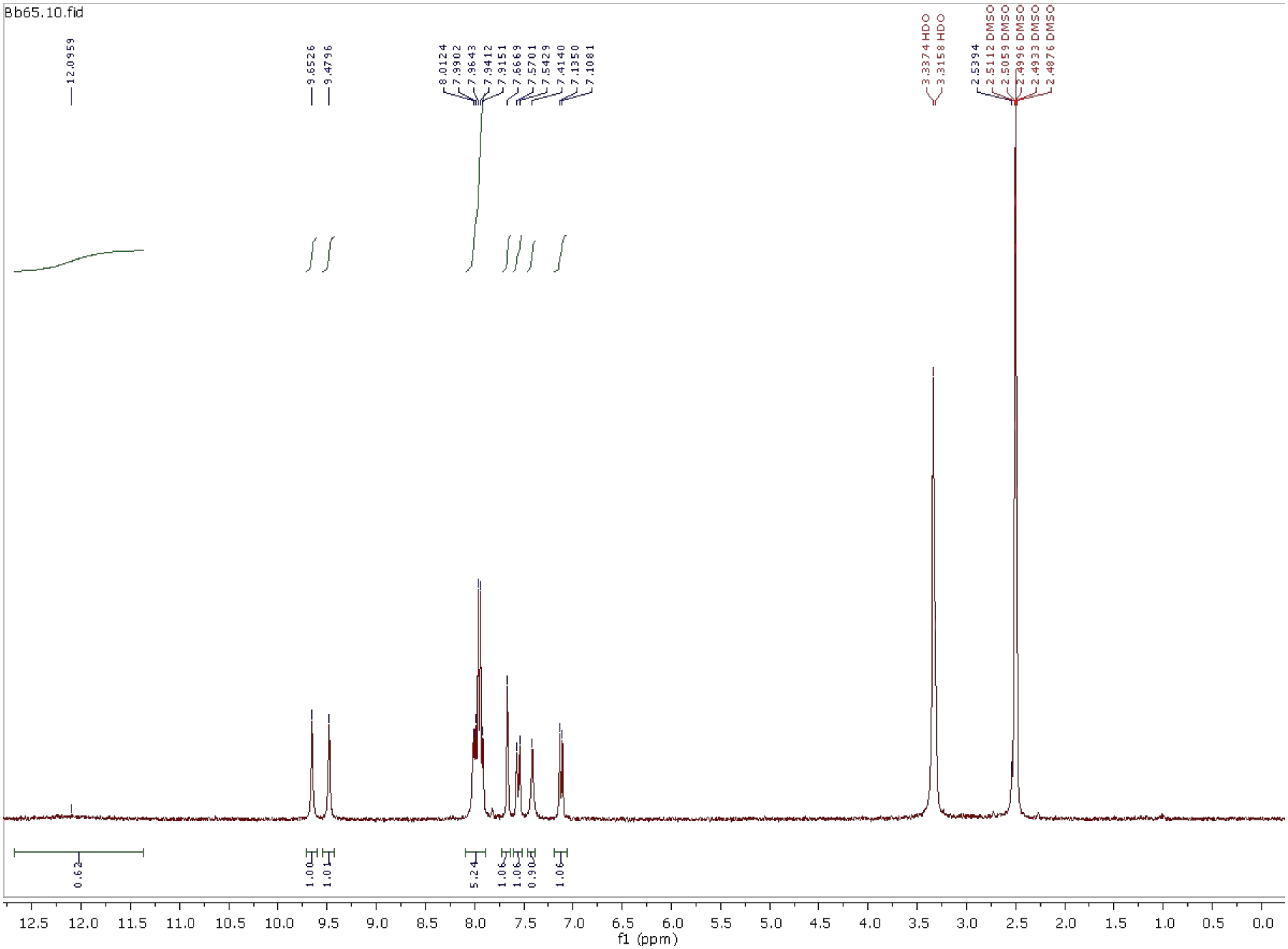

CN4

^{13}C NMR

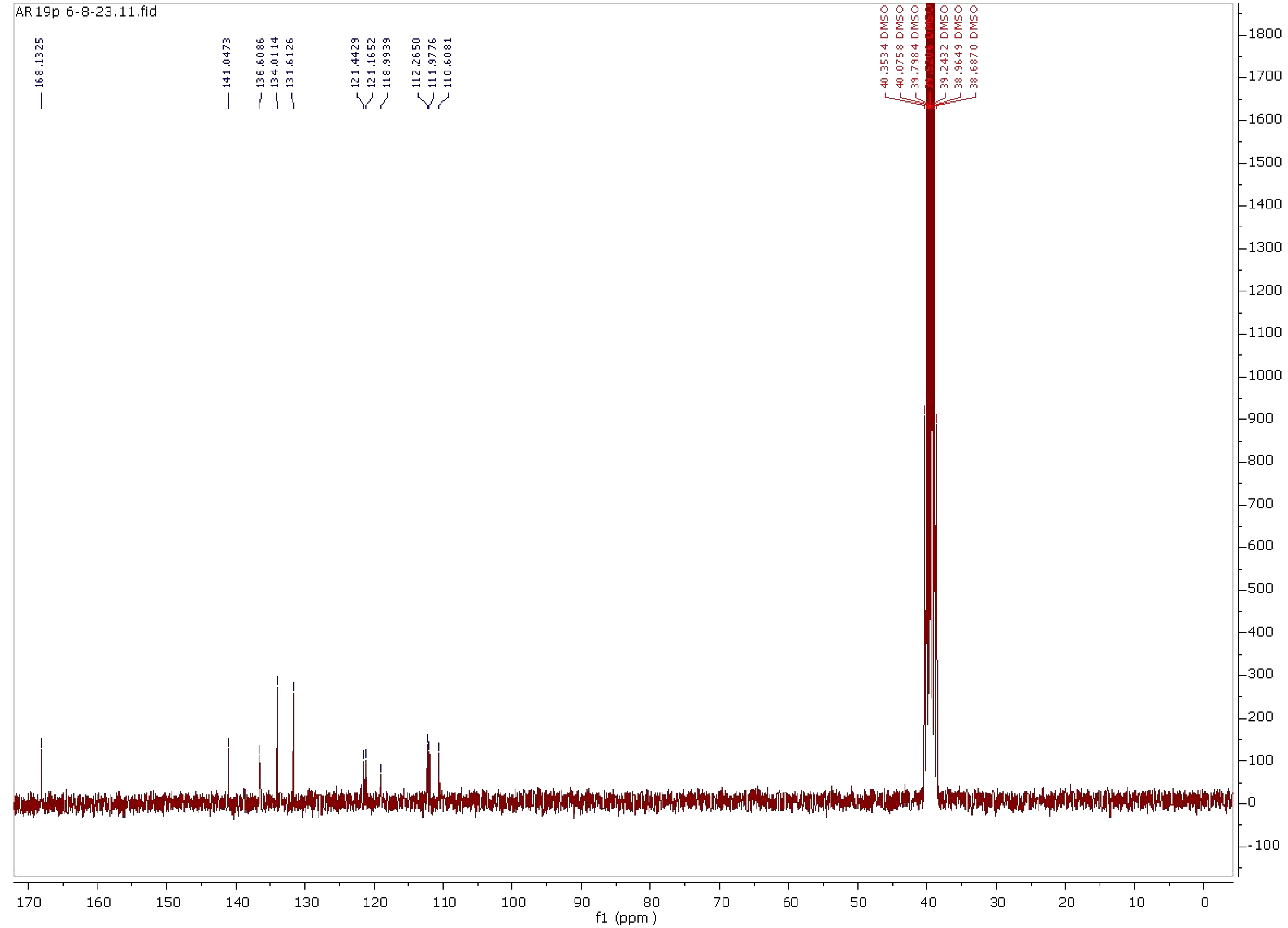

AR 19p 6-8-23.11.fid
168.1325
141.0473
136.6086
134.0114
131.6126
121.4429
121.1652
118.9939
112.2650
111.9776
110.6081
40.3534 DMSO
40.0758 DMSO
39.7984 DMSO
39.2432 DMSO
38.9649 DMSO
38.6870 DMSO
1800
1700
1600
1500
1400
1300
1200
1100
1000
900
800
700
600
500
400
300
200
100
0
-100
170
160
150
140
130
120
110
100
90
80
70
60
50
40
30
20
10
0
f1 (ppm)

CN4

IR

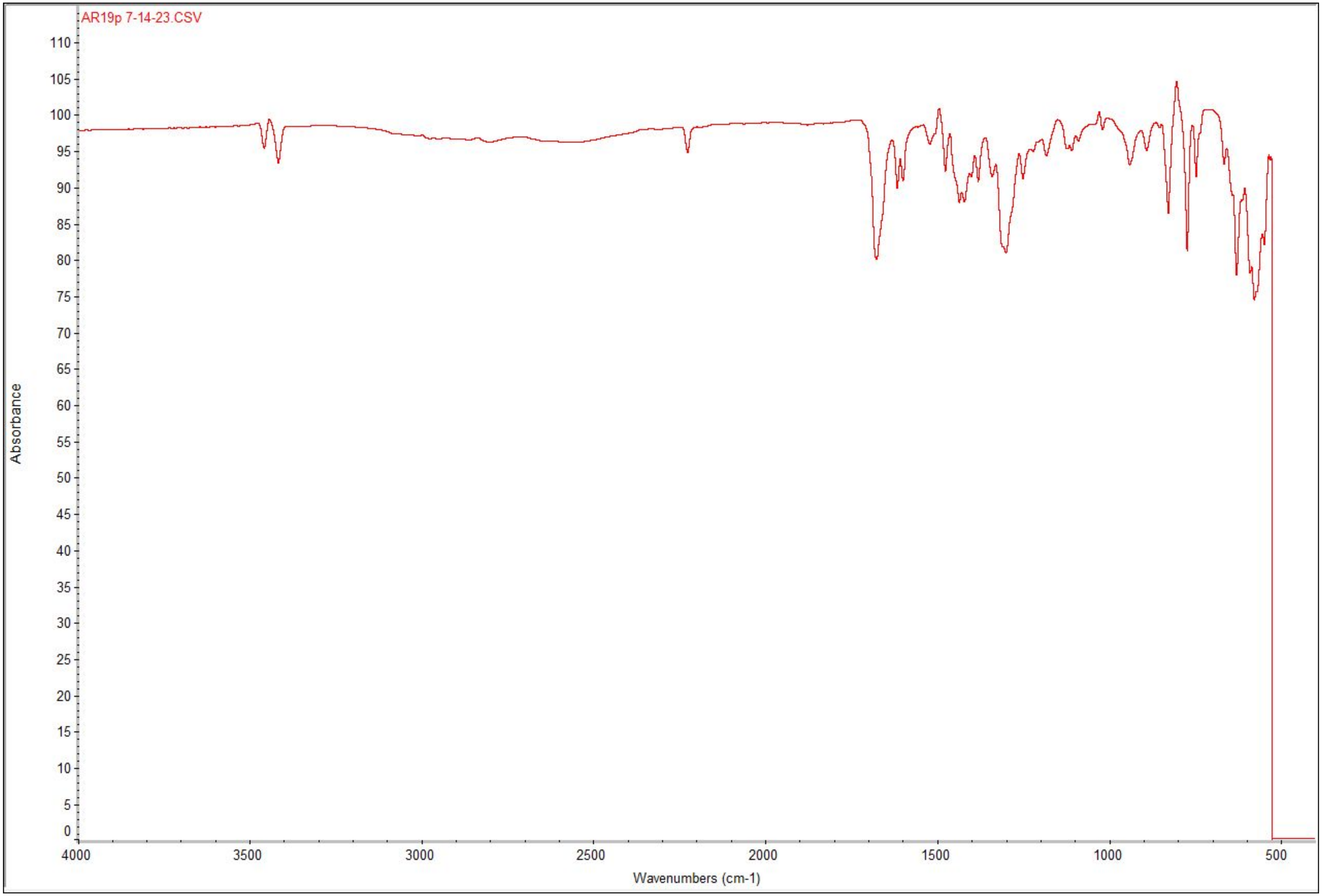

AR19p 7-14-23.CSV
Absorbance
Wavenumbers (cm-1)
110
105
100
95
90
85
80
75
70
65
60
55
50
45
40
35
30
25
20
15
10
5
0
4000
3500
3000
2500
2000
1500
1000
500

CN4

UV-vis

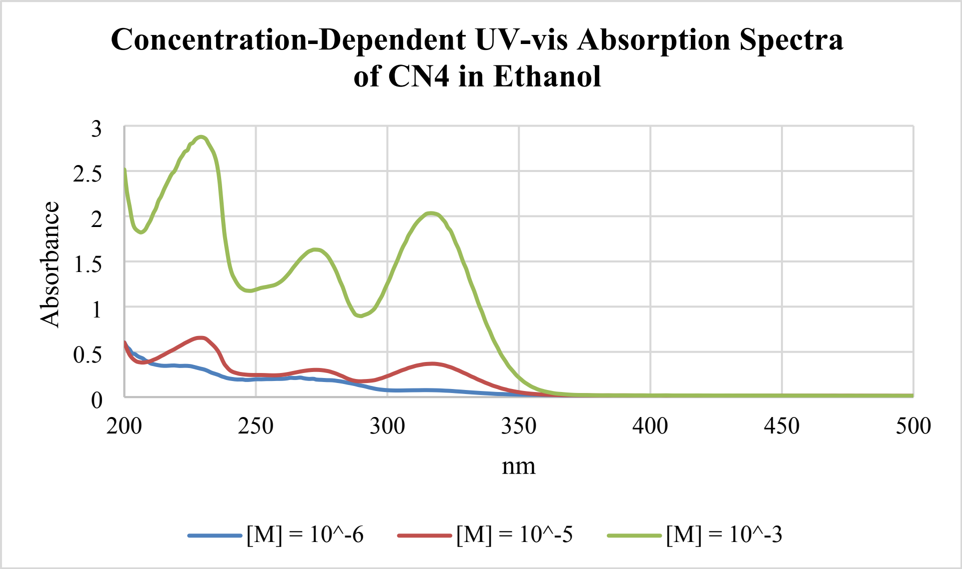
Concentration-Dependent UV-vis Absorption Spectra of CN4 in Ethanol
3
2.5
2
1.5
1
0.5
0
Absorbance
200
250
300
350
400
450
500
nm
[M] = 10^-6
[M] = 10^-5
[M] = 10^-3

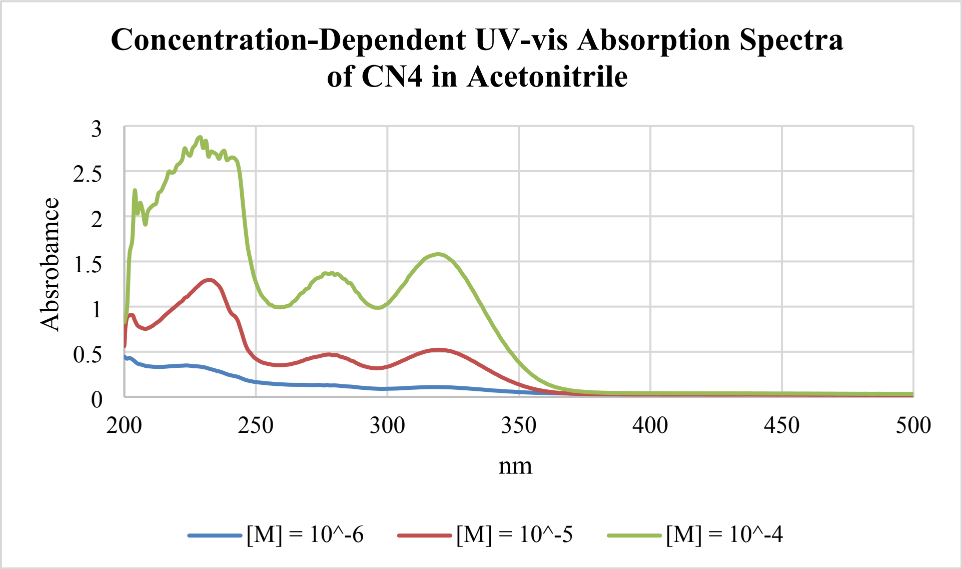
Concentration-Dependent UV-vis Absorption Spectra of CN4 in Acetonitrile
3
2.5
2
1.5
1
0.5
0
Absrobamce
200
250
300
350
400
450
500
nm
[M] = 10^-6
[M] = 10^-5
[M] = 10^-4

CN4

Fluorescence Emission

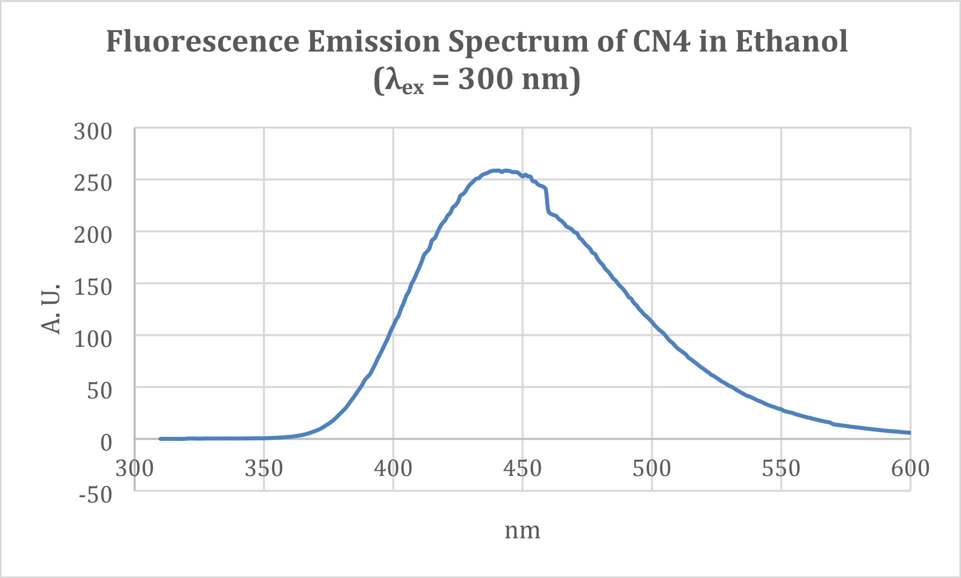

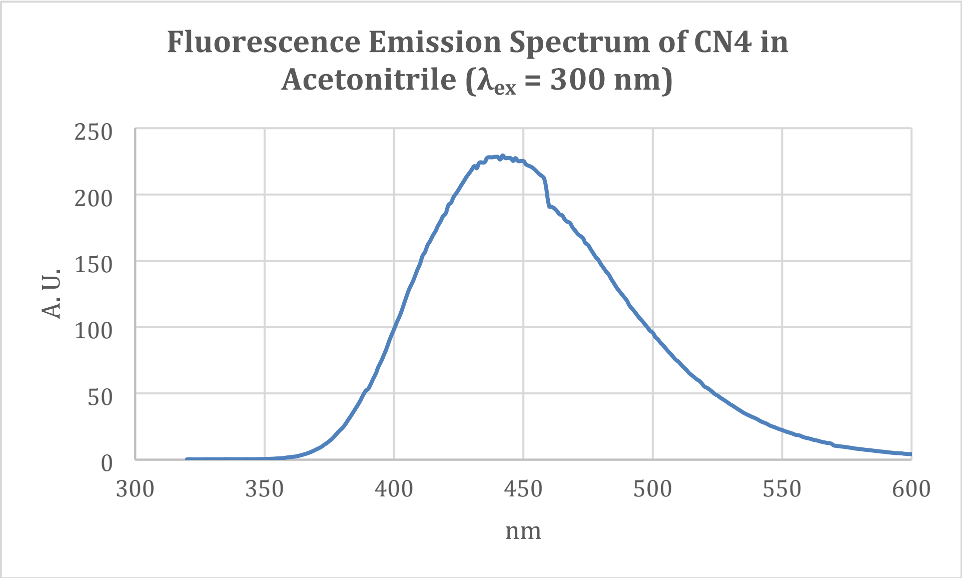

CN4

Fluorescence Excitation

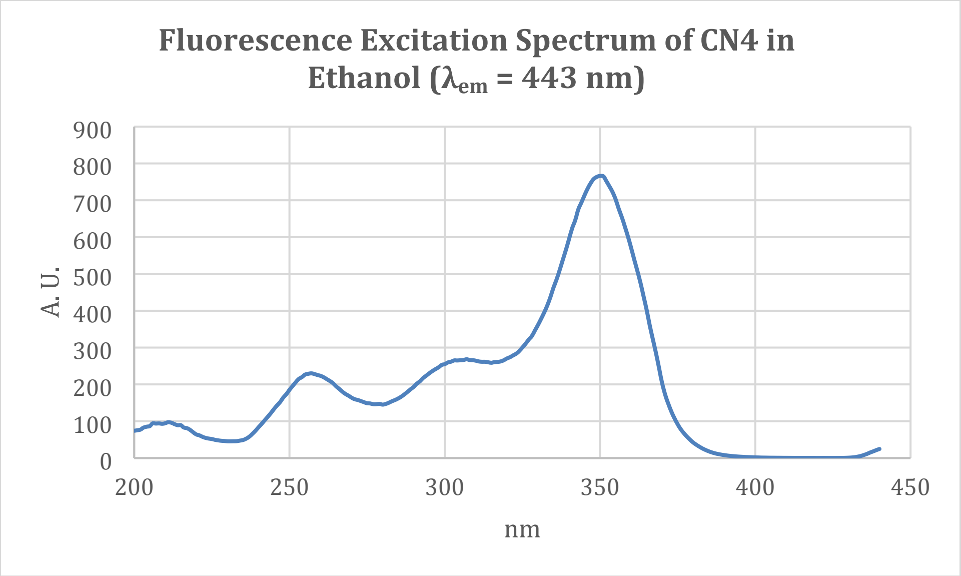

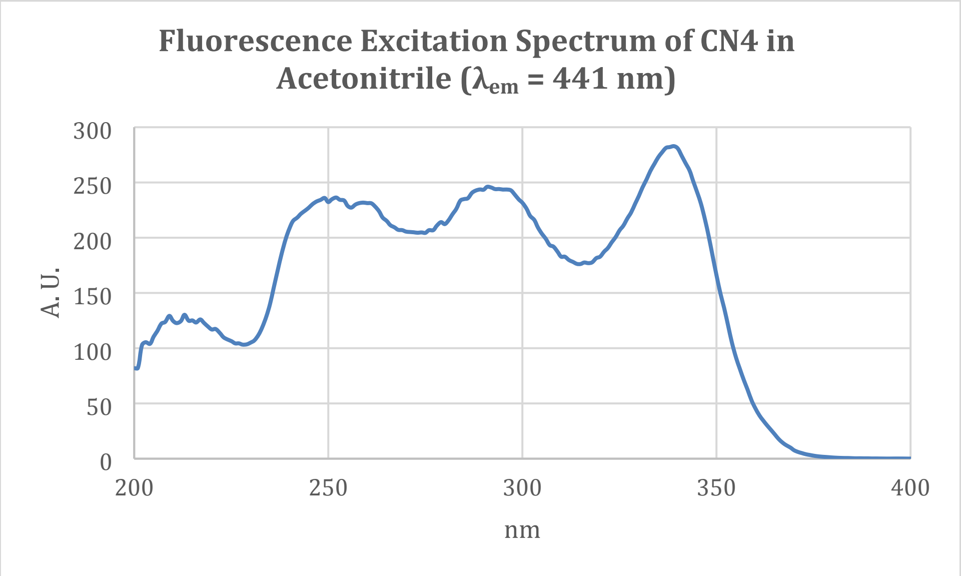

CN4

HRMS

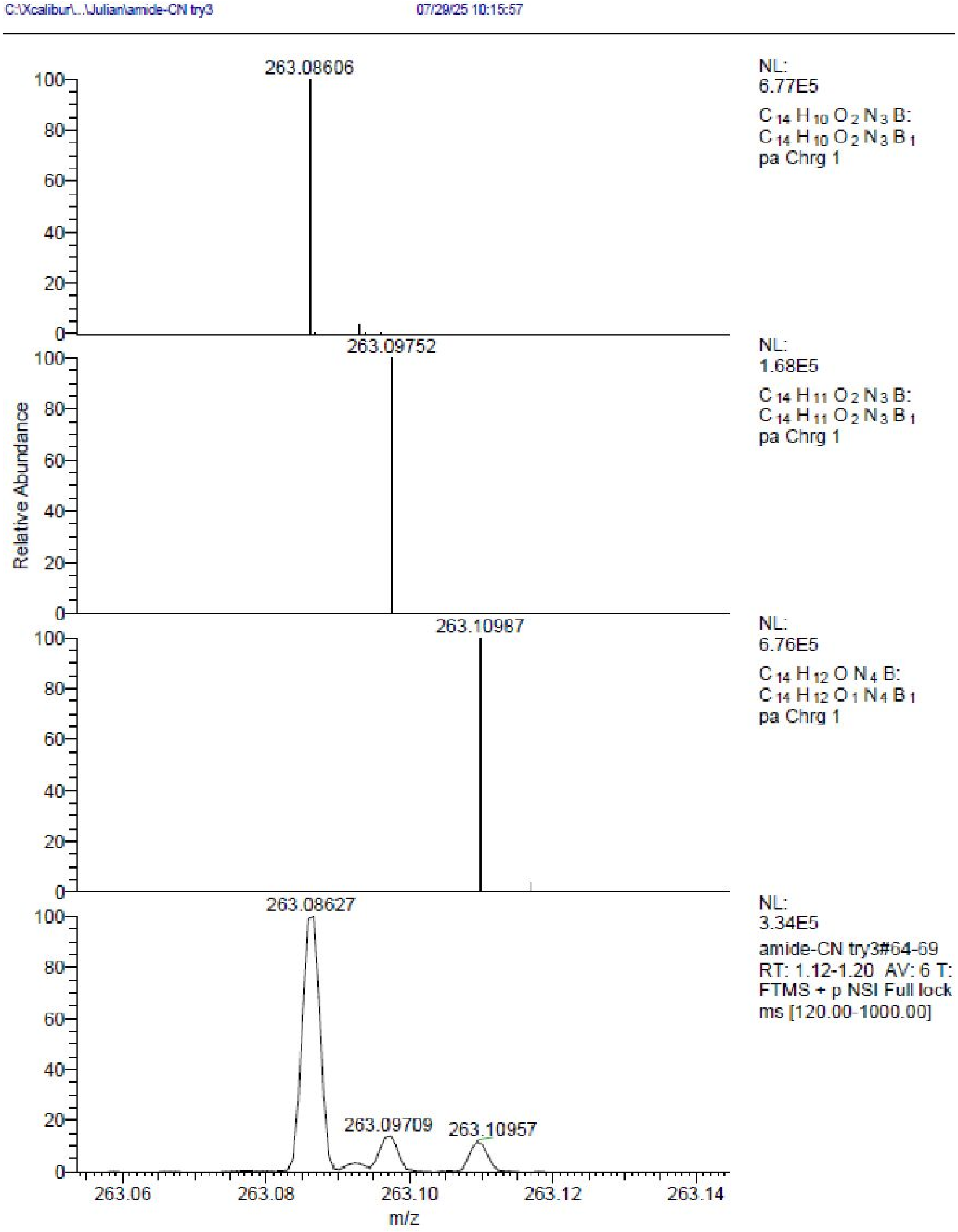

C:\Xcalibur\...\Julian\amide-CN try3
07/29/25 10:15:57
263.08606
NL:
6.77E5
C 14 H 10 O 2 N 3 B:
C 14 H 10 O 2 N 3 B 1
pa Chrg 1
263.09752
NL:
1.68E5
C 14 H 11 O 2 N 3 B:
C 14 H 11 O 2 N 3 B 1
pa Chrg 1
263.10987
NL:
6.76E5
C 14 H 12 O N 4 B:
C 14 H 12 O 1 N 4 B 1
pa Chrg 1
263.08627
263.09709
263.10957
NL:
3.34E5
amide-CN try3#64-69
RT: 1.12-1.20 AV: 6 T:
FTMS + p NSI Full lock
ms [120.00-1000.00]
Relative Abundance
m/z

CN4

Computational Data

MO 69/78
Energy = -0.0724 a.u.
Symmetry = A
Use the slider to adjust cutoff (start with 0.05)

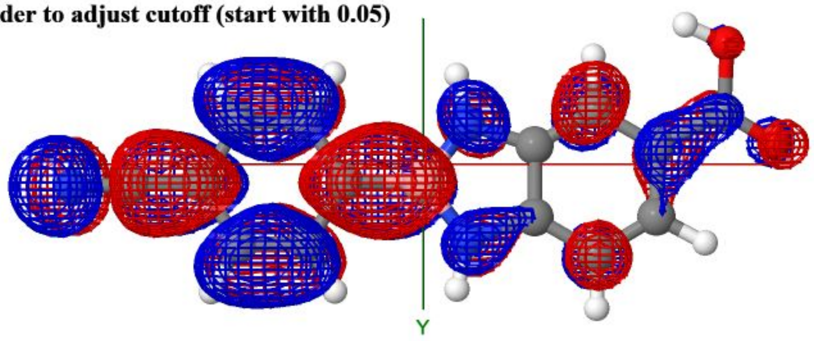

MO 68/78
Energy = -0.2233 a.u.
Symmetry = A
Use the slider to adjust cutoff (start with 0.05)

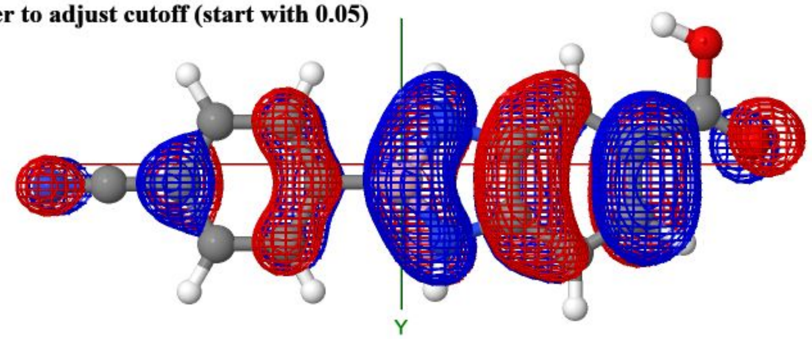

Am4

Am4

^{1}H NMR

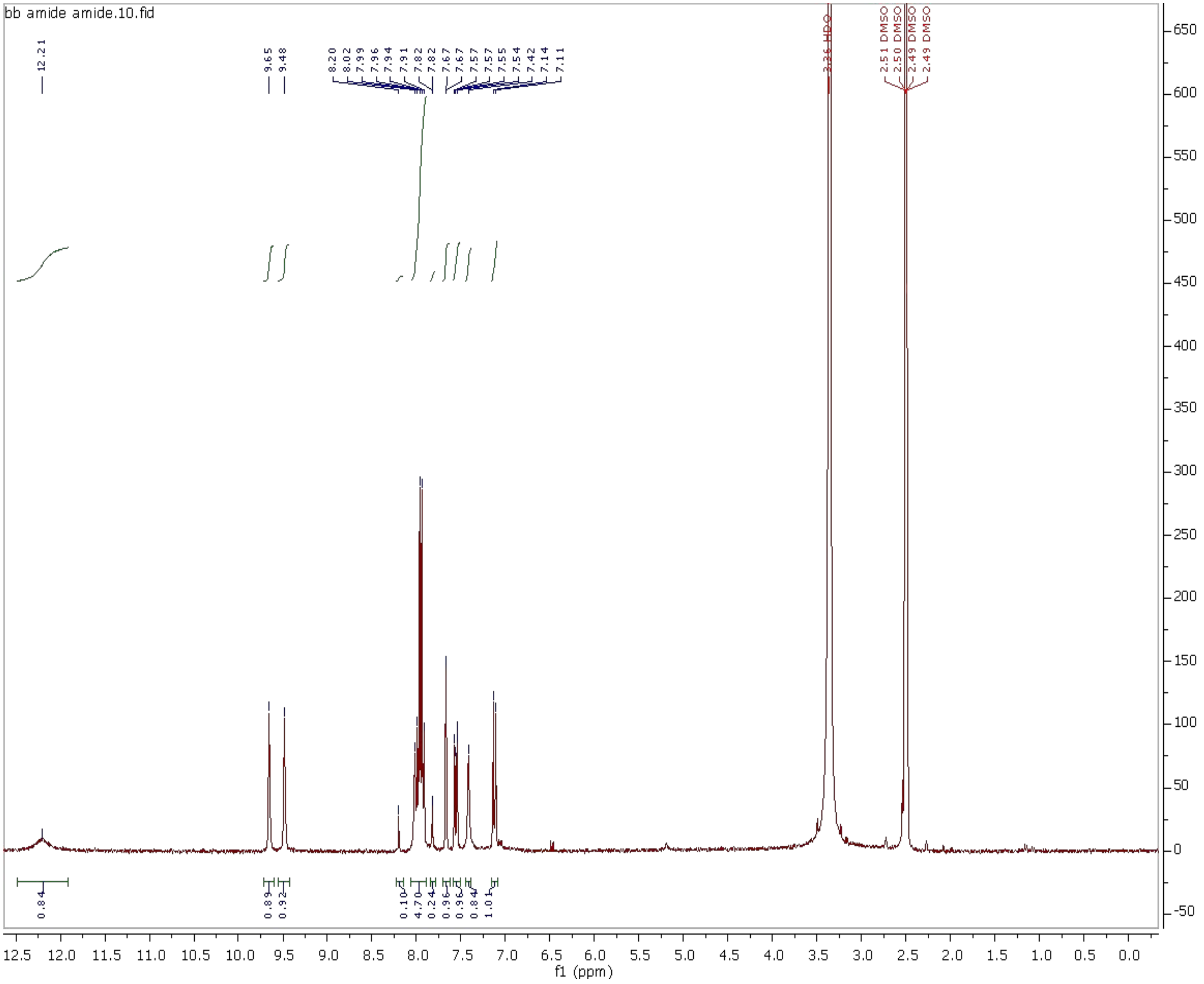

Am4

IR

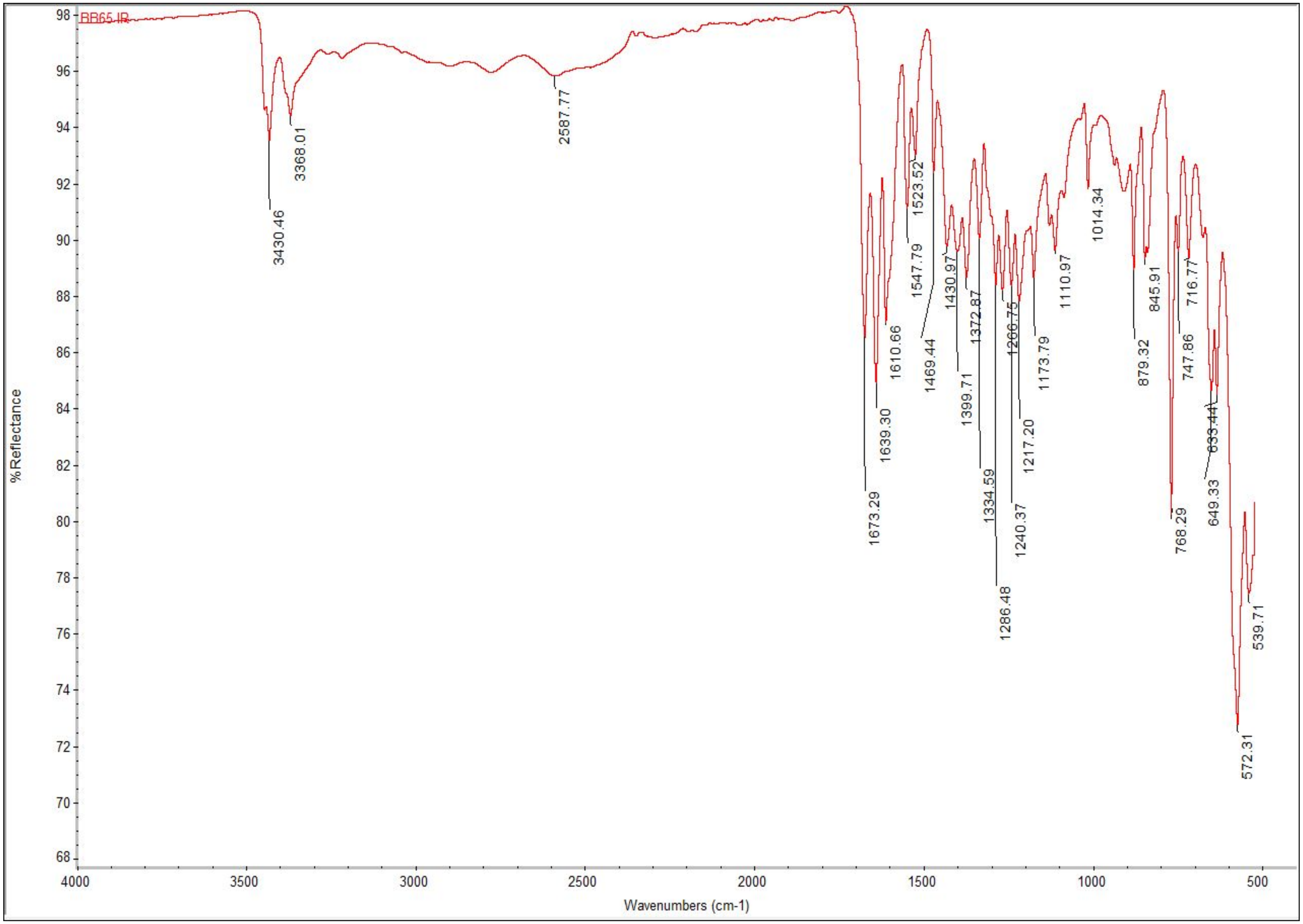
BR65 IR
%Reflectance
Wavenumbers (cm-1)
3430.46
3368.01
2587.77
1673.29
1639.30
1610.66
1547.79
1523.52
1469.44
1430.97
1399.71
1372.87
1334.59
1286.48
1266.75
1240.37
1217.20
1173.79
1110.97
1014.34
879.32
845.91
768.29
747.86
716.77
649.33
633.44
572.31
539.71

Am4

UV-vis

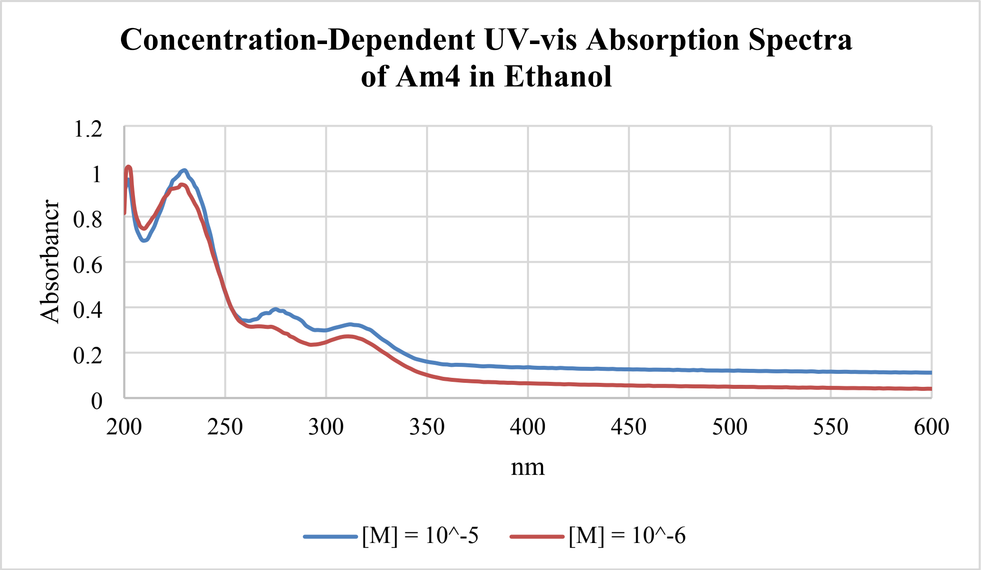

Concentration-Dependent UV-vis Absorption Spectra of Am4 in Ethanol
Absorbancr
1.2
1
0.8
0.6
0.4
0.2
0
200
250
300
350
400
450
500
550
600
nm
[M] = 10^-5
[M] = 10^-6

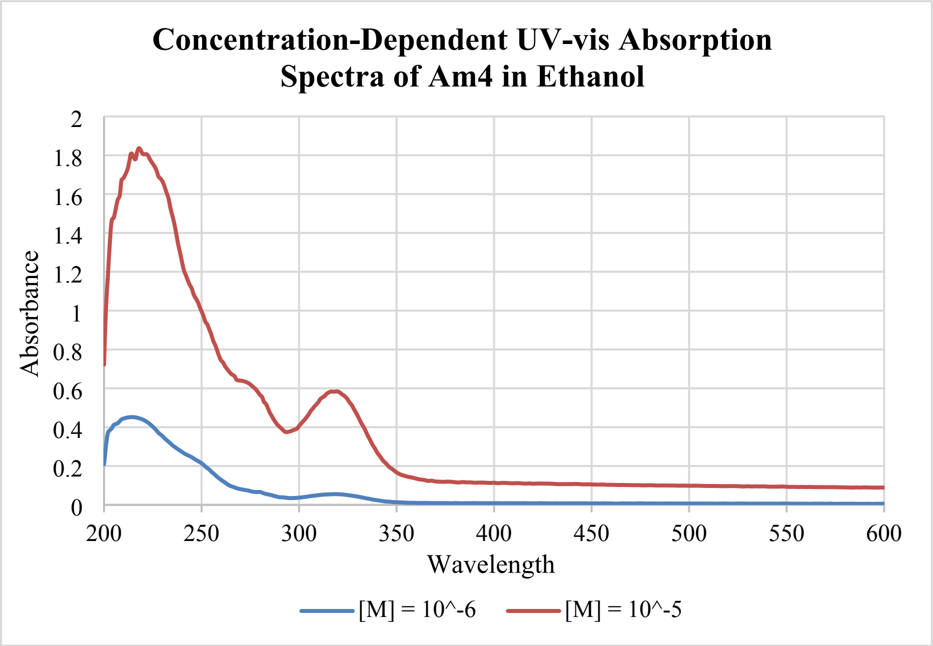

Concentration-Dependent UV-vis Absorption Spectra of Am4 in Ethanol
Absorbance
2
1.8
1.6
1.4
1.2
1
0.8
0.6
0.4
0.2
0
200
250
300
350
400
450
500
550
600
Wavelength
[M] = 10^-6
[M] = 10^-5

Am4

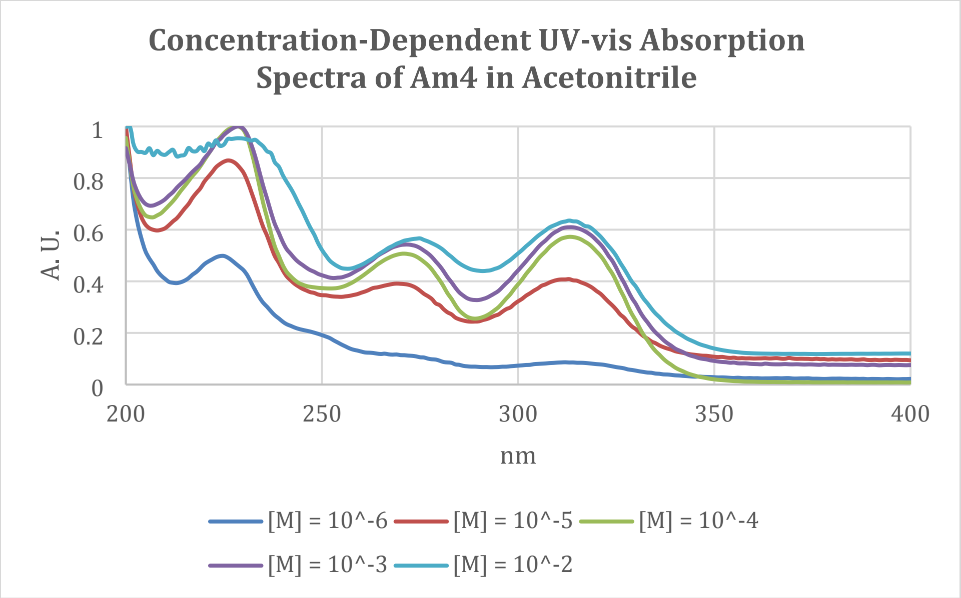

Fluorescence Emission

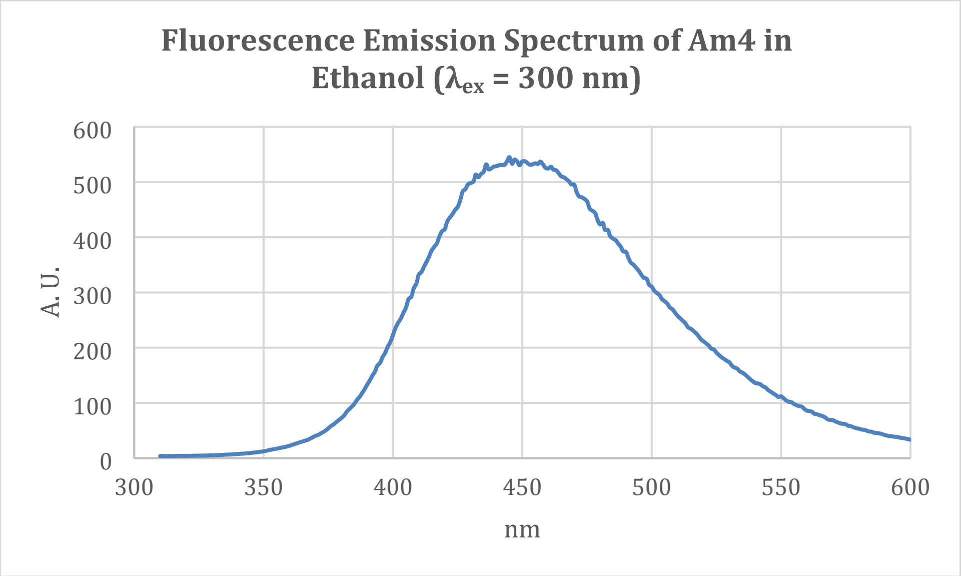

Am4

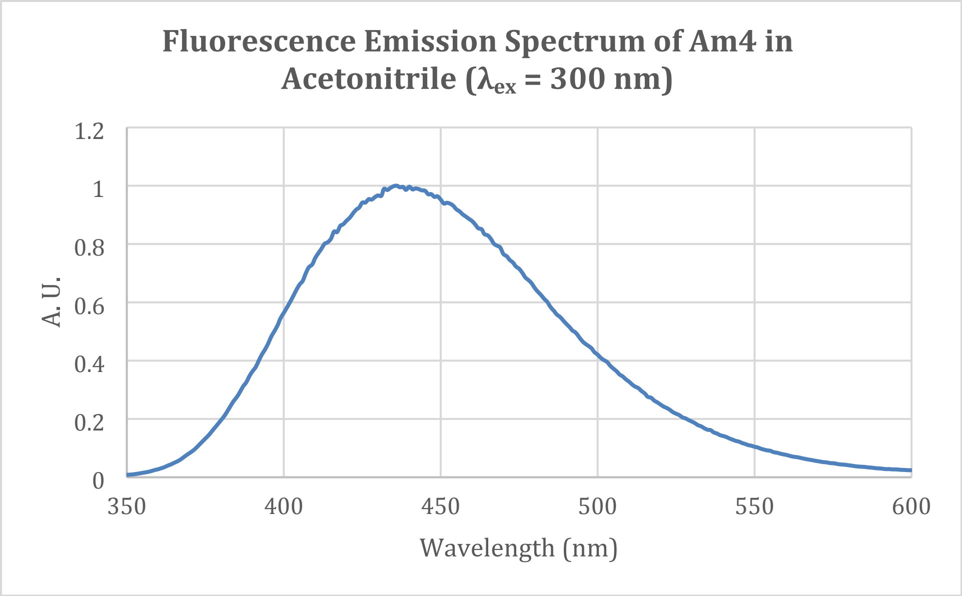

Fluorescence Excitation

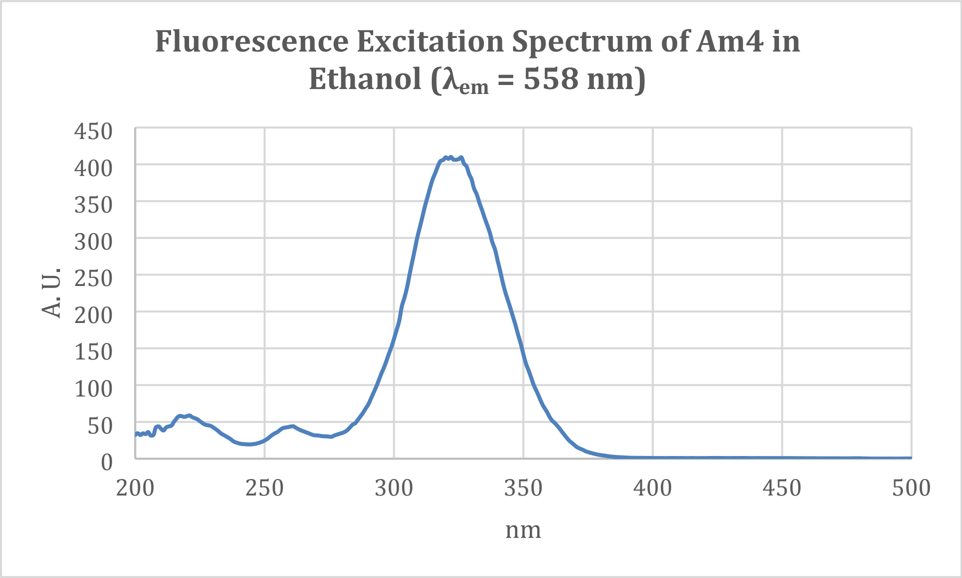

Am4

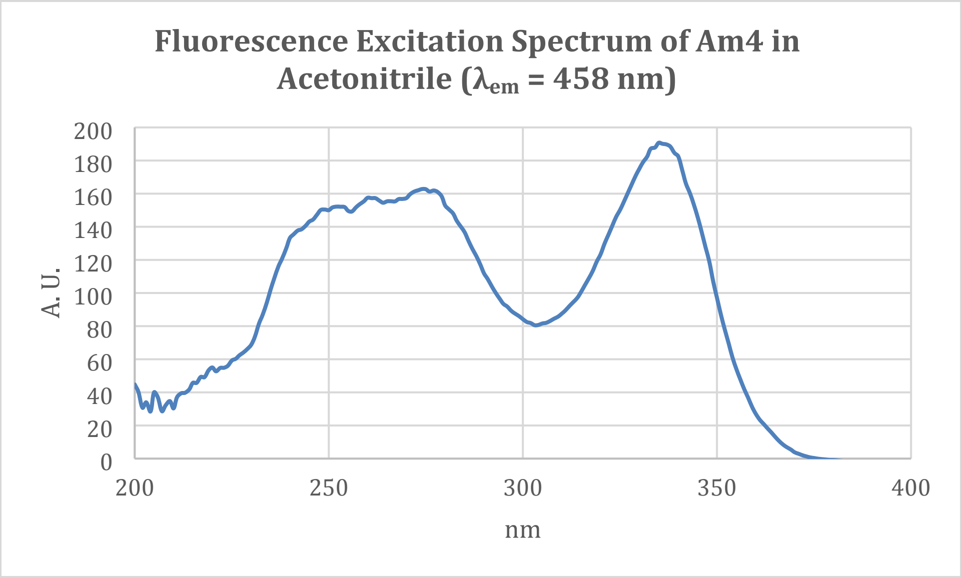

Fluorescence Excitation Spectrum of Am4 in Acetonitrile (λem = 458 nm)
200
180
160
140
120
100
80
60
40
20
0
A. U.
200
250
300
350
400
nm

Am4

HRMS

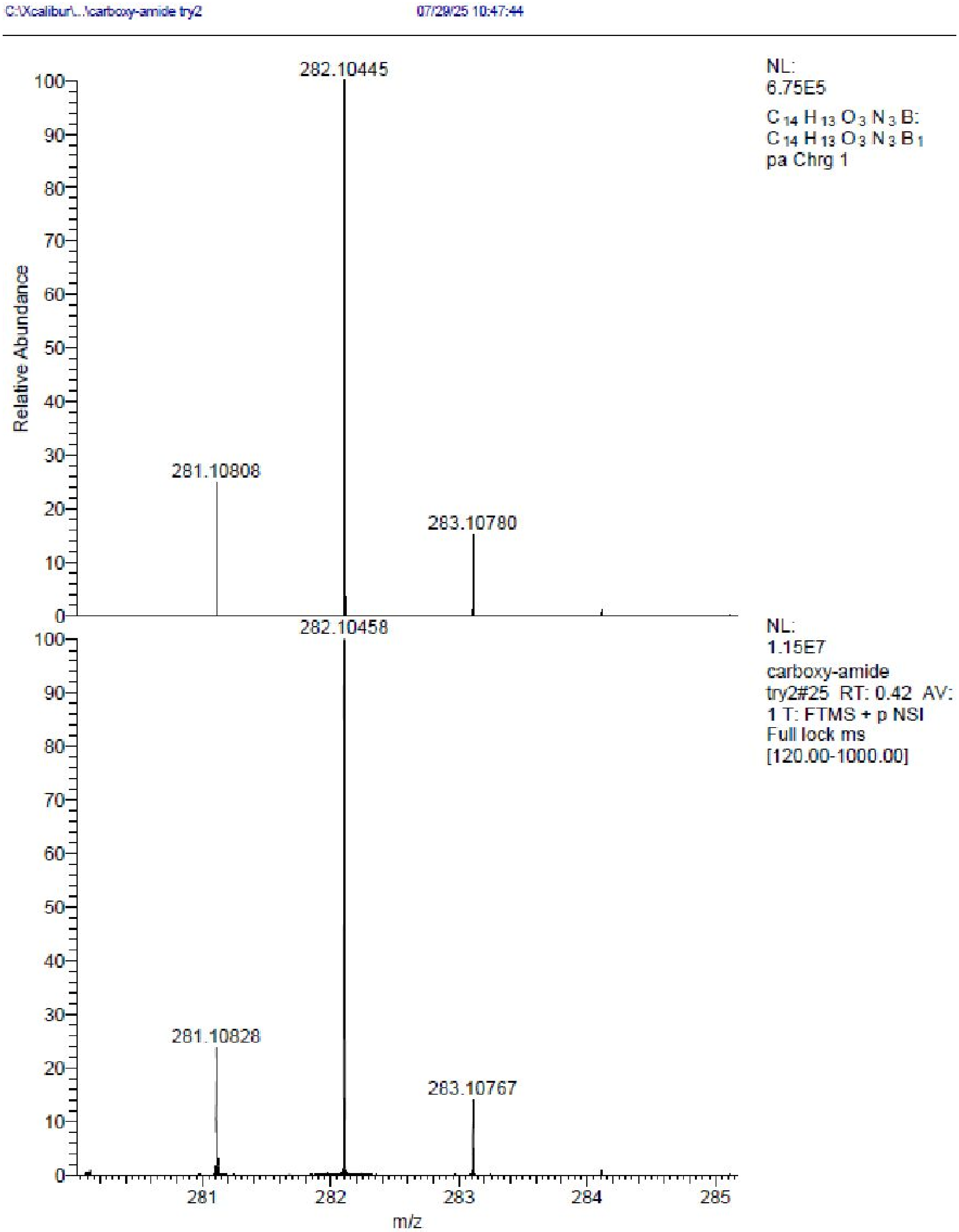
C:\Xcalibur\...\carboxy-amide try2
07/29/25 10:47:44
282.10445
281.10808
283.10780
NL:
6.75E5
C 14 H 13 O 3 N 3 B:
C 14 H 13 O 3 N 3 B 1
pa Chrg 1
282.10458
281.10828
283.10767
NL:
1.15E7
carboxy-amide
try2#25 RT: 0.42 AV:
1 T: FTMS + p NSI
Full lock ms
[120.00-1000.00]
Relative Abundance
m/z

Am4

Computational Data

MO 74/83
Energy = -0.0596 a.u.
Symmetry = A
Use the slider to adjust cutoff (start with 0.05)

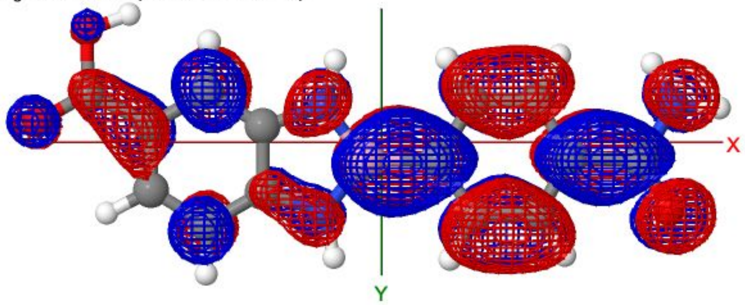

MO 73/83
Energy = -0.2163 a.u.
Symmetry = A
Use the slider to adjust cutoff (start with 0.05)

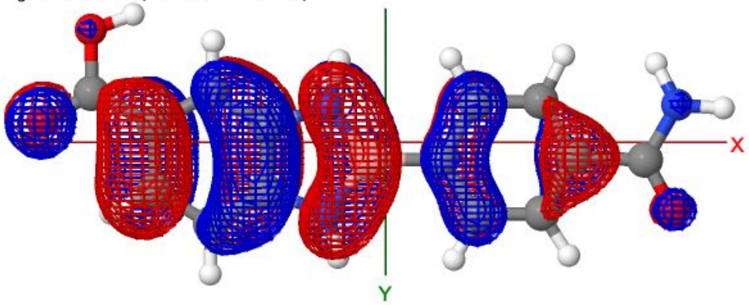

CA5

CA5

^{1}H NMR

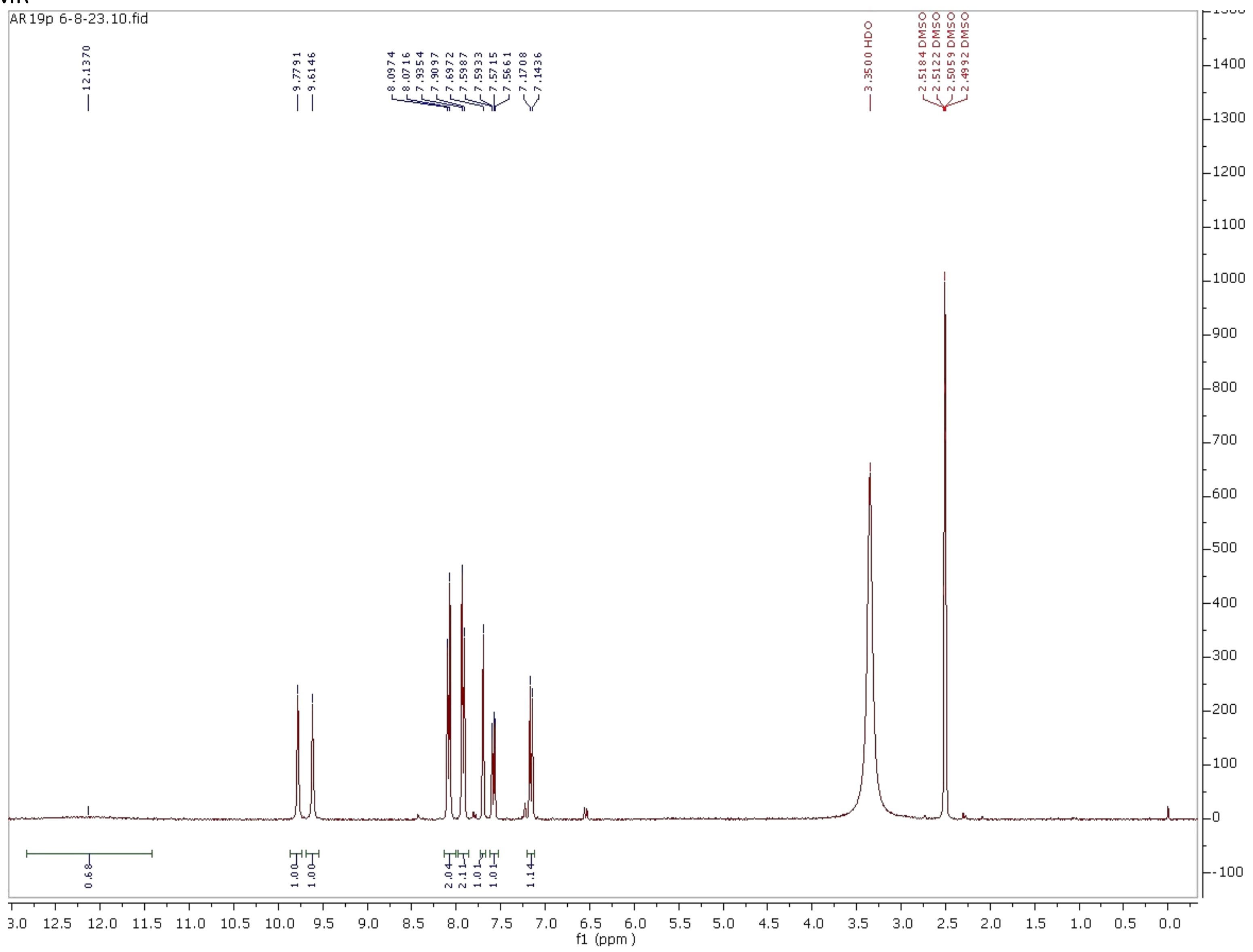

CA5

^{13}C NMR

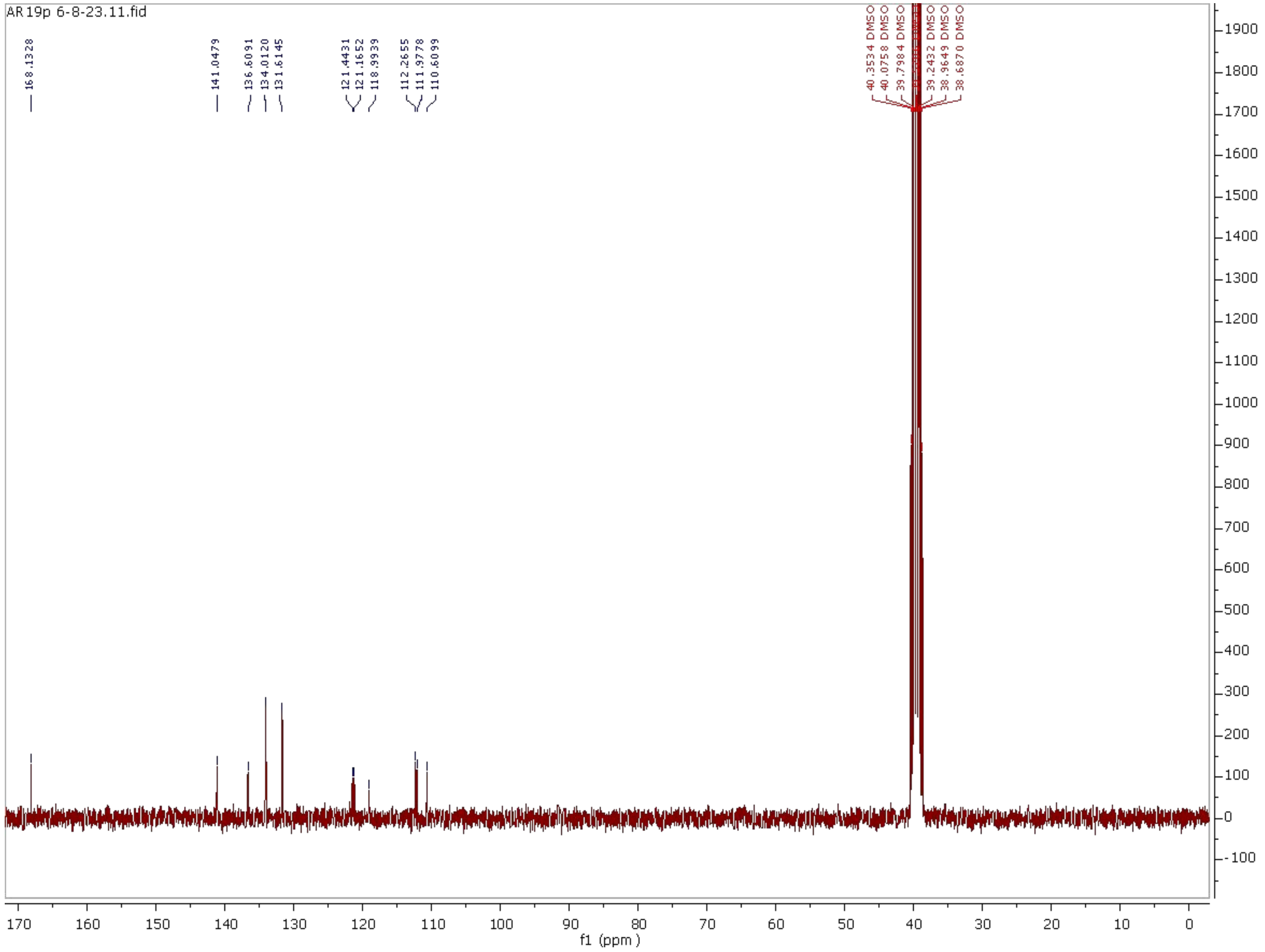
AR19p 6-8-23.11.fid
168.1328
141.0479
136.6091
134.0120
131.6145
121.4431
121.1652
118.9939
112.2655
111.9778
110.6099
40.3534 DMSO
40.0758 DMSO
39.7984 DMSO
39.2432 DMSO
38.9649 DMSO
38.6870 DMSO
f1 (ppm)

CA5

IR

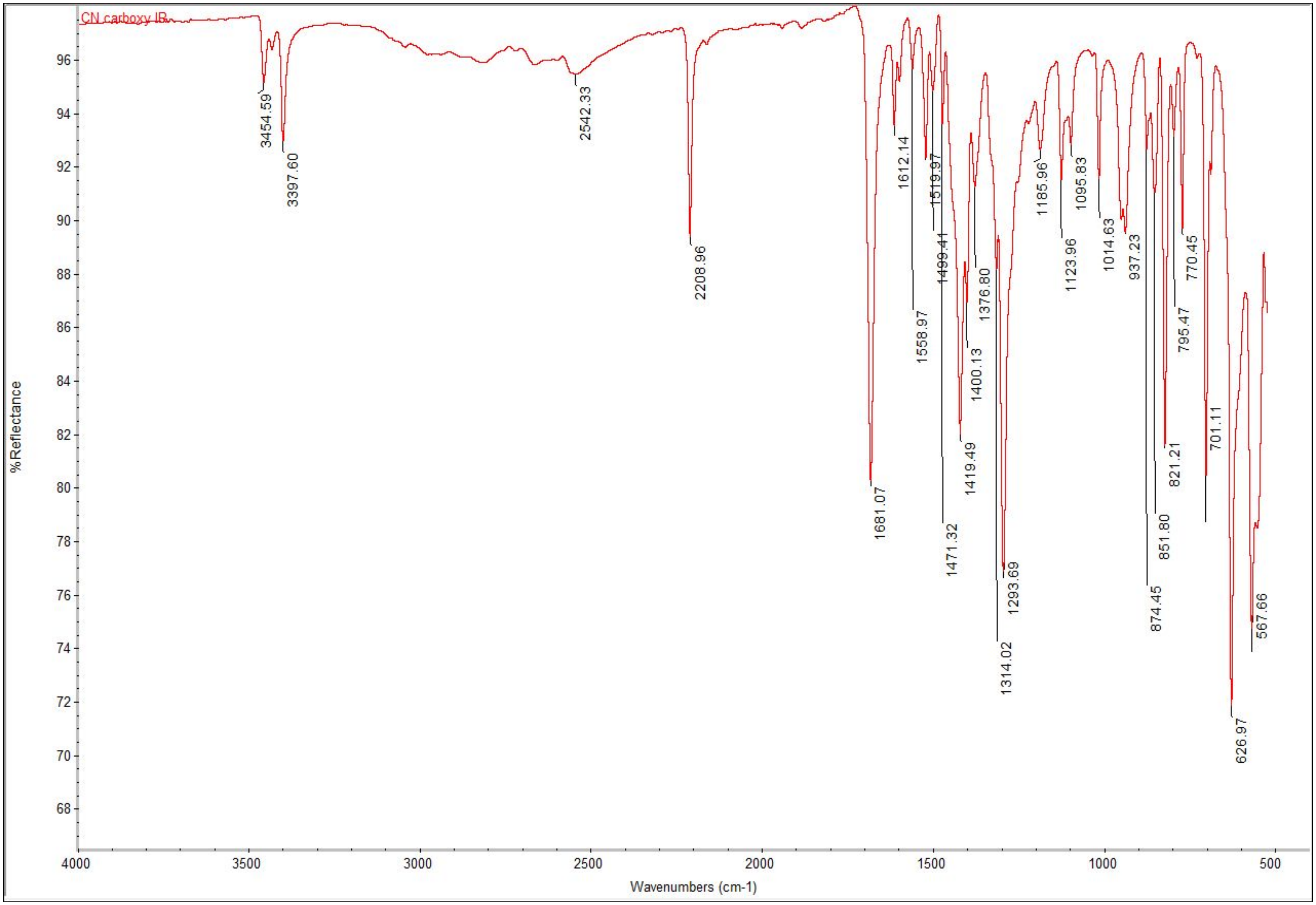
CN carboxy IR
%Reflectance
Wavenumbers (cm-1)
96
94
92
90
88
86
84
82
80
78
76
74
72
70
68
4000
3500
3000
2500
2000
1500
1000
500
3454.59
3397.60
2542.33
2208.96
1681.07
1612.14
1558.97
1519.97
1499.41
1471.32
1419.49
1400.13
1376.80
1314.02
1293.69
1185.96
1123.96
1095.83
1014.63
937.23
874.45
851.80
821.21
795.47
770.45
701.11
626.97
567.66

CA5

UV-vis

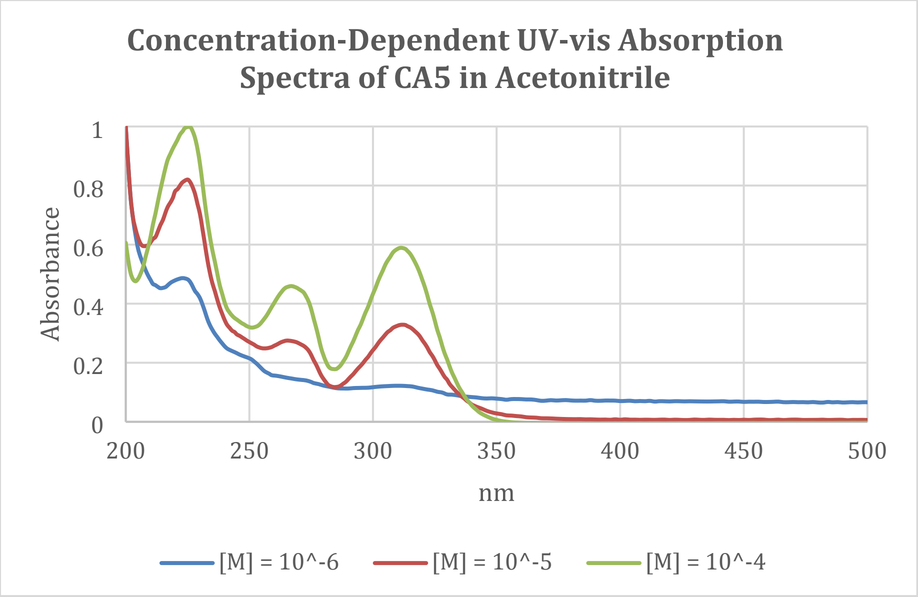

Fluorescence Emission

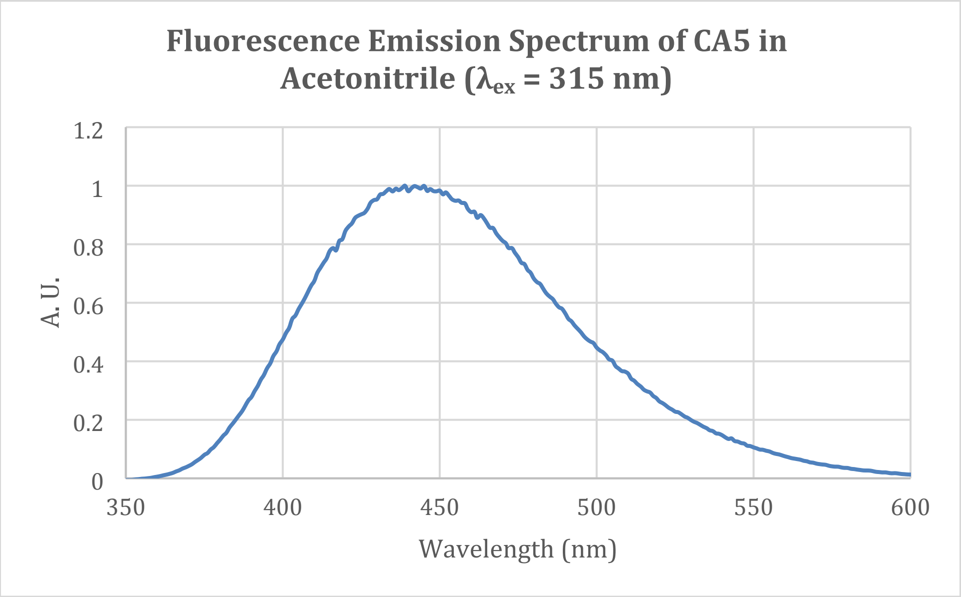

CA5

HRMS

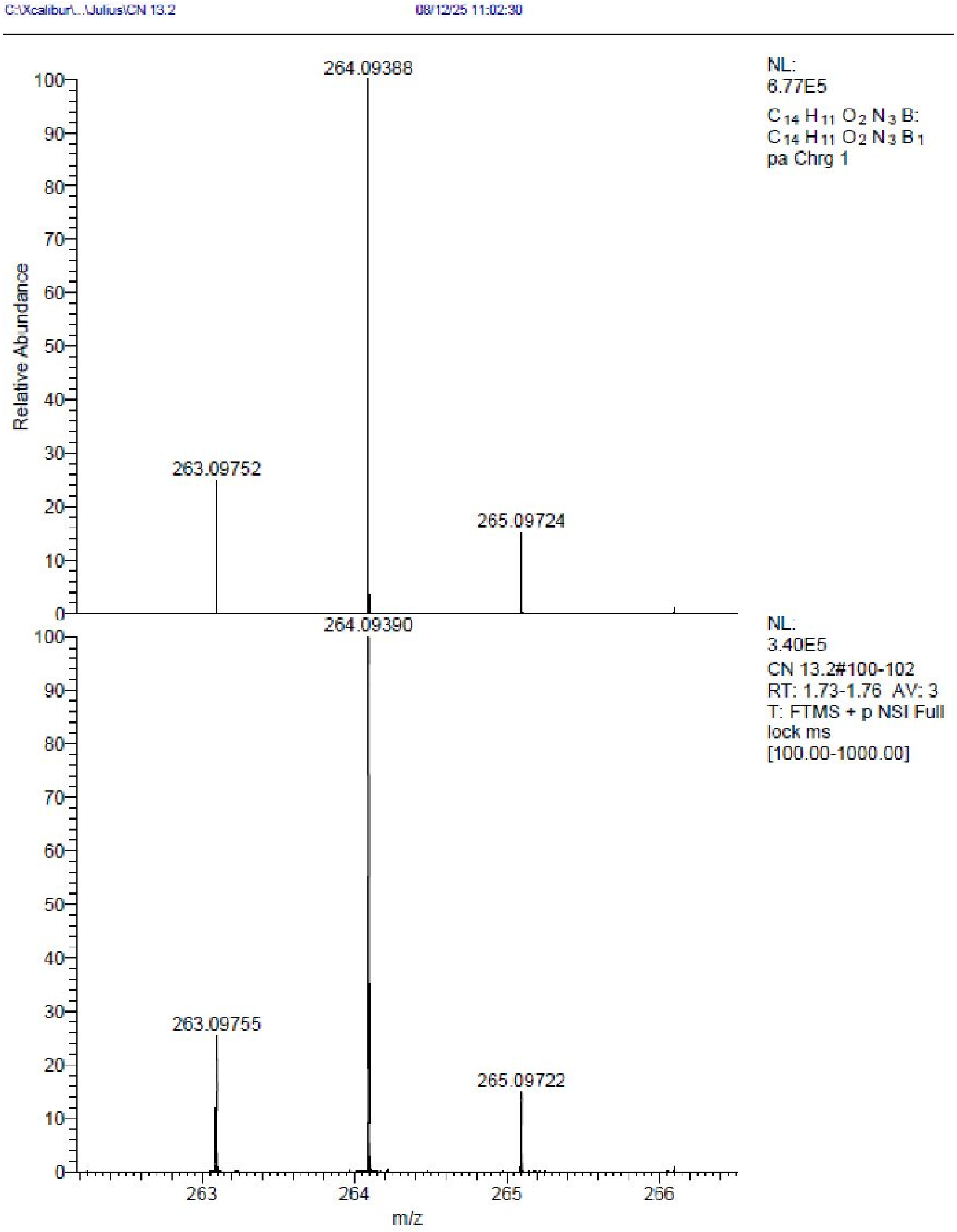

C:\Xcalibur\...\Julius\CN 13.2
08/12/25 11:02:30
NL:
6.77E5
C 14 H 11 O 2 N 3 B:
C 14 H 11 O 2 N 3 B 1
pa Chrg 1
264.09388
263.09752
265.09724
NL:
3.40E5
CN 13.2#100-102
RT: 1.73-1.76 AV: 3
T: FTMS + p NSI Full
lock ms
[100.00-1000.00]
264.09390
263.09755
265.09722
Relative Abundance
m/z

CA5

Computational Data

MO 69/78
Energy = -0.0725 a.u.
Symmetry = A''
Use the slider to adjust cutoff (start with 0.05)

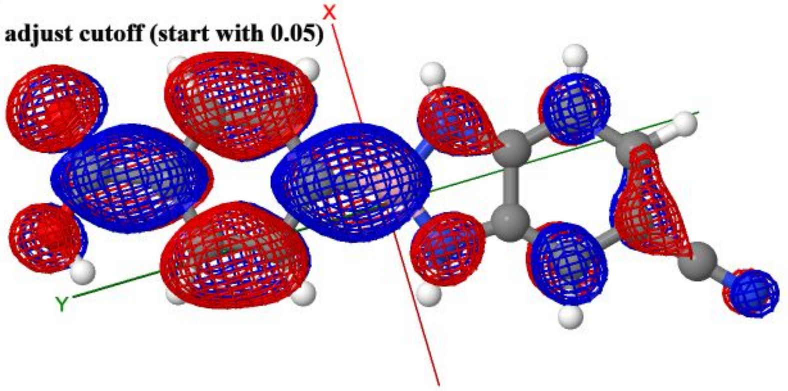

MO 68/78
Energy = -0.2238 a.u.
Symmetry = A''
Use the slider to adjust cutoff (start with 0.05)

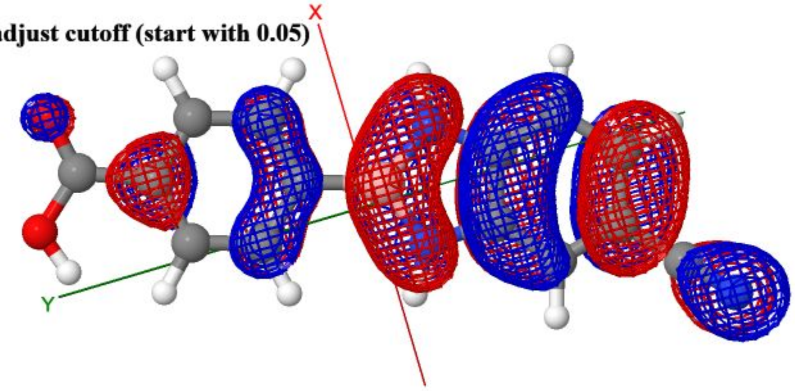

Am5

Am5

^{1}H NMR

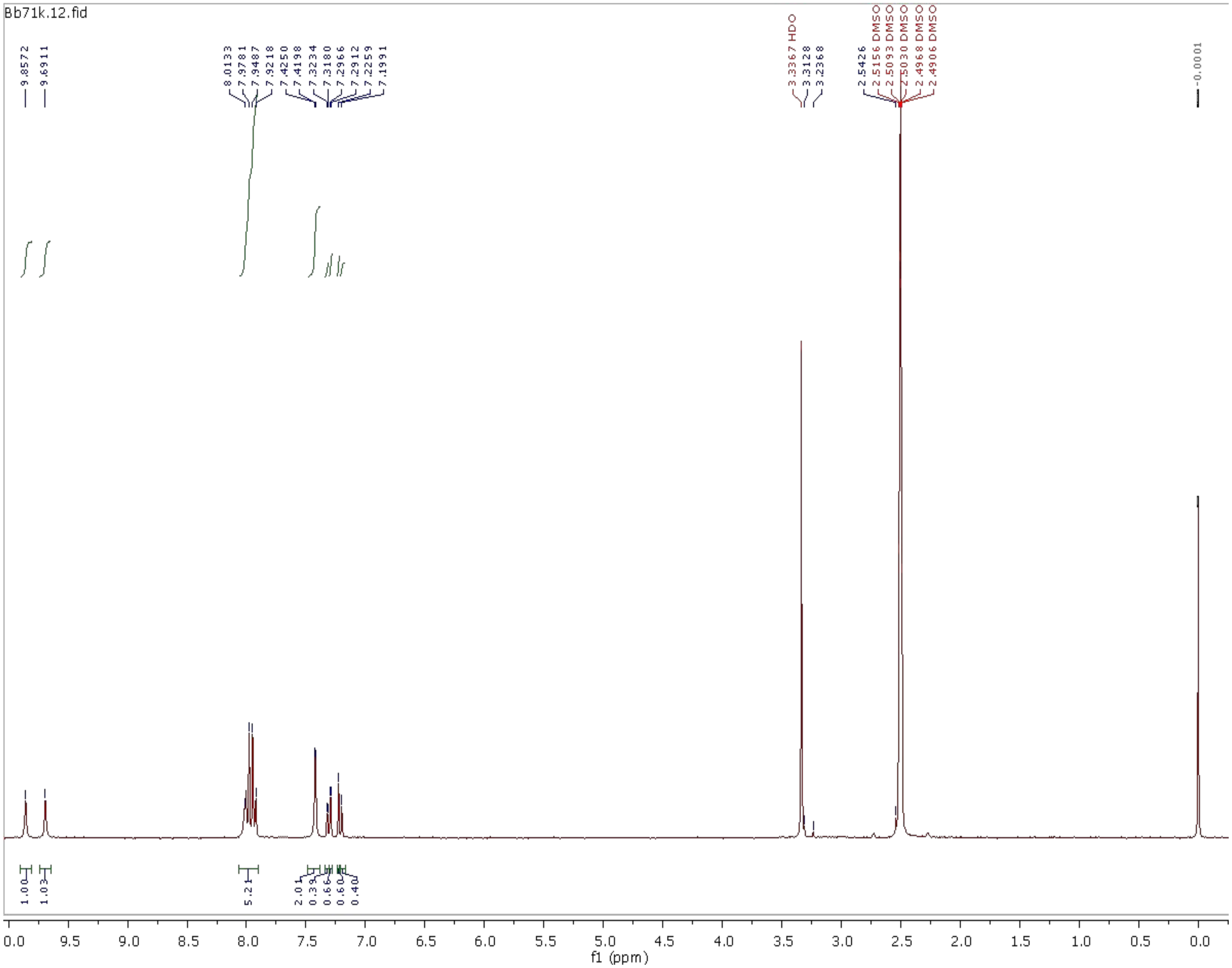

Am5

^{13}C NMR

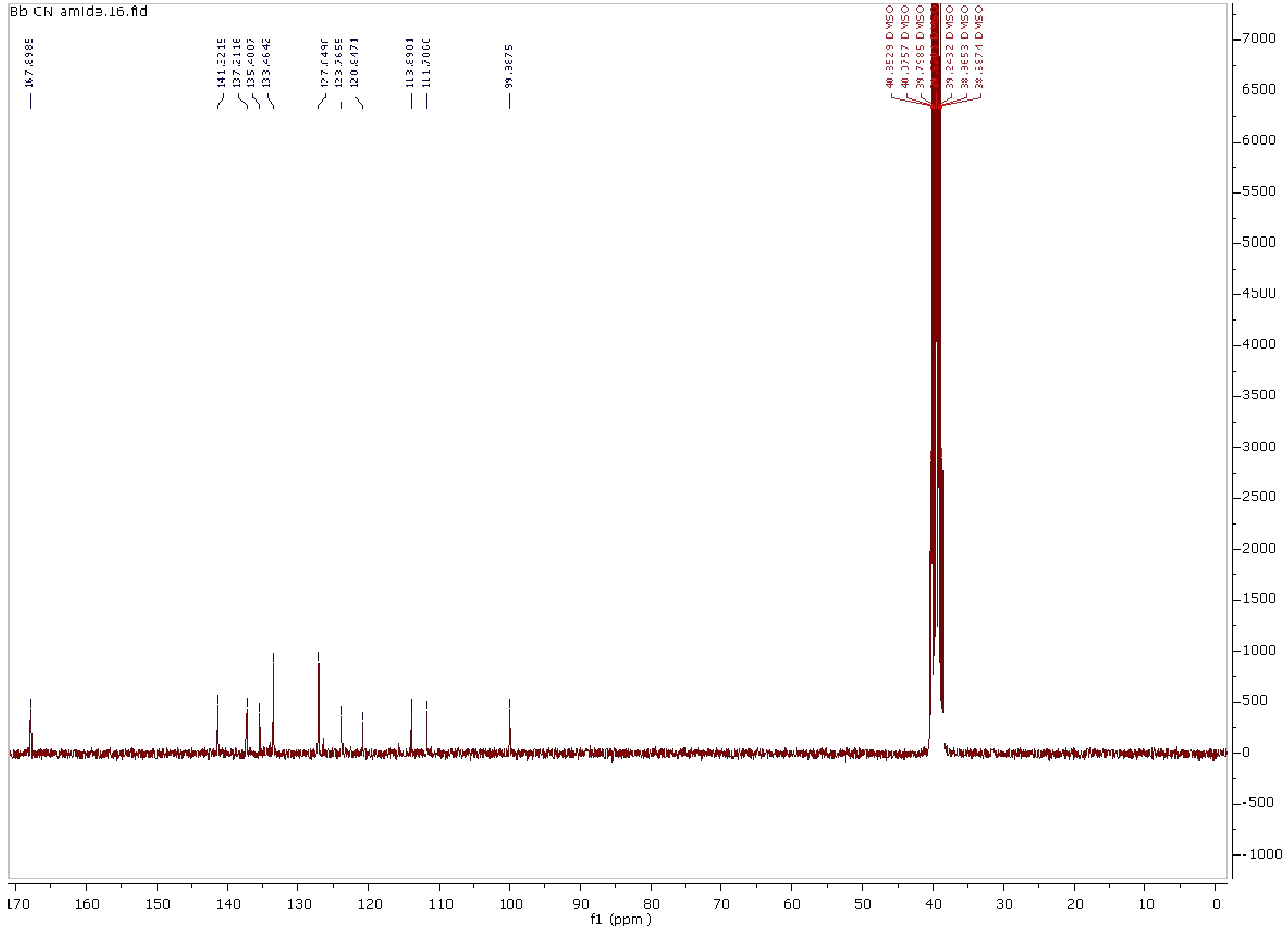
Bb CN amide.16.fid
167.8985
141.3215
137.2116
135.4007
133.4642
127.0490
123.7655
120.8471
113.8901
111.7066
99.9875
40.3529 DMSO
40.0757 DMSO
39.7985 DMSO
39.2432 DMSO
38.9653 DMSO
38.6874 DMSO
f1 (ppm)

Am5

^{11}B NMR

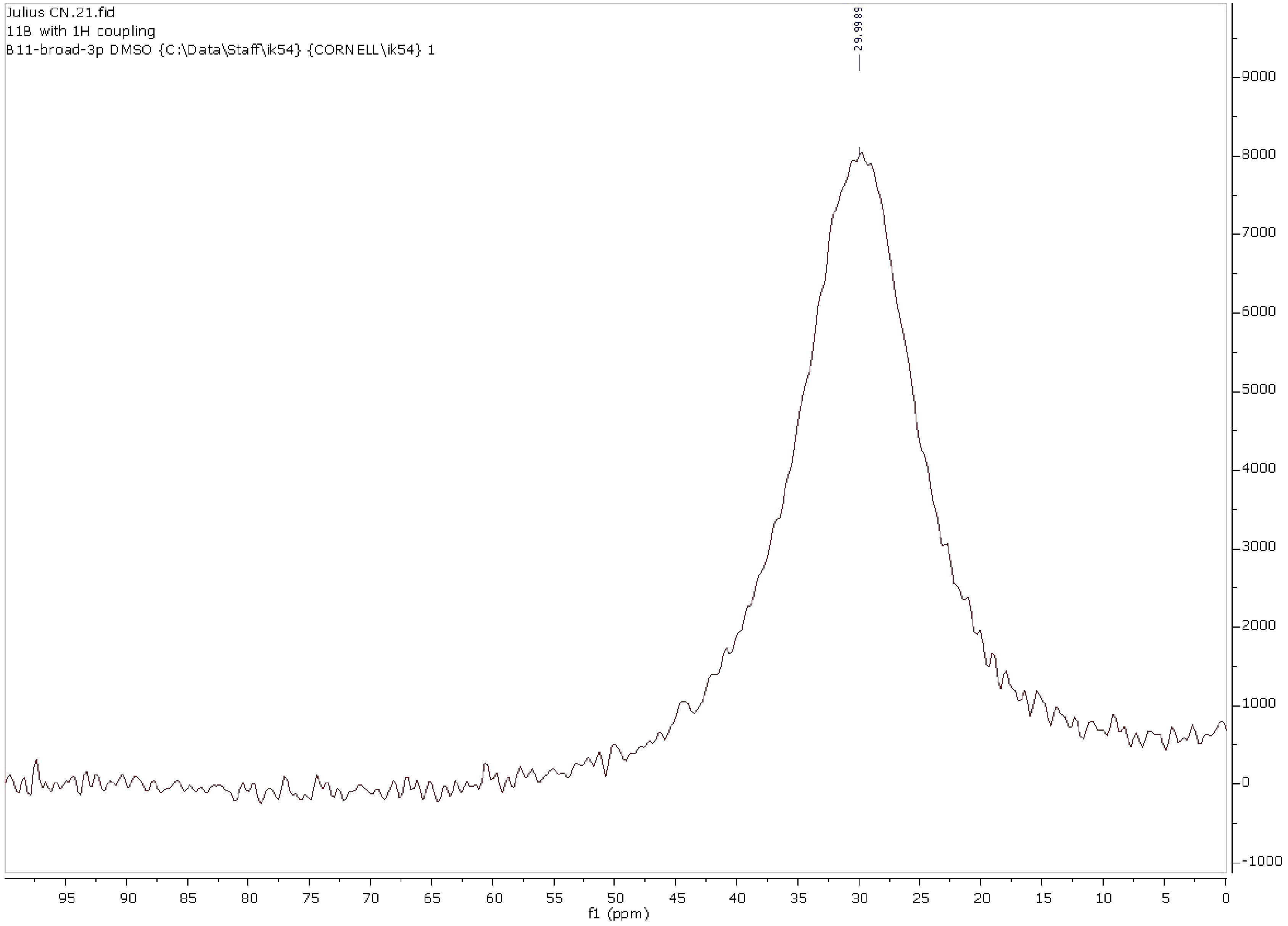
Julius CN.21.fid
11B with 1H coupling
B11-broad-3p DMSO {C:\Data\Staff\ik54} {CORNELL\ik54} 1
29.9989
9000
8000
7000
6000
5000
4000
3000
2000
1000
0
-1000
95
90
85
80
75
70
65
60
55
50
45
40
35
30
25
20
15
10
5
0
f1 (ppm)

Am5

IR

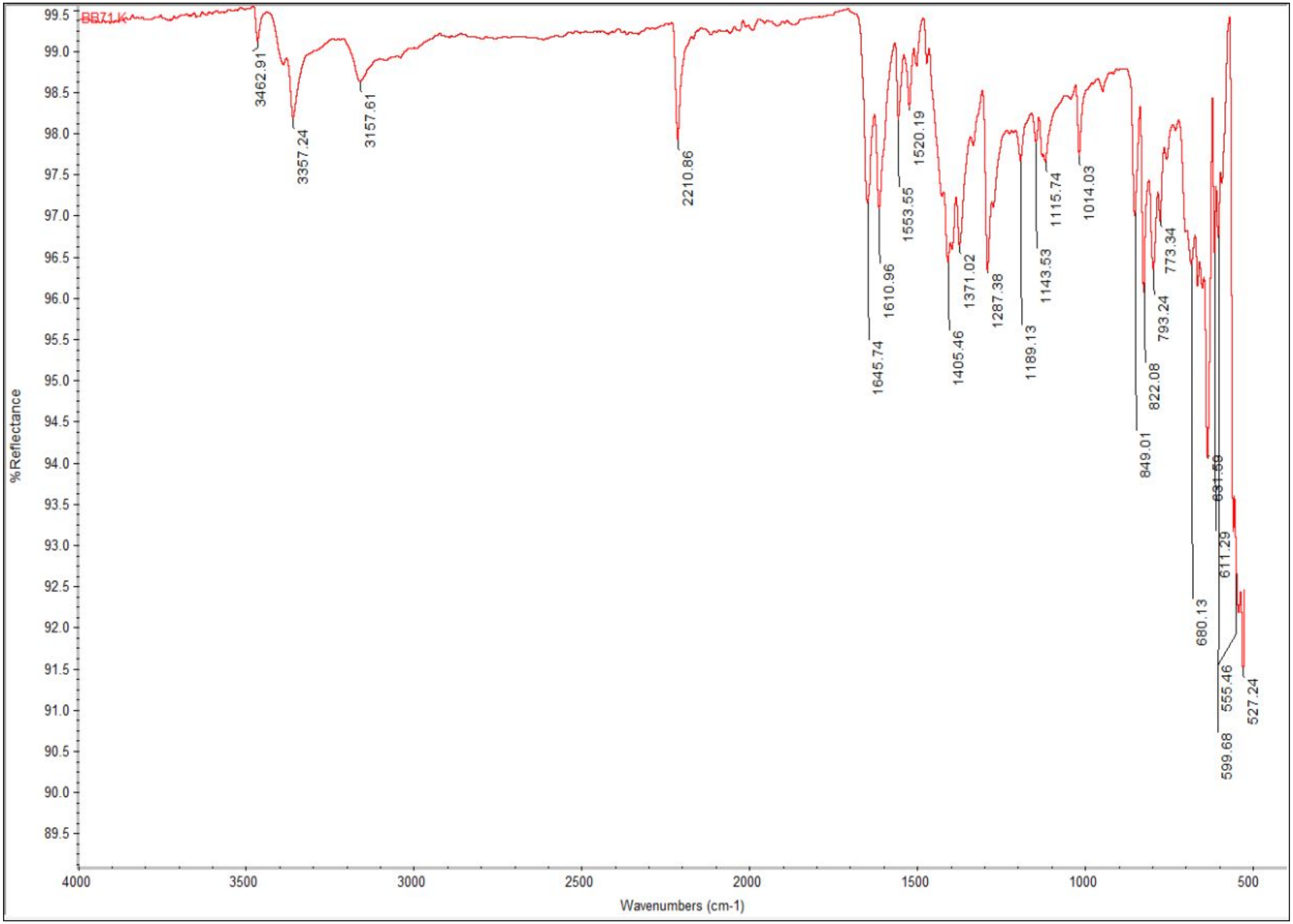

BB71.K
%Reflectance
Wavenumbers (cm-1)
3462.91
3357.24
3157.61
2210.86
1645.74
1610.96
1553.55
1520.19
1405.46
1371.02
1287.38
1189.13
1143.53
1115.74
1014.03
849.01
822.08
793.24
773.34
680.13
631.59
611.29
599.68
555.46
527.24

Am5

UV-vis

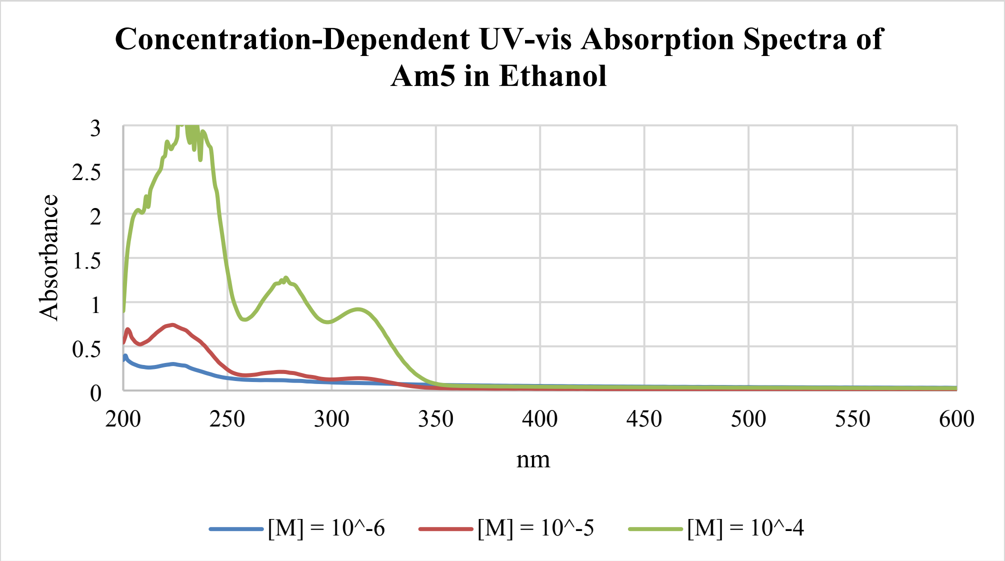

Concentration-Dependent UV-vis Absorption Spectra of Am5 in Ethanol
Absorbance
3
2.5
2
1.5
1
0.5
0
200
250
300
350
400
450
500
550
600
nm
[M] = 10^-6
[M] = 10^-5
[M] = 10^-4

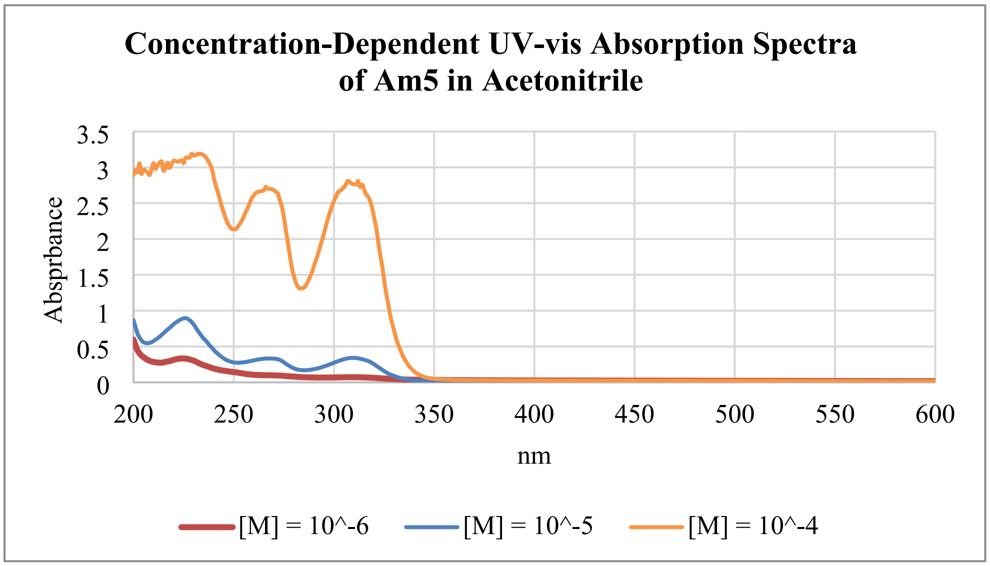

Concentration-Dependent UV-vis Absorption Spectra of Am5 in Acetonitrile
Absprbance
3.5
3
2.5
2
1.5
1
0.5
0
200
250
300
350
400
450
500
550
600
nm
[M] = 10^-6
[M] = 10^-5
[M] = 10^-4

Am5

Fluorescence Emission

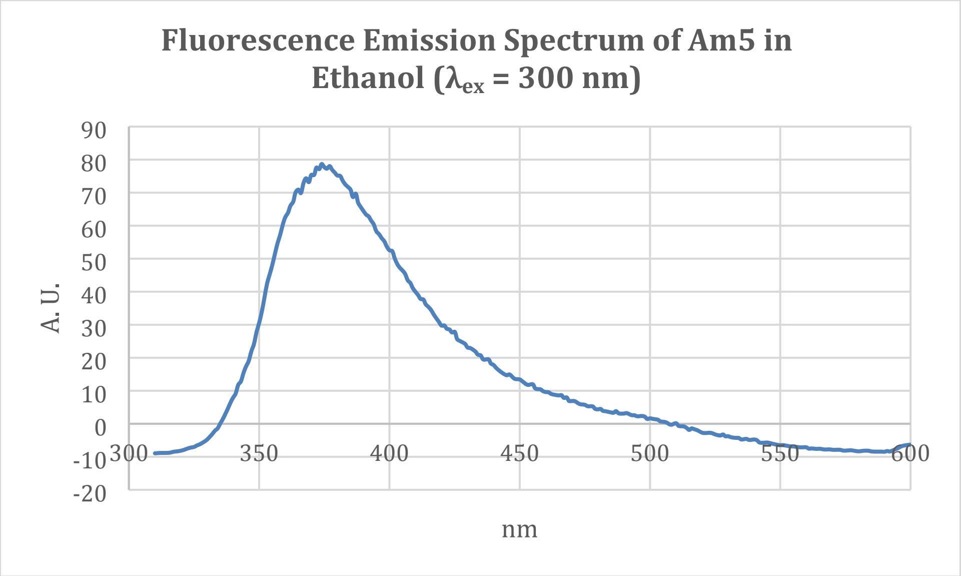

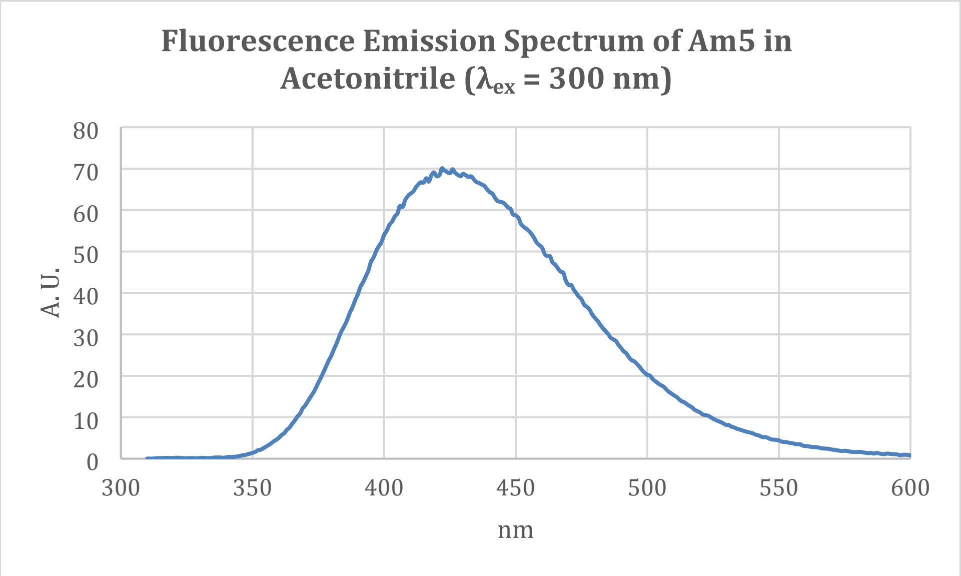

Am5

Fluorescence Excitation

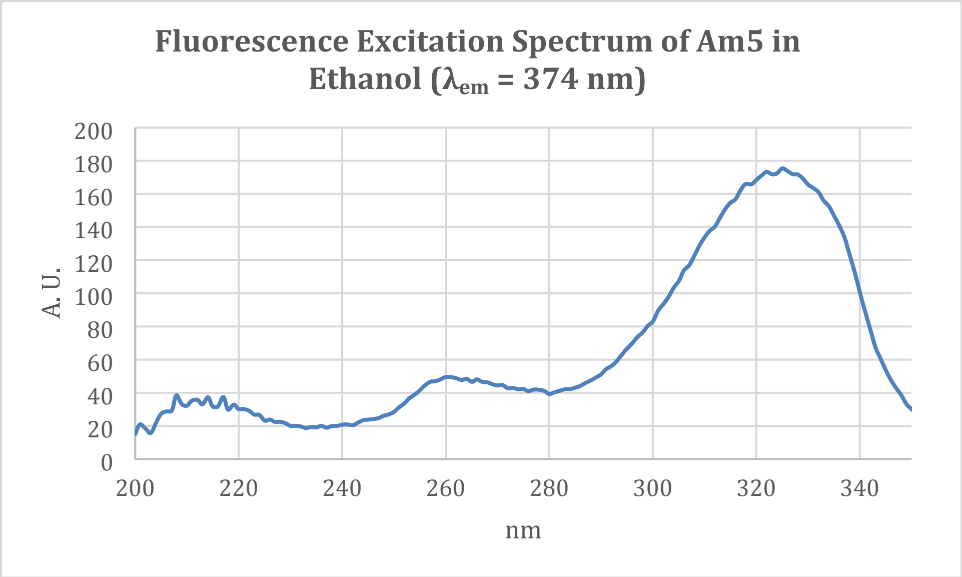

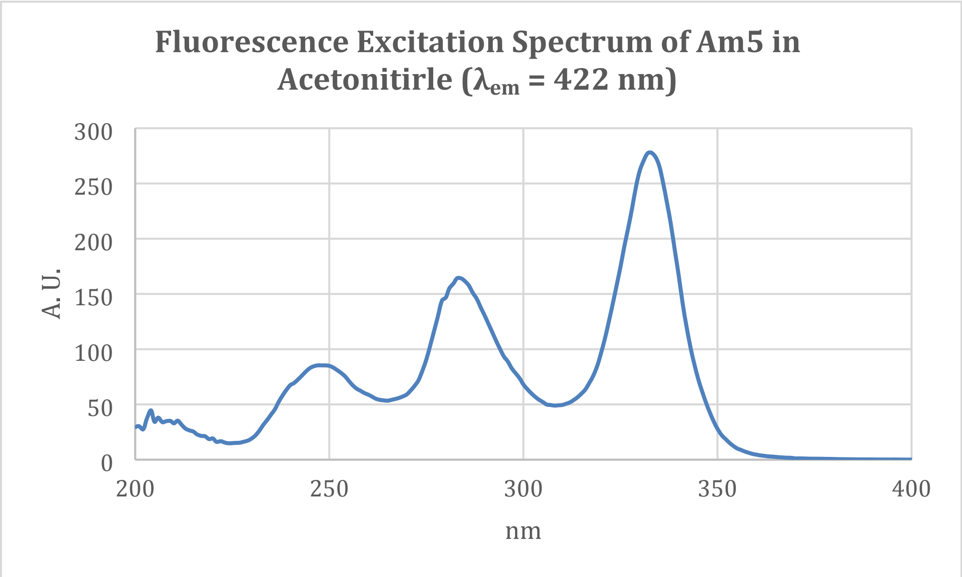

Am5

HRMS

C:\Xcalibur\...\Julian\CN-amide 07/29/25 10:26:47

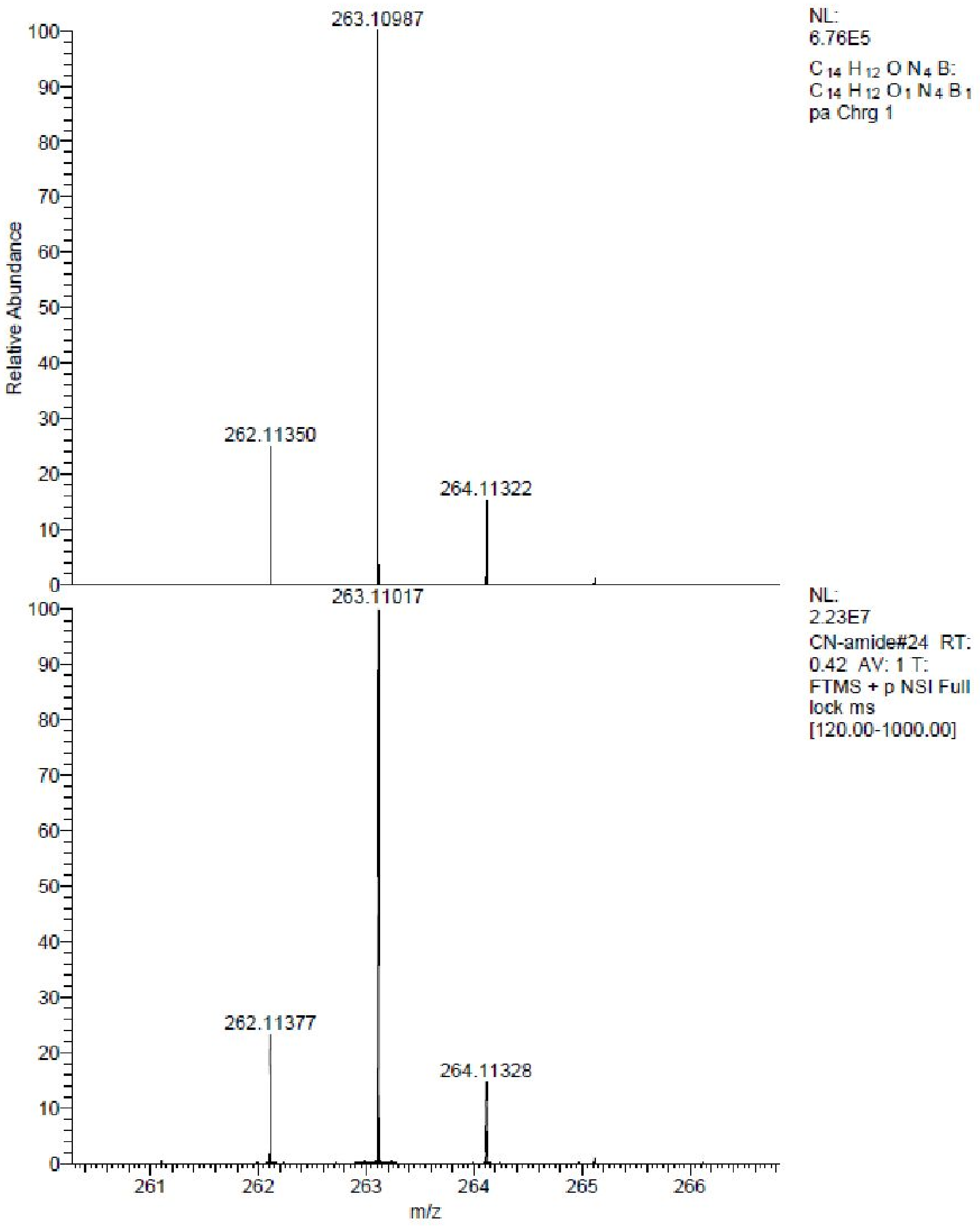

Am5

Computational Data

MO 69/78
Energy = -0.0591 a.u.
Symmetry = A''
Use the slider to adjust cutoff (start with 0.05)

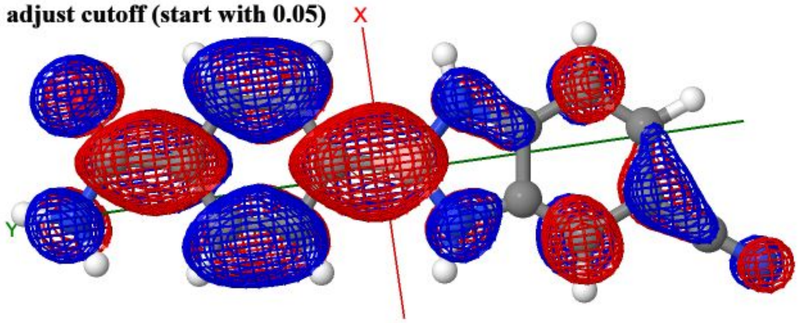

MO 68/78
Energy = -0.2175 a.u.
Symmetry = A''
Use the slider to adjust cutoff (start with 0.05)

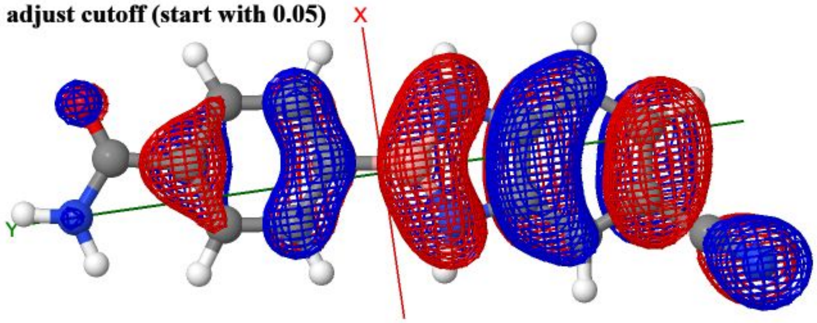

Am5

Optimized Cartesian Coordinates

Optimized Cartesian coordinates were extracted from the final converged geometry reported in the GAMESS output file under the section labeled ‘COORDINATES OF ALL ATOMS ARE (ANGS)’. Atomic positions correspond to DFT-optimized structures and are reported in Ångstroms.

00

C	-1.9207700000	-0.7399900000	0.0031400000
C	-1.9156800000	0.7240200000	0.0120600000
C	-3.0564900000	1.4209800000	0.0110200000
C	-4.3372300000	0.6703300000	-0.0002500000
C	-4.3407500000	-0.6765000000	-0.0089200000
C	-3.0641500000	-1.4321800000	-0.0070100000
N	-0.5987600000	-1.2618900000	0.0051500000
B	0.2783600000	-0.0162000000	0.0120100000
N	-0.5883100000	1.2374500000	0.0194700000
C	1.8525100000	-0.0266500000	0.0036800000
C	2.5346800000	-1.1868300000	-0.0017000000
C	4.0121800000	-1.1866700000	-0.0133200000
C	4.6829400000	-0.0296700000	-0.0191100000
C	3.9448100000	1.2495400000	-0.0129300000
C	2.6057100000	1.2529600000	-0.0016700000
H	-3.0497900000	2.5032700000	0.0171600000
H	-5.2729700000	1.2137200000	-0.0020400000
H	-5.2786800000	-1.2160100000	-0.0177400000
H	-3.0610600000	-2.5145100000	-0.0143500000
H	-0.3288600000	-2.2702500000	-0.0017800000
H	-0.3083000000	2.2425500000	0.0249600000
H	2.0340200000	-2.1463100000	0.0013200000
H	4.5484800000	-2.1271200000	-0.0178700000
H	5.7650000000	-0.0268600000	-0.0283900000
H	4.4923500000	2.1831300000	-0.0176700000
H	2.0874900000	2.2032700000	0.0023600000

Am5

Am1

C	-2.3112700000	-0.5584600000	0.0043900000
C	-2.2989600000	0.8583400000	0.0172500000
C	-3.4922500000	1.5661200000	0.0279200000
C	-4.7034600000	0.8592500000	0.0156400000
C	-4.7081000000	-0.5439300000	-0.0044700000
C	-3.5050200000	-1.2674600000	-0.0062300000
N	-0.9907900000	-0.9999900000	-0.0053100000
B	-0.0995700000	0.1341200000	0.0030400000
N	-0.9665300000	1.2792500000	0.0075300000
C	1.4577200000	0.1165400000	0.0221200000
C	2.1778500000	-1.0925700000	0.0319800000
C	3.5701200000	-1.1180200000	0.0253800000
C	4.3030300000	0.0772300000	-0.0047800000
C	3.6023900000	1.2915300000	-0.0067300000
C	2.2124500000	1.3057300000	0.0209400000
O	-5.8467500000	-1.3101300000	-0.0201800000
C	-7.0965000000	-0.6459200000	-0.0525400000
C	5.8059300000	0.1597600000	-0.0210300000
N	6.5019800000	-1.0220500000	0.0178600000
O	6.3893500000	1.2390800000	-0.0362800000
H	-3.4997800000	2.6532800000	0.0402400000
H	-5.6330900000	1.4152100000	0.0207500000
H	-3.5381300000	-2.3529600000	-0.0211200000
H	-0.7773400000	-1.9856000000	0.0094900000
H	-0.7346100000	2.2608300000	0.0266200000
H	1.6432800000	-2.0402600000	0.0436300000
H	4.0666800000	-2.0846000000	0.0533100000
H	4.1773000000	2.2112000000	-0.0267300000
H	1.7028000000	2.2674300000	0.0275200000
H	-7.1976000000	-0.0137500000	-0.9457900000
H	-7.2502900000	-0.0239300000	0.8412400000
H	-7.8558500000	-1.4297800000	-0.0842300000
H	6.0728900000	-1.9186800000	-0.1460500000
H	7.5036400000	-0.9526100000	-0.0858100000

Am5

CA1

C	-2.3308800000	-0.5658000000	-0.0365000000
C	-2.3158200000	0.8513000000	-0.0384700000
C	-3.5084500000	1.5616400000	-0.0338900000
C	-4.7194000000	0.8572400000	-0.0271300000
C	-4.7281800000	-0.5462000000	-0.0238900000
C	-3.5265800000	-1.2727100000	-0.0266300000
N	-1.0096800000	-1.0083500000	-0.0470500000
B	-0.1185100000	0.1242700000	-0.0529200000
N	-0.9827100000	1.2701800000	-0.0517100000
C	1.4407300000	0.1134000000	-0.0400400000
C	2.1686900000	-1.0837400000	0.0963200000
C	3.5596000000	-1.0933200000	0.1393900000
C	4.2821500000	0.1049500000	0.0441800000
C	3.5743600000	1.3093400000	-0.0892800000
C	2.1856000000	1.3062900000	-0.1390800000
O	-5.8656700000	-1.3128200000	-0.0199300000
C	-7.1174500000	-0.6500300000	-0.0443400000
C	5.7784900000	0.1853800000	0.1166800000
O	6.4724300000	-0.9683000000	-0.0896500000
O	6.3745000000	1.2081700000	0.3489200000
H	-3.5133500000	2.6489000000	-0.0394300000
H	-5.6494900000	1.4141200000	-0.0287800000
H	-3.5599400000	-2.3579400000	-0.0219900000
H	-0.7988100000	-1.9955400000	-0.0610900000
H	-0.7493700000	2.2523600000	-0.0295800000
H	1.6419200000	-2.0311700000	0.1857400000
H	4.0704500000	-2.0434400000	0.2904700000
H	4.1361300000	2.2364200000	-0.1470600000
H	1.6667200000	2.2557500000	-0.2523000000
H	-7.2366000000	-0.0352000000	-0.9465300000
H	-7.2587500000	-0.0118200000	0.8414200000
H	-7.8765000000	-1.4343600000	-0.0348900000
H	5.8801300000	-1.6730900000	-0.3919100000

Am5

Am2

C	-2.8300000000	0.0500000000	0.0000000000
C	-2.5200000000	-1.3800000000	0.0000000000
C	-3.4900000000	-2.3000000000	0.0000000000
C	-4.9000000000	-1.8200000000	0.0000000000
C	-5.1900000000	-0.5000000000	0.0000000000
C	-4.0900000000	0.4900000000	0.0000000000
N	-1.6400000000	0.8300000000	0.0000000000
B	-0.5300000000	-0.2000000000	0.0000000000
N	-1.1200000000	-1.6100000000	0.0000000000
C	1.0100000000	0.1400000000	0.0000000000
C	1.4300000000	1.4100000000	0.0000000000
C	2.8700000000	1.7300000000	0.0000000000
C	3.8000000000	0.7600000000	0.0000000000
C	3.3300000000	-0.6600000000	0.0000000000
C	2.0200000000	-0.9500000000	0.0000000000
C	-6.6100000000	-0.0100000000	0.0000000000
C	5.2600000000	1.0400000000	0.0000000000
N	5.8100000000	2.3500000000	0.0000000000
O	6.0400000000	0.1000000000	0.0000000000
H	-3.2700000000	-3.3500000000	0.0000000000
H	-5.6900000000	-2.5700000000	0.0000000000
H	-4.2900000000	1.5600000000	0.0000000000
H	-1.5900000000	1.8800000000	0.0000000000
H	-0.6300000000	-2.5400000000	0.0000000000
H	0.7300000000	2.2400000000	0.0000000000
H	3.1200000000	2.7800000000	0.0000000000
H	4.0400000000	-1.4700000000	0.0000000000
H	1.7200000000	-1.9900000000	0.0000000000
H	-6.7800000000	0.6100000000	0.9000000000
H	-6.7800000000	0.6100000000	-0.9000000000
H	-7.3400000000	-0.8400000000	0.0000000000
H	5.2400000000	3.2000000000	0.0000000000
H	6.8300000000	2.4800000000	0.0000000000

Am5

CA2

C	-2.7559800000	-0.6317800000	-0.0395500000
C	-2.7588800000	0.8336300000	-0.0378800000
C	-3.9056200000	1.5195900000	-0.0298100000
C	-5.1794200000	0.7594400000	-0.0258700000
C	-5.1802100000	-0.5950000000	-0.0308700000
C	-3.8895300000	-1.3303600000	-0.0376400000
N	-1.4337500000	-1.1549900000	-0.0411600000
B	-0.5710300000	0.1026600000	-0.0438000000
N	-1.4376100000	1.3549800000	-0.0442600000
C	0.9945700000	0.0897900000	-0.0280200000
C	1.6613500000	-1.0716600000	0.0282400000
C	3.1343200000	-1.0925900000	0.0675100000
C	3.8376900000	0.0574400000	0.0303300000
C	3.0903000000	1.3358700000	-0.0476500000
C	1.7535100000	1.3615800000	-0.0677900000
C	-6.4534000000	-1.3839800000	-0.0302900000
C	5.3240400000	0.0796400000	0.0777200000
O	6.0550900000	-1.0598700000	0.3212700000
O	5.9259000000	1.1393900000	-0.0516300000
H	-3.9068200000	2.6046900000	-0.0271800000
H	-6.1063700000	1.3116100000	-0.0196300000
H	-3.8681400000	-2.4174000000	-0.0386500000
H	-1.1664500000	-2.1628600000	-0.0394400000
H	-1.1600400000	2.3654700000	-0.0401600000
H	1.1533800000	-2.0308800000	0.0592800000
H	3.6049100000	-2.0607900000	0.1270400000
H	3.6226400000	2.2801600000	-0.0770500000
H	1.2443200000	2.3135000000	-0.1152000000
H	-6.4864400000	-2.0324800000	0.8720400000
H	-6.4946500000	-2.0226500000	-0.9357000000
H	-7.3452900000	-0.7282400000	-0.0204700000
H	5.7476600000	-1.7475000000	-0.3246500000

Am5

Am3

C	-3.1342200000	-0.7033100000	-0.0039000000
C	-3.1287400000	0.7119600000	0.0012900000
C	-4.3235300000	1.4282200000	0.0085000000
C	-5.5251300000	0.7125200000	0.0009000000
C	-5.5298100000	-0.6880600000	-0.0094600000
C	-4.3330700000	-1.4133700000	-0.0104800000
N	-1.8104700000	-1.1409200000	-0.0039000000
B	-0.9244300000	-0.0052200000	-0.0031800000
N	-1.8006900000	1.1368300000	-0.0013900000
C	0.6345000000	-0.0110300000	0.0041800000
C	1.3642300000	-1.2146900000	0.0002000000
C	2.7568300000	-1.2272900000	-0.0022100000
C	3.4785400000	-0.0247400000	0.0142600000
C	2.7677300000	1.1830000000	0.0258200000
C	1.3772900000	1.1853600000	0.0149700000
C	4.9813000000	0.0739900000	0.0049300000
N	5.6848100000	-1.0995400000	-0.0801900000
O	5.5562100000	1.1548900000	0.0310700000
H	-4.3213500000	2.5155200000	0.0180300000
H	-6.4674400000	1.2531100000	0.0067000000
H	-6.4755600000	-1.2232700000	-0.0120500000
H	-4.3392200000	-2.5005200000	-0.0135700000
H	-1.5923900000	-2.1248900000	-0.0252100000
H	-1.5766000000	2.1211000000	0.0153800000
H	0.8391900000	-2.1671400000	-0.0098000000
H	3.2624200000	-2.1897300000	-0.0283100000
H	3.3352400000	2.1078800000	0.0336200000
H	0.8620100000	2.1434100000	0.0130200000
H	5.2733000000	-2.0008400000	0.1296800000
H	6.6890900000	-1.0228200000	0.0175900000

Am5

CA3

C	-3.1441400000	-0.7174400000	-0.0419400000
C	-3.1481000000	0.7495600000	-0.0450900000
C	-4.2912300000	1.4369600000	-0.0447000000
C	-5.5654100000	0.6767800000	-0.0443500000
C	-5.5613400000	-0.6706500000	-0.0443400000
C	-4.2829400000	-1.4173600000	-0.0425200000
N	-1.8219100000	-1.2339000000	-0.0367700000
B	-0.9575200000	0.0225400000	-0.0412100000
N	-1.8246700000	1.2769600000	-0.0480000000
C	0.6096200000	0.0216500000	-0.0247800000
C	1.2759800000	-1.1452500000	0.0401100000
C	2.7472300000	-1.1615600000	0.0783800000
C	3.4549500000	-0.0202100000	0.0322800000
C	2.7126100000	1.2672800000	-0.0557300000
C	1.3743700000	1.2873200000	-0.0738300000
C	4.9377700000	-0.0263400000	0.0812300000
O	5.6440900000	-1.1820000000	0.3362700000
O	5.5627300000	1.0171400000	-0.0565700000
H	-4.2947300000	2.5221300000	-0.0457700000
H	-6.5020200000	1.2101000000	-0.0450400000
H	-6.4956300000	-1.2100300000	-0.0445100000
H	-4.2728400000	-2.5028400000	-0.0404700000
H	-1.5504000000	-2.2463200000	-0.0319100000
H	-1.5498400000	2.2821100000	-0.0461600000
H	0.7692200000	-2.0985500000	0.0777700000
H	3.2190300000	-2.1352600000	0.1456400000
H	3.2503000000	2.2016400000	-0.0928600000
H	0.8637300000	2.2438400000	-0.1282600000
H	5.3806300000	-1.8333400000	-0.3615500000

Am5

Am4

C	-1.9806000000	-0.4269700000	-0.0224000000
C	-1.9937300000	1.0349000000	0.0489800000
C	-3.1405700000	1.7098600000	0.0887200000
C	-4.4072900000	0.9397100000	0.0573300000
C	-4.4122900000	-0.4134500000	-0.0244200000
C	-3.1113600000	-1.1347300000	-0.0665700000
N	-0.6630700000	-0.9375500000	-0.0344100000
B	0.1935200000	0.3195400000	0.0192800000
N	-0.6756500000	1.5690100000	0.0661300000
C	1.7504500000	0.2924400000	0.0219900000
C	2.4062900000	-0.8803900000	0.0106300000
C	3.8728300000	-0.9112000000	0.0117200000
C	4.5862400000	0.2333200000	0.0214300000
C	3.8462300000	1.5295600000	0.0335100000
C	2.5128800000	1.5580300000	0.0350200000
C	-5.6925400000	-1.1670700000	-0.0698500000
O	-6.7642000000	-0.5979700000	0.0594400000
O	-5.6903400000	-2.5250400000	-0.3038700000
C	6.0734000000	0.2456800000	0.0199100000
N	6.8621800000	-0.9348000000	0.0163800000
O	6.6579800000	1.3221700000	0.0216200000
H	-3.1519500000	2.7943000000	0.1415100000
H	-5.3279600000	1.4988900000	0.0903000000
H	-3.0494400000	-2.2154200000	-0.1221000000
H	-0.3934900000	-1.9428200000	-0.0727600000
H	-0.4037600000	2.5797800000	0.1064200000
H	1.8948600000	-1.8326700000	0.0013700000
H	4.3254100000	-1.8869600000	0.0027400000
H	4.3853400000	2.4663800000	0.0411600000
H	2.0050500000	2.5181200000	0.0433300000
H	-5.1549400000	-2.9345700000	0.4210600000
H	6.4700400000	-1.8861300000	0.0167600000
H	7.8853400000	-0.8638500000	0.0150700000

Am5

CA4

C	-2.0129500000	-0.4192200000	-0.0035400000
C	-2.0161900000	1.0360500000	0.1251200000
C	-3.1555900000	1.7220000000	0.1826300000
C	-4.4296000000	0.9661000000	0.1122800000
C	-4.4420200000	-0.3830600000	-0.0108200000
C	-3.1507400000	-1.1174700000	-0.0718300000
N	-0.6947600000	-0.9410700000	-0.0446900000
B	0.1724500000	0.3099500000	0.0631000000
N	-0.6911400000	1.5558300000	0.1726000000
C	1.7417900000	0.2880200000	0.0429500000
C	2.4019100000	-0.8714700000	-0.0850100000
C	3.8749200000	-0.8970700000	-0.1207300000
C	4.5792100000	0.2443400000	-0.0205100000
C	3.8487300000	1.5249100000	0.1300900000
C	2.5092700000	1.5524100000	0.1580800000
C	6.0603000000	0.2180400000	-0.0654200000
O	6.7378400000	-0.9584900000	-0.2666900000
O	6.6975800000	1.2493800000	0.0563700000
C	-5.7164300000	-1.1379700000	-0.0865600000
O	-6.7911100000	-0.5672200000	-0.0158400000
O	-5.7027200000	-2.5016000000	-0.2538000000
H	-3.1574800000	2.8025200000	0.2771100000
H	-5.3469800000	1.5313000000	0.1591000000
H	-3.1132300000	-2.1925400000	-0.1678400000
H	-0.4284000000	-1.9434000000	-0.1325400000
H	-0.4142700000	2.5561900000	0.2552000000
H	1.8907500000	-1.8247600000	-0.1737900000
H	4.3582400000	-1.8560700000	-0.2319200000
H	4.3921000000	2.4587100000	0.2145900000
H	2.0051500000	2.5010000000	0.2646300000
H	6.7759400000	-1.3924600000	0.6272100000
H	-5.6015800000	-2.8723200000	0.6573300000

Am5

CN4

C	-1.5357300000	-0.4327900000	-0.0231000000
C	-1.5407600000	1.0291900000	0.0726700000
C	-2.6854600000	1.7103300000	0.1181800000
C	-3.9548000000	0.9448600000	0.0647600000
C	-3.9630400000	-0.4069600000	-0.0371400000
C	-2.6695000000	-1.1351100000	-0.0825200000
N	-0.2190700000	-0.9479100000	-0.0418000000
B	0.6426300000	0.3003100000	0.0337600000
N	-0.2180600000	1.5580200000	0.1044600000
C	2.2017400000	0.2826700000	0.0320900000
C	2.8706400000	-0.8868300000	0.0048400000
C	4.3425400000	-0.8866100000	0.0019200000
C	5.0083600000	0.2688200000	0.0229100000
C	4.2835300000	1.5556300000	0.0512100000
C	2.9476200000	1.5610000000	0.0572100000
C	-5.2398400000	-1.1588400000	-0.1014100000
O	-6.3141600000	-0.5912400000	0.0013000000
O	-5.2285000000	-2.5172600000	-0.3071800000
C	6.4382500000	0.2727600000	0.0180900000
N	7.5898900000	0.3003700000	0.0277300000
H	-2.6933700000	2.7928500000	0.1892700000
H	-4.8744200000	1.5066600000	0.1007700000
H	-2.6254800000	-2.2165900000	-0.1551900000
H	0.0515500000	-1.9572300000	-0.1007900000
H	0.0538100000	2.5613300000	0.1634000000
H	2.3704200000	-1.8434800000	-0.0135900000
H	4.8637600000	-1.8391300000	-0.0183300000
H	4.8254100000	2.4894300000	0.0672200000
H	2.4319100000	2.5158300000	0.0774100000
H	-4.9254800000	-2.9112900000	0.5483800000

Am5

Am5

C	-0.8900000000	-2.3100000000	0.0000000000
C	0.5600000000	-2.5200000000	0.0000000000
C	1.0800000000	-3.7500000000	0.0000000000
C	0.1400000000	-4.9000000000	0.0000000000
C	-1.1900000000	-4.6900000000	0.0000000000
C	-1.7500000000	-3.3200000000	0.0000000000
N	-1.2200000000	-0.9300000000	0.0000000000
B	0.1400000000	-0.2600000000	0.0000000000
N	1.2600000000	-1.2900000000	0.0000000000
C	0.3500000000	1.2800000000	0.0000000000
C	-0.7100000000	2.1000000000	0.0000000000
C	-0.5100000000	3.5500000000	0.0000000000
C	0.7300000000	4.0800000000	0.0000000000
C	1.9000000000	3.1600000000	0.0000000000
C	1.7200000000	1.8300000000	0.0000000000
C	-2.0900000000	-5.8100000000	0.0000000000
C	0.9700000000	5.5500000000	0.0000000000
N	-0.0800000000	6.5000000000	0.0000000000
O	2.1200000000	5.9600000000	0.0000000000
N	-2.8600000000	-6.6600000000	0.0000000000
H	2.1500000000	-3.9100000000	0.0000000000
H	0.5500000000	-5.9000000000	0.0000000000
H	-2.8200000000	-3.1500000000	0.0000000000
H	-2.1800000000	-0.5200000000	0.0000000000
H	2.3000000000	-1.1600000000	0.0000000000
H	-1.7300000000	1.7400000000	0.0000000000
H	-1.4000000000	4.1500000000	0.0000000000
H	2.9100000000	3.5400000000	0.0000000000
H	2.5900000000	1.1900000000	0.0000000000
H	-1.0800000000	6.2500000000	0.0000000000
H	0.1400000000	7.5000000000	0.0000000000

Am5

CA5

C	-1.2620000000	-2.1750000000	0.0000000000
C	0.0920000000	-2.6020000000	0.0000000000
C	0.4170000000	-3.9500000000	0.0000000000
C	-0.6250000000	-4.8870000000	0.0000000000
C	-1.9640000000	-4.4610000000	0.0000000000
C	-2.3020000000	-3.0870000000	0.0000000000
N	-1.2650000000	-0.7850000000	0.0000000000
B	0.0730000000	-0.2840000000	0.0000000000
N	0.9080000000	-1.4580000000	0.0000000000
C	0.5500000000	1.2160000000	0.0000000000
C	-0.3760000000	2.2650000000	0.0000000000
C	0.0450000000	3.5940000000	0.0000000000
C	1.3940000000	3.9270000000	0.0000000000
C	2.3450000000	2.8690000000	0.0000000000
C	1.9150000000	1.5580000000	0.0000000000
C	-3.0110000000	-5.4320000000	0.0000000000
C	1.9360000000	5.3170000000	0.0000000000
O	1.0520000000	6.3470000000	0.0000000000
O	3.1350000000	5.5500000000	0.0000000000
N	-3.8640000000	-6.2230000000	0.0000000000
H	1.4590000000	-4.2720000000	0.0000000000
H	-0.4070000000	-5.9420000000	0.0000000000
H	-3.3420000000	-2.7810000000	0.0000000000
H	-2.1440000000	-0.2740000000	0.0000000000
H	1.8970000000	-1.5550000000	0.0000000000
H	-1.4340000000	2.0570000000	0.0000000000
H	-0.7340000000	4.3520000000	0.0000000000
H	3.3870000000	3.1380000000	0.0000000000
H	2.6650000000	0.7690000000	0.0000000000
H	0.1440000000	6.0210000000	0.0000000000

Am5

Am4 Dimer

C	6.0	4.7309300000	-1.1116700000	0.9694700000
C	6.0	4.7922200000	-0.8318400000	-0.4238800000
C	6.0	3.6036000000	-0.9157500000	-1.1897700000
C	6.0	3.5182200000	-1.4654000000	1.5841000000
C	6.0	2.3728900000	-1.5366300000	0.7993500000
C	6.0	2.4145900000	-1.2678600000	-0.5563300000
B	5.0	0.2496900000	-1.7804900000	-0.0184300000
N	7.0	1.0745300000	-1.8670400000	1.2378800000
N	7.0	1.1475300000	-1.3906600000	-1.1621300000
H	1.0	0.9159300000	-1.2347900000	-2.1531000000
H	1.0	0.7838900000	-2.0984300000	2.1980600000
C	6.0	-1.3031000000	-2.0287200000	-0.1150300000
C	6.0	-2.0376400000	-2.3763300000	1.0255100000
C	6.0	-3.4156700000	-2.5895200000	0.9410700000
C	6.0	-4.0970000000	-2.4599900000	-0.2858700000
C	6.0	-3.3469400000	-2.1105800000	-1.4286100000
C	6.0	-1.9677600000	-1.8982500000	-1.3413300000
H	1.0	5.6050700000	-1.0604300000	1.5990100000
H	1.0	3.4759100000	-1.6749900000	2.6450000000
H	1.0	3.5980000000	-0.7092800000	-2.2525300000
H	1.0	-1.5540800000	-2.4825200000	1.9879300000
H	1.0	-3.9487100000	-2.8538100000	1.8457200000
H	1.0	-3.8005200000	-1.9917400000	-2.3992000000
H	1.0	-1.4271900000	-1.6281100000	-2.2393200000
B	5.0	-0.2491400000	1.6866900000	0.5210700000
C	6.0	1.3041000000	1.9497700000	0.5359400000
C	6.0	1.9764700000	2.2944900000	-0.6432900000
H	1.0	1.4435900000	2.3884700000	-1.5806000000
C	6.0	3.3549800000	2.5211100000	-0.6306500000
H	1.0	3.8391100000	2.7827300000	-1.5631700000
C	6.0	4.0988400000	2.4072500000	0.5610300000
C	6.0	3.4110600000	2.0603300000	1.7429600000
H	1.0	3.9141800000	1.9539800000	2.6902800000
C	6.0	2.0313400000	1.8354400000	1.7277300000
H	1.0	1.5397300000	1.5680000000	2.6542100000
N	7.0	-1.1372000000	1.7524700000	-0.6927500000
H	1.0	-0.8972400000	1.9761200000	-1.6686300000
C	6.0	-2.4087600000	1.4142200000	-0.1868100000
C	6.0	-3.5922900000	1.3233300000	-0.9161400000
H	1.0	-3.5853100000	1.5247000000	-1.9798600000
C	6.0	-4.7799800000	0.9619400000	-0.2394000000
C	6.0	-4.7362000000	0.7046300000	1.1546900000

Am5

H	1.0	-5.6334300000	0.4241700000	1.6914500000
C	6.0	-3.5303700000	0.8026900000	1.8681900000
H	1.0	-3.4977600000	0.6026100000	2.9311700000
C	6.0	-2.3784000000	1.1592000000	1.1735700000
N	7.0	-1.0837900000	1.3004600000	1.7130600000
H	1.0	-0.8007600000	1.1571700000	2.6925000000
C	6.0	-5.5772200000	-2.6901700000	-0.3291100000
N	7.0	-6.3045900000	-2.5766400000	-1.4693300000
O	8.0	-6.1609800000	-2.9895200000	0.7029500000
C	6.0	6.0696000000	-0.4484300000	-1.0985400000
O	8.0	7.2422600000	-0.3345300000	-0.4335600000
O	8.0	6.0691000000	-0.2203200000	-2.2984800000
H	1.0	7.3513700000	-0.4842200000	0.5126200000
H	1.0	-5.8899800000	-2.3262300000	-2.3743000000
H	1.0	-7.3219400000	-2.7422900000	-1.4478000000
C	6.0	5.5771000000	2.6507400000	0.5275000000
N	7.0	6.3649600000	2.5351700000	1.6264700000
O	8.0	6.1027300000	2.9621200000	-0.5319300000
C	6.0	-6.0600800000	0.8462500000	-0.9786900000
O	8.0	-7.1907800000	0.4951300000	-0.3283400000
O	8.0	-6.1005200000	1.0616700000	-2.1815100000
H	1.0	-8.0353600000	0.4085900000	-0.7927200000
H	1.0	6.0013400000	2.2734900000	2.5499800000
H	1.0	7.3782600000	2.7101800000	1.5521300000
